# Medical specialists in LMICs: a systematic review and best-fit framework synthesis of the evidence on their roles and contribution to health systems

**DOI:** 10.1136/bmjgh-2025-018905

**Published:** 2026-01-09

**Authors:** Giuliano Russo, Veena Sriram, Tamara Mulenga Willows, Renata Alonso Miotto, Ana Mocumbi, Mário C Scheffer

**Affiliations:** 1 Wolfson Institute of Population Health, Queen Mary University of London, London, UK; 2 School of Public Policy and Global Affairs, The University of British Columbia, Vancouver, British Columbia, Canada; 3 Queen Mary University of London Faculty of Medicine and Dentistry, London, UK; 4 Preventative Medicine, Universidade de Sao Paulo Faculdade de Medicina, Sao Paulo, Brazil; 5 Instituto Nacional de Saúde, Maputo, Mozambique

**Keywords:** Global Health, Health Personnel, Health systems, Systematic review, Universal Health Care

## Abstract

**Background:**

Medical specialists are integral to the medical workforce and play a pivotal role in referral systems. However, in low-income and middle-income countries (LMICs), there is a perception that specialists often fail to align with local health needs, system capacities and Universal Health Coverage (UHC) objectives.

**Methods:**

A systematic review was conducted in 2024 using a best-fit framework to assess the contributions of specialists to health systems and population health in LMICs. Searches covered eight databases and specialist journals, guided by an expert-validated ‘a priori’ framework for data extraction and analysis. We used the Johanna Briggs Institute critical appraisal tools to assess the quality of the evidence, and the Preferred Reporting Items for Systematic Review and Meta-Analyses guidelines to report the findings. The study protocol was registered in the PROSPERO database (CRD42024572877).

**Findings:**

We found and reviewed 89 studies, focusing on the stock of specialists in LMICs and highlighting a critical shortage of specialists, particularly surgeons, anaesthetists and psychiatrists. Evidence linked specialists’ availability to improved health outcomes such as lives saved through expanded surgical capacity, though broader health system contributions were less clear. Specialists were reported to play key roles in referrals, hospital management, mentoring and research. Governance of their professions was found to be rather uneven across LMICs, with wide differences in specialty types, training curricula, accreditation systems and regulation of private-sector involvement. Reports frequently documented specialists’ engagement with private health markets, revealing blurred boundaries between public and private care. A dynamic market for specialists was also observed, driven by a sustained global demand for their services. However, few policies were found addressing shortages and improving governance of specialties, with existing strategies focusing on task-shifting, clinical training and sharing responsibilities.

**Conclusions:**

This review offers an evidence-based framework for understanding specialists’ roles and health system engagement in LMICs. We discuss the need to reconsider specialists’ deployment, prioritise alignment with UHC goals and enhance governance to optimise their contributions to health systems.

**WHAT IS ALREADY KNOWN ON THIS TOPIC**

- Medical specialists are considered a key component of curative services, scarce and expensive to train.
- In low-income and middle-income countries, they are typically based in urban areas and not easily accessible in the public sector.
- Their contribution to health systems and population health is unclear.

**WHAT THIS STUDY ADDS**

- We found evidence of scarcity of specific specialists, such as surgeons, anaesthetists and psychiatrists.
- Literature was uncovered on some of their functions within health systems, such as referral of cases, hospital management, mentoring and research.
- We found governance of specialties to be uneven across countries, with gaps in regulation of the professions.

**HOW THIS STUDY MIGHT AFFECT RESEARCH, PRACTICE OR POLICY**

- We offer a theoretical, empirically-based framework to conceptualise specialists’ role within health systems.
- We identify areas of further research and policy to align the role of specialists within Universal Health Coverage goals.

## Introduction and background

 Medical specialists—doctors with a recognised advanced qualification and training in a narrower field of medicine—are considered a key component of health systems in low-income and middle-income countries (LMICs), essential to providing the highest quality of services, medical training and professional leadership.^1^ Nowadays, they also already represent the majority of the medical workforce in high-income countries (HICs).^2^

In LMICs, where opportunities for training and resources are scarcer, the number of specialists is steadily increasing, driven by growing demand for specialised services as well as doctors’ own interest to increase their career options.^3^ However, types of medical specialties and ratios of specialists to doctors without a further specialisation vary considerably across such countries.^4^

Although the optimal ratio of specialists to population varies depending on the specific specialty and country’s health needs, a minimum of four specialist anaesthetists per 100 000 population is a widely cited target for achieving a reasonable standard of healthcare.^5^ The WHO estimated a global gap of 2.66 million medical doctors, including generalists, specialists and the undefined.^6^ More broadly, a 2018 Lancet Commission recommended a density of 20 specialist surgeons, anaesthetists and obstetricians per 100 000 population for a minimum standard of surgical care.^4^ As specific specialist needs are complex and country-specific, data from 2019 show that LMICs had significantly fewer specialists (0.5–2.2 per 1000 population) compared with HICs.^7^

From a theoretical standpoint, specialist healthcare services seem ostensibly at odds with LMICs’ specific health needs and health systems; here, primary care services and cost-effective health staff are key for working closer to communities, often in rural areas, and realising Universal Health Coverage (UHC) goals.^8^ The WHO talks of ‘integrated primary care systems that refer to models of care that extend from community to tertiary’, in which specialists would contribute to national health systems.^9^ However, most specialists are in fact known to work predominantly in tertiary care level hospitals in urban settings and engage often with the less affordable private sector market.^10^ Consistently, evidence from the USA suggests that health systems relying excessively on specialists would neither be cost efficient nor improve population health outcomes.^11^

Although primary care is firmly at the centre of UHC and key to strengthening health systems in LMICs,^12^ attention has also been drawn to the equity implications of basic care-only-focused health systems, and the perils of creating lower-quality services for the poor.^13^ A growing consensus exists that primary healthcare (PHC) also comprises essential specialist services, such as life-saving, cost-effective surgical procedures^14^ and emergency care.^15^ Scholars from LMICs have also contested the goal of securing universal coverage of basic services as opposed to access to universal systems,^16^ and recognised the importance of specialised services to realise Sustainable Development Goals and health rights,^17^ and to consolidate the professions through adequate planning.^18^

From a sociology of professions perspective, specialisation is often understood as a central trajectory around which the medical profession continues to develop; this process reflects how roles, expertise and professional identities in medicine increasingly evolve through specialised pathways.^2^ However, leaving the shaping of medical specialties entirely to market forces may not serve the broader public interest. To ensure that specialisation grows in ways aligned with national health priorities, interventions from decision makers—such as governments, regulatory agencies or professional colleges—may be necessary.

The objective of this literature review is to take stock of the arguments, theoretical aspects and evidence on the contributions of medical specialists to population health and health systems in LMICs. To lend substance to such arguments, we explore the specific evidence on specialists’ contribution to health systems strengthening across its pillars^19^ and to provision of services in LMICs.

The research questions to be answered by this review are as follows:

1. What is the contribution of medical specialists to population health and health outcomes in LMICs?
2. Within national healthcare systems, what is their contribution to provision of services and system strengthening in such contexts?
3. What are the factors and drivers of specialists’ contributions in LMICs?

## Methods

We adopted a ‘best-fit framework’ synthesis approach for our systematic review, a qualitative method that uses a pre-existing theory or conceptual model—the ‘framework’—to organise and analyse existing evidence on the topic of interest.^20^ Such a framework was used to organise the initial searches, was iteratively refined to accommodate the empirical evidence uncovered and helped develop a theoretical interpretation of our findings. The a priori framework was used both to identify the relevant literature and then to extract and synthesise the different types of evidence, as per similar reviews.^21^

**Figure 1:**
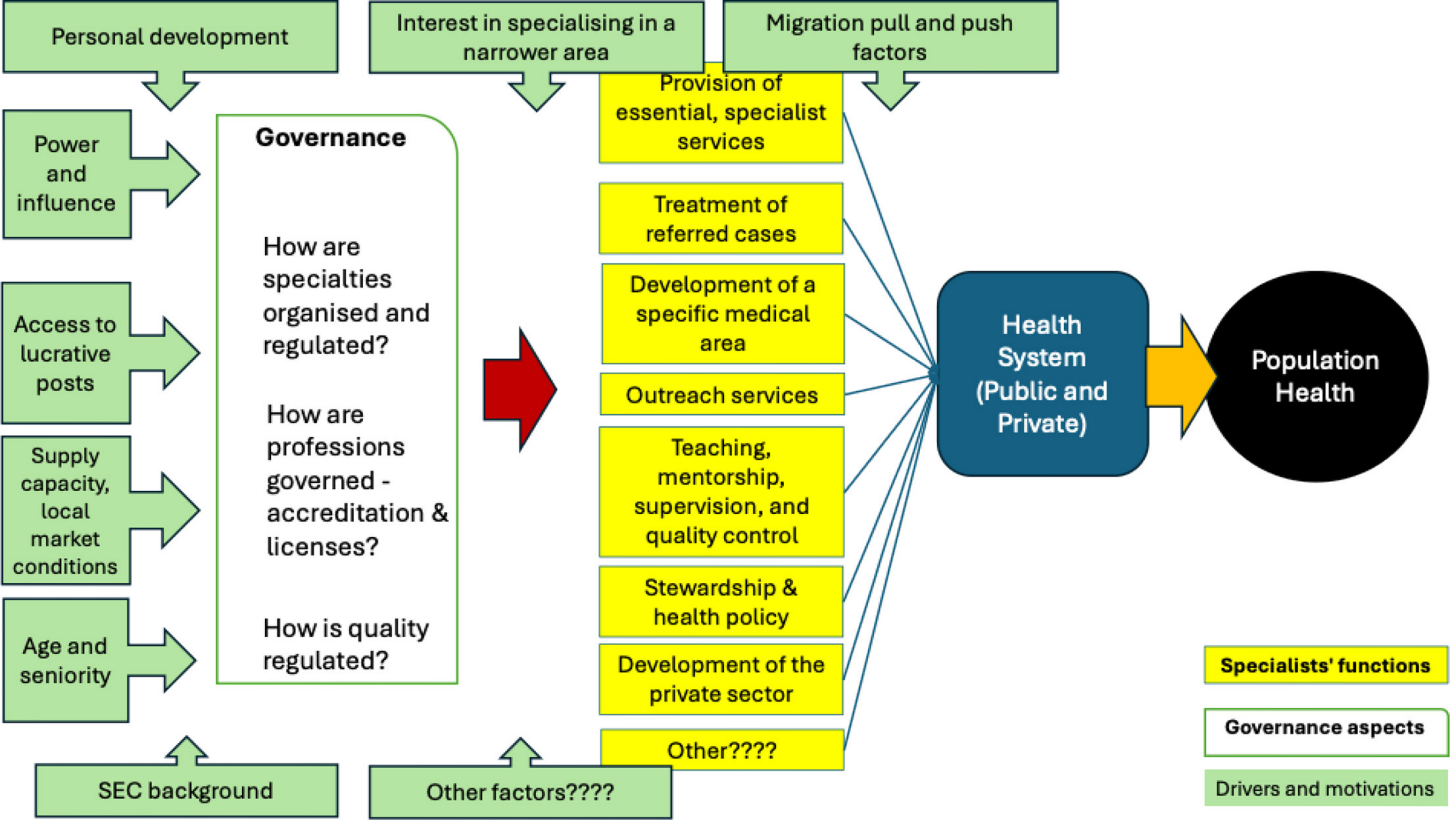
A priori framework on contribution and governance of specialist doctors for health system strengthening in LMICs. LMICs, low-income and middle-income countries.

Our a priori framework draws from previous conceptualisations of specialists’ contribution to health systems strengthening^1 2 17^ and was validated by a panel of experts and patient representatives (see the Patients and public involvement section below). Our framework identifies specific functions that specialists typically perform that contribute to health system strengthening and population health—from provision of specialistic services, to referral for complicated cases, or contribution to medical research, teaching and policy elaboration. We understand such functions are shaped by institutional factors such as the existing governance, organisation and regulation of specialties in the country, as well as by individual doctors’ socioeconomic characteristics. Underlying health labour market conditions and specialists’ motivation to choose their specific area of practice are likely to influence both the governance of the professions and functions carried out by specialists in different countries (figure 1).

### Patient and public involvement

Prior to conducting the searches, a meeting was organised in São Paulo (Brazil) to consult health system experts, policymakers and patients’ representatives on the objectives of our study (online supplemental table S1). The study protocol was registered in the PROSPERO database after the elaboration of the a priori framework and before the formal screening of the identified studies^22^—registration number CRD42024572877.

Our outcomes of interest referred to the relevant parts of the conceptual framework (see figure 1), in relation to specialists’ impact on population health and health systems, their functions as described in the literature such as the WHO building blocks—referral of services, teaching, research, etc—drivers of the professions, and governance of specialties (see online supplemental table S2 for the full list of outcomes of interest and search terms). Our search strategy focused on medical specialists rather than on other health workers or doctors, in the belief such cadres play a different role in health systems.^2^

### Search strategy

We searched eight relevant databases across the health, health systems and economic literature (PubMed; ISI Web of Science—core collection; Scopus; Cochrane Library; PDQ-Evidence; Health Evidence.org; Scielo and Econ Lit). We separately searched the websites of specialist journals publishing on our topic, such as The Lancet Global Health, BMJ Global Health, Social Science and Medicine, Health Policy and Planning, Human Resources for Health, and The International Journal of Health Policy and Management.

The challenge of our search strategy was to identify literature focusing specifically on medical specialists in LMICs and not on other health cadres. Searches for specialist doctors included general terms such as: “specialist*”, “medical special*” as well as terms of specific specialties highlighted as ‘essential’ for providing universal health coverage in LMICs by the Lancet Non-Communicable Disease (NCDI) Poverty Commission^23^ (such as Gynaecolog* OR Cardiothoracic surg* OR Surg* OR “Internal medicine” OR Critical care OR Palliative care OR Oncolog* OR Cardiolog* OR Ophthalmolog* OR Patholog* OR Radiolog* OR Rehabilitation OR “Orthopaedic surg*” OR Otorhinolaryngolog* OR Neurolog* OR Psychiatr* OR Paediatric* OR Obstetric*) – see online supplemental table S2 for detailed search strategy and terms.

We used the OECD Development Assistance Committee’s list of Official Development Assistance recipients to identify LMICs, which as of 2024 includes 47 least-developed and low-income countries, 35 lower-middle income and 59 upper-middle income countries and territories.^24^

### Eligibility criteria

We included academic literature published in English, Portuguese, French, Spanish or Italian, based on primary empirical evidence from quantitative, qualitative or mixed-methods studies, or original analysis of secondary data. We excluded: unpublished study reports, papers published in non-peer-reviewed journals, commentaries and opinion pieces, non-systematic reviews, studies not presenting evidence as defined by the adopted tools (see Data management, selection process and quality assessment section below), and papers with no title or abstract in any of the five languages above.

For the purposes of this review, drawing from the medical science literature,^11 25 26^ we adopted the following definition of specialist physicians: ‘Medical doctors with a recognised advanced qualification in a narrower field of the medical science, who operate primarily from a hospital setting’. Conversely, generalist doctors were defined as those practising in primary care closer to communities rather than in hospital settings, with undifferentiated experience across multiple clinical specialties. It is worth noting that in many LMICs, such primary care doctors lack a further qualification beyond the statutory medical degree.

We considered publications focusing on: (1) organisation, regulation and policies on medical specialties and specialty services in LMICs; (2) contribution of specialty services to health outcomes and population health; (3) population access to specialty services; (4) medical specialists’ functions and performance in LMICs and (5) contribution of medical specialists to health system strengthening (see the inclusion and exclusion criteria section in online supplemental table S3).

### Data management, selection process and quality assessment

Bibliographic references were managed in the open-source Zotero software; characteristics of each study and respective key findings were organised in an Excel database and analysed through pivot dynamic tables.

After being shared through Zotero software and deduplicated, with a view to fine-tuning our searches, titles-and-abstracts screening was performed on a random sample of 5% of the entries by one researcher with systematic review experience to test accuracy and specificity of the searches. Search terms were refined accordingly, and a full titles-and-abstracts screening was subsequently conducted by three authors (level 1 screening). Level 2 screening (full-text assessment) was divided among three authors. All the included and excluded papers were checked by two authors.

The database searches were conducted by TMW and RAM. The pilot screening and the specific journals screening were conducted by GR to identify sources that may be of relevance to our study objectives. Levels 1 and 2 screening were conducted by TMW, RAM and GR, with each reviewer independently looking at the abstract and full text to assess whether the source met the inclusion criteria. The level 2 reviewers returned three possible decisions: ‘included,’ ‘excluded’ or ‘uncertain: seek further information.’ For each source that was excluded, the reviewers recorded a reason related to the inclusion and exclusion criteria. For each source that was judged as ‘seek further information’, a third reviewer (GR) made an independent assessment regarding whether the sources should be included.

Where studies met the eligibility criteria, their methods’ quality was also appraised using the Joanna Briggs Institute’s critical appraisal tools covering 12 different types of study designs.^27^ The reviewers who performed the Level 2 screenings also performed this assessment, using one checklist per paper, appropriate for the study design.

### Data collection and data items

A data extraction form was populated based on the a priori conceptual framework and covering key methodological and contextual features (such as type of study, type of evidence, type of specialist, impact on health system) for the included studies. The rubrics of this form guided the extraction of relevant empirical evidence and the refinement of the a priori framework. We piloted the data extraction form ahead of level 1 screening and adjusted the form accordingly. The contents of papers were extracted independently by three reviewers, without the use of a specific coding structure.

The quantitative and qualitative findings from the included papers were coded and synthesised against the a priori framework, which was refined throughout the process to produce the ‘best-fit’ framework.^20^ Whenever the empirical evidence did not match the categories of the a priori framework, we revised the framework to accommodate emerging themes and diverging evidence, particularly for different contexts.^21^

Because of its heterogeneity, quantitative information was not pooled across the selected papers. When studies reported different estimates for density of specialists, we reported the conflicting figures.

## Findings

**Figure 2:**
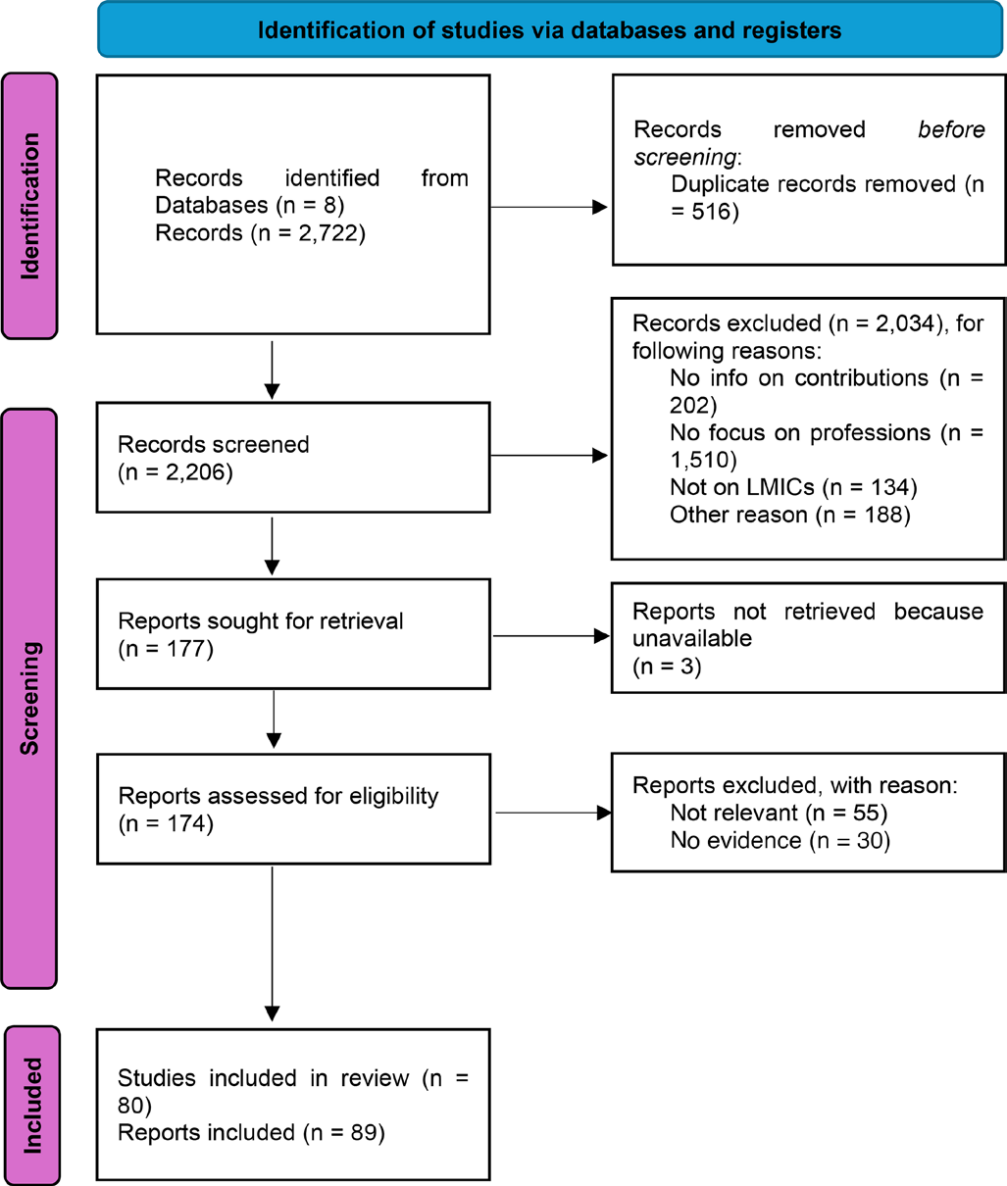
PRISMA flow diagram for searches, assessment and selection of the literature. LMICs, low-income and middle-income countries; PRISMA, Preferred Reporting Items for Systematic Reviews and Meta-Analyses.

Our initial searches generated 2722 records from 8 databases. After removing duplicates and level 1 screening, we assessed 174 records for eligibility. Of these, 55 records were excluded for not meeting our inclusion criteria, and 30 were excluded for not presenting evidence, based on the JBI’s assessment criteria. In total, 89 studies were included in this systematic review (figure 2). See a list of the papers excluded and the reasons for exclusion in online supplemental table S4.

**Figure 3:**
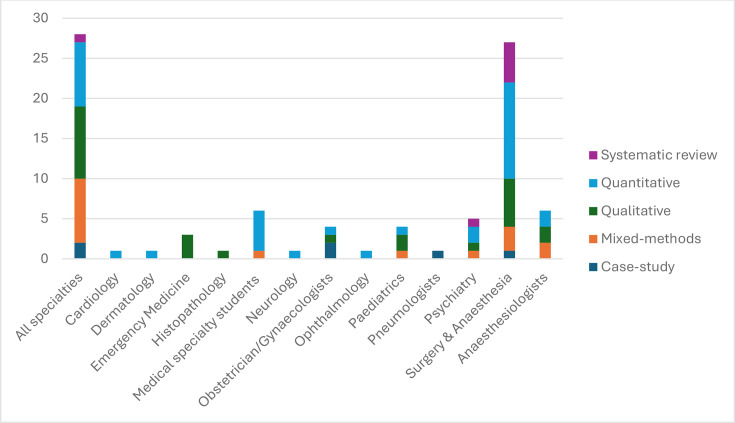
Papers included in the review, by medical specialty and methods.

The papers retained focused either on low-income countries in general, or on specific countries from sub-Saharan Africa (SSA). Although many papers (28) discussed medical specialists in general, 27 focused specifically on surgeons and anaesthetists jointly as parts of surgical teams, and 6 on anaesthetists only. Medical specialty students were the subject of six papers, psychiatrists of five, followed by other specialties like obstetrics/gynaecology and paediatrics (four papers each) (see figure 3).

Of the papers eventually included, 35 were based on quantitative analysis of census data, surveys or discrete choice experiments and modelling exercises. There were 25 qualitative papers, 16 mixed-methods and 6 case studies reporting on specific medical specialty development experiences. As per the Best Fit Framework methodology,^20^ we used our conceptual framework to synthesise the evidence; four main bodies of evidence emerged related to the functions and influencing factors as hypothesised in our framework, covering stock and distribution of specialists workforce in low-income settings, their contribution to health systems and population health, their governance and their interaction with health markets (see table 1 and online supplemental table S5).

**Table 1:** Summary findings of the framework’s domains and supporting references

Domain of the framework	Key findings	Key bibliographic references
Stock and scarcity of specialists	Substantial evidence on low numbers of specialists in many LMICs, and lack of surgeons, anaesthetists and psychiatrists from Sub-Saharan countries and Southeast Asia. Substantial evidence on lack of paediatricians and paediatric surgeons in sub-Saharan countries. Some evidence on the lack of cardiologists and paediatrists from China. Some evidence of lack of specialists in Eastern Europe.	Meara *et al* 2015, Daniels *et al* 2020, Bulamba *et al* 2022^4 28 30^ (DeVries 2022a) (English *et al* 2020a) van Rensburg *et al* 2022, Fitts *et al* 2020 Gong *et al* 2016 (Zhang *et al* 2019).^31^,^3354 65^
Contribution to population health	Consolidated evidence that increasing numbers of surgical specialists (both surgeons and anaesthetists) would reduce the global burden of disease and maternal mortality in LMICs. Density of specialists is associated with reduced amenable mortality in some South American countries (Mexico and Brazil). Lack of oncologists compounds the high mortality rates for cancers in Sub-Saharan countries.	Daniels *et al* 2020,^28^ Falk *et al* 2020,^35^ Mock *et al* 2015,^36^ Meara *et al* 2015^4^ Nikoloski *et al* 2021,^37^ Davies *et al* 2018.^5^ Scheffer *et al* 2017.^38^ Vilaly *et al* 2021.^39^ Erem *et al* 2020^40^
Specialists’ roles in health systems	Evidence that specialists organise and lead systems of teaching, research and health policy making Specialists are integral for the referral of complex cases and for the development of private healthcare sector. Crucial debate on where exactly specialists and specialised services should be placed within health systems, whether at the top of curative services in urban areas, or closer to rural communities, as per in first referral hospitals. Laboratory, scan and analysis specialists are essential for diagnostic services. Specialists often take responsibilities for managing hospital departments, although with consequences for the development of other health workers.	(Sriram and Bennett 2020a).^2^ Noormahomed *et al* 2013.^41^ Wu *et al* 2017^42^ Russo, de Sousa, *et al* 2014;^43^ Russo, McPake, *et al* 2014a).^10^ English *et al* 2024.^45^ Atiyeh *et al* 2010.^46^ Ruggunan and Singh 2013.^47^ Jones and Fulop 2021,^48^ Badejo *et al* 2020.^49^ Binyaruka *et al* 2021^50^
Governance and regulation of specialties	Rich reports that many aspects of sub-specialties are not formalised or regulated in many LMICs, particularly in India and Morocco. Evidence from China that financial pressure on specialists has distorted their incentives and governance of hospitals. Perceptions of weak regulations of specialists in Latin America, and on conflicts of interest with private sector activities in South Africa.	Haastrup *et al* 2015,^52^ Kempthorne *et al* 2017^53^ (DeVries 2022b).^54^ Belrhiti *et al* 2021,^59^ Sriram *et al* 2018^7^ (Sriram and Bennett 2020b).^2^ Wu 2018,^55^ Gong *et al* 2016,^33^ He *et al* 2020.^56^ Pei and Xiao 2011^57^ Nigenda and Muños 2015^18^ (Russo, McPake, *et al* 2014b).^10^ Ashmore and Gilson 2015^58^
Specialists and the market for specialised healthcare services	Rich evidence on specialists’ involvement with private services from Asia, Africa and South America. Evidence that domestic and international market forces shape the availability of public services and governance of specialties in LMICs. Losses of specialists to internal private markets, and migration to higher-income countries. Lack of regulation for specialists’ dual practice. Some evidence that international migration from doctors-rich countries like Cuba and the former Soviet countries can help mitigate shortages of specialists.	Bayat *et al* 2018^60^ Miotto *et al* 2018^61^ (Russo, McPake, *et al* 2014a).^10^ Karekezi *et al* 2020,^63^ Janse van Rensburg *et al* 2018.^64^ Meara *et al*^4^ 2015 (English *et al* 2020b).^65^ Botezat and Moraru 2020,^66^ Oman *et al* 2012.^67^ Abukmail and Albarqouni 2021.^68^ Vio 2006^69^ (DeVries 2022b).^54^

Source: online supplementalappendix 5–table S5: Full evidence table.

LMICs, low-income and middle-income countries.

### Evidence on stock and distribution of specialists

As many as 36 papers provided (mostly quantitative) evidence on scarcity of medical specialists in low-income countries, with many of such works (19) focusing on surgeons and anaesthetists (table 1 and online supplemental table S5). Key papers for this area were the ones originating from the Lancet Commission on Global Surgery,^4^ that showed how, in low-income and lower-middle-income countries, 9 people out of 10 cannot access basic surgical care. Meara *et al* demonstrate how 143 million additional surgical procedures would be needed in LMICs each year to save lives and prevent disability, as only 6% of the world’s procedures occur in the poorest countries. The commission calculated that unmet surgical needs are the greatest in eastern, western and central SSA, and south Asia.^4^

Building on the Lancet Commission’s estimates, in their modelling study, Daniels *et al* calculated that 94.9% of world countries have a density of surgeons lower than 20 surgeons/100 000 population, with the SSA region, East Asia and Pacific, Latin America and Caribbean, Middle East and North Africa, and South Asia the regions with more countries with suboptimal surgeons’ density (<20 surgeons/100 000 population).^28^ In the 20 most populous countries evaluated (154, including both HICs and LMICs), the lowest surgeon densities were observed for Nigeria (1.4), Uganda (0.9), Ethiopia (0.6), Tanzania (0.3) and Democratic Republic of the Congo (0.2).

Henry *et al* quantified the surgical capacity in Malawi’s hospitals in 2014 and found that, of the 370 surgical workers they surveyed, 92.7% were non-surgeons and 77% were clinical officers^29^; of the 109 anaesthesia providers, 95.4% were non-physician anaesthetists. Non-surgeons and anaesthesia clinical officers were the only providers of surgical services and anaesthetic services in 85% and 88.9% of hospitals, respectively, with no specialist serving in the district hospitals. Another study focusing on anaesthesia found that Uganda has 68 specialist physicians and approximately 600 non-physician anaesthetist providers for 43 million people, with most providers located in Kampala and other urban centres.^30^

As for the scarcity of psychiatry specialists, Janse van Rensburg *et al* found in South Africa that the number of active qualified psychiatrists in 2019 working in the public and private sectors was 850, giving a ratio of 1.53 of psychiatrists per 100 000 population.^31^ Furthermore, of the total number of psychiatrists, close to 80% (n=625) were working full time in the private sector, while only about 20% (n=168) were working full time in the public sector. Another study on psychiatry capacity in Sierra Leone^32^ calculated a 98% treatment gap for severe mental disorders nationwide, as there were only two psychiatrists and 20 psychiatric nurses in the country in 2020.

In their survey of the national cardiology workforce in China, Gong and Huo found there were 25 240 cardiologists in the mainland in 2016; the ratio to population was 19 per million, which compares poorly with the ratios of around 50 per million in HICs.^33^ Another study on paediatricians in China^34^ talks of a full-blown crisis facing paediatric care services in China, with a single physician typically responsible for 80 to 100 visits per day in some tertiary hospitals, with an average of 50 work hours per week. The same study reports how in 2006, the Paediatric Society of Chinese Medical Doctors Association reported 831 incidents of serious medical violence, including 319 attacks on paediatricians, with many being beaten to death or disabled.

### Specialists’ contribution to population health

Overall, we found consolidated evidence on the impact of specialist services on population health in LMICs, particularly for surgeons, anaesthetists, obstetricians and cardiologists (table 1 and the full evidence online supplemental table S5).

Daniels *et al* report that 90% of maternal mortality could be averted worldwide with timely surgical intervention, 32% of the global burden of disease requires surgical decision making; and that 77.2 million disability-adjusted life-years could be averted.^28^ Falk *et al*’s literature review finds evidence that up to 32% of the global burden of disease is surgical in nature, but 5 billion people on our planet have minimal access to surgical services and 2 billion have no access to basic surgical services.^35^ Such findings are supported by Mock *et al*, who calculated that provision of essential surgical procedures would avert about 1.5 million deaths a year, or 6%–7% of all avertable deaths in LMICs.^36^

Based on the Lancet Commission’s global surgery dataset, Meara *et al* calculate there are particularly steep improvements in maternal survival when specialist providers per 100 000 population are improved from 0 to 20; beyond densities of 40 per 100 000 population, gains are still present, but the gradient of the curve is flatter, displaying diminishing returns of lives saved.^4^

A doctors’ panel data study from Mexico^37^ found the density of specialist physicians to be negatively associated with amenable mortality rate, as a unit increase in density of such doctors was associated with a 0.5% reduction in the overall amenable mortality. Also, Davies *et al* found an inverse linear relationship between density of anaesthesia providers and maternal mortality in LMICs.^5^ On the same theme, Scheffer *et al* attribute the reduction of maternal mortality to the improved rates of surgical specialists per population in Brazil.^38^

Vilaly’s study on paediatric surgery reports that, in SSA, the burden of surgical disease is estimated between 115.3 million and 131.8 million in children under 15 years of age, and that 85% of children in LMICs will have a surgically treatable condition by the age of 15 years.^39^ A smaller study on gynaecologic oncology in Ghana^40^ reports that, in 2018, cervical cancer was the leading cancer in half of the countries in SSA and responsible for 21.7% of all cancer deaths in SSA women; a lack of trained gynaecologic oncology specialists contributes to poor patient outcomes, with a critical need for specialists to lead diagnosis, imaging and surgical and oncologic care.

### Specialists’ role in national health systems

The evidence on medical specialists’ contribution to health systems is comparatively patchier and fragmented (table 1 and online supplemental table S5). Sriram and Bennett mention how, in high- as well as low-income settings, specialists help organise and lead systems of academic medicine, advance teaching and research, and play a major role in developing the sector’s policies.^2^ Such a view is corroborated by a case study from Mozambique^41^ where specialists initially employed only for teaching and supervision later committed to developing the national medical research system, mentorship in bioclinical science, regional networks for mutual support, attracting research funds and establishing national research ethics institutional capacity.

Specialist services are typically an essential part of national curative systems; in China, after the 2009 reform, the health system has become crucially hinged to hospitals and specialists because of the current insurance-based financing system. However, Wu *et al*^42^ show how such centrality has come at the expense of primary care services, with patients trying to bypass such parts of the system perceived as lower quality and seek directly more sophisticated and more expensive specialist services.^42^

Interviews and survey-based studies from Cape Verde, Guinea-Bissau and Mozambique show how specialists are crucial for the development of private services in urban areas, expanding availability and diversity of healthcare services, catering for different sections of the population, and therefore increasing overall patients’ welfare.^10 43^

Sriram and Bennett identify a recurrent trend in urban areas within LMICs with better specialist supply, experiencing a ‘bypassing’ of primary care, with patients directly seeking specialist care.^2^ Such a phenomenon, where specialists work outside or in parallel to a primary care-led healthcare system, raises major concerns around efficiency, overmedicalisation, affordability and possibly quality.^44^

A debate exists on whether some specialist, life-saving services should be located closer to communities, such as for basic surgical interventions in First (district) Referral Hospitals. English *et al*^45^ make the argument that overproduction of and overreliance on specialists in SSA countries risks fragmenting care and undermining the functions of First Referral Hospitals, reducing the system’s resilience and its ability to deal with multi-morbidity. In the same vein, Atiyeh *et al* review argues that for years, surgeries were not planned considering similarities and disparities between developed or urban areas and rural and remote areas,^46^ and concludes that in LMICs, surgery might have been thought to lie outside the scope of public health.

Less visible specialties such as histopathology are also essential for medical tests and the functioning of laboratories, without which diagnostic systems could not truly work. To this respect, a qualitative study from KwaZulu-Natal, South Africa^47^ describes how such specialty is perceived as comparatively less prestigious, its specialists subject to the attraction of the private sector, and therefore finding it particularly difficult to recruit and retain specialists in key parts of public health systems.

Specialists very often take charge of management and oversight responsibility.^48^ However, in a case study from Nigeria, Badejo *et al* see such medical dominance as deleterious to collaboration across different hospital professions,^49^ while Binyaruka *et al* show that such dominance helps specialists in Tanzania to access informal payments.^50^

### Evidence on the governance of specialties

In our review, we found relatively rich evidence on the ‘level’ of governance of specialties as a whole or in their specific disciplines (policy, institutions and their regulatory roles including government sanctioned professional bodies). Although less common, we also located examples of ‘meso and micro-level governance’ of specialists as they practise at the meso (subnational) or even micro level (facility).

The literature highlights different functions involved in governance of medical specialties, such as identification/approval of specialties; seat allotment/forecasting; approval of training programmes; curricular development; system integration and continued medical education.^48 51^ In their study of ophthalmologists in South Africa, Haastrup *et al* highlight the absence of official governance systems for the specialty, as specialists are expected to self-govern to maintain the necessary quality of services.^52^ Looking at the global anaesthesia workforce, Kempthorne *et al* find training availability and duration of courses to be inconsistent across world regions.^53^ DeVries finds that subspecialties such as paediatric urology, although being consolidated and registering an excess of professionals in high and middle-income countries, are absent in Africa, as urological care on the continent is provided by general surgeons or paediatric surgeons.^54^

In China, the 2009 health reform created hospital competition, and specialists now have to generate revenues for their hospitals by selling drugs and medical appointments; according to Wu,^55^ this would be distorting specialists’ governance, incentives and practice. In their survey of the cardiology workforce in China,^33^ Gong and Huo point out the country only has national medical licence certification and interventional cardiology licence certification, but broader cardiovascular disease licence certification did not exist until 2016. As for child and adolescent psychiatry, He *et al* found how the subspecialty is not yet comprehensively regulated in the country, and specialised child psychiatrists are not the only medical professionals providing their service to the population as this role is also fulfilled by adult psychiatrists and other physicians.^56^ As for emergency medicine (EM), a specialty that is underdeveloped in China, specialists are recruited from non-specialist doctors to deliver emergency care in their own departments^57^; with a view to attracting customers under the new health insurance system, hospitals have built new EM departments and actively advertise such services.

Nigenda and Muños report a generalised perception of weak regulation of medical training in Mexico, where specialist physicians are predominantly trained at public sector health institutions and subject to political influences from the government and powerful medical councils.^18^ In most countries, public doctors’ engagement with the private sector (a phenomenon known as dual practice) is only lightly regulated^10^; in South Africa, where the practice should be governed by the official Remuneration for Work Outside the Public Service (RWOPS) regulation, Ashmore and Gilson found that doctors do not always ask permission for dual practice.^58^

For Belrhiti *et al*, weak governance of the medical professions in Morocco created an opportunity for unofficial professional hierarchies to form within hospitals.^59^ Such arguments are echoed by a study from India on EM,^7^ and arriving at the conclusion that regulation of medical specialisation is only emerging aspect of health sector governance in LMICs, covering regulatory functions such as recognised categories for specialisation, types of practitioners who may acquire skills in certain specialisations, approval or accreditation of specialist training programmes, specialist licensing, registration and re-registration, continuing medical education and complaint investigations. Within such a context of weak governance, for Sriram and Bennett, the number of specialties in LMICs is expanding because of specialists’ influence on policy and policy-makers.^2^

### Specialists and the markets

The literature shows the relation between specialists and market forces is complex and multidimensional; Zhang *et al* talk about an unmet demand for paediatric services in China,^34^ Nigenda and Muños about the misalignment between types and numbers of specialists trained, market demand and opportunities for employment in Mexico.^18^ For Sriram and Bennett, market forces can make it difficult to deploy and retain specialists where they are needed the most, but the market for specialist services is also likely to accompany economic growth of lower-income countries.^2^ For Russo *et al*, local market conditions are a key determinant of specialists’ engagement with different existing forms of private services.^10^

We found comprehensive evidence of specialists’ participation in private services, either through the formal private sector or within their own public institutions. In Iran, Bayat *et al* found that 48% of public sector specialists were engaged in dual practice.^60^ In Brazil, a cross-sectional national survey^61^ established that 51.45% were currently working in both the public and private services, while 26.95% and 21.58% were working exclusively in the private and public sectors, respectively; dual practitioners were mostly middle-aged, male specialists with 10 to 30 years of medical practice. We found also that, with few exceptions (such as RWOPS in South Africa and special clinics in Mozambique), dual practice in LMICs is largely ignored by the law, therefore not explicitly permitted or banned.^62^

A study on neurosurgeons in LICs^63^ shows that over half of neurosurgeons trained in sub-Sahara Africa return to SSA countries for professional practice and are involved in dual practice, and less than 10% work exclusively in private practice. Janse van Rensburg *et al*^31^ report that most psychiatrists in South Africa (80%) work full-time in the private sector. Those in public are joint-appointment academics or on RWOPS contracts.^64^

We found a wealth of evidence on the influence of market forces on specialists’ location decisions, both within and outside low-income countries. For the 2015 Lancet Commission on Global Surgery, specialist providers are often concentrated in urban areas, which have more surgical infrastructure and better-equipped tertiary care centres than do rural areas.^4^ English *et al* report that in Kenya, despite the government’s efforts to increase the supply of specialists, there has been no concern with the retention of professionals in the public sector, and it is estimated that 60% of paediatricians and 40% of non-specialists are employed in the private sector.^65^

On the other hand, there is substantial evidence of the existence of a dynamic global market for medical specialists, with doctors pushed and pulled to foreign markets attracted by favourable salary differentials, security and working conditions (table 1). A study on the diaspora of specialist doctors from Romania^66^ finds that most doctors who want to leave the country currently live in underserved or rural areas, with cardiology, surgery, psychiatry, radiology or anaesthetics the most requested specialists. For Fijian specialists, the attraction of the Australian and New Zealand markets is difficult to resist, particularly for Indo-Fijian doctors looking to escape the island country’s political and economic insecurity.^67^ For Palestinian specialists trained abroad,^68^ only a minority intends to return to Palestine, while most intend to return after a few years of practice abroad, with the UK as the preferred destination.

However, Vio also talks about an oversupply of specialists from former Soviet countries and Cuba, that was exploited to staff rural hospitals in Mozambique using doctors recruited internationally.^69^ In the same vein, De Vries discusses world market unbalance in the supply of paediatric urologists, pointing to an excess of professionals in some HICs and MICs (including Latin America), and complete lack of such specialists in Africa.^54^

## Discussion

We found and reviewed 89 studies examining medical specialties, specialists, population health and health systems in LMICs. A significant portion of the literature highlights an absolute or relative scarcity of specialists, particularly surgeons, anaesthetists and psychiatrists. Several studies explored the link between specialists’ availability and burden of disease. Evidence on the broader contributions of specialists to health systems remains less robust; however, a few studies did mention key system functions by specialists, such as referrals for curative care, hospital management, oversight, training and quality of junior colleagues, private sector development and research. A consensus exists that governance of specialties and specialists in LMICs is highly heterogeneous—particularly in SSA countries—characterised by substantial variation in number and types of specialties, diversity of training curricula and accreditation systems, and limited regulation of private sector involvement.

Many reports document specialists’ engagement with private health markets, often revealing blurred boundaries between public and private healthcare services. We found evidence of a dynamic global market for specialist medical services, driven by the movement of doctors across national labour markets. The growing corporatisation within particular domestic health labour markets, such as India, would be one factor impacting the growth and distribution of specialists.^70^

Our expert-driven, a priori framework was broadly validated by the evidence identified in the review, though further refinement appears necessary. The original framework effectively captured specialists’ roles and highlighted the linkages between external influences, health sector governance, healthcare systems and population health outcomes. However, the maturity and stage of development of health systems seem to play a greater role in shaping specialties than initially anticipated. While these factors are partly reflected in the ‘supply capacity and local market conditions’ component of our framework, additional research is needed to clarify the specific mechanisms through which health system consolidation influences the governance of specialties.

We also identified a few unresolved ‘tensions’ in the literature regarding: (a) the cost-effectiveness and equity aspects of investing in specialty services in LMICs; (b) the need for some specialists to be deployed at peripheral levels and (c) the consequences of specialists’ dual practice for public services (see the Table of Tensions in online supplemental table 6). We discuss below such key tensions.

We found no clear evidence on the mechanisms through which regulation of medical specialties would influence the development of doctors’ roles in health systems or population health. Additionally, the literature did not explicitly examine the contribution of specialists’ private services to population health. Given the growing recognition of private healthcare providers’ role for service availability and achievement of UHC,^71^,^73^ a more focused and narrowly scoped review may be necessary to explore the relationship between specialists’ private services and population health outcomes.

It looks like governance of medical specialties within national health systems has remained an underexamined area of health governance, as evidenced by the relatively few papers addressing this topic in our review. The papers we found provide an emerging picture of the complexity of institutional frameworks and organisations involved in governance of specialties,^7^ the relationships between national and state governments, statutory and/or self-regulatory mechanisms. For the literature from HICs,^74^ problems of accountability, accreditation, standards of treatment, and ultimately, quality of service are likely to arise for those specialties and subspecialties that have not consolidated their jurisdiction as a (specific) profession, and specialists share their area of practice with others. Further research is needed to better understand the institutional frameworks shaping the development and oversight of medical specialties. These issues are also salient to questions of power and governance processes, given the evidence of elitism and professional hierarchies that emerged within the review,^75^ exacerbated by the challenges of regulating private healthcare markets in many LMICs.^76^

A key debate emerging from our review concerns where life-saving specialist services should be made available within health systems (see online supplemental table 6). Due to their complexity and high costs, these services are often placed in urban locations, at the top of the curative care pyramid.^1^ However, the *Lancet Global Surgery Commission* highlights that surgical interventions for treatable conditions—such as appendicitis, hernia, fractures and obstructed labour—should be included in national UHC programmes.^4^ By the same token, there is ongoing advocacy for integrating psychiatric services into PHC, enabling early identification and treatment at the community level.^77 78^ Yet, at the district level, such services are seldom delivered by specialists and are more commonly managed by surgical technical officers^79^ or community psychosocial workers.^80^ We propose a re-evaluation of where and by whom less complex specialist services are delivered within health systems. First-referral (district) hospitals could serve as their natural hub, offering care closer to communities in less complex settings and improving access for all.^45 81^

Some of our references emphasised the barriers specialists encounter when embarking on their chosen careers in LMICs. In countries like China, those wanting to specialise in child psychiatry do not often have appropriate training placements to allow them to become competent in their chosen specialty.^56^ Other scholars have written extensively about the difficulties surgeons face when training and attempting to practise across many countries in SSA.^82^ We consider the barriers faced by specialists to train in their chosen specialty and practise are likely to work alongside market forces to push specialists to regions where both educational and financial opportunities align. The primary challenge, therefore, lies in retaining at least some specialist expertise within domestic public health systems.

Our review highlighted the significant influence of market forces in drawing specialists away from public health systems in LMICs, either to wealthier nations or to more lucrative opportunities within the domestic private sector. If governments are unable to offer competitive, near-market salaries for highly qualified health professionals, it seems unrealistic to expect that regulatory measures such as bans would effectively attract specialists to the public sector.^83^ Despite the potential risks of conflict of interest and the possibility of public resources being diverted for private gain,^84^ a practical approach may involve allowing limited private practice in exchange for a defined public service commitment, which could better balance these competing demands. Some countries are currently piloting the strategic purchasing of specialist services in large public hospitals.^85^ Expanding such initiatives beyond the clinical sphere to encompass other essential health system functions could be beneficial. Encouraging specialists to contribute to national training, mentorship and quality control programmes may yield great long-term benefits. In return, allowing these specialists to engage in private clinical practice could serve as an incentive, creating a mutually beneficial arrangement.

Despite the plentiful evidence on scarcity of specialists and lack of governance of medical specialties in LMICs, we encounter only limited literature on policy options to mitigate such gaps. Admittedly, there are studies on effectiveness and barriers of task-shifting specialist functions to physicians and clinical officers (also known as ‘task-sharing’), as a way to mitigate scarcity and weak governance,^86^ particularly as the practice has already been adopted in many LMICs for obstetrical and basic surgery services.^79^ Other reports suggest alternative training modalities for specialists within and outside LMICs, with different retention rates.^87^ However, to our eyes, such measures appear to at least in part side-step the broader question of whether specialists are a priority investment for health systems in LMICs, and how these should be governed to support the achievement of UHC goals.

We did not find much evidence on the opportunity costs associated with investing in hospital-based specialisation. Although some of the selected studies did focus on the cost-effectiveness of training doctors inside LMICs or abroad,^88^ we believe it would be important to assess the economic case of training specialists within the context of human resources for health and the cost-effectiveness of workforce planning. Particularly as the consensus seems to be that investments in primary care are the most cost-effective interventions.^89^

We recognise limitations in our systematic review. First, the literature on specialists is heavily dominated by clinical sciences, whereas our primary focus was on health systems and medical professions outcomes. This imbalance resulted in a high number of false positives during our searches, increasing the risk of inadvertently excluding relevant studies. Second, our use of an a priori framework guided both our choice of search terms and data extraction process. While such an approach aligns with the best-fit analysis framework methodology, we acknowledge it may have introduced some bias in identifying key findings. As per the evidence included in our review, we recognise that it is likely that the information and evidence we sought on policy, regulation and governance of the professions is unlikely to be in the research literature, but rather in country-specific policy documents which were not included in our review. Some of the older reports on the development of the specialties need to be considered in the context of the time at which studies were done (such as those for China and Malawi), and might no longer be applicable to the current situation of the specialties in those countries.

Our working definition of ‘specialists’ might have underplayed the importance of family medicine, a recognised specialty in many contexts and key for the achievement of UHC goals.^90^ Finally, in addition to employing general terms for medical specialists, we conducted targeted searches for those specialties considered essential by The Lancet NCDI Poverty Commission^23^; we recognise such an approach may have disproportionately inflated the number of hits for certain fields, such as surgery, psychiatry, cardiology and EM, while other less common specialties might have been overlooked.

## Conclusions

Medical specialists are central to referral systems and a major part of the health workforce in HICs. In LMICs, however, specialist services often diverge from local health needs, system capacity and UHC priorities.

Our best-fit framework systematic review found critical shortages of key specialists—including surgeons, anaesthetists and psychiatrists—alongside their varied roles in clinical care, hospital management, training, research and private sector development. Governance of specialist practice is highly uneven, with wide variation in specialties offered, training curricula, accreditation and regulation of private practice. A dynamic global labour market, driven by migration and private sector opportunities, further shapes specialist supply; yet evidence on effective policies to address shortages or improve governance remains scarce.

This review provides a framework for understanding how specialists interact with health systems and influence population health. Greater attention from researchers and policymakers is needed to ensure specialist deployment in LMICs is better aligned with UHC goals.

## Supplementary material

10.1136/bmjgh-2025-018905 online supplemental file 1

10.1136/bmjgh-2025-018905 online supplemental file 2

10.1136/bmjgh-2025-018905 online supplemental file 3

10.1136/bmjgh-2025-018905 online supplemental file 4

10.1136/bmjgh-2025-018905 online supplemental file 5

10.1136/bmjgh-2025-018905 online supplemental file 6

10.1136/bmjgh-2025-018905 online supplemental file 7

## Data Availability

Data are available on reasonable request.
