# Supplementary material for: Medical specialists in LMICs: a systematic review and best-fit framework synthesis of the evidence on their roles and contribution to health systems
Source: BMJ Glob Health. 2026 Jan 9;11(1):e018905. doi: 10.1136/bmjgh-2025-018905 (PMC12815179; doi:10.1136/bmjgh-2025-018905)
Supplement: online supplemental file 1 [file bmjgh-11-1-s001.docx]

**Appendix 1**

**Table S1: List of experts that provided feedback on the a-priori framework**

| **Name** | **Position** | **Organisation** | **Feedback** |
| --- | --- | --- | --- |
| Alex Jones Flores Cassenote | Member of the specialists panel in São Paulo (Brazil) | FMUSP | Contribution to the development of the framework and its fit to Brazil’s market for specialist medical services (see Methods) |
| Alisson Sampaio Lisboa | Member of the specialists panel in São Paulo (Brazil) | SGTES/Ministério da Saúde | Contribution to the development of the framework and its fit to Brazil’s market for specialist medical services (see Methods) |
| Ana Olga Mocumbi | Director of research and senior EM specialist | National Institute of Health (Mozambique) | Good example of specialists piloting new schemes / projects of care in remote settings.  Interesting point about ‘transferring skills to new specialists and generalists’ – is this different from ‘training’ and ‘mentoring’? |
| Cristiane de Jesus Almeida | Member of the specialists panel in São Paulo (Brazil) | FMUSP | Contribution to the development of the framework and its fit to Brazil’s market for specialist medical services (see Methods) |
| Gerson Alves Pereira Junior | Member of the specialists panel in São Paulo (Brazil) | CBC | Contribution to the development of the framework and its fit to Brazil’s market for specialist medical services (see Methods) |
| Giuliano Russo | Member of the specialists panel in São Paulo (Brazil) | QMUL | Contribution to the development of the framework and its fit to Brazil’s market for specialist medical services (see Methods) |
| Ivan Wilson Hossni Dias | Member of the specialists panel in São Paulo (Brazil) | FMUSP | Contribution to the development of the framework and its fit to Brazil’s market for specialist medical services (see Methods) |
| Jakub Gajewski | Programme Director (Research) at RCSI Institute of Global Surgery  Extraordinary Senior Lecturer at the Centre for Global Surgery, University of Stellenbosch, South Africa | Royal College of Surgery, Ireland; University of Stellenbosch, South Africa | Need to clarify the definition of medical specialists. Comments on some of the functions; it is not ‘making referrals’, but rather ‘receiving referred patients from primary care staff. Other functions that could be mentioned, such as mentorship, supervision, and quality assurance. Outreach specialist services, such as ‘eye-care brigades’ in Zambia and Nigeria. |
| Juliana S. Oliveira | Member of the specialists panel in São Paulo (Brazil) | SGTES/Ministério da Saúde | Contribution to the development of the framework and its fit to Brazil’s market for specialist medical services (see Methods) |
| Juliana Sousa | Member of the specialists panel in São Paulo (Brazil) | FMUSP | Contribution to the development of the framework and its fit to Brazil’s market for specialist medical services (see Methods) |
| Karima Khalid MD, MMed | Anaesthesia & critical care physician \| Lecturer | Muhimbili University of Health & Allied Sciences \| Tanzania | Interesting point about ‘critical life-saving services’ to be provided to patients in hospital settings. a set of such services can be identified by specialists, and then carried out by hospital staff.  In order to start a private clinic in Tanzania, you need to be a specialist – or employ a specialist. |
| Laerge Cerqueira | Member of the specialists panel in São Paulo (Brazil) | DEGES/SGTES//Ministério da Saúde | Contribution to the development of the framework and its fit to Brazil’s market for specialist medical services (see Methods) |
| Luís Varandas | Director of Paediatric Services and lecturer | Hospital Dona Estefânia e Maternidade Alfredo da Costa; senior lecturer at NOVA University of Lisbon (Portugal) | Good choice of drivers; outcomes of what represents health system strengthening not well-defined. The governance features of specialties will depend on the country and health systems context, so unlikely to be similar. However, there will be patterns for the ‘basic specialties’, but then smaller specialties will differ. |
| Luiz Alonso David | Member of the specialists panel in São Paulo (Brazil) | SES/SP | Contribution to the development of the framework and its fit to Brazil’s market for specialist medical services (see Methods) |
| Mário Scheffer | Member of the specialists panel in São Paulo (Brazil) | FMUSP | Contribution to the development of the framework and its fit to Brazil’s market for specialist medical services (see Methods) |
| Nivaldo Alonso | Member of the specialists panel in São Paulo (Brazil) | FMUSP | Contribution to the development of the framework and its fit to Brazil’s market for specialist medical services (see Methods) |
| Paola Soledad Mosquera | Member of the specialists panel in São Paulo (Brazil) | FMUSP | Contribution to the development of the framework and its fit to Brazil’s market for specialist medical services (see Methods) |
| Paulo Fernando Constâncio de Souza | Member of the specialists panel in São Paulo (Brazil) | SES/SP/CEREM | Contribution to the development of the framework and its fit to Brazil’s market for specialist medical services (see Methods) |
| Ramiro Colleoni | Member of the specialists panel in São Paulo (Brazil) | CBC | Contribution to the development of the framework and its fit to Brazil’s market for specialist medical services (see Methods) |
| Rodrigo Alves Rodrigues | Member of the specialists panel in São Paulo (Brazil) | SGTES/Ministério da Saúde | Contribution to the development of the framework and its fit to Brazil’s market for specialist medical services (see Methods) |
| Rodrigo Cariri | Member of the specialists panel in São Paulo (Brazil) | SAES/Ministério da Saúde | Contribution to the development of the framework and its fit to Brazil’s market for specialist medical services (see Methods) |

|  |  |
| --- | --- |
| **Acronym** | **Institution** |
| FMUSP | Faculdade de Medicina da Universidade de São Paulo |
| SGTES | Secretaria de Gestão do Trabalho e da Educação na Saúde |
| CBC | Colégio Brasileiro de Cirurgiões |
| DEGES | Departamento de Gestão da Educação na Saúde |
| SES/SP | Secretaria de Estado da Saúde de São Paulo |
| SAES | Secretaria de Atenção Especializada à Saúde |
