# Supplementary material for: Medical specialists in LMICs: a systematic review and best-fit framework synthesis of the evidence on their roles and contribution to health systems
Source: BMJ Glob Health. 2026 Jan 9;11(1):e018905. doi: 10.1136/bmjgh-2025-018905 (PMC12815179; doi:10.1136/bmjgh-2025-018905)
Supplement: online supplemental file 2 [file bmjgh-11-1-s002.docx]

**Appendix 2**

**Table S2: Search terms for first level screening**

**Web of Science**

|  | **Search – screening 2** | **Results** |
| --- | --- | --- |
| 1 | TS=((developing OR "less* developed" OR "under developed" OR underdeveloped OR "middle income" OR "middle-income" OR "low* income" "low-income" OR (underserved OR "under served" OR deprived OR poor* OR emerging) AND (countr* OR nation* OR world OR population* OR econom*))) | 6,808,328 |
| 2 | TS=((Gynaecolog* OR Cardiothoracic surg* OR Surg* OR surg OR "Internal medicine" OR "Critical care" OR "Palliative care" OR Oncolog* OR Cardiolog* OR Ophthalmolog* OR Patholog* OR Radiolog* OR Rehabilitation OR "Orthopedic surg*" OR Otorhinolaryngolog* OR Neurolog* OR Psychiatr* OR Paediatric* OR Obstetric* OR Anaesth*) AND (doctors OR physicians OR medics)) | 145,098 |
| 3 | TS= ((LMIC* OR Africa* OR (africa* OR magreb OR maghrib OR "sub-Saharan" OR Saharan)) OR Asia* (asia* OR subcontinent OR sub-continent) OR (caribbean OR Caribbean* OR west indies OR "Caribbean IslANDs") OR ("pacific region" OR "South America" OR " Central America" OR "Central America" OR "Latin America") OR (Chin* OR Indonesia* OR Mexic* OR Brazil*)) | 2,989,154 |
| 4 | #1 AND #2 AND #3 | [2,213](https://www-webofscience-com.ez67.periodicos.capes.gov.br/wos/woscc/summary/589d81e0-e46c-4ea3-b287-b3fbc4c83258-01074eaa97/relevance/1) |
| 5 | #4 AND TS=special* | 452 |
| 6 | #4 AND TS=special* AND TS=("health system*" OR governance) | 46 |

**PubMed/Medline**

|  | **Search – screening 2** | **Results** |
| --- | --- | --- |
| **1** | ("medical special*" OR Gynaecolog* OR gynaecolog* OR Cardiothoracic surg* OR Surg* OR surg* OR "Internal medicine" OR "critical care"OR "palliative care" OR Oncolog* OR Cardiolog* OR Ophthalmolog* OR Patholog* OR Radiolog* OR Rehabilitation OR "Orthopedic surg*" OR Otorhinolaryngolog* OR Neurolog* OR Psychiatr* OR Paediatric* OR Obstetric* OR Anaesth* OR anaesth*) AND (doctors OR physicians OR medics) Filters: English, Italian, Portuguese, from 1999 - 2025 | [4,106,930](https://pubmed-ncbi-nlm-nih-gov.ez67.periodicos.capes.gov.br/?term=%28%22medical+special%2A%22+OR+Gynaecolog%2A+OR+gynaecolog%2A+OR+Cardiothoracic+surg%2A+OR+Surg%2A+OR+surg%2A+OR+%22Internal+medicine%22+OR+%22critical+care%22OR+%22palliative+care%22+OR+Oncolog%2A+OR+Cardiolog%2A+OR+Ophthalmolog%2A+OR+Patholog%2A+OR+Radiolog%2A+OR+Rehabilitation+OR+%22Orthopedic+surg%2A%22+OR+Otorhinolaryngolog%2A+OR+Neurolog%2A+OR+Psychiatr%2A+OR+Paediatric%2A+OR+Obstetric%2A+OR+Anaesth%2A+OR+anaesth%2A%29+AND+%28doctors+OR+physicians+OR+medics%29&filter=lang.english&filter=lang.italian&filter=lang.portuguese&filter=years.1999-2025&sort=relevance) |
| **2** | (developing OR "less* developed" OR "under developed" OR underdeveloped OR "middle income" OR "middle-income" OR "low income" "low-income"OR (underserved OR "under served" OR deprived OR poor* OR emerging)) AND (countr* OR nation* OR world OR population* OR econom*) Filters: English, Italian, Portuguese, from 1999 - 2025 | 821,522 |
| **3** | #1 AND #2 | 210,86 |
| **4** | Africa* OR (africa* OR magreb OR maghrib OR "sub-Saharan" OR Saharan) OR Asia* OR (asia* OR subcontinent OR sub-continent) OR (caribbean OR Caribbean* OR west indies OR "Caribbean IslANDs") OR ("pacific region" OR "South America" OR "South AND Central America" OR "Central America") OR (Chin* OR Indonesia* OR Mexic* OR Brazil*) Filters: English, Italian, Portuguese, from 1999 - 2025 | [4,337,078](https://pubmed-ncbi-nlm-nih-gov.ez67.periodicos.capes.gov.br/?term=Africa%2A+OR+%28africa%2A+OR+magreb+OR+maghrib+OR+%22sub-Saharan%22+OR+Saharan%29+OR+Asia%2A+OR+%28asia%2A+OR+subcontinent+OR+sub-continent%29+OR+%28caribbean+OR+Caribbean%2A+OR+west+indies+OR+%22Caribbean+Islands%22%29+OR+%28%22pacific+region%22+OR+%22South+America%22+OR+%22South+and+Central+America%22+OR+%22Central+America%22%29+OR+%28Chin%2A+OR+Indonesia%2A+OR+Mexic%2A+OR+Brazil%2A%29&filter=lang.english&filter=lang.italian&filter=lang.portuguese&filter=years.1999-2025&sort=relevance) |
| **5** | #1 AND #2 AND #4 | 55,712 |
| **6** | special* AND (#1 AND #2 AND #4) | [4,347](https://pubmed-ncbi-nlm-nih-gov.ez67.periodicos.capes.gov.br/?term=special%2A+AND+%2315&filter=lang.english&filter=lang.italian&filter=lang.portuguese&sort=relevance) |
| **7** | (health system* OR governace) AND ((special* AND (#1 AND #2 AND #4)) | [574](https://pubmed-ncbi-nlm-nih-gov.ez67.periodicos.capes.gov.br/?term=%28%22health+system%2A%22+OR+governance%29+AND+%2317&sort=&filter=lang.english&filter=lang.italian&filter=lang.portuguese) |

**Scopus**

|  | **Final search** | **Results** |
| --- | --- | --- |
| 1 | ("medical special*" OR Gynaecolog* OR gynaecolog* OR Cardiothoracic surg* OR Surg* OR surg* OR "Internal medicine" OR "critical care"OR "palliative care" OR Oncolog* OR Cardiolog* OR Ophthalmolog* OR Patholog* OR Radiolog* OR Rehabilitation OR "Orthopedic surg*" OR Otorhinolaryngolog* OR Neurolog* OR Psychiatr* OR Paediatric* OR Obstetric* OR Anaesth* OR anaesth*) AND (doctors OR physicians OR medics) AND PUBYEAR > 1998 AND PUBYEAR < 2025 AND ( LIMIT-TO ( LANGUAGE,"English" ) OR LIMIT-TO ( LANGUAGE,"Portuguese" ) OR LIMIT-TO ( LANGUAGE,"Italian" ) ) | 145,998 |
| 2 | (developing OR "less* developed" OR "under developed" OR underdeveloped OR "middle income" OR "middle-income" OR "low income" "low-income"OR (underserved OR "under served" OR deprived OR poor* OR emerging)) AND (countr* OR nation* OR world OR population* OR econom*) AND ( LIMIT-TO ( LANGUAGE,"English" ) OR LIMIT-TO ( LANGUAGE,"Portuguese" ) OR LIMIT-TO ( LANGUAGE,"Italian" ) ) | 1,635,581 |
| 3 | Africa* OR (africa* OR magreb OR maghrib OR "sub-Saharan" OR Saharan) OR Asia* OR (asia* OR subcontinent OR sub-continent) OR (caribbean OR Caribbean* OR west indies OR "Caribbean IslANDs") OR ("pacific region" OR "South America" OR "South AND Central America" OR "Central America") OR (Chin* OR Indonesia* OR Mexic* OR Brazil*) AND PUBYEAR > 1998 AND PUBYEAR < 2025 AND ( LIMIT-TO ( LANGUAGE,"English" ) OR LIMIT-TO ( LANGUAGE,"Portuguese" ) OR LIMIT-TO ( LANGUAGE,"Italian" ) ) | 24,903,517 |
| 4 | #1 AND #2 AND #3 | 10,954 |
| 5 | special* AND (#1 AND #2 AND #3) | 4,826 |
| 6 | ("health system*" OR governance) AND (special* AND (#1 AND #2 AND #3)) | 1,599 |

**Final: 2722 (with duplicates) / after removing duplicates: 2206 (Zotero - "Merged_2nd_search_round" file)**

- EconLit (Giuliano): 17

- Web_of_Science: 452

- Schoolar (Renata): 11

- Specific journals (Giuliano): 49

- Scopus (Tamara): 20

- Scopus (with ‘health system’; Renata): 1599

- PubMed (Renata): 574
