# Supplementary material for: Medical specialists in LMICs: a systematic review and best-fit framework synthesis of the evidence on their roles and contribution to health systems
Source: BMJ Glob Health. 2026 Jan 9;11(1):e018905. doi: 10.1136/bmjgh-2025-018905 (PMC12815179; doi:10.1136/bmjgh-2025-018905)
Supplement: online supplemental file 3 [file bmjgh-11-1-s003.docx]

**Appendix 3**

**Table S3: Inclusion and exclusion criteria**

| **Inclusion criterion** | **Example of paper to include** | **Example of what to exclude** |
| --- | --- | --- |
| Academic, published papers | Papers published in academic journals of medical sciences, health systems, human resources for health, health services | Newspapers articles, non-peer-reviewed pieces |
| Papers presenting qualitative, quantitative, or mixed-methods evidence | Original research papers, or papers presenting original analysis of secondary data | Opinion pieces, commentaries |
| Papers available in English, Spanish, French, Portuguese and Italian | Academic papers with abstracts in the four languages | Papers without titles or abstract in any of the four languages (eg, Arabic, Chinese) |
| Article/paper originating from a Low-income country or a middle-income country | Use OECD Development Assistance Committee’s list of Official Development Assistance recipients[20] to identify low- and middle-income countries, which as of 2024 includes 47 least-developed and low-income countries, 35 lower-middle income, and 59 upper-middle income countries and territories | Papers on high-income countries |
| Focus on medical specialists | Papers focussing on surgeons, psychiatrists, gynaecologists, paediatricians, etc | Focus on generalist doctors, physicians, general medicine, or other health workers |
| Focus on specialties | One of the key specialties highlighted by the WHO | Papers focusing on doctors in general, or on the medical science without specifying the specific medical field |
| Contribution of specialists on population health and health systems strengthening | Papers assessing specialists’ direct contribution to treatments, provision of medical services | Papers not mentioning specific outcomes of specialists’ services |
| Focus on specialists’ specific ‘functions’ performed within a health system (see the list of functions in Figure 1) | Papers reporting on functions like provision of specialist services, teaching, mentoring, outreach services etc | Papers not mentioning specific functions performed by specialists |
| Papers describing organisation and governance of medical specialties (see the full list of specialists recommended by the WHO and Lancet Commission in LMICs) | Papers providing data on proportion of specialists per population, organisation of different specialties in specific countries, organisation of specialty colleges | Papers focussing on governance issues in higher-income countries |
| Focus on determinants of doctors’ decisions to engage with specific specialties | Papers focussing on motifs and motivations to study specific areas of medicine, such as income-related decisions, availability of courses or training places | Papers discussing doctors’ motivations to practice medicine in general without any specific reference to specialties |
| Focus on labour market conditions influencing working opportunities within specific areas of medicine | Scarcity of specialists in specific countries or regions, demand for specialised services, specialty residence places | Labour market papers without specific reference to doctors, specialties, or health services |
| Focus on specialists’ socio-economic characteristics, and their association with ‘functions’ | Focus on family economic background of specialists, class, or secondary education | Papers without specific focus on medical specialties |
| Focus on specialists’ engagement with private sector | Specialists’ employment in private hospitals or clinics, dual practice | Papers without specific focus on medical specialties, specialists, or doctors in general |
|  |  |  |
