# Supplementary material for: Medical specialists in LMICs: a systematic review and best-fit framework synthesis of the evidence on their roles and contribution to health systems
Source: BMJ Glob Health. 2026 Jan 9;11(1):e018905. doi: 10.1136/bmjgh-2025-018905 (PMC12815179; doi:10.1136/bmjgh-2025-018905)
Supplement: online supplemental file 4 [file bmjgh-11-1-s004.pdf]

Appendix 4

Table S4: List of papers excluded, with reason

Key	Year	Author	Title	Publication Title	Reason for exclusion
UHS4AU7X	2016	Aaron, Eliana Marc	Integration of advanced practice provid	Isr. J. Health Policy	Not on LMICs
HYULLJ9B	2005	Aaserud, Morten; L	Translating research into policy and pra	BMC Health Serv. R	No info on specialists' contribution to health systems/population health, or their governance
2UVJDVPQ	2014	Abas, Melanie A; NI	Building mental health workforce capa	Int. Rev. Psychiatry	No info on specialists' contribution to health systems/population health, or their governance
QMA5GIET	2021	Abbas, Karrar Moha	Retrospective Assessment of the Clinic	J. Commun. Dis.	No specific focus on specialists or the professions
B82ACBFH	2018	Abbas, Mohamed; F	Conflicts of interest in infection preven	Intensive Care Med	No specific focus on specialists or the professions
L8LMAF9V	2016	Abdel-Aleem, Hany	Mobile clinics for women's and children	Cochrane Databas	No specific focus on specialists or the professions
A7SLBC43	2024	AbdEl-Aty, Mahmou	Exploring factors for antibiotic over-pre	Antimicrob. Resist.	No specific focus on specialists or the professions
D24ZGZ79	2024	Abdel-Wahab, May	Addressing challenges in low-income ar	Lancet. Oncol.	No specific focus on specialists or the professions
SVGTK7JN	2024	Abdelgadir, Hiba Sa	Implementation of the clinical practice	BMC Prim. Care	No specific focus on specialists or the professions
G8K2WVBL	2019	Abdelrahman, Moh	Temporary Migration for Training: A Spe	Ann. Plast. Surg.	No info on specialists' contribution to health systems/population health, or their governance
LGNYBFZ5	2024	Abdul Rahim, Koma	No healthcare coverage, big problem: la	Trauma Surg. acute	No specific focus on specialists or the professions
HTQWKS2S	2018	Abelson, Jonathan	Racial and ethnic disparities in promot	Am. J. Surg.	Not on LMICs
9YTDUAI5	2014	Abera, Mubarek; Te	Perceived challenges and opportunities	BMC Health Serv. R	No specific focus on specialists or the professions
WT3GE2HY	2023	Åberg, Daniel; Gad	Serum IGFBP-1 Concentration as a Pre	Int. J. Mol. Sci.	No specific focus on specialists or the professions
YN7FQDKH	2024	Abiona, Olukorede;	Physician responses to insurance bene	Heal. Econ. (United	Not on LMICs
TDEJ7EWJ	2024	Abiri, Onome Thom	Assessment of self-medication practice	J. Pharm. Policy Pra	No specific focus on specialists or the professions
YVESUNI7	2022	Aboelkhir, Hesham	A Bibliometric Analysis and Visualizatio	Int. J. Environ. Res.	No specific focus on specialists or the professions
TK53DZ4L	2023	Abraha, Hiluf Ebuy;	Impact of a double catastrophe, war an	Confl. Health	No specific focus on specialists or the professions
TD4FJ77G	2022	Abrokwa, Seth Kofi;	Task shifting for point of care ultrasound	eClinicalMedicine	No specific focus on specialists or the professions
5FLNJIGC	2018	Abubakar, Ibrahim;	The UCL–Lancet Commission on Migrat	Lancet	No specific focus on specialists or the professions
8MRYR49F	2022	Abubakar, Ibrahim;	The Lancet Nigeria Commission: invest	Lancet	No specific focus on specialists or the professions
XNDSSU56	2016	Abughosh, Susan M	A pharmacist telephone intervention to	J. Manag. Care Spec	No specific focus on specialists or the professions
W4W44JZ7	2021	Abukmail, Eman; Al	Postgraduate training abroad and migra	Lancet	
M7WB5TUR	2011	Ache, Kevin A; Shar	A preliminary study comparing attitudes	J. Palliat. Med.	No specific focus on specialists or the professions
AVDUPUAT	2019	Adamu, A N; Okusa	Maternal near-miss and death among w	BJOG An Int. J. Obst	No specific focus on specialists or the professions
6GJPHKP3	2024	Adde, Håvard A; Og	The economic burden associated with u	BMJ Open	No specific focus on specialists or the professions
XW9WCEQ3	2019	Adedinsewo, Demi	Arrhythmia care in Africa	J. Interv. Card. Elec	No specific focus on specialists or the professions
KLBFXRMB	2022	Adejumo, P O; Oluw	Oncology Training Needs Assessment a	JCO Glob. Oncol.	No specific focus on specialists or the professions
TDVV29TY	2017	Adeola, Henry A; Sc	Omics-based molecular techniques in c	Cancer Cell Int.	No specific focus on specialists or the professions
8LIAGKBZ	2021	Adeyemi, Olukemi;	Integration of non-communicable disea	BMJ Glob. Heal.	No specific focus on specialists or the professions
2Y2TY425	2008	Adler, Nancy E; Pag	Cancer care for the whole patient: Meeting psychosocial he		No specific focus on specialists or the professions
379B7ND4	2022	Adrissi, Jennifer; Fl	Moving the Dial Toward Equity in Parkin	Curr. Neurol. Neuro	No specific focus on specialists or the professions
GIU4U76Z	2022	Adu-Bonsaffoh, Kw	Health professionals' perspectives on c	Front. Glob. Women	No specific focus on specialists or the professions
6PBAM33C	2015	Adusi-Poku, Yaw; A	Quality of care: A review of maternal de	Afr. J. Reprod. Healt	No specific focus on specialists or the professions
7F7J5BFL	2023	Aechtner, Thomas	Antivaccination and Vaccine Hesitancy: A Professional Gui		No specific focus on specialists or the professions
VUCKH8CP	2020	Afiat, Thanh-Phuon	Diffuse Intrasinusoidal Hepatic Metast	Am. J. Case Rep.	No specific focus on specialists or the professions
C5YZIQ4I	2011	Afzal, Muhammad;	Global health workforce alliance: Incre	Rev. Peru. Med. Exp	No specific focus on specialists or the professions
TBBNSEV2	2018	Aguado, J M; Silva,	Management of multidrug resistant Gra	Transplant. Rev.	No specific focus on specialists or the professions
6XMAYS4P	2017	Agyepong, Irene Ak	Spanning maternal, newborn and child	Heal. Res. Policy Sy	No specific focus on specialists or the professions
HHMX37F9	2024	Ahmed, Fasih Ali; Z	Burns in South Asia: Outcomes from So	Burns	No specific focus on specialists or the professions
J45T8E7L	2023	Ahmed, Frass; Jone	Orthopaedic Surgery Residency Advice	JB JS open access	No specific focus on specialists or the professions
EFUDV7ZJ	2019	Ahmed, Shakil; Ada	Impact of traffic variability on geograph	PLoS One	No specific focus on specialists or the professions
75FXSBLA	2020	Ahmed, Syed A K Sh	Impact of the societal response to covi	BMJ Glob. Heal.	No specific focus on specialists or the professions
LYXE7X8E	2024	Ahmed, Syed Masu	Delivering non-communicable disease	Lancet Glob. Heal.	No specific focus on specialists or the professions
TJXCZXC4	2013	Ahuja, Rajeev B; Go	Cost of providing inpatient burn care in	BURNS	No specific focus on specialists or the professions
GINDFX3A	2020	Akbar, Shahid	Handbook of 200 medicinal plants: A comprehensive review		No specific focus on specialists or the professions
EGWMVY2E	2016	Akeju, David O; Vid	Community perceptions of pre-eclamps	Reprod. Health	No specific focus on specialists or the professions
R56V9QV8	2020	Åkesson, Kristina E	Advances in delivery of health care for N	Best Pract. Res. Cli	No specific focus on specialists or the professions
5U8R6XTD	2021	Akhaddar, Ali	Raphael Acquaviva: The Forgotten Pion	World Neurosurg.	Other
D589PYBT	2016	Akhlaq, Ather; McK	Barriers and facilitators to health inforr	Health Policy Plan.	No specific focus on specialists or the professions
BWCHN7EE	2021	Akhter, Mohammed	Global health: Where do cardiologists f	Heart	
9V99EZMD	2022	Akinwumi, Adebow	Prevalence and pattern of migration int	Hum. Resour. Healt	No info on specialists' contribution to health systems/population health, or their governance
NY99EB5J	2008	Akl, E A; Maroun, N;	Post-graduation migration intentions of	BMC Public Health	
JVIBAYZ2	2022	Al Imam, Mahmudu	Situation analysis of rehabilitation serv	Disabil. Rehabil.	No specific focus on specialists or the professions
PRHHRIUB	2017	Al-Areefi, Mahmou	Perceptions of Yemeni physicians abou	J. Pharm. Heal. Serv	No info on specialists' contribution to health systems/population health, or their governance
G58SAYNR	2021	Al-Busaidi, Ahmed	Development and validation of an instru	BMC Med. Ethics	No info on specialists' contribution to health systems/population health, or their governance
WM347MLT	2024	Al-Salman, J; Amir,	The Management of Patients at High Ris	CUREUS J. Med. Sci	No specific focus on specialists or the professions
7CJGSSZW	2018	Al-Yateem, N; Al-Ta	Research priorities for specialized nurs	Int. Nurs. Rev.	No specific focus on specialists or the professions
62Q2BPSC	2023	Alanazi, Abdullah M	The Intersection of Health Rehabilitatio	Healthc.	No specific focus on specialists or the professions
3FH7FNVK	2023	Alaqeel, Saud Abdu	Investigating factors that influence resi	BMC Med. Educ.	
V97C36RD	2024	Alavian, Sara; Birkl	A needs assessment for formal emergen	AFRICAN J. Emerg. I	No info on specialists' contribution to health systems/population health, or their governance
99A7GDWU	2023	Alayande, Barnaba	Determining Critical Topics for Undergr	Cureus	No info on specialists' contribution to health systems/population health, or their governance
X9MBIH5W	2022	Alayande, Barnaba	Disparities in Access to Trauma Care in	Curr. Trauma Repor	No info on specialists' contribution to health systems/population health, or their governance
6HGNUEXW	2023	Albarqouni, Loai; Pa	Overuse of medications in low-and mid	Bull. World Health (	No specific focus on specialists or the professions
AI5ER97D	2021	Aldana Lopez, Jesu	Digital survey of mental health, associa	SALUD Ment.	No info on specialists' contribution to health systems/population health, or their governance
2SS7XE3K	2021	Alhazzani, Waleed;	Surviving Sepsis Campaign Guidelines c	Crit. Care Med.	No specific focus on specialists or the professions
S6M8QLC7	2022	Ali, Mostafa A; Huss	The Impact of Intellectual Capital on Dy	J. Risk Financ. Mana	No specific focus on specialists or the professions
85V9TQRX	2021	Ali, Qurban; Ahmar	Research advances and applications of	Environ. Sci. Pollut.	No specific focus on specialists or the professions
L57JYM6V	2020	Ali, Sumera Aziz; Kh	Prevalence and determinants of anemia	J. Coll. Physicians S	No specific focus on specialists or the professions
IUWXKF2G	2014	Aliu, Oluseyi; Auger	The effect of pre-Affordable care act (AC	Med. Care	Not on LMICs
KF5Q5MXK	2019	Aliyu, M H; Abdulla	Bridging the childhood epilepsy treatme	Contemp. Clin. TRIA	No specific focus on specialists or the professions
HQ6G7PPT	2022	Aliyu, Muktar H; Sa	Building Research Capacity in HIV and	J. Contin. Educ. Hea	No info on specialists' contribution to health systems/population health, or their governance
W7IYPCP8	2020	Aljurf, M; Weisdorf,	Worldwide Network for Blood and Marr	Hematol. Oncol. Ste	No specific focus on specialists or the professions
LVIZR56G	2017	Allanson, Emma R;	Implementation of effective practices i	BMJ Glob. Heal.	No specific focus on specialists or the professions
YLESTBPJ	2010	Allegranzi, Benede	Successful implementation of the Worl	Infect. Control Hos	No specific focus on specialists or the professions
WRNYVWA3	2024	Allehebi, Ahmed; Al	Recommended approaches for screeni	J. Thorac. Dis.	No specific focus on specialists or the professions
Z95SFVIK	2021	Allen, John; Zareen	Multi-Organ Dysfunction in Cerebral Pa	Front. Pediatr.	No specific focus on specialists or the professions
UVJJISIJ	2023	Allen, LaRue; Hutto	Closing the opportunity gap for young children		No specific focus on specialists or the professions
ETFVCGUW	2024	Allende, Silvia; Turc	Early Incorporation to Palliative Care (E	Oncologist	No specific focus on specialists or the professions
429CQG8L	2024	Almalki, Nabat; Boy	What helps or hinders effective end-of-	BMC Palliat. Care	No specific focus on specialists or the professions
NXS7CGX3	2021	Almazrou, Saja H; A	Assessing the quality of clinical practic	J. Multidiscip. Healt	No specific focus on specialists or the professions
BP75WWPJ	2020	Alonge, Olakunle; C	Identifying pathways for large-scale imp	Health Policy Plan.	No specific focus on specialists or the professions
Z4GHRPXQ	2015	Alston, Margaret	Women and climate change in Bangladesh		No specific focus on specialists or the professions
JJE4GSXH	2022	Amarasinghe, Gaya	Help-seeking intention for depression a	Rural Remote Healt	No specific focus on specialists or the professions
IH8W5V82	2017	Amato, Laura; Fusc	Volume and health outcomes: Evidence	Epidemiol. Prev.	Not on LMICs
E92BFQGL	2013	Ambresin, Anne-Em	Assessment of youth-friendly health car	J. Adolesc. Heal.	No specific focus on specialists or the professions
VL9W6B3J	2022	Ameh, Charles A; M	A synthesis of clinical and health syster	Int. J. Gynaecol. Obstet. Off. organ Int. Fed. Gynaecol. Obstet.	
7LPQGP9G	2006	Ameh, Emmanuel A	Pediatric surgery in Nigeria.	J. Pediatr. Surg.	
P4ZUWJAZ	2018	Amendola, Laura M	The Clinical Sequencing Evidence-Gen	Am. J. Hum. Genet.	No specific focus on specialists or the professions
3M374SS4	2015	Amgad, Mohamed;	Medical student research: An integrate	PLoS One	
R7FAPFU3	2018	Ammer, Kurt	Medical Thermology 2017 - A computer-	Thermol. Int.	No specific focus on specialists or the professions
ZN5J4AGZ	2014	Amsterdam, Ezra A	2014 AHA/ACC guideline for the manag	Circulation	Not on LMICs
I7I5WKTW	2021	Anand, S Vivek; Shu	One year on: An overview of Singapore's	Int. J. Environ. Res.	No specific focus on specialists or the professions
DG3LIG8F	2022	Anastassakis, Kons	Androgenetic Alopecia From A to Z: Vol. 2 Drugs, Herbs, Nut		No specific focus on specialists or the professions
TF4N8W38	2019	Anazodo, Antoinett	How can we improve oncofertility care	Hum. Reprod. Upda	No info on specialists' contribution to health systems/population health, or their governance
4U7TB9I2	2012	Andermann, Anne	Evidence for health: From patient choice to global policy		No specific focus on specialists or the professions
NKRGIRER	2013	Andermann, Anne;	Case studies of patient safety research	Jt. Comm. J. Qual. P	No specific focus on specialists or the professions
INLKF6UY	2019	Andersen, Bjørg Ma	Prevention and control of infections in hospitals: Practice a		No specific focus on specialists or the professions

Appendix 4

Table S4: List of papers excluded, with reason

3AWEZ5YZ	2019	Anderson, Brian J; L	Pharmacokinetics and Pharmacology of Drugs Used in Child		Not on LMICs
LTV4XRQ7	2019	Anderson, Harry; B	What's new in academic medicine? Thi	Int. J. Acad. Med.	Not on LMICs
7WG4ZMX8	2019	Ang, Ian Yi Han; Ng,	Right-Site Care Programme with a com	BMJ Open	Not on LMICs
3Z6DP279	2017	Angel, Ronald J; Ang	Family, intergenerational solidarity, and post-traditional so		No specific focus on specialists or the professions
V27EV7JY	2012	Angela, Tulaar B M	The Challenges Of Physical Medicine Ar	3RD ASIA-OCEANIA	Other
S6NYZ7JQ	2020	Angelini, Gianni D;	Developing a cardiac surgery unit in the	J. Card. Surg.	No specific focus on specialists or the professions
9P4KW4PF	2020	Anjana, Ranjit Moh	Contrasting Associations Between Dial	Diabetes Care	No specific focus on specialists or the professions
SDA3QZIS	2016	Anteyi, Kate; Shi, Le	The impact of primary care and the importance of indicator		No specific focus on specialists or the professions
TAIJZU9F	2015	Anunobi, Echezona	Comparison of Advance Medical Direct	J. AGING & Soc. POl	No specific focus on specialists or the professions
UAQQG7JU	2011	Anyanwu, Ebere Cy	Advances in environmental health effects of toxigenic mold		No specific focus on specialists or the professions
B3RBCTDI	2018	Apisarnthanarak, A	Infection Prevention in Resource-Limited Settings		No specific focus on specialists or the professions
3EFK4Q4J	2021	Aponte-Rueda, Ma	Strengthening breast surgery workforce	Ecancermedicalsci	Other
RFG8PU27	2023	Appiah, Evans Osei	Exploring the challenges and roles of nu	BMC Palliat. Care	No specific focus on specialists or the professions
8BFAIPEC	2024	Arafat, S M Yasir; Ka	Prison mental health in South-East Asia	Brain Behav.	No specific focus on specialists or the professions
YLLBQLCL	2022	Aragonès, Enric; de	Psychological impact of the COVID-19	Br. J. Gen. Pract.	No specific focus on specialists or the professions
IQAD8W6E	2021	Araiza-Garaygordo	Impact of the COVID-19 pandemic on h	QJM	No specific focus on specialists or the professions
EW4PC7NK	2009	Araujo, Aderson; Di	Management of transfusional iron overl	HEMATOLOGY	No specific focus on specialists or the professions
NAS4Y7F3	2016	Are, C; Caniglia, A;	Variations in Training of Surgical Oncolo	Ann. Surg. Oncol.	No info on specialists' contribution to health systems/population health, or their governance
6JNFBJ3C	2014	Argent, Andrew C; K	Pediatric critical care: A global view		
4JAUY2KR	2023	Aristizabal, Paula; I	Childhood Leukemia Survival in the US-	JCO Glob. Oncol.	No specific focus on specialists or the professions
3D3TGN5G	2007	Arora, Sanjeev; Ge	Academic health center management c	Acad. Med.	No specific focus on specialists or the professions
NU7B7RPW	2024	Arsenault, Catheri	Antenatal care quality and detection of	PLoS Med.	No specific focus on specialists or the professions
P5PDGVEZ	2023	Arusi, Temesgen Ta	Predictors of Uterine Rupture After One	Int. J. Womens. Hea	No specific focus on specialists or the professions
GCWEMDWK	2024	Asa, Gregorius Aba	Medical tourism among Indonesians: a	BMC Health Serv. R	No specific focus on specialists or the professions
XTL83EEH	2022	Asamani, James Av	Exploring the availability of specialist h	BMJ Glob. Heal.	
IL3LRQR4	2023	Asante, Dennis; Mc	Understanding Unmet Care Needs of Ru	Int. J. Environ. Res.	No specific focus on specialists or the professions
XZLCM68B	2017	Ashengo, Tigistu; Sk	Bridging the human resource gap in sur	Hum. Resour. Health	
LSBX89MD	2021	Asher, L; Birhane, F	"Like a doctor, like a brother": Achieving	PLoS One	No info on specialists' contribution to health systems/population health, or their governance
33JRS3N8	2022	Asher, Laura; Birha	Community-based rehabilitation interv	Lancet. Glob. Heal.	No specific focus on specialists or the professions
VTPV5TBR	2024	Ashley, Louise; McI	When the Penny Drops: Understanding	Soc. Sci. & Med.	
GWKY7L87	2015	Ashmore, J; Gilson,	Conceptualizing the impacts of dual pra	Hum. Resour. Health	
VXP8QMVA	2013	Ashmore, John	Going private': A qualitative compariso	Hum. Resour. Health	
TC26C7XQ	2022	Ashraf, Muhammad	Access to safe, timely and affordable su	East. Mediterr. Hea	No specific focus on specialists or the professions
6C6VXN2B	2021	Asiimwe, Savina; A	Medicinal Plants in Uganda as Potential	Therapeutics again	No specific focus on specialists or the professions
AF7FFU3H	2023	Aslam, Muhammad	Artificial intelligence applications using	ChatGPT in educat	No specific focus on specialists or the professions
D5TP2DS7	2018	Aso, Michitake	Rubber and the Making of Vietnam: An Ecological History, 18		No specific focus on specialists or the professions
48W4GZHQ	2020	Asokan, G V; Yusuf,	Levels and determinants of health litera	Oman Med. J.	No specific focus on specialists or the professions
K89AUVL6	2006	Asthana, Sheena; H	What works in tackling health inequalities?: Pathways, polic		No specific focus on specialists or the professions
PI47YDVG	2023	Athikarisamy, Sam	Retinopathy of prematurity in India – wh	Lancet Reg. Heal. -	No info on specialists' contribution to health systems/population health, or their governance
WUJ8FMNM	2010	Atiyeh, Bishara S; G	Provision of essential surgery in remote	Int. J. Surg.	
YIHIN4BD	2012	Atkinson, Arthur J; H	Principles of Clinical Pharmacology		No specific focus on specialists or the professions
AY9XCX2K	2024	Atnafu, Asmamaw;	Health system responsiveness and its a	BMJ Open	No specific focus on specialists or the professions
MXQQGLEJ	2024	Attri, Bhawna; Nag	Prandial Insulins: A Person-Centered C	Curr. Diab. Rep.	No specific focus on specialists or the professions
BBA7VARE	2022	Atuhairwe, Susan;	Exploring health care providers' experie	PLoS One	No specific focus on specialists or the professions
JQFEECKB	2021	Atuhairwe, Susan;	Abortion-related near-miss morbidity a	BMJ Glob. Heal.	No specific focus on specialists or the professions
HB34TYGW	2020	Atun, Rifat; Bhakta	Sustainable care for children with canc	Lancet. Oncol.	No specific focus on specialists or the professions
DP34G6UC	2021	Aula, Oyime Poise;	Schistosomiasis with a focus on Africa	Trop. Med. Infect. D	No specific focus on specialists or the professions
PRYD38YP	2019	Avasthi, Ajit; Basu,	Epidemiology of dependence on illicit s	Asian J. Psychiatr.	No specific focus on specialists or the professions
DWSWXH46	2023	Avlijas, Tanja; Squi	A concept analysis of the patient experi	Patient Exp. J.	No specific focus on specialists or the professions
W84N32YC	2018	Avortri, Gertrude S	Women's perspective of facility-based	African J. Prim. Hea	No specific focus on specialists or the professions
7XSNXGSE	2020	Ayah, Richard; Ong	Responding to maternal, neonatal and	BMJ Innov.	No specific focus on specialists or the professions
3XLKWJCZ	2021	Ayebare, Elizabeth	Health care workers' experiences of ma	Reprod. Health	No specific focus on specialists or the professions
D9GKKXP3	2014	Azeka, Estela; Jate	I guidelines of heart failure (IC) and hea	Arq. Bras. Cardiol.	No specific focus on specialists or the professions
MQAWRAT9	2022	Azharuddin, Nur Sy	Ethical Issues in Tissue Engineering: A S	Malaysian J. Med. H	No info on specialists' contribution to health systems/population health, or their governance
87XHP86R	2017	Baatiema, Leonard	Barriers to evidence-based acute strok	BMJ Open	
9NPRC63X	2017	Baatiema, Leonard	Towards best practice in acute stroke c	BMC Health Serv. R	No info on specialists' contribution to health systems/population health, or their governance
CYP9NF7Q	2023	Babor, Thomas F; C	Alcohol: No ordinary commodity research and public policy		No specific focus on specialists or the professions
9LMRSBXG	2022	Bachler, Mirjam; A	Thromboprophylaxis with argatroban in	Blood Coagul. Fibri	No specific focus on specialists or the professions
ZMYNVI2R	2020	Badejo, Okikiolu; S	Confronting power in low places: histor	BMJ Glob. Heal.	
ZTFLI7V8	2020	Bae, Crystal; Naik,	Assessment of Local Health Worker Atti	J. Epidemiol. Glob. Health	
MSXVPXMH	2022	Bagalkot, Naveen;	Embodied Negotiations, Practices and	Conf. Hum. Factors	No specific focus on specialists or the professions
6WDQCN8H	2007	Baggott, Rob	Understanding health policy		No specific focus on specialists or the professions
CFMKZ958	2004	Baggott, Rob	Health and health care in Britain		Not on LMICs
WA8MU6JL	2024	Bagnasco, Annama	Protective and risk factors of workplace	J. Clin. Nurs.	No specific focus on specialists or the professions
Z3P62NEG	2013	Bahadori, Mohamn	Perceived barriers affecting access to p	Iran. Red Crescent	No info on specialists' contribution to health systems/population health, or their governance
GDE5UIT9	2020	Bahri, Priya	A multilayered research framework 1 for humanities and epi		No specific focus on specialists or the professions
YJABNQYC	2023	Bai, Long; Tao, Gan	Hydrogel Drug Delivery Systems for Bon	Pharmaceutics	No specific focus on specialists or the professions
SSE4TC6C	2022	Baig, Muhammad F	The Role(s) of Psychopharmacology in the Treatment of PTS		Not on LMICs
KTABZC7R	2023	Bakhbakhi, Danya;	Interventions, outcomes and outcome	BJOG An Int. J. Obst	No specific focus on specialists or the professions
NSJWRTED	2021	Bakker, Juul; Van D	Barriers to increase surgical productivi	BMJ Open	No info on specialists' contribution to health systems/population health, or their governance
4D9Y2V6J	2020	Bakshandeh, Arta;	Artificial Intelligence in Subspecialties		No specific focus on specialists or the professions
UJD3DYCI	2016	Bakwatanisa, Bosc	Biomaterials use in Mulago National Re	J. Biomed. Mater. R	No specific focus on specialists or the professions
FV5QBKA8	2005	Baldwin, Laura-Ma	Explaining black-white differences in re	J. Natl. Cancer Inst.	No specific focus on specialists or the professions
8X8CQ23N	2022	Balogun, Joseph Ab	The Nigerian Healthcare System: Pathway to Universal and		No specific focus on specialists or the professions
9S4UMEDX	2023	Balogun, Joseph Ab	Health Research in Nigeria: A Bibliometric Analysis		No specific focus on specialists or the professions
BSYFY7RN	2021	Balogun, Mobolanl	Challenges in access and satisfaction w	PLoS One	No specific focus on specialists or the professions
GWYW5QJK	2022	Balogun, S; Ubom,	Nigerian resident doctors' work schedu	Niger. J. Clin. Pract.	No info on specialists' contribution to health systems/population health, or their governance
S4URCFSB	2020	Bamias, Giorgos; La	The Greek Response to COVID-19: A Tru	Inflamm. Bowel Dis	Not on LMICs
TSQQLCFF	2024	Bammert, Philip; Sc	The role of mesolevel characteristics of	Int. J. Equity Health	No specific focus on specialists or the professions
GED3V3FD	2024	Bandyopadhyay, Sc	A review of twenty-first century develop	Pediatr. Surg. Int.	
SQTAKV5W	2017	Banik, Bijoy Krishna	Improving Maternal Health: A Comparis	J. Health Manag.	No specific focus on specialists or the professions
UV4DXA6Z	2022	Bansal, Shyam; Khe	Preparing for Transplant-Screening and	INDIAN J. Transplan	No specific focus on specialists or the professions
QDW2T2KN	2023	Barabino, Gilda A; I	Advancing antiracism, diversity, equity, and inclusion in STE		No info on specialists' contribution to health systems/population health, or their governance
U5XCFW8F	2022	Barakat, Philipp; St	Oncology specialists' perspective on ca	Lancet Oncol.	No specific focus on specialists or the professions
ZR5RIWIN	2022	Baral, Stefan; Rao,	Competing health risks associated with	PLoS One	No specific focus on specialists or the professions
4NIX6IIA	2021	Barayev, Edward; S	WhatsApp Tele-Medicine – usage patte	Isr. J. Health Policy	No specific focus on specialists or the professions
24I8NDQ3	2015	Barbazza, Erica; La	Health workforce governance: Process	Health Policy (New	No specific focus on specialists or the professions
YDJTE4UM	2016	Barbosa, Flavio; Tr	Meta-generalis: A novel method for stru	Appl. Clin. Inform.	No specific focus on specialists or the professions
HDMJG5M7	2020	Barbosa, William; Z	Improving Access to Care: Telemedicin	Annu. Rev. Public H	No specific focus on specialists or the professions
A7NFXLSM	2016	Bard, Eleanor; Knig	Perinatal health care services for impri	BMC Pregnancy Ch	No specific focus on specialists or the professions
IFLGU92M	2011	Barken, Frederick N	Out of practice: Fighting for primary care medicine in Ameri		Not on LMICs
8ZA7VXUC	2013	Barr, Juliana; Frase	Clinical practice guidelines for the mar	Crit. Care Med.	No specific focus on specialists or the professions
VK4QVSUX	2022	Barrie, Umaru; Will	Characteristics of graduating medical s	Clin. Neurol. Neuro	Not on LMICs
EXG2DBA5	2019	Basavarajaiah, D M	HIV transmission: Statistical modelling		No specific focus on specialists or the professions
7J47PKLR	2022	Bashir, Saima; Nas	Association between efficiency and qua	Int. J. Health Plann.	No specific focus on specialists or the professions
VACE4FTB	2009	Bateman, E; Feldm	Systems for the management of respira	Prim. Care Respir. J	No specific focus on specialists or the professions
2K3Y5C36	2016	Bateman, R M; Sha	36th International Symposium on Intensive Care and Emerg		Not on LMICs
JBHPUWMC	2024	Bathula, Archana;	Blockchain, artificial intelligence, and	Artif. Intell. Rev.	No specific focus on specialists or the professions
M3YKKYSN	2009	Battin, Margaret P;	The Patient as Victim and Vector: Ethics and Infectious Dise		No specific focus on specialists or the professions

Appendix 4

Table S4: List of papers excluded, with reason

QL3W9ZU4	2007	Batty, G David; Alv	Examining life-course influences on chr	Brazilian J. Med. Bio	No specific focus on specialists or the professions
2DBZ29UE	2009	Batty, G David; Vic	Family-based life course studies in low-and middle-income		No specific focus on specialists or the professions
YV2EL5IG	2021	Baumann, A A; Hoo	Exploring contextual factors influencing	BMJ Open	No specific focus on specialists or the professions
NRMBGKDN	2019	Bayat, Mahboubeh	Factors associated with dual practice i	Iran. J. Public Healt	No info on specialists' contribution to health systems/population health, or their governance
JAPZG59U	2018	Bayat, Mahboubeh	Extent and nature of dual practice enga	Hum. Resour. Health	
CBAHIXDW	2019	Bayati, Mohsen; Ra	Income inequality among general pract	BMC Health Serv. R	No specific focus on specialists or the professions
6BXRKW72	2024	Baynam, Gareth; H	Global health for rare diseases through	Lancet. Glob. Heal.	No info on specialists' contribution to health systems/population health, or their governance
XAQ784AJ	2014	Bayrami, Roghieh;	Challenges of providing cervical cancer	Asian Pac. J. Cance	No specific focus on specialists or the professions
RESX68TK	2016	Bazargan, Mohsen;	Inappropriate Medication Use Among U	J. Aging Health	No specific focus on specialists or the professions
5RJUXA3T	2016	Bazemore, Andrew	The paradox of primary care		No specific focus on specialists or the professions
8TI7S39G	2023	Bdaiwi, Yamama; S	Impact of armed conflict on health prof	BMJ Open	No info on specialists' contribution to health systems/population health, or their governance
KKS6CKTC	2018	Becerra-Culqui, Tr	Patterns and correlates of cervical can	Am. J. Obstet. Gyne	No specific focus on specialists or the professions
8KWM75AE	2022	Beddoe, Ann Marie	Community-Institutional Partnerships f	Front. Public Heal.	No info on specialists' contribution to health systems/population health, or their governance
YXCWYQ44	2016	Bedoya-Vaca, Rita	Gender and physician specialization ar	BMC Health Serv. Res.	
35SDQXH6	2024	Beecher, Claire; H	Health Care Worker Education for Palli	Am. J. Hosp. Palliat.	No info on specialists' contribution to health systems/population health, or their governance
5WSW3V83	2019	Beghetti, Maurice;	Selexipag treatment for pulmonary arte	Eur. J. Heart Fail.	No specific focus on specialists or the professions
4G76SRXU	2022	Bégin, Philippe; Ca	Author Correction: Convalescent plasma for hospitalized p		No specific focus on specialists or the professions
E2JCF4NV	2022	Behera, Basanta K	Healthcare Strategies and Planning for Social Inclusion and		No specific focus on specialists or the professions
NABLV27I	2019	Behera, Basanta K	Move towards zero hunger		No specific focus on specialists or the professions
DSK2BGK5	2022	Bell, Cathrine; App	Improving Health Care for Patients with	Int. J. Integr. Care	No specific focus on specialists or the professions
QTVYVWWT	2016	Bell, Kirsten	Health and other unassailable values: Reconfigurations of h		No specific focus on specialists or the professions
982J6GNT	2024	Bello, Aminu K; Okp	An update on the global disparities in ki	Lancet Glob. Heal.	No specific focus on specialists or the professions
MBZFL5AC	2013	Bello, Jennifer K; A	Perceptions of a reproductive health se	Patient Educ. Coun	No specific focus on specialists or the professions
2Z8QE6TG	2021	Belrhiti, Zakaria; B	How medical dominance and interprofe	BMJ Glob. Heal.	
EN84ATAX	2012	Benagiano, Giusep	The special programme of research in h	Gynecol. Obstet. In	No specific focus on specialists or the professions
IIG86VV7	2017	Benavides, Christia	Prospects and challenges in human fac	Acta Colomb. Cuid.	No info on specialists' contribution to health systems/population health, or their governance
HDC6AWBF	2023	Benbassat, Jochan	Teaching Professional Attitudes and Basic Clinical Skills to		No specific focus on specialists or the professions
V8YI2QZS	2023	Bencheikh, Nissma	Vascular Surgery in Low-Income and Mi	Ann. Vasc. Surg.	No info on specialists' contribution to health systems/population health, or their governance
QBITLCHY	2022	Bengo, Eva Maria M	Sufficient informed consent to medical	MALAWI Med. J.	No specific focus on specialists or the professions
7RHM5DSL	2023	Benová, Lenka; Ser	Obstetric referrals, complications and	BMJ Open	No specific focus on specialists or the professions
49SPWM23	2006	Berendsen, Annett	Collaboration with general practitioners : preferences of m		Not on LMICs
QIW9643U	2015	Bergström, Staffan	Training non-physician mid-level provid	Best Pract. Res. Clin. Obstet. Gynaecol.	
I5AQ4RCF	2015	Bergström, Staffan	Workforce Innovations to Expand the Capacity for Surgical S		No info on specialists' contribution to health systems/population health, or their governance
27UZ7SS3	2018	Berhe, Abadi Kidan	Prevalence of hypertensive disorders o	BMC Pregnancy Ch	No info on specialists' contribution to health systems/population health, or their governance
ZC2HVY3A	2012	Bernat, James L	Ethical issues in neurology: Third edition		No specific focus on specialists or the professions
K7Z8INHI	2021	Bernstein, Adrien N	Assessment of Prostate Cancer Treatm	JAMA Oncol.	Not on LMICs
IP3HMUFC	2010	Berry, Nicole S	Unsafe motherhood : Mayan maternal mortality and subject		No specific focus on specialists or the professions
QEU2TF2K	2018	Betrán, Ana Pilar; E	Provision of medical supply kits to impr	Lancet Glob. Heal.	No specific focus on specialists or the professions
NLH8QB84	2018	Betrán, Ana Pilar; T	Interventions to reduce unnecessary ca	Lancet (London, En	No specific focus on specialists or the professions
6UYFCKTE	2024	Beyrer, Chris; Kam	Under threat: the International AIDS So	Lancet	No info on specialists' contribution to health systems/population health, or their governance
K9BVJ2RA	2020	Bhan, Nandita; Mc	Access to women physicians and uptak	EClinicalMedicine	No specific focus on specialists or the professions
UPD7ULPR	2010	Bhana, Arvin; Peter	Implementing the World Health Report	Int. Rev. Psychiatry	No specific focus on specialists or the professions
PGTAW97T	2021	Bhatia, M; Dwivedi	Pro-poor policies and improvements in	BMC Pregnancy Ch	No specific focus on specialists or the professions
D4YE2LY5	2024	Bhugra, Dinesh; Lie	World Psychiatric Association-Asian Jo	Asian J. Psychiatr.	No specific focus on specialists or the professions
A6E5VZDA	2024	Bhugra, Dinesh; Sm	World Psychiatric Association-Asian Jo	Asian J. Psychiatr.	Other
J58FVYDA	2023	Bhugra, Dinesh; Sm	World Psychiatric Association-Asian Jo	Asian J. Psychiatr.	Other
V4FZL8KE	2005	Bhutta, Zulfiqar A;	Community-based interventions for imp	Pediatrics	No specific focus on specialists or the professions
5LK6M32I	2003	Bigal, Marcelo E; R	Burden of migraine in Brazil: estimate o	Headache	No specific focus on specialists or the professions
8H4URW8K	2018	Bijlmakers, Leon; C	The cost of providing and scaling up surg	Health Policy Plan.	No info on specialists' contribution to health systems/population health, or their governance
YEZ3VNS6	2023	Bijok, Benjamin; Ja	Guidelines on human factors in critical	Anaesth. Crit. Care	No specific focus on specialists or the professions
WCCRNDCD	2024	Bîlbîie, Abigaela; P	Investigating Physicians' Adoption of Te	Healthc.	No info on specialists' contribution to health systems/population health, or their governance
UNUB2TXK	2021	Binyaruka, Peter; B	Supply-side factors influencing informa	Health Policy Plan.	
AZZ2G6FN	2021	Birch, Andrea A; Sp	Historically Black Schools of Medicine	Acad. Radiol.	No specific focus on specialists or the professions
K6NZB6TT	2018	Birhanu, Zewdie; C	Ethiopian women's perspectives on ant	Matern. Child Nutr.	No specific focus on specialists or the professions
7BW4C9FP	2022	Black, Kirsten I; Mi	Interconception Health: Improving Equ	Semin. Reprod. Me	No specific focus on specialists or the professions
F43QR3CT	2021	Blakley, Christoph	`I have been obliged to Send Nassaw': a	Med. Hist.	No specific focus on specialists or the professions
5C7D664X	2021	Block, Miguel Á Gor	Health Systems in Transition: Mexico		No info on specialists' contribution to health systems/population health, or their governance
KBGRG5XZ	2020	Blom, Lisa	mHealth for image-based diagnostics o	Glob. Health Action	No specific focus on specialists or the professions
TUYZA52P	2022	Blonde, Lawrence;	American Association of Clinical Endoc	Endocr. Pract.	No specific focus on specialists or the professions
6Q76QJ6Q	1986	Bloom, Gerald; Lai	Dactors, private paractice and primary	Health Policy Plan.	
5DS8DMPI	2018	Bloomer, Fiona; Pi	Reimagining global abortion politics: A social justice perspe		No specific focus on specialists or the professions
CZBZZY7F	2023	Blout Zawatsky, Ca	Elective genomic testing: Practice reso	J. Genet. Couns.	No specific focus on specialists or the professions
HCXRBYYL	2023	Blum, Torsten Gerr	European Respiratory Society guideline	Eur. Respir. J.	No info on specialists' contribution to health systems/population health, or their governance
IH248INI	2022	Bochatay, Naike; B	Towards equitable learning environmer	Med. Educ.	No info on specialists' contribution to health systems/population health, or their governance
3FASKAW4	2008	Bodnar, Richard J	Endogenous opiates and behavior: 2007	Peptides	No specific focus on specialists or the professions
66HHLNKF	2020	Bogin, Barry	Cambridge Studies in Biological and Evolutionary Anthropo		No specific focus on specialists or the professions
97SNQ3I7	2023	Bognini, Maeve S; C	Assessing the impact of anaesthetic an	Health Policy Plan.	No info on specialists' contribution to health systems/population health, or their governance
6YAJSM9U	2015	Bohren, Meghan A;	The Mistreatment of Women during Chi	PLoS Med.	No specific focus on specialists or the professions
4UN6TJYG	2017	Bohren, Meghan A;	Mistreatment of women during childbir	Reprod. Health	No specific focus on specialists or the professions
Z6VWKFN8	2016	Bolkan, Håkon A; H	The Surgical Workforce and Surgical Pr	World J. Surg.	No specific focus on specialists or the professions
7VSLDSRS	2019	Bolton, W S; Arupa	Disseminating technology in global surg	Br. J. Surg.	No info on specialists' contribution to health systems/population health, or their governance
7NVR8FQN	2012	Bone, Kerry; Mills,	Principles and practice of phytotherapy: Modern herbal me		No specific focus on specialists or the professions
HWVJS9S2	2024	Bonet, Mercedes;	Maternal and perinatal health research	BMJ Glob. Heal.	No specific focus on specialists or the professions
N4FKBRM8	2022	Bonilla, Carolina; A	Medical geneticists, genetic diseases a	Per. Med.	No info on specialists' contribution to health systems/population health, or their governance
5S59JMPQ	2023	Bonnechère, Brun	Current Technology Developments Car	Sensors	No specific focus on specialists or the professions
5AYAQSE2	2023	Bonner, Sidra N; Po	Surgical Care for Racial and Ethnic Min	Ann. Surg.	Not on LMICs
Z3APYIGK	2011	Bosch-Capblanch,	Managerial supervision to improve prim	Cochrane Databas	No specific focus on specialists or the professions
RSL2WMRA	2021	Bosire, Edna N; No	Pathways to care for patients with type	Glob. Heal. Sci. Pra	No info on specialists' contribution to health systems/population health, or their governance
PD9RTUHI	2007	Boswell, Mark V; Tr	Interventional techniques: Evidence-ba	Pain Physician	No specific focus on specialists or the professions
QVA8EQEE	2020	Botezat, Alina; Mor	Brain drain from Romania: What do we	East. J. Eur. Stud.	
7KD3XSZX	2021	Botti-Lodovico, Yo	The Origins and Future of Sentinel: An E	Viruses	No specific focus on specialists or the professions
AFFGZYY8	2024	Bottomley, Jennife	Geriatric Rehabilitation: A Textbook for the Physical Therap		No specific focus on specialists or the professions
NRSDFTYC	2016	Boucher, Julie; Kon	Understanding inequalities of maternal	Int. J. Environ. Res.	No specific focus on specialists or the professions
C7QKNRT6	2019	Bourke, Christophe	Transforming institutional racism at an	Aust. Heal. Rev.	No specific focus on specialists or the professions
7W3VH3L6	2006	Bower, Peter; Gilb	Collaborative care for depression in pri	Br. J. Psychiatry	No specific focus on specialists or the professions
FEFBKH2E	2022	Bozzola, Elena; Spi	The Use of Social Media in Children and	Int. J. Environ. Res.	No specific focus on specialists or the professions
IEI3B3NK	2017	Braithwaite, Jeffre	Health systems improvement across the globe: Success sto		No specific focus on specialists or the professions
ZFECKPUD	2015	Braithwaite, Jeffre	Discussion: Integrating and Synthesizing the Evidence Acro		No specific focus on specialists or the professions
D53ULQN9	2017	Braithwaite, Jeffre	Healthcare Reform, Quality and Safety: Perspectives, Partic		No specific focus on specialists or the professions
YWGVYEBZ	2013	Branca, Margherita	A manual for cervical cancer screening and control: Princip		No specific focus on specialists or the professions
XEPD979E	2024	Brandao, Gabriela	Women in Surgical Residency Programs	J. Surg. Res.	
TG9X7SFI	2018	Brandão, Glauber S	Association of sleep quality with excess	Multidiscip. Respir.	No specific focus on specialists or the professions
49J4ATHN	2012	Brannan, Sophie; C	Medical Ethics Today The BMA's Handbook of Ethics and Lav		No specific focus on specialists or the professions
L5UVA4GW	2019	Brant, Jeannine M;	Global Survey of the Roles, Satisfaction	J. Palliat. Med.	No specific focus on specialists or the professions
6ULHDY8S	2013	Bratzler, Dale W; D	Clinical practice guidelines for antimic	Am. J. Heal. Pharm.	No info on specialists' contribution to health systems/population health, or their governance
JM2P7W6W	2024	Brauer, Michael; R	Global burden and strength of evidence	Lancet	No specific focus on specialists or the professions
QA2N7R9K	2023	Bray, Francesca	Technology and gender: Fabrics of power in late imperial Ch		No specific focus on specialists or the professions
RTAWQ3WH	2010	Breathnach, Steph	Drug Reactions		No specific focus on specialists or the professions
ZCDTZ9CL	2016	Breman, Joel G; He	Discovery and Description of Ebola Zai	J. Infect. Dis.	No specific focus on specialists or the professions

Appendix 4

Table S4: List of papers excluded, with reason

KZWFC5K4	2014	Breuer, Erica; De S	Using workshops to develop theories of	Int. J. Ment. Health	No specific focus on specialists or the professions
T64HIMSG	2022	Brewer-Smyth, Kat	Adverse Childhood Experiences: The Neuroscience of Traun		No specific focus on specialists or the professions
VIMJN9Z7	2018	Brieger, David; Am	National Heart Foundation of Australia	Hear. Lung Circ.	No info on specialists' contribution to health systems/population health, or their governance
4KZBKEZJ	2014	Briggs, Gerald G; Fr	Drugs in pregnancy and lactation: A reference guide to fetal		No specific focus on specialists or the professions
53ABFEZ4	2011	Britnell, Mark	The role of the 'specialist' in healthcare	Clinical Medicine	
YF9ZAVNE	2017	Brockington, Ian	The psychoses of menstruation and childbearing		No specific focus on specialists or the professions
IXAXGFLV	2011	Brody, Tom	Clinical Trials: Study Design, Endpoints and Biomarkers, Dr		No specific focus on specialists or the professions
QJRWRF76	2023	Brokensha, Susan;	AI in and for Africa: A Humanistic Perspective		No specific focus on specialists or the professions
FGDMUG7P	2016	Bronstein, Janet M	Preterm birth in the United States: A sociocultural approach		Not on LMICs
AVH7KIFS	2021	Brown, Patrick	On vulnerability: A critical introduction		No specific focus on specialists or the professions
HUZP2NYK	2023	Brown, Taylor; Jord	Developing Early Pathways to Otolaryng	Curr. Otorhinolaryn	Not on LMICs
CDNRBXGX	2020	Buchbinder, David	Predictors of Loss to Follow-Up Among F	Biol. blood marrow	No specific focus on specialists or the professions
V2UVR5AV	2016	Buck, Trevor; Kirkh	The Ombudsman Enterprise and Administrative Justice		No specific focus on specialists or the professions
IW67SZ5J	2014	Buckle, Jane	Clinical aromatherapy: Essential oils in healthcare		No info on specialists' contribution to health systems/population health, or their governance
LVUSAMZB	2022	Budhwani, Henna;	mHealth Phone Intervention to Reduce	Int. J. Womens. Hea	No specific focus on specialists or the professions
HECNZRB7	2021	Buendía, Jefferson	Budget impact analysis of high-flow nas	Curr. Med. Res. Opi	No specific focus on specialists or the professions
XPVMM8Y7	2018	Bujan, Louis	ZIKA virus, human genital tract and sexu	Bull. L Acad. Natl. M	No specific focus on specialists or the professions
D3Q6RKEP	2020	Bukhman, Gene; M	The Lancet NCDI Poverty Commission:	Lancet	
736PVXGU	2022	Bulamba, F; Bisege	Development of the anaesthesia workfo	South. AFRICAN J. Anaesth. Analg.	
4WW77QYN	2024	Bulcha, Gebeyehu;	Effectiveness of a Mobile Phone Messag	JMIR Res. Protoc.	No specific focus on specialists or the professions
CP8S58AN	2011	Burch, V C; McKinle	Career intentions of medical students t	Educ. Health (Abingdon).	
SIGZA89Z	2022	Burks, Ciersten A; F	Strategies to Increase Racial and Ethni	Otolaryngol. NECK S	Not on LMICs
7CXWYNJQ	2023	Burn, Emily; Waring	The evaluation of health care leadershi	Leadersh. Heal. Ser	No specific focus on specialists or the professions
LDG272FC	2009	Burnham, Gilbert M	Doctors leaving 12 tertiary hospitals in	Soc. Sci. Med.	
MA3R92V5	2020	Buxton, Julia; Marg	The Impact of Global Drug Policy on Women: Shifting the Ne		No specific focus on specialists or the professions
S9QWRA65	2024	Byers, Richard J; By	Development of an online teaching plat	Front. Med.	No info on specialists' contribution to health systems/population health, or their governance
BFS5XA9I	2020	Byhoff, Elena; Kang	A Society of General Internal Medicine	J. Gen. Intern. Med.	Other
7AHAJ77K	2022	Cable, Jennifer; Fa	Lessons from the pandemic: Respondin	Ann. N. Y. Acad. Sci.	No specific focus on specialists or the professions
LY22TGXF	2022	Cai, Yiyuan; Gong,	Residual Effect of Texting to Promote M	JMIR mHealth uHea	No specific focus on specialists or the professions
R5M9GVD8	2024	Çalış, Fatih; Şimşe	Factors Affecting Turkish Medical Stude	World Neurosurg.	
X4GA7PVK	2018	Calkins, Hugh; Hin	2017 HRS/EHRA/ECAS/APHRS/SOLAEC	Europace	No specific focus on specialists or the professions
YHA8GKD7	2017	Calkins, Hugh; Hin	2017 HRS/EHRA/ECAS/APHRS/SOLAEC	Hear. Rhythm	No specific focus on specialists or the professions
2ED2BKM5	2014	Calland, James For	Burn management in sub-Saharan Afric	Burns	No info on specialists' contribution to health systems/population health, or their governance
M2ZH4SYH	2015	Campanella, Nand	Medical teleconsultation to general pra	Eur. J. Intern. Med.	
LAAJW9SG	2016	Campbell, Oona M	The scale, scope, coverage, and capabi	Lancet	No specific focus on specialists or the professions
8LYYEN69	2019	Cao, Yang; Zhen, Fe	Public transportation environment and	Int. J. Environ. Res.	No specific focus on specialists or the professions
GDQ4DX27	2005	Caparros-Lefebvre	Atypical unclassifiable parkinsonism or	Mov. Disord.	No specific focus on specialists or the professions
GJW8RV2W	2023	Carey, Megan E; Dy	Global diversity and antimicrobial resis	Elife	No specific focus on specialists or the professions
F47YMHC3	2017	Carlos, Ruth C; Sic	Capacity for Cancer Care Delivery Res	J. Am. Coll. Radiol.	No info on specialists' contribution to health systems/population health, or their governance
DPXFSH3U	2009	Carmeli, Abraham;	The relational underpinnings of quality i	Soc. Sci. Med.	Not on LMICs
RXLA2C34	2013	Carod-Artal, Franc	Burnout syndrome in an international setting		No specific focus on specialists or the professions
V48PMKJL	2010	Carolan, Mary	Pregnancy health status of sub-Saharar	Midwifery	No specific focus on specialists or the professions
F7MEDIJM	2020	Carroll, Anna L; Ch	Medical Student Values Inform Career I	J. Surg. Res.	Not on LMICs
S5IETD7Y	2019	Carson, Savanna L;	Health Challenges and Assets of Forest	Ecohealth	No specific focus on specialists or the professions
QT3X858X	2018	Carter, Chris	Critical care nursing in resource limited environments		No specific focus on specialists or the professions
FVL5XUTC	2013	Casey, Frances; Go	Disparities in contraceptive access and	Semin. Reprod. Med	No specific focus on specialists or the professions
ZIQ8H7FQ	2022	Castaldo, Matteo;	Headache onset after vaccination agai	J. Headache Pain	No specific focus on specialists or the professions
AP2RCZ5N	2016	Castañeda-Guillot	Pediatric Gastroenterology in Cuba: Ev	MEDICC Rev.	
9VVAWEDE	2009	Castleman, Laura;	Providing Abortion in Low-Resource Settings		No specific focus on specialists or the professions
3BXMJK4I	2018	Catalá-López, Ferr	Mapping of global scientific research in	PLoS One	No specific focus on specialists or the professions
ZEPYRSVL	2021	Cesar, Juraci A; Bla	Antenatal care in Southern Brazil: Cove	Prev. Med. (Baltim)	No specific focus on specialists or the professions
6ZSK9AVI	2023	Chacko, Jose; Paw	Controversies in Critical Care		No info on specialists' contribution to health systems/population health, or their governance
EBWBBAU4	2019	Chan, Jason Y K; W	Challenges in the delivery of surgical ca	Curr. Opin. Otolary	No info on specialists' contribution to health systems/population health, or their governance
ZK9AS3AR	2011	Chan, Kay-Fei; Tan	Occupational rehabilitation in Singapo	J. Occup. Rehabil.	No specific focus on specialists or the professions
XD4URZEQ	2024	Chan, Olivia S K; La	Why prescribe antibiotics? A systemati	Soc. Sci. Med.	No specific focus on specialists or the professions
J7NJ8DBK	2024	Chandrasekar, Abi	mHealth interventions for postpartum f	PLOS Glob. Public H	No specific focus on specialists or the professions
Z8QLXS5W	2008	Chanussot-Deprez	Telemedicine in wound care.	Int. Wound J.	No specific focus on specialists or the professions
SJBPTZDX	2012	Chapman, Ann L N;	Good practice recommendations for ou	J. Antimicrob. Chem	No specific focus on specialists or the professions
ALCBIYA6	2018	Charanthimath, Ur	The feasibility of task-sharing the identi	Reprod. Health	No specific focus on specialists or the professions
CQI8VNZ8	2023	Chareyron, Sylvain	Cream skimming and discrimination in	Health Econ.	Not on LMICs
2SIY9GYB	2024	Charoenraj, Pornar	Pediatric anesthesia in Mongolia and Th	Pediatr. Anesth.	
9QRTID9B	2022	Chassagne, Franço	Polynesian medicine used to treat diarr	J. Ethnopharmacol.	No specific focus on specialists or the professions
RBEWV92J	2020	Chaulagain, A; Pac	WHO Mental Health Gap Action Progra	Int. J. Ment. Health	No specific focus on specialists or the professions
58W77ADX	2017	Chaves, Nadia J; Pa	The Australasian Society for Infectious I	Med. J. Aust.	Not on LMICs
ZHCIXDDE	2013	Chavkin, Wendy; Le	Conscientious objection and refusal to	Int. J. Gynecol. Obs	No specific focus on specialists or the professions
6WXQT677	2010	Chavkin, Wendy; M	The Globalization of Motherhood: Deconstructions and rec		No specific focus on specialists or the professions
VQRZEGTD	2020	Chawla, Rajeev; M	RSSDI-ESI clinical practice recommen	Indian J. Endocrinol	No info on specialists' contribution to health systems/population health, or their governance
XIHN8QPN	2016	Chehade, M J; Gill,	Building multidisciplinary health workfo	Best Pract. Res. Cli	No info on specialists' contribution to health systems/population health, or their governance
LJQ5YAAY	2021	Chekijian, Sharon;	Healthcare in transition in the Republic	Int. J. Emerg. Med.	No info on specialists' contribution to health systems/population health, or their governance
GJD2EH9N	2022	Chelmow, David; B	Executive Summary of the Uterine Canc	Obstet. Gynecol.	No specific focus on specialists or the professions
IL2P8SYT	2022	Chemali, Zeina; He	Developing a Dual Residency Program i	J. Neuropsychiatry	Not on LMICs
QVGG4B6J	2024	Chen, Jiang-Ping; D	Factors influencing turnover intention a	Int. Nurs. Rev.	No specific focus on specialists or the professions
PZM59T7A	2022	Chen, Jiao-Jiao; Hu	Data mining for adverse drug reaction s	Int. J. Clin. Pharm.	No specific focus on specialists or the professions
WE5GUYUV	2021	Chen, Sharon C-A;	Global guideline for the diagnosis and m	Lancet. Infect. Dis.	No info on specialists' contribution to health systems/population health, or their governance
S28EXQ4N	2023	Chen, Yanhua; Wu,	Radiology Residents' Perceptions of Art	J. Med. INTERNET R	No specific focus on specialists or the professions
DPCLGBDB	2020	Cheng, Harriet S; K	Demographics of New Zealand women	N. Z. Med. J.	Not on LMICs
QN65VJ6W	2024	Cheng, Lei; Cai, Siy	In Every Detail: Spiritual Care in Pediat	J. Pain Symptom Ma	No specific focus on specialists or the professions
9VXTGLRH	2022	Cheng, Leo; Cheng	Global Surgery and Mercy Ships.	J. oral Biol. craniofa	No info on specialists' contribution to health systems/population health, or their governance
8FPNACIK	2014	Cherny, N I	ESMO clinical practice guidelines for th	Ann. Oncol.	No specific focus on specialists or the professions
RPLKR8PM	2018	Chetwood, John Da	High-Fidelity Realistic Acute Medical S	Simul. Healthc.	No info on specialists' contribution to health systems/population health, or their governance
IQQLBY4Y	2015	Cheung, Jennifer; V	Analysis of the Science and Technology	Technologies	Not on LMICs
QIPVB7L4	2020	Chevance, A; Gour	Ensuring mental health care during the	Encephale.	Not on LMICs
7IKABYXD	2014	Chhabra, Pragti	Maternal near miss: An indicator for ma	Indian J. Communit	No specific focus on specialists or the professions
VQE63685	2022	Chidinma, Ajegbo U	Prevalence of Cervical Cancer in Enugu	Texila Int. J. Public H	No specific focus on specialists or the professions
VAG9NZWH	2019	Chimatiro, George	Scoping review of acute stroke care ma	BMC Health Serv. R	No specific focus on specialists or the professions
EVIRTKZ6	2021	Ching, Peñafrancia	Preparation, roles, and responsibilities	Disabil. Rehabil.	No specific focus on specialists or the professions
8NMW3KIJ	2018	Chippaux, Jean-Ph	Yellow fever in Africa and the Americas:	J. Venom. Anim. Tox	No specific focus on specialists or the professions
PPHCPSIY	2023	Chiu, Joanne Wing	Clinical Guidance on the Monitoring an	Drug Saf.	No specific focus on specialists or the professions
RZ2A7YXI	2021	Chiware, Tendai M	IVF and other ART in low- and middle-in	Hum. Reprod. Upda	No specific focus on specialists or the professions
IQUH2NT8	2016	Chokotho, Linda; Ja	A review of existing trauma and muscul	Injury	No info on specialists' contribution to health systems/population health, or their governance
3D2UE35Q	2020	Chong, Ka Chun; Le	Association between meteorological va	J. Infect.	No specific focus on specialists or the professions
3QXRI7CI	2013	Choo, Shelly; Papa	Quality improvement activities for surg	World J. Surg.	No info on specialists' contribution to health systems/population health, or their governance
4XUIEKLN	2016	Chootipongchaivat	Vaccination program in a resource-limi	Vaccine	No specific focus on specialists or the professions
YX2FWXFN	2012	Chopra, Mickey; Sh	Strategies to improve health coverage a	Lancet	No specific focus on specialists or the professions
678ZHAJZ	2024	Chow, Andrea J; Sa	Family-centred care interventions for c	Heal. Expect.	No specific focus on specialists or the professions
ZSRJZGLQ	2021	Chowdhury, Avijit;	The Role of Image Quality in Telehealth:	Pacific Asia J. Assoc	No info on specialists' contribution to health systems/population health, or their governance
PTPNRPRE	2024	Chowdhury, Devya	Addressing Disparities in Pediatric Con	J. Am. Heart Assoc.	No specific focus on specialists or the professions
MLPEIKNS	2020	Christen, Peter; Ra	Linking Sensitive Data: Methods and Techniques for Practic		No specific focus on specialists or the professions
L4X3RYAA	2021	Christiansen, Brya	Handbook of research on applied social psychology in multi		No specific focus on specialists or the professions

Appendix 4

Table S4: List of papers excluded, with reason

MZRCPKA9	2020	Chu, Hin; Chan, Jas	Comparative tropism, replication kinet	The Lancet. Microb	No specific focus on specialists or the professions
QMY4PR8N	2022	Chu, Kathryn M; Bu	Colorectal Surgery Practice, Training, a	Clin. Colon Rectal S	No info on specialists' contribution to health systems/population health, or their governance
9ZWFC5WU	2022	Chua, Gilbert T; Kw	Epidemiology of Acute Myocarditis/Peri	Clin. Infect. Dis. an	Not on LMICs
FHZGE4FP	2017	Ciapponi, Agustín;	Delivery arrangements for health syster	Cochrane Databas	No specific focus on specialists or the professions
JHEUIKDK	2019	Cicolello, Katherin	Multiple Stakeholders' Perspectives Re	J. Pain Symptom Ma	No specific focus on specialists or the professions
U9GWI84D	2020	Çınaroğlu, Songül	Equity and healthcare reform in developing economies: The		No specific focus on specialists or the professions
JEAWTBP9	2016	Claiborne, Anne; E	Mitochondrial Replacement Techniques: Ethical, Social, ar		No info on specialists' contribution to health systems/population health, or their governance
E3RGSCRP	2024	Clark, Susan G; An	Policy Sciences and the Human Dignity	Nat. Resour. Manag	No specific focus on specialists or the professions
KNMHCDK5	2007	Clark, Wesley; Sha	Misoprostol for uterine evacuation in in	Expert Rev. Obstet.	No specific focus on specialists or the professions
EV3BGNJJ	2014	Coast, Ernestina; J	Maternity care services and culture: A s	PLoS One	No specific focus on specialists or the professions
WU2P2KK4	2018	Coccolini, Federic	WSES worldwide emergency general su	World J. Emerg. Sur	No info on specialists' contribution to health systems/population health, or their governance
MB9XIQKN	2022	Coccolini, Federic	The LIFE TRIAD of emergency general surgery.		No info on specialists' contribution to health systems/population health, or their governance
BG8U2TBH	2024	Cockburn, Neil; Os	Clinical decision support systems for m	eClinicalMedicine	No specific focus on specialists or the professions
44NGUV9T	2020	Coetzee, J F; Kluyts	Burnout and areas of work-life among a	South. African J. An	No info on specialists' contribution to health systems/population health, or their governance
EBJE9UN4	2020	Cohen, Alan A; Ken	Lack of consensus on an aging biology p	Mech. Ageing Dev.	No specific focus on specialists or the professions
VP46M5BW	2023	Cohen, Mírian; Car	Mental health outcomes in frontline he	Compr. Psychiatry	No info on specialists' contribution to health systems/population health, or their governance
FXCT4LR2	2016	Colaci, Daniela; C	mHealth Interventions in Low-Income C	Ann. Glob. Heal.	No specific focus on specialists or the professions
WMJYYTB7	2022	Coleman, E; Radix,	Standards of Care for the Health of Trar	Int. J. Transgender H	No specific focus on specialists or the professions
X4VUDUWU	2021	Colomar, Mercede	Collaborative model of intrapartum car	BMJ Open	No info on specialists' contribution to health systems/population health, or their governance
NYAM43AV	2015	Colomar, Mercede	Barriers and Promoters of an Evidenced	Matern. Child Heal	No specific focus on specialists or the professions
8BEPSA6H	2012	Colombini, Manuel	An integrated health sector response to	BMC Public Health	No specific focus on specialists or the professions
HQY4PJDN	2023	Comelli, Agnese; G	Schistosomiasis in non-endemic areas:	Infection	Not on LMICs
3NWG7HES	2022	Comninellis, Nick;	Contemporary issues in global medicine and moving toward		No specific focus on specialists or the professions
3CQI8LM8	2023	Conley, Brooke; Bu	What are the core recommendations fo	BMC Rheumatol.	No info on specialists' contribution to health systems/population health, or their governance
KKNJCI7H	2010	Connell, John	Migration and the globalisation of health care: The health w		No specific focus on specialists or the professions
U35XDWUC	2021	Conti, P; Caraffa, A	The British variant of the new coronavirus-19 (Sars-Cov-2) s		No specific focus on specialists or the professions
6574E7D7	2022	Cooper, Ryan; Hou	Chapter 10: Treatment of active tuberc	Can. J. Respir. Crit.	No info on specialists' contribution to health systems/population health, or their governance
W6TKIQRY	2023	Copelton, Denise A	The Sociology of Health, Healing, and Illness: 11th Edition		No specific focus on specialists or the professions
S2KX5U8K	2000	Corbie-Smith, G; F	The intersection of race, gender, and pr	J. Natl. Med. Assoc.	No specific focus on specialists or the professions
9CRAHJYQ	2014	Cordella, Marisa; F	Behavioural oncology: Psychological, communicative, and s		No info on specialists' contribution to health systems/population health, or their governance
3L243IWQ	2018	Cormick, Gabriela;	Are women with history of pre-eclamps	BMC Pregnancy Ch	No specific focus on specialists or the professions
X6RFWMQE	2019	Correia, Jorge Césa	Diabetes management in Guinea Bissa	Pan Afr. Med. J.	No specific focus on specialists or the professions
K593PUH4	2019	Correia, M Isabel T	The Practical Handbook of Perioperative Metabolic and Nut		No specific focus on specialists or the professions
VEJU33GJ	2016	Cosminsky, Sheila	Midwives and mothers: The medicalization of childbirth on a		No specific focus on specialists or the professions
D4HJEXC5	2004	Costa, Lucio G; Ste	Structural Effects and Neurofunctional	Pharmacol. Rev.	No specific focus on specialists or the professions
P5DKBCAT	2009	Coté, Charles J; Le	Pharmacokinetics and Pharmacology of Drugs Used in Child		No specific focus on specialists or the professions
V6JXW8LY	2020	Cox, Megan; Masur	A successful hybrid emergency medicin	African J. Emerg. Me	No info on specialists' contribution to health systems/population health, or their governance
9LUR6HRJ	2008	Coyne, C P	Comparative Diagnostic Pharmacology: Clinical and Resea		No specific focus on specialists or the professions
IKPLDV54	2021	Crafford, Lucille; W	Exploring Factors Associated With the N	Front. Med.	No specific focus on specialists or the professions
DWA725PY	2023	Crawford, Ana Mar	Global critical care: a call to action.	Crit. Care	Other
H6BA67KD	2017	Croskerry, Pat; Cos	Diagnosis: Interpreting the shadows		No specific focus on specialists or the professions
L8FDARHN	2017	Crowe, Sophie; Cla	'You do not cross them': Hierarchy and	Soc. Sci. & Med.	Not on LMICs
BWQ5T8L5	2011	Cruz, Norma I; Kor	Rising medical liability cost and patient	P. R. Health Sci. J.	No info on specialists' contribution to health systems/population health, or their governance
K8XR5PBZ	2020	Cui, Zhen; Gao, Lia	Core competencies in neurocritical ca	BMJ Open	No info on specialists' contribution to health systems/population health, or their governance
TMHB49DA	2009	Cunningham, Peter	High medical cost burdens, patient trus	J. Gen. Intern. Med.	No specific focus on specialists or the professions
FXK9LYR3	2024	Curry, Susan J; Bel	Essential health care services addressing intimate partner		No specific focus on specialists or the professions
W9YMZWJV	2023	Czech, Herwig; Hil	The Lancet Commission on medicine, N	Lancet	No specific focus on specialists or the professions
GRDFGDWX	2019	D'Angelo Seixas, Pa	Physicians' commuting in five health re	Cad. Saude Publica	No specific focus on specialists or the professions
JPS2P4IN	2024	D'Souza, Karishma	A qualitative assessment of barriers and	Hum. Resour. Healt	No specific focus on specialists or the professions
6FWEEBFY	2016	da Costa, Élide Sba	Analysis of the costs and quality of card	Rev. Assoc. Med. Br	No info on specialists' contribution to health systems/population health, or their governance
6JVBURV5	2014	da Cruz, Thalita Alv	ICD-10 mental and behavioural disorde	Int. Rev. Psychiatry	No specific focus on specialists or the professions
D3X2V7VB	2023	da Silva Neto, Prisc	Health professionals' knowledge about	Monash Bioeth. Rev	No specific focus on specialists or the professions
JD2TEFLJ	2022	da Silva, Alessandr	An experience with the use of WISN too	Hum. Resour. Healt	No info on specialists' contribution to health systems/population health, or their governance
2KY37FCQ	2021	Dadi, Abel Fekadu;	"We do not know how to screen and pro	Int. J. Ment. Health	No info on specialists' contribution to health systems/population health, or their governance
PGKGKDBV	2019	Dalglish, Sarah L; S	A framework for medical power in two c	Glob. Public Health	
5HJC4WVW	2020	Damani, Anuja; Gh	Approaches and Best Practices for Man	Indian J. Palliat. Ca	No specific focus on specialists or the professions
7PUMCRQJ	2017	Dan, Amitava; Mon	Clinical course and treatment outcome	Asian J. Psychiatr.	No specific focus on specialists or the professions
4KDWFXIE	2024	Dang, Patrick; Cha	Proxy use of patient portals on behalf of	Digit. Heal.	Not on LMICs
YXTTIJ4B	2020	Daniels, Kimberly N	The Scale-Up of the Global Surgical Wo	World J. Surg.	
IMPDZ7QC	2022	Danquah, Michael	Emerging Nanomedicines for Diabetes Mellitus Theranostic		No specific focus on specialists or the professions
34S5HSZR	2017	Danziger, Larry; Ito	Clindamycin and lincomycin		No specific focus on specialists or the professions
YV89G69L	2020	Daoud, Nihaya; Kra	Patterns of healthcare services utilizat	PLoS One	No specific focus on specialists or the professions
DJ95HLV5	2020	Darabi, Negar; Hos	System dynamics modeling in health an	Syst. Dyn. Rev.	No specific focus on specialists or the professions
U9AUF3YX	2018	Darwinkel, Mariann	Evaluating the role of clinical officers in	Hum. Resour. Health	
3AHWNFNY	2023	Das, Jishnu; Do, Qu	The Prices in the Crises: What We Are L	J. Econ. Perspect.	No specific focus on specialists or the professions
DXREKY3E	2023	Das, Shukanto; Gra	Task shifting healthcare services in the	PLOS Glob. Public H	No specific focus on specialists or the professions
CKCKGG8A	2012	Dasgupta, Amitava	Resolving Erroneous Reports in Toxicology and Therapeutic		No specific focus on specialists or the professions
MUSSBAKN	2020	Davahli, Mohamma	A system dynamics simulation applied t	Int. J. Environ. Res.	No specific focus on specialists or the professions
PYQE4UHM	2021	Dave, Nandini; Var	Challenges and Opportunities Facing P	Paediatr. Anaesth.	No info on specialists' contribution to health systems/population health, or their governance
HKB5KPJU	2020	Davey, Kevin; Jena	Postgraduate Diploma in Emergency M	Clin. Teach.	No info on specialists' contribution to health systems/population health, or their governance
29XNR4BG	2013	Davey, Peter; Brow	Interventions to improve antibiotic pres	Cochrane Databas	No specific focus on specialists or the professions
KZDSUURB	2019	Davids, Eugene Lee	Child and adolescent mental health in A	J. Child Adolesc. Me	No specific focus on specialists or the professions
F9DW3P2B	2018	Davidson, Melissa	Thiazolidinedione drugs in the treatmen	Crit. Rev. Toxicol.	No specific focus on specialists or the professions
K35X3U7Y	2018	Davies, Justine I.; V	What is the minimum number of specia	BMJ Global Health	
AWZBZJAL	2024	Davies, Theophilus	Medical Geology of Africa		No specific focus on specialists or the professions
RXT43KB8	2017	Dawson, Angela J; N	Medical termination of pregnancy in ge	Reprod. Health	Not on LMICs
2FV6GILS	2016	Dayan, Gustavo H;	Staphylococcus aureus: the current sta	Expert Rev. Vaccine	No specific focus on specialists or the professions
PXZRYJY2	2022	de Araujo, Carolina	Palliative sedation in patients with adva	Palliat. & Support. C	No specific focus on specialists or the professions
IHSE4QQ9	2022	de Arruda, Camila	Maternal mortality in South region of Br	J. Obstet. Gynaecol	No specific focus on specialists or the professions
ADAJR64A	2014	de Freitas Campos	De Geneve a Belo Horizonte, une histoi	Paedagog. Hist.	
2M4IUJYA	2013	de Jesus Mari, Jair;	Pharmacological and psychosocial mar	Drugs	No specific focus on specialists or the professions
H3LRHC6W	2014	de la Fuente, Juan;	Teaching Psychiatry in Mexico	SALUD Ment.	No info on specialists' contribution to health systems/population health, or their governance
2B87A4ZY	2020	de la Fuente, Sofia;	Evaluation of mental health and occupa	SALUD Ment.	No info on specialists' contribution to health systems/population health, or their governance
YNZSMI8S	2022	de Oliveira, Camila	Health complexity assessment in prima	PLoS One	No specific focus on specialists or the professions
GPJH2QWJ	2013	De Silva, A Pubudu	Migration of Sri Lankan medical special	Hum. Resour. Health	
AY9NESVN	2015	de Souza, Ligia Cor	Medical Specialty Choice and Related	PLoS One	No info on specialists' contribution to health systems/population health, or their governance
PINGBCSG	2010	Deakin, Charles D;	European Resuscitation Council Guide	Resuscitation	No specific focus on specialists or the professions
DFY73T3M	2017	Deb, Shreyasi; Mill	Relations among race/ethnicity, gende	Psychiatr. Rehabil.	No specific focus on specialists or the professions
ESVBFV3F	2023	Deb, Sibnath; Sunn	Community Psychology: Emerging Issues and Challenges		No specific focus on specialists or the professions
VQFSNERV	2012	DeBaun, Michael R	Transition and sickle cell disease.	Pediatrics	No specific focus on specialists or the professions
B8P2B7TR	2020	Debelius, Justine W	Subspecies Niche Specialization in the	mSystems	No info on specialists' contribution to health systems/population health, or their governance
T4DV7YU8	2009	Dedmon, Robert E	The future of primary care in Thailand in	Asian Biomed.	No specific focus on specialists or the professions
IA2ECWNK	2001	Deftereos, S; Lamb	A Java-based electronic healthcare rec	J. Med. Internet Res	No specific focus on specialists or the professions
AT3TGI9V	2020	Degge, Hannah M;	Insights from birthing experiences of fis	Nurs. Inq.	No specific focus on specialists or the professions
PAGFBIJE	2017	Dehn, Richard W; E	Research on the PA profession: The me	J. Am. Acad. Physici	No specific focus on specialists or the professions
Q9G2IHLW	2022	Dejene, Daniel; St	Assessment of core teaching competen	BMJ Open	No specific focus on specialists or the professions
AG7JRNGY	2022	Delaney, Megan M	Does Quality Certification Work? An As	Glob. Heal. Sci. Pra	No specific focus on specialists or the professions
SWTY2YNA	2022	Della Casa, France	Development and Implementation of th	Front. Med.	No specific focus on specialists or the professions
FXLCX8FG	2012	Demaria, Lisa M; C	Non-physician providers of obstetric ca	Hum. Resour. Healt	No specific focus on specialists or the professions

Appendix 4

Table S4: List of papers excluded, with reason

44KM2Q68	2011	DeMello, Jim P; De	Career Satisfaction of Psychiatrists	Psychiatr. Serv.	Not on LMICs
QGT4T5T9	2022	Denadai, Rafael; Lo	Current Concept in Cleft Surgery: Moving Toward Excellenc		No specific focus on specialists or the professions
Q5Q72DIU	2019	Derbew, Miliard	Pediatric surgery in Eastern Africa: The	J. Pediatr. Surg.	No info on specialists' contribution to health systems/population health, or their governance
TEV3SC4C	2012	Derienzo, Christop	Handoffs in the era of duty hours reform	Acad. Med.	Not on LMICs
U64YSBQQ	2009	Deshpande, Amol;	Asynchronous telehealth: A scoping rev	Open Med.	No info on specialists' contribution to health systems/population health, or their governance
28CJV735	2023	Dev, Sukh	Prime Ayurvedic Plant Drugs, Second Edition		No specific focus on specialists or the professions
SUEWXQ5Q	2022	DeVries, Catherine	A global view of pediatric urology.	J. Pediatr. Urol.	
FGPLTM7M	2016	Dew, Kevin; Scott,	Social, political and cultural dimensions of health		No specific focus on specialists or the professions
4BZPPIVR	2023	Dey, Mrinalini; R, N	Higher risk of short term COVID-19 vaccine adverse events		No specific focus on specialists or the professions
PDNR4ST4	2011	Dey, Rosalind M; D	Collaboration in chronic care: Unpacki	Int. J. Pharm. Pract.	No specific focus on specialists or the professions
XRMGU8BL	2023	Dheensa, Sandi; M	Healthcare Professionals' Own Experie	Trauma, Violence, A	No specific focus on specialists or the professions
UU3SCDWT	2011	Dhillon, B S	Patient Safety: An Engineering Approach		No specific focus on specialists or the professions
MN2PKTXL	2023	Dhungel, Binod; Sh	Use of antimicrobials during the COVID	PLOS Glob. Public H	No specific focus on specialists or the professions
N5TVDTRZ	2023	Diaz-Castro, Lina;	Mental health needs and accessing spe	BJPSYCH OPEN	No info on specialists' contribution to health systems/population health, or their governance
27N9H2C3	2009	Diaz-Ledezma, Cla	Factors associated with variability in le	Spine J.	No specific focus on specialists or the professions
PMKFZ4SI	2017	Dickenson, Donna	Property in the body: Feminist perspectives, second edition		No specific focus on specialists or the professions
8YAJ4NGF	2014	Dickson, Kim E; Sin	Every Newborn: Health-systems bottle	Lancet	No specific focus on specialists or the professions
B27RK3SH	2023	Dietrich, Janan J; Jo	Examining the Relationship Between Ps	AIDS Behav.	No specific focus on specialists or the professions
TGIWM5D4	2024	Dingel, Julius; Klein	Predictors of Health Care Practitioners	J. Med. Internet Res	No info on specialists' contribution to health systems/population health, or their governance
T7V5L7DJ	2022	Dingle, Arden D; Ch	Considerations for rural training programs		Not on LMICs
4WDM5FWS	2023	Diniz-Freitas, Márc	World Workshop on Oral Medicine VIII:	Oral Surg. Oral Med	No info on specialists' contribution to health systems/population health, or their governance
USYFBSQZ	2016	Dittrich, Christian;	ESMO/ASCO Recommendations for a G	ESMO Open	No info on specialists' contribution to health systems/population health, or their governance
Y94CJWLQ	2021	Dobry, Allison; Beg	Implementation and Impact of a Store-a	Telemed. e-Health	No info on specialists' contribution to health systems/population health, or their governance
4QSXC4C8	2023	Doetsch, Julia Nad	2008 economic crisis impact on perina	J. Epidemiol. Comm	No specific focus on specialists or the professions
3FFVKYGE	2023	Doherty, Tanya; Fa	Experiences of public-private contracti	PLOS Glob. Public H	No specific focus on specialists or the professions
QETQZ67D	2023	Doherty, Tanya; So	Utilisation of private general practition	BMJ Open	No specific focus on specialists or the professions
M98X4DD2	2023	Dominguez, Teofilo	Situation of the training of surgical pers	Rev. Climatol.	No info on specialists' contribution to health systems/population health, or their governance
4FQTHHTB	2018	Dong, Birong; Yue,	Transformation of a Geriatric Departme	J. Am. Geriatr. Soc.	No info on specialists' contribution to health systems/population health, or their governance
UAI6KQRL	2022	Dong, Shengjie; Shi	The Application of Graph Theoretical An	JMIR Med. informat	No info on specialists' contribution to health systems/population health, or their governance
I377CMPI	2015	Dorj, Gereltuya; He	A questionnaire study of injections pres	PeerJ	No specific focus on specialists or the professions
JU5H3U6F	2012	Dorsch, Jerry A; Do	Understanding anesthesia equipment: Fifth edition		No info on specialists' contribution to health systems/population health, or their governance
XPXZGQB2	2022	Douglas, Pamela S;	2022 ACC Health Policy Statement on E	J. Am. Coll. Cardiol.	Not on LMICs
I35TQ2DE	2024	Douglass, Paul L; It	Achieving Equitable Cardiovascular Ca	JACC. Adv.	Not on LMICs
IAGH9V49	2019	Downe, Soo; Finlay	Provision and uptake of routine antenat	Cochrane Databas	No specific focus on specialists or the professions
KMILQWXX	2013	Doyal, Len Lesley L	Living with HIV and dying with AIDS: Diversity, inequality and		No specific focus on specialists or the professions
XT3ADC3R	2017	Dresser, C; Periyar	Management and Outcomes of Acute S	WORLD J. Surg.	
RYTKMKM3	2013	Drèze, Jean; Sen, A	An uncertain glory: India and its contradictions		No specific focus on specialists or the professions
C6B4BF3X	2008	Drife, James	Maternal mortality in well-resourced co	Best Pract. Res. Cli	Not on LMICs
VGYSW7N3	2013	Driouchi, Ahmed	Skilled human resources in the health sectors and impacts		No specific focus on specialists or the professions
6FIHNFCJ	2021	Drudi, Laura M; Nis	The impact of the COVID-19 pandemic	Semin. Vasc. Surg.	No info on specialists' contribution to health systems/population health, or their governance
YKD2RHP7	2024	Drysdale, Andrew T	New treatments: Opportunities and cha	Semin. Perinatol.	No specific focus on specialists or the professions
TFNZKNIT	2024	Du, Lanfang; Li, Yar	A national survey on current state and c	BMC Med. Educ.	
TIFN7SZE	2018	Dua, Anahita; Kopr	Factors that Predict Failure to Meet Me	Ann. Vasc. Surg.	Not on LMICs
WMNB5DKF	2013	Duane, Therese M;	Surgeons do not listen: Evaluation of co	Am. Surg.	Not on LMICs
BWNIFS9N	2022	Duarte, Cristiane S	The Child Mental Health Treatment Gap	Psychiatr. Serv.	No info on specialists' contribution to health systems/population health, or their governance
8BV3AWDH	2023	Dubey, Sweta; Des	Evolution of Government-funded health	Lancet Reg. Heal. S	No specific focus on specialists or the professions
TTXYYXXH	2019	Duggan, Ashley P	Health and illness in close relationships		No specific focus on specialists or the professions
SNAZMBMN	2014	Dukes, Graham; Br	Pharmaceuticals, corporate crime and public health		No specific focus on specialists or the professions
AHK2QYUH	2023	Dumitrascu, Dan L	Update on the Role of Rifaximin in Dige	J. Gastrointestin. Li	No specific focus on specialists or the professions
RXYN8M52	2015	Dunning, Lorna; Hs	Point-of-care HIV early infant diagnosis	J. Int. AIDS Soc.	No specific focus on specialists or the professions
IIRI9Y8W	2015	Duston, Paula Stan	Analyzing form, function, and financing of the U.S. health ca		Not on LMICs
GAP93EGK	2020	Dutfield, Graham	That high design of purest gold: A critical history of the pharm		No specific focus on specialists or the professions
7G64XV7W	2019	Dutta, Suchismita;	Molecular targets of aspirin and preven	Int. J. Mol. Sci.	No specific focus on specialists or the professions
32L4BDP9	2012	Dwamena, Frances	Interventions for providers to promote a	Cochrane Databas	No specific focus on specialists or the professions
RXF3LWAV	2023	Dweib, Mohammad	Diabetes-Related Microvascular Comp	J. Clin. Med.	No specific focus on specialists or the professions
TXIQYFQQ	2018	Dwyer, James G	Liberal Child Welfare Policy and its Destruction of Black Liv		No specific focus on specialists or the professions
ED6F7YIJ	2020	Dzobo, Kevin; Adot	Integrating Artificial and Human Intellig	OMICS	No specific focus on specialists or the professions
H8ED2IDQ	2018	Dzudie, Anastase;	Development of the certificate course in the management c		No info on specialists' contribution to health systems/population health, or their governance
TCJQ8TNN	2022	Eala, Michelle Ann	Geographic Distribution of Cancer Car	JCO Glob. Oncol.	No info on specialists' contribution to health systems/population health, or their governance
UJ23NI4D	2024	Ebeid, Mohamed; V	Effect of the timing of initial exposure to	Early Child. Res. Q.	No specific focus on specialists or the professions
SNCJSWXB	2019	Ebrahim, Ahmed H	A comparative analysis of medical touri	Tourism	No specific focus on specialists or the professions
6TPTFD8U	2009	Eckardt, Kai-Uwe;	Foreword	Am. J. Transplant.	No specific focus on specialists or the professions
Q5BMX5MW	2009	Eckardt, Kai-Uwe;	Abstract	Am. J. Transplant.	No specific focus on specialists or the professions
WR57T8LI	2020	Edelman, Alexandr	Health Service Delivery In Norther Aust	Rural Remote Heal	Not on LMICs
QVBT7KDV	2014	Eden, Jill; Berwick,	Graduate medical education that meets the nation's health		Not on LMICs
HBFSWS2G	2020	Ehrlich, Bella S; Mc	Barriers to the early integration of pallia	Cancer	No info on specialists' contribution to health systems/population health, or their governance
NFKQBJ6M	2015	Eichbaum, Quentin	New Medical Schools in Africa: Challen	Ann. Glob. Heal.	Other
9UM454TF	2024	Eick, Frode; Vallers	Perinatal mortality among pregnant und	Soc. Sci. Med.	Not on LMICs
BZZF7LV6	2021	Ekawati, Fitriana M	Hypertensive disorders of pregnancy (H	BMC Pregnancy Ch	No specific focus on specialists or the professions
58PY3HTK	2015	Ekenze, S O; Ugwu,	Evaluation of neonatal circumcision tra	J. Pediatr. Urol.	No info on specialists' contribution to health systems/population health, or their governance
VID99I6M	2014	Ekeroma, Alec J; Ke	Building reproductive health research a	BMC Med. Educ.	No info on specialists' contribution to health systems/population health, or their governance
TPNKJGLN	2020	El Hayek, Samer; N	Telepsychiatry in the Arab World: A View	Neuropsychiatr. Di	No info on specialists' contribution to health systems/population health, or their governance
6FPM375I	2020	El-Begermy, M A; D	Save the hearing, an ear surgery trainin	Egypt. J. Otolaryngo	No info on specialists' contribution to health systems/population health, or their governance
H44LUDVD	2014	Elder, John P; Pequ	Caregiver behavior change for child sur	J. Health Commun.	No specific focus on specialists or the professions
AYG4D3WG	2019	Ellis, Lee; Farringto	Handbook of crime correlates		No specific focus on specialists or the professions
AXFTLSBY	2018	Ellis, Lee; Hoskin, A	Handbook of social status correlates		No specific focus on specialists or the professions
4RM3RFHQ	2013	Ellsworth, P; Marso	Bladder health across the life course	Int. J. Clin. Pract.	No specific focus on specialists or the professions
DEG5MJ2H	2008	Emejulu, J K C	Neurosurgery in Nigeria--an evaluation	Niger. J. Clin. Pract.	
PDWMTQMM	2023	Emmelkamp, Paul M	Mental health of refugees: Etiology and treatment		No specific focus on specialists or the professions
P42WYND2	2021	Emwodew, Deleleg	Knowledge and attitude toward evidenc	JMIR Med. Educ.	No specific focus on specialists or the professions
9B5BIDPB	2021	Endo, Justin; Peebl	Geriatric Dermatology		Not on LMICs
86P5MDFJ	2009	England, Mary Jane	Depression in parents, parenting, and children: Opportuniti		No info on specialists' contribution to health systems/population health, or their governance
3CJNFGP3	2020	English, M; Stracha	The paediatrician workforce and its role	Arch. Dis. Child.	
MD9IIKCG	2024	English, Mike; Rispe	Breaking the silence on first referral hos	Lancet Glob. Heal.	
7L4GP9ZJ	2013	Enright, A	Review article: Safety aspects of anest	Can. J. Anesth. Can	No info on specialists' contribution to health systems/population health, or their governance
625GIJIV	2018	Epiu, Isabella; Alia,	Estimating the cost and cost-effectiven	Health Policy Plan.	No info on specialists' contribution to health systems/population health, or their governance
T2S878DS	2017	Epiu, Isabella; Tind	Challenges of Anesthesia in Low- and M	Anesth. Analg.	No info on specialists' contribution to health systems/population health, or their governance
5I5YWI5U	2018	Epstein, Danny; Pe	Pocket-size point-of-care ultrasound in	Travel Med. Infect.	No info on specialists' contribution to health systems/population health, or their governance
F9K89YX9	2023	Erchick, D J; Hazel,	Vulnerable newborn types: analysis of s	BJOG An Int. J. Obst	No specific focus on specialists or the professions
K6MWFVL8	2020	Erem, Anna Sarah;	Gynecologic Oncology Sub-Specialty Tr	Front. public Heal.	
LRFDLZKL	2017	Eriksen, J G	Postgraduate Education in Radiation O	Clin. Oncol.	No info on specialists' contribution to health systems/population health, or their governance
BENKGD6R	2021	Eriksen, Nancy L; V	Preconceptual Health		No specific focus on specialists or the professions
CU3WZJDC	2021	Errani, Costantino;	Women and men in orthopaedics	SICOT-J	
28HV65XQ	2016	Ertel, P; Adalig, B; D	Understanding patient and physician pe	Int. J. Clin. Pract.	No info on specialists' contribution to health systems/population health, or their governance
3YPWDZDZ	2019	Erumeda, Neetha J	A self-assessment study of procedural s	African J. Prim. Hea	No specific focus on specialists or the professions
HWAWAUDZ	2018	Esala, Jennifer J; Vu	Collaborative care for refugees and tort	Traumatology (Talla	No specific focus on specialists or the professions
DY9SW7QB	2015	Escott-Stump, Sylv	Nutrition and diagnosis-related care		No specific focus on specialists or the professions
GW65L9HY	2013	Escott-Stump, Sylv	Nutrition and diagnosis-related care: Seventh edition		No info on specialists' contribution to health systems/population health, or their governance
GSEGA2VY	2024	Espinoza-Moya, Ma	Mapping inter-professional collaborati	Crit. Rev. Oncol. He	No info on specialists' contribution to health systems/population health, or their governance

Appendix 4

Table S4: List of papers excluded, with reason

AE2ZQAQD	2018	Essounga, Yvette N	Entrepreneurship and a healthcare syst	Transnatl. Corp. Re	No specific focus on specialists or the professions
CCSERNEW	2019	Essuman, A; Gold, I	Establishing the First Geriatric Medicin	J. Am. Geriatr. Soc.	
MF65EP26	2016	Esteban Montalvo-	The importance of master's degree and	Cir. Cir.	Not on LMICs
JFVUSK34	2017	Estenssoro, Elisa; A	Organizational Issues, Structure, and P	Crit. Care Med.	No info on specialists' contribution to health systems/population health, or their governance
QPWJ4TXI	2023	Evangelidou, Stella	Structural and Intercultural Factors to	J. Immigr. Refug. Stu	No specific focus on specialists or the professions
VI97X2GB	2004	Evans, David; Shea	Educating health professionals to impro	Paediatr. Respir. Re	No info on specialists' contribution to health systems/population health, or their governance
SGYXCA4U	2021	Evans, Laura; Rhod	Executive Summary: Surviving Sepsis C	Crit. Care Med.	No info on specialists' contribution to health systems/population health, or their governance
9MS9G9KG	2011	Everett, Margaret	They say it runs in the family: Diabetes a	Soc. Sci. Med.	No specific focus on specialists or the professions
6YUX37WS	2017	Ezekowitz, Justin A	2017 Comprehensive Update of the Ca	Can. J. Cardiol.	Not on LMICs
XUZP7BBK	2023	Fabbri, Leonardo M	COPD and multimorbidity: recognising	Lancet Respir. Med	No specific focus on specialists or the professions
EQDMDW7S	2021	Fabian, Ido Didi; St	Travel burden and clinical presentation	Br. J. Ophthalmol.	No specific focus on specialists or the professions
LHMB3CPD	2023	Fadzil, Malindawat	Cost volume profit analysis for full payi	PLoS One	No specific focus on specialists or the professions
8L265C9K	2015	Faguet, Guy	The conquest of cancer: A distant goal		No specific focus on specialists or the professions
U3ZLEDAB	2015	Faisal-Cury, Alexar	The relationship between socioeconom	Int. J. Equity Health	No specific focus on specialists or the professions
ZIQINBPF	2020	Falk, Ryan; Taylor, I	Surgical Task-Sharing to Non-specialist	World J. Surg.	
BUAPZPG9	2017	Fallah, Parisa Nico	Unifying a fragmented effort: a qualitati	Global. Health	Not on LMICs
7DZE9CHV	2022	Farahat, Taghreed	The paradigm shift from NAFLD to MAFL	Liver Int. Off. J. Int. A	No specific focus on specialists or the professions
ZJM6XM9J	2023	Farias, Terence; Ko	Guidelines from the Brazilian society of	Arch. Endocrinol. M	No info on specialists' contribution to health systems/population health, or their governance
Q4A72PNQ	2011	Farrell, Anne-Mare	Organ shortage: Ethics, law and pragmatism		No specific focus on specialists or the professions
J64GTTKL	2015	Farrell, Carole	Advanced nursing practice and nurse-led clinics in oncolog		No specific focus on specialists or the professions
8CGMJXQK	2017	Fatima, Iram; Hum	How do patients perceive and expect q	Oman Med. J.	Other
4IMYGI6Z	2018	Favier, Mary; Greer	Safe abortion in South Africa: "We have	Int. J. Gynecol. Obs	No info on specialists' contribution to health systems/population health, or their governance
WBFGT3TC	2019	Fawcus, Susan	Practical approaches to managing post	Best Pract. Res. Cli	No specific focus on specialists or the professions
XMH7IUIW	2024	Fazio, Linda S	Developing Occupation-Centered Programs With the Comm		No specific focus on specialists or the professions
S7FPEEYY	2009	Feder, G; Ramsay,	How far does screening women for dom	Health Technol. Ass	No info on specialists' contribution to health systems/population health, or their governance
5AKLV7X8	2024	Fehlings, Michael G	An Update of a Clinical Practice Guidel	Glob. spine J.	No info on specialists' contribution to health systems/population health, or their governance
M5P4MEQX	2013	Feldman-Winter, L	Evidence-based Interventions to Suppo	Pediatr. Clin. North	No specific focus on specialists or the professions
CUXDTLTI	2009	Feldman, Arthur M	Pursuing excellence in healthcare: Preserving america's ac		Not on LMICs
LQ2UAPKZ	2017	Fell, Deshayne B; A	Influenza epidemiology and immunizati	Vaccine	No specific focus on specialists or the professions
SDQKWPMP	2020	Fenton, Kathleen N	Global health initiatives in cardiothora	Asian Cardiovasc. T	No info on specialists' contribution to health systems/population health, or their governance
K8ZCX35F	2020	Ferber, Sarah; Mar	IVF and Assisted Reproduction: A Global History		No info on specialists' contribution to health systems/population health, or their governance
RRC7QBR5	2016	Ferguson, Pamela F	Inspiring a medico-legal revolution: Essays in honour of She		No specific focus on specialists or the professions
HR5U3NIU	2015	Ferguson, Pamela F	Inspiring a medico–legal revolution: Essays in honour of she		No specific focus on specialists or the professions
TCBQYT7T	2013	Fernandes, Rubens	Psychiatry career in Brazil: regional dis	Int. Rev. Psychiatry	No info on specialists' contribution to health systems/population health, or their governance
BFJVZSM4	2020	Fernandez Turienzo	Midwifery continuity of care versus star	PLoS Med.	Not on LMICs
DMHEXWIS	2020	Fernández-Hidalgo	Enterococcus faecalis endocarditis: W	Future Microbiol.	No specific focus on specialists or the professions
I2SY2R5E	2024	Ferreira, Fabio de C	Quality of emergency oncological surge	Rev. Assoc. Med. Br	No info on specialists' contribution to health systems/population health, or their governance
4CURXIX6	2017	Ferrell, Betty R; Ter	Integration of palliative care into stand	J. Clin. Oncol.	Not on LMICs
W8HGNIVH	2022	Fields, Emma C; Ka	Education in gynecological brachythera	Int. J. Gynecol. Can	No info on specialists' contribution to health systems/population health, or their governance
7QCNDANS	2021	Fiest, Kirsten M; Pa	Experiences and management of physic	BMC Psychiatry	No specific focus on specialists or the professions
6C2DBMAT	2024	Fiks, Eva	State Intimacies: Sterilization, Care and Reproductive Chro		No info on specialists' contribution to health systems/population health, or their governance
7ECLPM2Y	2016	Filby, Alex; McConv	What prevents quality midwifery care?	PLoS One	No specific focus on specialists or the professions
N8ZEKFHC	2019	Filipovic, Nenad	Computational modeling in bioengineering and bioinformat		No specific focus on specialists or the professions
XVIKB2JE	2007	Finch, Caleb E	The Biology of Human Longevity: Inflammation, Nutrition, ar		No specific focus on specialists or the professions
QY3QK6DX	2021	Finney, Robyn E; To	Second victim experiences of nurses in	J. Nurs. Manag.	No specific focus on specialists or the professions
QLAGIBRB	2022	Firdi, Nabila Puspit	Using Pectoral Muscle Removers in Ma	J. BIOMIMETICS Bio	No info on specialists' contribution to health systems/population health, or their governance
EK2LFDSH	2022	Firoz, Tabassum; Pi	Non-communicable diseases and mate	BMC Pregnancy Ch	No info on specialists' contribution to health systems/population health, or their governance
ZVGLLJQP	2017	Fisher, Leon; Fishe	Acid-Suppressive Therapy and Risk of In	Clin. Drug Investig.	No specific focus on specialists or the professions
7JL5823L	2021	Fite, Brett Z; Hinos	Increasing Diversity in Radiology and M	Mol. Imaging Biol.	No specific focus on specialists or the professions
8MH9ZK4L	2020	Fitts, Jessica J; Geg	Strengthening mental health services ir	Health Policy Plan.	
TPM8TPCC	2018	Fitzgerald, Elizabet	A pediatric death audit in a large referra	BMC Pediatr.	No specific focus on specialists or the professions
8UMPQPUJ	2023	Fitzgerald, Tamara	Gaps and priorities in innovation for chi	Semin. Pediatr. Sur	No info on specialists' contribution to health systems/population health, or their governance
EM5NC4G8	2012	Fitzgibbon, Dermot	Cancer pain: Assessment, diagnosis, and management		No info on specialists' contribution to health systems/population health, or their governance
JSY8VCTB	2019	Fitzmaurice, Chris	Global, regional, and national cancer ir	JAMA Oncol.	No specific focus on specialists or the professions
GDVNI7H5	2017	Fitzmaurice, Chris	Global, Regional, and National Cancer	JAMA Oncol.	No specific focus on specialists or the professions
TK89DTDU	2017	Fleming, Matthew;	Surgical referral coordination from a fir	BMC Health Serv. R	Other
CSERFKG2	2015	Flodgren, Gerd; Ra	Interactive telemedicine: Effects on pro	Cochrane Databas	No info on specialists' contribution to health systems/population health, or their governance
F6EFLJYT	2016	Flores, J R; Rivera,	Assessment of the Chilean rural practit	Rev. Med. Chil.	No specific focus on specialists or the professions
VUANRPI8	2022	Folch, Erik E; Bowli	NAVIGATE 24-Month Results: Electroma	J. Thorac. Oncol.	No info on specialists' contribution to health systems/population health, or their governance
9GRFKT9M	2018	Foley, Paul Bernard	Encephalitis lethargica: The mind and brain virus		No specific focus on specialists or the professions
IW97E8UU	2024	Folland, Sherman;	The Economics of Health and Health Care, Ninth Edition		No specific focus on specialists or the professions
L648M63S	2023	Fong, Khi Yung; Lai,	Clinical validation of a smartphone app	Int. Wound J.	No specific focus on specialists or the professions
5HW58YZC	2020	Fontanella, Marco	Neurosurgical activity during COVID-19	J. Neurosurg. Sci.	No info on specialists' contribution to health systems/population health, or their governance
QLL52QRI	2018	Footman, Katharin	Medical Abortion Provision by Pharmac	Stud. Fam. Plann.	No specific focus on specialists or the professions
VYY2FL9C	2016	Forouzanfar, M H; A	Global, regional, and national compara	Lancet	No specific focus on specialists or the professions
LMP2J7CB	2021	Forsetlund, Louise;	Continuing education meetings and wo	Cochrane Databas	No specific focus on specialists or the professions
ZRN7DAPE	2010	Foster, Jennifer; Bu	A community-based participatory resea	Midwifery	No info on specialists' contribution to health systems/population health, or their governance
EW6FHGKT	2021	Fountoulakis, Kons	Psychiatry: From Its Historical and Philosophical Roots to th		Not on LMICs
TRG2J3VE	2024	Fountoulakis, Kons	Somatic multicomorbidity and disabilit	CNS Spectr.	No specific focus on specialists or the professions
HM5HAHA3	2022	Fountoulakis, Kons	The effect of different degrees of lockdo	Psychiatry Res.	No specific focus on specialists or the professions
MYVNYPQ2	2011	Fovargue, Sara	Xenotransplantation and risk: Regulating a developing biote		No specific focus on specialists or the professions
XWRDLQ3V	2022	Fowler, Zachary; D	Academic Output in Global Surgery afte	World J. Surg.	
W8R49HBQ	2018	Fraccaro, Paolo; Vi	The influence of patient portals on user	Int. J. Med. Inform.	No specific focus on specialists or the professions
AWR6TWJU	2023	Franco, Helena; Sir	Scaling Surgical Resources: A Capacity	World J. Surg.	No info on specialists' contribution to health systems/population health, or their governance
UWSDRGZQ	2019	Frankel, Lorry R; Hs	Criteria for Critical Care Infants and Ch	Pediatr. Crit. Care I	No info on specialists' contribution to health systems/population health, or their governance
32R4FXH2	2018	Franzblau, Lauren I	Identifying barriers to the care of the rh	Int. J. Rheum. Dis.	Other
KUSS4NGJ	2018	Fraser, B A; Powell,	Palliative care development in Africa: L	J. Glob. Oncol.	No info on specialists' contribution to health systems/population health, or their governance
BP8YMS2I	2020	Frattini, Silvia; Mad	Coronavirus disease 2019 and cardiova	J. Cardiovasc. Med.	No info on specialists' contribution to health systems/population health, or their governance
4RSHPGNW	2024	Frie, Lonneke S; de	How workers meet new expertise needs	Int. J. Manag. Rev.	No specific focus on specialists or the professions
MZ5B6SGZ	2023	Friedma, Mark T; W	Immunohematology, Transfusion Medicine, Hemostasis, an		No info on specialists' contribution to health systems/population health, or their governance
WJCVRIK6	2024	Frontera, Walter; S	Low birth weight babies and disability		No specific focus on specialists or the professions
JRH6FIX7	2018	Fuchs, Victor R	Health economics and policy: Selected writings By Victor F		No specific focus on specialists or the professions
HWMYNFZ3	2018	Fuentes-Alabi, Soa	The cost and cost-effectiveness of child	Cancer	No specific focus on specialists or the professions
MIKVQANR	2022	Fugazzola, Paola; C	The WSES/SICG/ACOI/SICUT/AcEMC/S	World J. Emerg. Sur	No specific focus on specialists or the professions
6SDYQ3TU	2019	Furst, Branko	The heart and circulation: An integrative model		No specific focus on specialists or the professions
MJVYB4KG	2016	Fusheini, Adam; Ey	Achieving universal health coverage in	BMC Health Serv. R	No specific focus on specialists or the professions
FQTKJ5XK	2014	Gage-Bouchard, El	Factors influencing patient pathways fo	PLoS One	No specific focus on specialists or the professions
9LM2BGQ3	2022	Gaggiano, Carla; Vi	The Autoinflammatory Diseases Allianc	Front. Med.	No specific focus on specialists or the professions
7LWHDWX5	2019	Gajewski, J; Borgst	Evaluation of a surgical training progran	Br. J. Surg.	No info on specialists' contribution to health systems/population health, or their governance
TRIUPFN8	2019	Gajewski, J; Borgst	Evaluation of a surgical training progran	Br. J. Surg.	No info on specialists' contribution to health systems/population health, or their governance
9YGP7RV8	2018	Gajewski, Jakub; Bi	Global Surgery - Informing National Strategies for Scaling Up Surgery in Sub-Saharan Africa.		
3QUUS4F3	2020	Gajewski, Jakub; M	Supervision as a tool for building surgica	Hum. Resour. Health	
96AIISPI	2022	Gajewski, Jakub; W	Why Do They Leave? Challenges to Rete	Int. J. Heal. Policy M	No info on specialists' contribution to health systems/population health, or their governance
XR5PNP5D	2020	Galagali, Preeti M;	Psychological care in low-resource sett	Clin. Child Psychol.	No specific focus on specialists or the professions
KHHDPFDS	2021	Galiatsatos, Panag	Comparing Critical Care Admissions Ar	Heal. Secur.	No info on specialists' contribution to health systems/population health, or their governance
36NEQLFS	2013	Galukande, Moses;	Use of surgical task shifting to scale up	BMC Health Serv. R	No specific focus on specialists or the professions
HXTYVUYQ	2024	Galvin, Annalynn M	Homelessness in the Perinatal Period a	Public Health Rep.	No specific focus on specialists or the professions
R9395QDM	2020	Gambir, Katherine;	Self-administered versus provider-adm	Cochrane Databas	No specific focus on specialists or the professions
WUHL3F7U	2012	Gang-xi, Lin; Yi-mir	Personal experience in pediatric emerg	Chin. Med. J. (Engl)	No info on specialists' contribution to health systems/population health, or their governance

Appendix 4

Table S4: List of papers excluded, with reason

XM3TAEFQ	2016	Ganle, John Kuumu	Addressing health system barriers to ac	Int. J. Health Plann.	No specific focus on specialists or the professions
GSNLWVM7	2021	García-Goñi, Manu	Mental illness inequalities by multimor	Int. J. Environ. Res.	No specific focus on specialists or the professions
DXEGH7TT	2004	García-Silberman,	Research of epidemiological and psych	SALUD Ment.	No specific focus on specialists or the professions
BJB8YX92	2018	Garcia, Lynne S; Ar	Laboratory diagnosis of parasites from t	Clin. Microbiol. Rev	No specific focus on specialists or the professions
J3GMQEJX	2023	Gathuya, Z; Nabuk	Children's Anaesthesia and perioperati	Semin. Pediatr. Sur	No info on specialists' contribution to health systems/population health, or their governance
GBXT4GLW	2018	Gatwiri, Kathomi	African womanhood and incontinent bodies: Kenyan women		No specific focus on specialists or the professions
2K42EVQX	2018	Gauld, Robin; Asga	Reshaping public hospitals: an agenda	BMJ Glob. Heal.	No specific focus on specialists or the professions
YE7RJ3MH	2019	Gautam, Bishnu; S	Employment preferences of obstetricia	Hum. Resour. Health	
SU7SIFJQ	2008	Gavazza, Cláudia Z	[Use of rehabilitation services by techn	Cad. Saude Publica	No specific focus on specialists or the professions
SRIHBJF4	2024	Gbobaniyi, Olabod	The strategic efficacy of artificial intelligence (AI) in medica		No specific focus on specialists or the professions
J4V5QZ3F	2020	Gehenne, Lucie; Le	Testing two competitive models of emp	Eur. J. Cancer Care	No specific focus on specialists or the professions
DWW54I5F	2023	Gellert, George A;	The Role of Virtual Triage in Improving C	Telemed. Reports	No specific focus on specialists or the professions
AVSWVZ4G	2020	Gemechu, Kasiye S	Prevalence of hypertensive disorders o	Women's Heal.	No specific focus on specialists or the professions
4T8DEV3F	2019	George, A; Blaauw,	Doctor retention and distribution in po	Hum. Resour. Healt	No specific focus on specialists or the professions
3FAFHR2B	2023	Ghebrehiwet, Sena	Building a postgraduate psychiatry trai	Front. public Heal.	No info on specialists' contribution to health systems/population health, or their governance
Y2N35D5K	2020	Gheorghe, Gina; To	Cardiovascular risk and statin therapy	Diagnostics	No specific focus on specialists or the professions
MLWTEA7U	2021	Ghosh, Jay; Papado	Methods for managing miscarriage: a ne	Cochrane Databas	No specific focus on specialists or the professions
5KAP2LHM	2020	Giavina-Bianchi, M	Teledermatology reduces dermatology	eClinicalMedicine	
DB65BDSH	2006	Gibbon, Sahra	Breast cancer genes and the gendering of knowledge: Scien		No info on specialists' contribution to health systems/population health, or their governance
8A9IEWAY	2021	Giesel, Louise Mar	Clinical prediction rules for the diagno	BMC Infect. Dis.	No specific focus on specialists or the professions
5QKLAW5K	2018	Gile, Philipos Petro	The effect of human resource managem	Hum. Resour. Healt	No specific focus on specialists or the professions
2RHWY8TM	2023	Ginsburg, Ophira;	Women, power, and cancer: a Lancet C	Lancet	No specific focus on specialists or the professions
SGGDZKH5	2023	Giordano, Simona	Children and Gender: Ethical Issues in Clinical Managemen		No specific focus on specialists or the professions
P9J7LZW4	2021	Giuliani, Meredith;	Motivations for and Challenges in the D	Acad. Med.	
L64PHLBR	2023	Giunta, Diego Herr	Development and validation of nonatte	Int. J. Health Plann.	No specific focus on specialists or the professions
7PYXSA7J	2016	Glasser, Saralee; H	Rate, risk factors and assessment of a c	J. Adv. Nurs.	No specific focus on specialists or the professions
ABBCKERH	2014	Gleit, Rebecca; Fr	Transition planning: Teaching sexual se	Contemp. Pediatr.	No specific focus on specialists or the professions
45C28F37	2024	Glenton, Claire; Pa	Healthcare workers' informal uses of m	Cochrane Databas	No specific focus on specialists or the professions
R4UW9EPK	2017	Glenton, Claire; Sc	Implementation considerations when e	BMC Public Health	No specific focus on specialists or the professions
AXSAG63Q	2019	Goga, A; Slogrove,	The impact of health programmes to pr	S. Afr. Med. J.	No specific focus on specialists or the professions
8QE4X7ZA	2024	Goh, Amy H; Altma	Communication Between Pregnant Pe	J. Midwifery Wome	Not on LMICs
NMS4X299	2017	Goh, Su-Yen; Huss	Review of insulin-associated hypoglyce	J. Diabetes Investi	No specific focus on specialists or the professions
FHL4L72L	2012	Golden, Sherita Hi	Health disparities in endocrine disorde	J. Clin. Endocrinol.	No specific focus on specialists or the professions
VP54EAEE	2011	Goldman, Ran D; C	An international fellowship training pro	Pediatr. Emerg. Ca	No info on specialists' contribution to health systems/population health, or their governance
87WD8TGG	2017	Goldstein, Andrew	Evidence appraisal: A scoping review, c	J. Am. Med. Informa	No specific focus on specialists or the professions
Q8SF63DX	2013	Golightly, Larry K; T	Renal pharmacotherapy: Dosage adjustment of medication		No specific focus on specialists or the professions
E4XM2EB3	2021	Golightly, Larry K; T	Renal pharmacotherapy: Dosage adjustment of medication		No specific focus on specialists or the professions
7B4SB3PZ	2020	Gonçalves-Bradley	Mobile technologies to support healtho	Cochrane Databas	No specific focus on specialists or the professions
Y72DCW46	2021	Goncalves, Maria I	Dynamic evaluation of swallowing in pe	Anat. Rec. Integr. A	No specific focus on specialists or the professions
6TPU28QR	2022	Gondwe, Mtisunge	Resource availability and barriers to de	PLOS Glob. Public H	No specific focus on specialists or the professions
582LTQZ3	2019	Gong, Enying; Gu,	System-integrated technology-enabled	Am. Heart J.	No specific focus on specialists or the professions
MSSYVJEH	2014	Gong, Wenjie; Xu,	Village doctor-assisted case managem	Implement. Sci.	No specific focus on specialists or the professions
T56SRY89	2021	Gong, Xun; Zhang,	Successful Implementation and Develo	Front. public Heal.	No specific focus on specialists or the professions
FKA26CGP	2016	Gong, Yanjun; Huo	A survey of national cardiology workfor	Eur. Hear. J. Suppl.	
LIZ7UX2E	2010	Goodfield, Mark J D	The 'Connective Tissue Diseases'		Other
R3M2Z5F8	2019	Goranitis, Ilias; Lis	Antibiotic prophylaxis in the surgical m	Lancet. Glob. Heal.	No specific focus on specialists or the professions
5JM9AJA7	2020	Gordon, C; Gordon	Handmaidens and pioneers: Three fema	S. Afr. Med. J.	No info on specialists' contribution to health systems/population health, or their governance
J4DY3XIU	2010	Gore, Richard M; L	High-Yield Imaging: Gastrointestinal: A volume in High Yield		Other
IVLSHMQW	2003	Gorn, S B; Icaza, M	Mental health services:: Twenty five yea	SALUD Ment.	No specific focus on specialists or the professions
Y6PVWD2V	2021	Gorski, Lisa A; Had	Infusion Therapy Standards of Practice,	J. Infus. Nurs.	No specific focus on specialists or the professions
X4AXK89S	2013	Goss, Paul E; Lee,	Planning cancer control in Latin Americ	Lancet Oncol.	No specific focus on specialists or the professions
IXYT8AUC	2011	Grady, K; Ameh, C;	Improving essential obstetric and newb	J. Obstet. Gynaeco	No specific focus on specialists or the professions
I7K6BKHJ	2017	Graudal, Niels Albe	Effects of low sodium diet versus high s	Cochrane Databas	No specific focus on specialists or the professions
ND94H6PY	2020	Graudal, Niels Albe	Effects of low sodium diet versus high s	Cochrane Databas	No specific focus on specialists or the professions
UEAWR8KA	2011	Greaves, Peter	Histopathology of Preclinical Toxicity Studies: Interpretatio		Other
FQCIZV5J	2022	Greenberg, Mara B	Society for Maternal-Fetal Medicine Co	Am. J. Obstet. Gyne	Not on LMICs
WWQNBYQ7	2021	Greene, M Claire;	Leveraging Systems Science to Promote	Harv. Rev. Psychiat	No specific focus on specialists or the professions
P3G2LR3D	2008	Greenhalgh, Trisha	Primary Health Care: Theory and Practice		Other
5V9ZQNFA	2021	Greenhawt, Matthe	The Risk of Allergic Reaction to SARS-C	J. Allergy Clin. Immu	No specific focus on specialists or the professions
TUV28HSH	2023	Greenhawt, Matthe	Updated guidance regarding the risk of a	J. Allergy Clin. Immu	No specific focus on specialists or the professions
PB9G5DAJ	2015	Greenson, Joel K; L	Diagnostic Pathology: Gastrointestinal		Other
VIRANM4B	2015	Greer, Raquel C; A	Specialist and primary care physicians'	BMC Nephrol.	Not on LMICs
DAQTFN8R	2018	Greydanus, Donald	Medical history: Some perspectives		Other
FWG3MIDX	2011	Greysen, S Ryan; D	Medical education in sub-Saharan Afric	Med. Educ.	No specific focus on specialists or the professions
UIAH4KP6	2013	Grochowski, Janet	Families and health		Other
5VSH47KA	2023	Grover, Ashoo; Bha	Developing Standard Treatment Workfl	Front. public Heal.	
N2BK7H2B	2019	Grundy, Scott M; S	2018 AHA/ACC/AACVPR/AAPA/ABC/AC	J. Am. Coll. Cardiol.	No specific focus on specialists or the professions
WW4TNDHZ	2008	Grunwald, Martin	Human haptic perception: Basics and applications		Other
PJJ9MISR	2013	Gruskin, Sofia; Mil	History, principles and practice of health and human rights		Other
7WVGCUX7	2020	Gryschek, Guilherr	Examining the effect of non-specialised	BMJ Open	No specific focus on specialists or the professions
LLJ6SE79	2023	Gu, Mengyue; Zhen	Would you choose to be a psychiatrist a	Int. J. Ment. Health	Other
GXALMV5K	2023	Guerra, Luciane M	Perceptions of the oral health of their p	PLoS One	No info on specialists' contribution to health systems/population health, or their governance
K9B84M6Z	2017	Guest, Glenn D; Sc	Surgical capacity building in Timor-Lest	ANZ J. Surg.	
PZIP78ZI	2017	Gundugurti, Prasad	Effects of oral versus long-acting antips	Asian J. Psychiatr.	Other
RRAA7GS6	2014	Gunn, John; Taylor,	Forensic Psychiatry: Clinical, legal and ethical issues, Seco		Other
9NPID3B3	2013	Guntzviller, Lisa M	Increasing medication adherence in LEP (low-english profic		Other
KLF8XIXZ	2024	Gurakar, Ahmet; C	An international, multicenter, survey-b	Liver Transplant. Of	No specific focus on specialists or the professions
9TWCL77U	2023	Gurney, Tiana; O'S	Understanding the non-professional ne	Int. J. Health Plann.	Not on LMICs
MZXARK27	2015	Guthrie, Susan; Bie	The impact of the national institute for	Health Technol. Ass	Not on LMICs
X3UFDR3Z	2019	Gyselaers, Wilfried	Mobile health applications for prenatal	Curr. Pharm. Des.	No specific focus on specialists or the professions
XI9RU93Z	2020	Haagsma, Juanita A	Burden of injury along the development	Inj. Prev. J. Int. Soc.	No specific focus on specialists or the professions
XNBLDAL4	2015	Haastrup, Oluwato	Are we monitoring the quality of catara	Middle East Afr. J. Ophthalmol.	
CKJHGHUH	2022	Haeusermann, Tob	Of care, cure and the in-between: COVI	Int. J. Care Caring	Not on LMICs
TU9I7TG9	2015	Hafferty, Frederic	References		Other
HF8MQWPP	2013	Hagander, Lars E;	Surgeon Migration Between Developing	WORLD J. Surg.	
P2AFDKNJ	2012	Hakro, Ahmed Naw	Water, sanitation and poverty linkages	Asian J. Water, Env	No specific focus on specialists or the professions
2WKFU24H	2022	Haldar, Shefali; St	Collaboration Challenges and Technolo	Proc. ACM Human-	No specific focus on specialists or the professions
F8H3LYLY	2015	Hale, Matthew; Ra	List of publications on the economic an	Econ. Hist. Rev.	Not on LMICs
QUAHXNUV	2018	Hale, Thomas W	Hale's Medications and Mothers' MilkTM: 2019 A Manual of		Other
PMW56DL5	2020	Hales, Pippa; Whit	A case study of a collaborative allied he	J. Interprof. Care	No specific focus on specialists or the professions
NAS9ATEJ	2017	Haley, Connie A; V	Impact of a critical health workforce sh	Health Policy Plan.	
8FJ4CFFT	2017	Halili, Servando 'B	Addressing health workforce inequities	Med. Teach.	No specific focus on specialists or the professions
JXJ5BX3S	2017	Hamdani, Syed Us	Problem Management Plus (PM+) in the	Int. J. Ment. Health	No specific focus on specialists or the professions
E3RY6B8F	2020	Hameed, Mohajer;	Psychological therapies for women wh	Cochrane Databas	No specific focus on specialists or the professions
CR43QPYJ	2022	Hameed, Susan Ta	Using Phone Calls to Promote Commun	Iraqi J. Pharm. Sci.	No specific focus on specialists or the professions
CDW597ZB	2024	Hameed, Waqas; K	Optimising Perinatal Mental Health: Im	Ment. Heal. Prev.	Other
ML4EHIXV	2010	Hamilton, D Kirk; S	Design for Critical Care: An Evidence-Based Approach		Other
C7CX875S	2023	Hammad, Nazik; N	Competency-Based Workforce Develo	Curr. Oncol.	Not on LMICs
DVQ2RPA7	2014	Hammerstedt, Hea	Addressing WHO resolution 60.22: A pi	Ann. Emerg. Med.	

Appendix 4

Table S4: List of papers excluded, with reason

3KCNEZIE	2024	Han, Fushi; Chen, S	Single-cell transcriptomic sequencing	J. Transl. Med.	No specific focus on specialists or the professions
72MZIAWX	2014	Hanlon, Charlotte;	Challenges and opportunities for imple	PLoS One	Other
3MRN6MUF	2022	Hannah, Wesley; B	Global burden of early pregnancy gesta	Acta Diabetol.	No specific focus on specialists or the professions
2KHYKMU2	2016	Hannon, Ruth A; Po	Porth pathophysiology: Concepts of altered health states: S		Other
INQFYUSY	2024	Haoucha, Malika	Global Insights on Women Empowerment and Leadership		Other
MPUPLEVD	2020	Haque, Mainul; Isla	Strengthening primary health-care serv	Risk Manag. Health	No specific focus on specialists or the professions
WSK3P24K	2016	Harder, Henry G; W	Mental illness in the workplace: Psychological disability ma		Other
GTYVUE26	2013	Harper, Joyce C; G	Current issues in medically assisted re	Eur. J. Hum. Genet.	Not on LMICs
BPPZLI48	2021	Harris, Bronwyn; A	Mobile consulting as an option for deliv	Digit. Heal.	No specific focus on specialists or the professions
Y4BXGQDP	2023	Harris, Matthew	DECOLONIZING HEALTHCARE INNOVATION: Low-Cost Solu		Other
BXH5N553	2022	Harris, Meredith G	Perceived helpfulness of service sector	Int. J. Ment. Health	Not on LMICs
EVLHLR3C	2017	Harris, Stewart B;	Call to action: A new path for improving	Diabetes Res. Clin.	No specific focus on specialists or the professions
GTK4V38G	2015	Harrison, Mark	A global perspective: Reframing the hist	Bull. Hist. Med.	No specific focus on specialists or the professions
RI7J6NSQ	1999	Harrold, Leslie R.;	Knowledge, patterns of care, and outco	Journal of General I	Not on LMICs
R4WBPIQ6	2006	Hart, Julian Tudor	The political economy of health care: A clinical perspective		Other
WPW5YLPM	2022	Hart, Valerie; Tosi,	Oral Health of the Palliative and Hospice Patient		Other
C9AIJLUG	2022	Hashem, Ferhana;	What are the impacts of setting up new	BMC Med. Educ.	Not on LMICs
28MA6HJT	2023	Hashemi, Fallah; H	A comprehensive health effects assess	Environ. Sci. Pollut.	No specific focus on specialists or the professions
CD7FYFWF	2023	Hashemi, Saied; Ka	Identifying structure, process and outc	Iran. J. Nurs. Midwi	No specific focus on specialists or the professions
KT2V7M35	2011	Hashimoto, Hideki	Cost containment and quality of care in	Lancet	Not on LMICs
NG55AB76	2013	Hashmi, Mubashira	Growing burden of stroke in Pakistan: a	Int. J. stroke Off. J. I	No specific focus on specialists or the professions
QE3BV4T3	2019	Hassan, Ibrahim Fa	The inaugural Qatar Critical Care Conference with its Qata		Other
86NYVGCA	2024	Hassan, Viana; Alb	Impact of AI and robotics on the medical tourism industry		Other
UMDDCVT4	2019	Hazen, Helen; Anth	An introduction to the geography of health: Second edition		Other
3IWGUGWG	2020	He, Fan; Chen, Siji	Training status of child and adolescent	Eur. CHILD & Adolesc. PSYCHIATRY	
UJG8KU8X	2023	He, Jiajia; Kang, De	Label-free detection of invasive microp	J. Biophotonics	No specific focus on specialists or the professions
7V9YIHP6	2011	Healy, Geraldine;	Diversity, ethnicity, migration and work: International persp		Other
H2FBQFCA	2016	Healy, Judith	Improving health care safety and quality: Reluctant regulat		Not on LMICs
KTN9P9BA	2009	Healy, Judith; McK	Accessing Healthcare: Responding to Diversity		Other
FMAXMQRP	2022	Heaney, Suzanne;	Termination of pregnancy for fetal anon	BMC Pregnancy Ch	No specific focus on specialists or the professions
TE42VHHL	2023	Hedstrom, Anna; N	Outborn newborns drive birth asphyxia	PLOS Glob. Public H	No specific focus on specialists or the professions
K6A7TKP3	2024	Heidt, Jon; Wheeld	Visions of cannabis control		Other
7DRTYVU2	2020	Heinrich, Megan L;	Antibodies from Sierra Leonean and Nig	Sci. Rep.	No specific focus on specialists or the professions
GUKBGPUU	2018	Heist, Brian S; Tor	Medical migration: A qualitative explora	Med. Teach.	No specific focus on specialists or the professions
IG8PYDT7	2008	Helander, Einar A	Children and violence: The world of the defenceless		Other
HJ8NZATE	2020	Held, Michael F G;	Topics, Skills, and Cases for an Undergr	J. Bone Joint Surg. Am.	
SA82AD5K	2019	Heller, David J; Kur	Assessment of Barriers and Facilitators	JAMA Netw. Open	No specific focus on specialists or the professions
38JVL3ML	2023	Hempel, Susanne;	Care coordination across healthcare sy	BMJ Open	No specific focus on specialists or the professions
75WQNIIY	2021	Hendriks, Hans; Ad	The contribution of family physicians to	African J. Prim. Hea	No specific focus on specialists or the professions
4T9EGZSS	2018	Henningsen, Peter;	Management of Functional Somatic Syn	Psychother. Psycho	No specific focus on specialists or the professions
KKHG9QNE	2015	Henry, Jaymie Ang;	Essential surgery: the way forward.	World J. Surg.	
ZX9L777D	2015	Henry, Jaymie Ang;	Surgical and anaesthetic capacity of ho	Health Policy Plan.	
I6LIS27E	2015	Henry, Jaymie Ang;	Surgical and anaesthetic capacity of ho	Health Policy and Planning	
3PXJ76UB	2023	Hernandez, Moises	Racial and ethnic variation in referral t	J. Thorac. Cardiova	No specific focus on specialists or the professions
KKRHGKUR	2020	Hershman, Dawn L	Randomized Trial of Text Messaging to R	J. Clin. Oncol. Off. J	No specific focus on specialists or the professions
MTWFDINP	2007	Hey, Edmund	Neonatal Formulary: Drug Use in Pregnancy and the First Ye		Other
IUSGXECU	2011	Hey, Edmund	Neonatal Formulary 6: Drug Use in Pregnancy and the First Y		Other
3KPY2W98	2010	Heyman, Bob; Alas	Risk, Safety and Clinical Practice: Health care through the l		Other
YEPNSRBY	2017	Hicks, David G; Les	Diagnostic Pathology: Breast		Other
V3ZD8PQI	2018	Hiemke, C; Bergem	Consensus Guidelines for Therapeutic	Pharmacopsychiatr	No specific focus on specialists or the professions
Q7EQIEBL	2021	Higginbotham, Eve	The impact of COVID-19 on the careers of women in acader		Other
DYXYPYLH	2016	Hildebrandt, Sabin	The anatomy of murder: Ethical transgressions and anatomi		Other
MVXVKMW5	2023	Hilker, Sidney; Mat	Shared features of successful tele-ICU	Heal. POLICY Techn	No specific focus on specialists or the professions
439PWTWB	2022	Hill, Colin; Deville,	Establishing a Deaf and American Sign L	Acad. Med.	Not on LMICs
5875SP4B	2015	Hill, Jenny; D'mello	Women's access and provider practice	PLoS Med.	No specific focus on specialists or the professions
353B35G5	2020	Hinman, R S; Allen,	Development of a core capability frame	Osteoarthr. Cartil.	No specific focus on specialists or the professions
86UCF82H	2021	Hlongwa, Phumzile	Interprofessional collaboration among	Hum. Resour. Healt	No specific focus on specialists or the professions
7V7FJTH4	2022	Ho, Cynthia M	BIOSIMILAR BIAS: A BARRIER TO ADDR	Denver Law Rev.	No specific focus on specialists or the professions
7HRXQZSZ	2017	Ho, L Y; Chan, C K;	Patient opinion of lower urinary tract sy	HONG KONG Med.	No specific focus on specialists or the professions
66L7EJLG	2022	Hobeanu, Cristina;	Risk of subsequent disabling or fatal str	Lancet. Neurol.	No specific focus on specialists or the professions
7I2IHELL	2023	Hod, Moshe; Divak	The femtech revolution—A new approa	Int. J. Gynecol. Obs	No specific focus on specialists or the professions
Y9BJFPST	2022	Hodkinson, Alexan	Associations of physician burnout with	BMJ	No specific focus on specialists or the professions
QAL5QIKH	2022	Hoffman, Richard	Implementing the Mediterranean Diet: Nutrition in Practice		Other
866WPMRT	2011	Hoffman, Steven J;	Assessing healthcare providers' knowle	Malar. J.	No specific focus on specialists or the professions
DNS93KLI	2016	Hoffman, Steven J;	Surveying the knowledge and practices	Am. J. Trop. Med. Hy	No specific focus on specialists or the professions
4JBIBJJX	2023	Hohenschurz-Schn	Recommendations for the developmen	BMJ	No specific focus on specialists or the professions
7SKG8JRF	2016	Hojat, Mohammad	Empathy in health professions education and patient care		Other
9BUKQRA3	2018	Holcombe, Sarah J	Medical society engagement in conten	Health Policy Plan.	
Z598X7QP	2014	Holloway, Robert G	Palliative and end-of-life care in stroke	Stroke	No specific focus on specialists or the professions
DPM75TXW	2014	Homer, Caroline S	The projected effect of scaling up midw	Lancet	No specific focus on specialists or the professions
CNF6ZGKB	2022	Honda, Mari; Inoue	Lessons learned from the history of pos	Hum. Resour. Healt	Not on LMICs
PDIVQZ4B	2022	Hoogland, Romy; H	Global Problem of Physician Dual Pract	Iran. J. Public Health	
LWWWN2YB	2022	Hoppmann, Richar	International consensus conference re	ultrasound J.	Not on LMICs
ECPIJS33	2016	Hoque, Muhamma	Factors influencing the recommendati	Afr. Health Sci.	No specific focus on specialists or the professions
2WLI66QU	2016	Horn, Brady P; Cra	The Economic Impact of Intensive Care	Popul. Health Mana	No specific focus on specialists or the professions
UJHVJM4Y	2019	Horne, David J; Ko	Xpert MTB/RIF and Xpert MTB/RIF ultra	Cochrane Databas	No specific focus on specialists or the professions
S73KFQ8P	2000	Horton, R	Development aid: manna or myth?	Lancet (London, En	No specific focus on specialists or the professions
7QHMZAXE	2019	Hossain, Puspita;	'Feminization' of physician workforce in	PLoS One	
797ZVDSQ	1999	Hotchkiss, D R; Go	Household health expenditures in Mor	Int. J. Health Plann.	No specific focus on specialists or the professions
S6ZI7YNW	2008	Hou, Xiang-Yu; Fitz	Introduction of emergency medicine in	EMA - Emerg. Med. Australas.	
F9VEUQDW	2020	Hovenga, Evelyn J S	Measuring Capacity To Care Using Nursing Data		Other
IT35ZBJ5	2004	Howard, Scott C; P	Establishment of a pediatric oncology p	JAMA	
WYZUU8QH	2014	Hoyler, Marguerite	Shortage of doctors, shortage of data: A	World J. Surg.	
3DSAMTBR	2013	Hsieh, Cheng-Yang	National survey of thrombolytic therapy	J. stroke Cerebrovasc. Dis. Off. J. Natl. Stroke Assoc.	
PJGAMNV3	2020	Hu, Huan; Li, Huan	Association of self-reported sleep durat	Hypertens. Res.	No specific focus on specialists or the professions
92JPG4IP	2024	Huang, Yijiao; Wan	COVID-19 vaccine updates for people u	Sci. China. Life Sci.	No specific focus on specialists or the professions
2PZRU9P2	2022	Hudgins, Sarah	Maternal health in low resource settings		Other
28G7JHT5	2021	Huertas-Zurriaga,	Motherhood and decision-making amon	Reprod. Health	No specific focus on specialists or the professions
AMIPB5D3	2024	Hughes, Phillip M;	Assessing the safety and efficacy of pre	Am. Psychol.	No specific focus on specialists or the professions
IJBWV7KB	2022	Hui, Chi Yan; Abdu	Mapping national information and com	J. Glob. Health	No specific focus on specialists or the professions
CYSTVJMQ	2018	Hui, David S; Azhar	Middle East respiratory syndrome coro	Lancet. Infect. Dis.	No specific focus on specialists or the professions
GYBVGW6A	2016	Hui, David; Mori, M	Referral criteria for outpatient specialt	Lancet Oncol.	Not on LMICs
CJ22RGAY	2020	Hui, Kenrie P Y; Ch	Tropism, replication competence, and i	Lancet. Respir. Med	No specific focus on specialists or the professions
HKXSG9K4	2007	Hui, Zhang; Jian-Sh	An analysis of the current status of hosp	Am. J. Infect. Contr	No specific focus on specialists or the professions
5CGGNRE7	2010	Hummers-Pradier,	Series: The research agenda for genera	Eur. J. Gen. Pract.	Not on LMICs
6YPMG7MH	2020	Hung, Yuen W; Ho	Using routine health information data f	BMC Health Serv. R	No specific focus on specialists or the professions
5XCWR9MD	2022	Hunter, Benjamin M	Decentred regulation: The case of priva	World Dev.	No specific focus on specialists or the professions
JNEJLKUN	2012	Hurlimann, T; Sten	Inclusion and exclusion in nutrigenetics	J. Nutrigenet. Nutri	No specific focus on specialists or the professions
W5UU54DQ	2023	Huse, Oliver; Back	A comparative analysis of the cost-utili	Lancet Reg. Heal. W	No specific focus on specialists or the professions

Appendix 4

Table S4: List of papers excluded, with reason

BIM7M82T	2013	Hyde, Michael J; H	After the genome: A language for our biotechnological future		Other
I5YMJ2GS	2013	Hyder, Omar; Dods	Referral patterns and treatment choice	J. Am. Coll. Surg.	Not on LMICs
IH5A72VI	2021	Hyland, Sara J; Bro	Perioperative pain management and op	Healthc.	No specific focus on specialists or the professions
EF8IWC26	2022	Ibrahim, Bridget Ba	Inequities in quality perinatal care in th	PLoS One	No specific focus on specialists or the professions
EGVQVA6J	2020	Igboanugo, Somker	Maternal risk factors for birth asphyxia	J. Obstet. Gynaecol	No specific focus on specialists or the professions
G8MU6JJK	2007	Ikiugu, Moses	Psychosocial Conceptual Practice Models in Occupational		Other
CIPXFWCF	2023	Iliopoulos, Ilias D;	Fragility fractures: Risk factors and management strategies		Other
LZ4ZMLII	2024	Im, Hyea Bin; Hwar	Patient-physician communication on h	BMJ Glob. Heal.	No specific focus on specialists or the professions
36RDGK9M	2024	Inchingolo, France	Management of Rheumatoid Arthritis in	Int. J. Environ. Res.	Not on LMICs
BJV7YIX6	2012	Inhorn, Marcia C	Local babies global science: Gender, religion, and in vitro fe		Other
NUIMLGVD	2014	Inhorn, Marcia C; F	Infertility around the globe: New thinkir	Hum. Reprod. Upda	No specific focus on specialists or the professions
AM2WAJLY	2013	Isaacs, David	Evidence-based neonatal infections		Other
NWV9P697	2017	Isenberg, Sarina R;	Implementing evidence-based palliativ	Epidemiol. Rev.	Not on LMICs
DD76CQLG	2023	Isezuo, Khadijat Or	Knowledge, attitude, and practice rega	Ann. Afr. Med.	No specific focus on specialists or the professions
BNKKXUWP	2022	Ishii-Rousseau, Jul	Leveraging Data Science for Global Sur	Sustain. Dev. Goals	No specific focus on specialists or the professions
XEPTSZ8R	2012	Ivers, Noah; Jamtve	Audit and feedback: Effects on professi	Cochrane Databas	No specific focus on specialists or the professions
XTNWWCCH	2018	Iwuh, I A; Fawcus, S	Maternal near-miss audit in the Metro V	S. Afr. Med. J.	No specific focus on specialists or the professions
FEYCIZL7	2020	Iyer, Veena; Mavala	Perceptions of quality of care during bir	Sex. Reprod. Heal. I	No specific focus on specialists or the professions
LQXPNZVY	2022	Jacob, Christine; S	Sociotechnical Factors Affecting Patien	JMIR mHealth uHea	No specific focus on specialists or the professions
YH6DBWBN	2023	Jacob, Thomas J; Be	Posterior Communicating Artery Aneury	CUREUS J. Med. Sci	No specific focus on specialists or the professions
EBXDFULD	2018	Jacobs, R; Kane, M	THE ROLE OF GENDER IN CAREER CHO	12TH Int. Technol. E	Not on LMICs
XHYS56ZB	2015	Jacobson, Terry A;	National lipid association recommenda	J. Clin. Lipidol.	No specific focus on specialists or the professions
XZJCVXQD	2023	Jafree, Sara Rizvi	Social Policy for Women in Pakistan		Other
ZLRU8QRF	2020	Jafree, Sara Rizvi; S	South Asian women's health behavior: Theoretical explanat		Other
GY4KS599	2021	Jain, Kewal K	Drug-induced Neurological Disorders, Fourth Edition		Other
MKVJQDW7	2015	James, Jack E	The Health of Populations: Beyond Medicine		Other
FWBC7HFR	2018	James, Peter Bai; W	Traditional, complementary and alterna	BMJ Glob. Heal.	No specific focus on specialists or the professions
QNW7IVPL	2021	James, Philip T; Ali,	The Role of Nutrition in COVID-19 Susc	J. Nutr.	No specific focus on specialists or the professions
TC4BXQ6G	2018	James, Spencer L; A	Global, regional, and national incidenc	Lancet	No specific focus on specialists or the professions
IF5ZYGSM	2020	James, Spencer L; C	Estimating global injuries morbidity and	Inj. Prev. J. Int. Soc.	No specific focus on specialists or the professions
QJD7KDR4	2022	Jamil, Hashaam; Ta	Interventional radiology in low- and mid	Ann. Med. Surg.	
ZYVB3PBQ	2018	Janse van Rensburg	State and non-state mental health serv	Health Policy and Planning	
YMM3T4BN	2022	Jantsch, Adelson G	Residency training in family medicine a	BMJ Open	No specific focus on specialists or the professions
7SQ5MNXM	2021	Jatoi, Nadia Nazir;	Are doctors protected enough during C	Glob. Heal. Res. po	No specific focus on specialists or the professions
CPRFI62X	2021	Javed, Afzal; Lee, C	Reducing the stigma of mental health d	Asian J. Psychiatr.	No info on specialists' contribution to health systems/population health, or their governance
5AR8PR4J	2014	Jayaraman, Sudha;	Advanced trauma life support training f	Cochrane database	No specific focus on specialists or the professions
M3QVLD7V	2023	Jayasooriya, S; Sto	Clinical standards for the diagnosis and	Int. J. Tuberc. lung D	No specific focus on specialists or the professions
GGWGC6Y5	2003	Jenicek, Milos	Foundations of evidence-based medicine		Other
XC64V6BS	2015	Jenkins, L S; Gunst,	What keeps health professionals workin	African J. Prim. Heal. Care Fam. Med.	
IM9CURIW	2013	Jenkins, Rachel; Ot	Health system challenges to integratio	BMC Health Serv. R	No specific focus on specialists or the professions
NX5TINS4	2021	Jidong, Dung Ezekie	Maternal mental health and child well-	Heal. Psychol. Oper	No specific focus on specialists or the professions
CIVCXAGI	2022	Jie, Christopher Lie	Mechanisms Driving Postgraduate Hea	Acad. Med.	No specific focus on specialists or the professions
DYU7PMAQ	2014	Jingi, Ahmadou M;	Access to diagnostic tests and essentia	PLoS One	No specific focus on specialists or the professions
I53AFV2R	2014	Joffe, Carole; Reich	Reproduction and society: Interdisciplinary readings		Other
HQLAQ4P6	2024	Joglar, José A; Chur	2023 ACC/AHA/ACCP/HRS Guideline fo	J. Am. Coll. Cardiol.	No specific focus on specialists or the professions
T6RKM93C	2014	Johnson, Candace	Maternal transition: A north-south politics of pregnancy and		Other
WW73NT8Z	2021	Johnson, Claire D;	Looking back at the lawsuit that transfo	J. Chiropr. Educ.	Not on LMICs
FHRKMA47	2017	Jolley, Emma; Mafw	Integration of eye health into primary ca	BMC Health Serv. R	No specific focus on specialists or the professions
YERR2AVS	2018	Jones, Desiree; Co	Complementary and Alternative Medic	Integr. Cancer Ther	No specific focus on specialists or the professions
I8W2QEGK	2024	Jones, Stacie M; An	Food insecurity and allergic diseases: A	J. Allergy Clin. Immu	No specific focus on specialists or the professions
DWZL4RJF	2022	Jose, Regi; Subram	Design and Process of Implementation	Asian Pac. J. Cance	No specific focus on specialists or the professions
8DLHKKZJ	2019	Joshi, Rajesh Dhoj;	Evaluation of a Hospital-Based Post-Pr	Am. J. Trop. Med. Hy	No specific focus on specialists or the professions
FMGQSBT6	2018	Joshi, Rohina; Faru	Reporting of ethics in peer-reviewed ve	Int. J. Epidemiol.	No specific focus on specialists or the professions
QSASCS8U	2004	Joshipura, Manjul;	Emergency care in South Asia: challeng	J. Coll. Physicians S	No specific focus on specialists or the professions
EFCRYHNT	2024	Joudeh, Rayan M; J	Illicit drug use among medical students	Ment. Heal. Rev. J.	No specific focus on specialists or the professions
Y2IC7I73	2021	Juhász, Zoltán; Het	Health communication during the COVID-19 pandemic in H		Other
NFZ5ALEA	2011	Kabakyenga, Jerom	Individual and health facility factors an	BMC Pregnancy Ch	No specific focus on specialists or the professions
VZ5J6PLZ	2019	Kabir, Md Faisal; S	Promoting Relational Agent for Health E	J. Med. Syst.	No specific focus on specialists or the professions
4FY6RLX5	2006	Kahn, D; Pillay, S; V	General surgery in crisis - Comparative	South African J. Surg.	
RQQ79GC7	2012	Kaiser, Georges L	Symptoms and signs in pediatric surgery		Other
USMK3JY2	2014	Kaissi, Amer	Flipping health care through retail clinics and convenient c		Other
U5FNMTP6	2013	Kaitelidou, Daphne	Informal payments for maternity health	Health Policy (New	Not on LMICs
KPK5FQB2	2020	Kakembo, Nasser;	Ugandan Medical Student Career Choic	World J. Surg.	No info on specialists' contribution to health systems/population health, or their governance
F4JDC48S	2011	Kakuma, Ritsuko; M	Human resources for mental health car	Lancet	
GLZSHTZM	2024	Kakumanu, Sujani;	Applying the dissemination and implem	J. Allergy Clin. Immu	Not on LMICs
C4R6B4LY	2018	Kampf, Günter	Antiseptic stewardship: Biocide resistance and clinical imp		Other
BN9QJXLK	2015	Kana, Musa Abubak	Maternal and child health interventions	BMC Public Health	No specific focus on specialists or the professions
T8IMZY3Z	2022	Kancherla, Vijaya;	Relationship between achieving Sustai	Birth Defects Res.	No specific focus on specialists or the professions
IJYSS3MT	2017	Kane-Gill, Sandra L	Clinical Practice Guideline: Safe Medic	Crit. Care Med.	No specific focus on specialists or the professions
EZKSKY2X	2020	Kang, Mee Joo; Ngi	Self-reported confidence and perceive	BMC Med. Educ.	No info on specialists' contribution to health systems/population health, or their governance
F5XGNPA2	2022	Kanyesigye, Hamso	Improved maternal–fetal outcomes am	BMC Pregnancy Ch	No specific focus on specialists or the professions
X4VRQVRH	2021	Kaphle, Sabitra	Socio-cultural insights of childbirth in South Asia: Stories of		Other
J2WHJ9JG	2022	Kaphle, Sabitra; Va	Respectful Maternity Care in South Asi	Int. J. Womens. Hea	No specific focus on specialists or the professions
KTC3EPGK	2014	Kaplan, Norman M;	Kaplan's clinical hypertension: Eleventh edition		Other
AI7YKWUU	2023	Kapra, Ori; Asna, N	The Oncology Clinical Nurse Specialist	Curr. Oncol.	
6RFGL4LP	2018	Kapur, Anil; Divaka	Perspectives on diagnostic strategies fo	Diabetes Res. Clin.	No specific focus on specialists or the professions
3H5XET4C	2024	Karam, Pascale E;	Genetic literacy among primary care ph	BMC Med. Educ.	No specific focus on specialists or the professions
U4N7B45W	2015	Karch, Steven B; D	Karch's pathology of drug abuse		Other
989YWWPE	2020	Karekezi, Claire; El	The impact of African-trained neurosurg	Neurosurg. Focus	
R2UVDMJP	2021	Karia, Aleesha; Zan	Socio-Economic Disparities in Access t	Int. J. Environ. Res.	Not on LMICs
H8G6SSVF	2019	Karimi-Shahanjarin	Barriers and facilitators to the impleme	Cochrane Databas	No specific focus on specialists or the professions
59GE7P6G	2023	Kasim, Sazzli Shah	Validation of the general Framingham R	Lancet Reg. Heal. W	No specific focus on specialists or the professions
SSCDVPTB	2014	Kassebaum, Nicho	Global, regional, and national levels an	Lancet	No specific focus on specialists or the professions
2VIL2VWI	2011	Kath, Elizabeth	Revolutionary health: State capacity, p	J. Iber. Lat. Am. Res	No specific focus on specialists or the professions
2FIN2F9C	2023	Kathree, Tasneem;	Management of Depression in Chronic	Community Ment. H	No specific focus on specialists or the professions
WE8ZVWGN	2024	Katz, Anne	Symptom Management Guidelines for Oncology Nursing		Other
GMHWTHU3	2016	Kawonga, Mary; Bla	The influence of health system organiza	Health Policy Plan.	No specific focus on specialists or the professions
6DDAD25T	2023	Kazzi, Ana I M; Dini	Challenging Outlook of Caring for Adole	JCO Glob. Oncol.	No info on specialists' contribution to health systems/population health, or their governance
MXC88H4F	2021	Kebede, Tesfaye; Ey	RADIOLOGY RESIDENTS' PERCEPTION	Ethiop. Med. J.	No info on specialists' contribution to health systems/population health, or their governance
4N2FSTGF	2019	Keijser, Wouter A;	Development of a national medical lea	BMC Med. Educ.	Not on LMICs
4CKX832M	2023	Keikhaee, Razieh; S	Cross-Cultural Adaptation and Psychor	Health Commun.	No specific focus on specialists or the professions
CWGLPZFB	2023	Keles, Elif; Bagci, U	The past, current, and future of neonata	npj Digit. Med.	No specific focus on specialists or the professions
S2VMD78F	2023	Keller, Kresten Kra	Recommendations for early referral of i	Ann. Rheum. Dis.	No specific focus on specialists or the professions
2I828PI5	2021	Kemp, Joy; Maclea	Global Midwifery: Principles, Policy and Practice		Other
WREMACF5	2017	Kempthorne, Peter	The WFSA Global Anesthesia Workforce	Anesth. Analg.	
Y2WE5XWL	2024	Kennedy, Ciaran; Ig	Commonalities and differences in injur	BMJ Open	No specific focus on specialists or the professions
MS9BRUV3	2024	Kenner, Carole; Bo	Neonatal Nursing Care from a Global P	Crit. Care Nurs. Cli	No specific focus on specialists or the professions
6QG96G34	2014	Kernan, Walter N;	Guidelines for the prevention of stroke i	Stroke	No specific focus on specialists or the professions
AGXB2BL2	2020	Kerr, Aisling; O'Con	A scoping review of health professional	Res. Soc. Adm. Pha	No specific focus on specialists or the professions

Appendix 4

Table S4: List of papers excluded, with reason

MTD6ID69	2013	Kerry, Vanessa B; V	US medical specialty global health trai	J. Glob. Health	Not on LMICs
7S5RYVAQ	2020	Keshri, Vikash Ranj	Reforming the regulation of medical ed	BMJ Glob. Heal.	
L84QIRMT	2018	Keya, Kaji Tamanna	"Poverty is the big thing": Exploring fina	Int. J. Equity Health	No specific focus on specialists or the professions
4B6MT9V4	2024	Khadka, Sushil; Sub	Collaborative leadership to empower n	Leadersh. Heal. Ser	No specific focus on specialists or the professions
9DY8YPNX	2021	Khajuria, Ankur; To	Workplace factors associated with me	BMC Health Serv. R	No specific focus on specialists or the professions
JHMWYJLM	2020	Khan, Mishal S; Bo	Is enhancing the professionalism of hea	Hum. Resour. Healt	No specific focus on specialists or the professions
FMUII8IX	2013	Khanal, Vishnu; Sa	Exclusive breastfeeding practices in re	BMC Public Health	No specific focus on specialists or the professions
7B3EJQ3U	2014	Khatun, Fatema; H	Prospects of mHealth to Improve the Health of the Disadva		Other
G8CKRUYU	2024	Khera, Nandita; Ai	Trends in volumes and survival after he	Blood Adv.	No specific focus on specialists or the professions
ASLS7RY9	2023	Khodneva, Yulia; Le	Disparities in Postdischarge Ambulator	J. Am. Heart Assoc.	Not on LMICs
QJZK9ZAF	2023	Khosla, Muskaan; V	New Media Platforms for Teaching and	Breast Care (Basel	No info on specialists' contribution to health systems/population health, or their governance
3VLQGA4H	2018	Khubchandani, Jas	Disparities in access to emergency gen	Surgery	Not on LMICs
3NDKFYC5	2009	Kidanto, Hussein L	Criteria-based audit on management o	BMC Pregnancy Ch	No specific focus on specialists or the professions
C8G7UG5G	2019	Kienle, G S; Ben-Ar	Contributing to Global Health: Develop	Evidence-based Co	Not on LMICs
I6HLSZ66	2023	Kietaibl, Sibylle; Ah	Management of severe peri-operative b	Eur. J. Anaesthesio	No specific focus on specialists or the professions
TKUDRYMP	2022	Killeen, Sarah Loui	Addressing the gaps in nutritional care	Proc. Nutr. Soc.	No specific focus on specialists or the professions
K2MU2C4C	2020	Kilshaw, Susie	Introduction: Ambiguities and navigations		Other
ATKKW48Y	2017	Kim, Baek-Nam; Pa	Cefotaxime		Other
Y9Z47QYW	2017	Kim, Baek-Nam; Pe	Ceftriaxone		Other
VLQBJCYS	2021	Kim, June-Ho; Bell,	Predictors of patient-reported quality o	Int. J. Qual. Heal. c	No specific focus on specialists or the professions
6K3LU4YL	2023	Kim, Sejin; Jeong, H	Extracurricular activities in medical ed	BMC Med. Educ.	No info on specialists' contribution to health systems/population health, or their governance
Q4MG2U2F	2024	Kimbowa, Isaac Ma	The role of medicines and therapeutics	PLoS One	No specific focus on specialists or the professions
YMJC7GA2	2021	Kingsley, Jennifer P	The Changing Aspects of Motherhood in	Matern. Child Heal	No specific focus on specialists or the professions
M64585AX	2020	Kinshella, Mai-Lei V	Barriers and facilitators to implementi	Public Health Rev.	No specific focus on specialists or the professions
7BQMTKFQ	2016	Kirchhof, Paulus; B	2016 ESC Guidelines for the managem	Eur. J. Cardio-thora	No specific focus on specialists or the professions
HHMDJEAF	2023	Kirchner, Varvara A	Management of Established Small-for-s	Transplantation	No specific focus on specialists or the professions
26GNR959	2013	Kirkpatrick, Andrew	Enabling the mission through trans-atla	Telemed. J. e-healt	No info on specialists' contribution to health systems/population health, or their governance
V6DWAILZ	2021	Kirubarajan, Abira	Barriers to fertility care for racial/ethni	F S Rev.	No specific focus on specialists or the professions
KQNZLTAS	2024	Klassen, Sheila L; C	Decentralization and Integration of Adv	Glob. Heart	No info on specialists' contribution to health systems/population health, or their governance
2372LC3N	2023	Klein, A; Berger, T C	Does the presence of a specialist docto	EPILEPSY & Behav.	
FDSGYCU6	2002	Klein, Catherine J	Nutrient requirements for preterm infa	J. Nutr.	No specific focus on specialists or the professions
7WPYAMZU	2016	Klingler, Corinna; N	Difficulties experienced by migrant phy	Hum. Resour. Healt	Not on LMICs
U9NA42VS	2021	Klionsky, Daniel J; A	Guidelines for the use and interpretatio	Autophagy	No specific focus on specialists or the professions
FPZ6SWTG	2016	Klionsky, Daniel J; A	Guidelines for the use and interpretatio	Autophagy	No specific focus on specialists or the professions
AWYD9M2E	2023	Knight, Amber; Mill	Prenatal Genetic Testing, Abortion, and Disability Justice		Other
7P52XSKE	2016	Knight, Jane	THE COMPLETE GUIDE TO FERTILITY AWARENESS		Other
7XE8UFQZ	2019	Knoll, K M; Philippi	Emigration-an option for African reside	OPHTHALMOLOGE	No info on specialists' contribution to health systems/population health, or their governance
H934VN38	2013	Knowlton, Lisa Mar	Liberian surgical and anesthesia infrast	World J. Surg.	Other
6J78AFX5	2022	Kobayashi, Sarah; A	A Framework to Guide the Developmen	Clin. Geriatr. Med.	No specific focus on specialists or the professions
I95ZH6HL	2023	Kobylarz, Damian;	Antidotes in Clinical Toxicology—Critic	Toxics	No specific focus on specialists or the professions
KUF8Y4CP	2022	Kocarnik, Jonathan	Cancer Incidence, Mortality, Years of L	JAMA Oncol.	No specific focus on specialists or the professions
SIGFIC5H	2023	Koech, Hilary; Alba	Minority Resident Physicians' Perspect	J. Surg. Educ.	No info on specialists' contribution to health systems/population health, or their governance
XR6IUX5B	2023	Koenig, Harold G; V	HANDBOOK OF RELIGION AND HEALTH: THIRD EDITION		Other
9LG5ZZIS	2024	Koi-Larbi, Koiwah;	Advancing hypertensive disorders of pr	BMC Proc.	No specific focus on specialists or the professions
PTF79MMX	2021	Koichubekov, B; Kh	System dynamics modeling for general	Ann. di Ig. Med. Prev	No specific focus on specialists or the professions
4Q6BG763	2021	Koltai, Deborah C;	Healthcare provider perspectives rega	Epilepsy Behav.	No specific focus on specialists or the professions
LG647WEN	2016	Komaromy, Miriam	Project ECHO (Extension for Communi	Subst. Abus.	No specific focus on specialists or the professions
4C9EXJC3	2024	Kontou, Angeliki; A	Antibiotics, Analgesic Sedatives, and A	Children	No specific focus on specialists or the professions
DU58YTKV	2021	Koo, Minjoung Mon	Conceptual framework to guide early di	J. Glob. Oncol.	No specific focus on specialists or the professions
P7CR8KVX	2018	Kool, E M; Bos, A M	Ethics of oocyte banking for thirdparty a	Hum. Reprod. Upda	No specific focus on specialists or the professions
G5I2Q7NJ	2022	Kopanczyk, Rafal; L	Developing Cardiothoracic Surgical Cr	Med.	Not on LMICs
Q8AYTC5Y	2022	Kost, Amanda; Phil	The Influence of Role Modeling and Me	Fam. Med.	No specific focus on specialists or the professions
6M6LZU77	2006	Kotzee, T J; Couper	What interventions do South African qu	Rural Remote Health	
T4PUE2K2	2023	Kovacevic, Pedja; D	Boosting ICU capacity during the COVI	J. Public health Res	No info on specialists' contribution to health systems/population health, or their governance
MAWIAI3R	2012	Kraemer, M; Berlit,	What is the expert's option on antiplate	Eur. J. Neurol.	No info on specialists' contribution to health systems/population health, or their governance
JJFQXBT5	2019	Kraft, Colleen S; Ko	The Special Pathogens Research Netwo	Heal. Secur.	No info on specialists' contribution to health systems/population health, or their governance
DY4L3CVW	2021	Krakauer, Eric L; Ka	Essential package of palliative care for	JCO Glob. Oncol.	
3DZ8C585	2019	Kreitler, Shulamith	Psycho-Oncology for the Clinician: The Patient Behind the D		Other
6QJHZV26	2007	Krueger, Hans; Wil	The health impact of smoking and obesity and what to do ab		Other
B9K5HYIM	2010	Kruk, Margaret E; W	Human resource and funding constrain	PLoS Med.	
KNP5J2AY	2009	Krupp, Karl; Madhiv	Leveraging human capital to reduce ma	Hum. Resour. Health	
L7U2MDN6	2021	Kruse, Clemens Sc	Telemedicine and health policy: A syste	Heal. Policy Techno	No specific focus on specialists or the professions
KKQ6K6PQ	2015	Krymchantowski, A	The pharmacological treatment of migr	Headache	
5ZMFFLSP	2018	Kudsk-Iversen, S; S	Strengthening the Anesthesia Workforc	Anesth. Analg.	
H8QVPSE2	2022	Kuhn, Jens H; Adkin	2022 taxonomic update of phylum Neg	Arch. Virol.	No specific focus on specialists or the professions
YQ5LG7DU	2024	Kumar, Rajeev; Jos	The ethical frontier of AI and data analysis		Other
HD8PI64Y	2022	Kumar, Vasantha K	Handbook on Opium: History and Basis of Opioids in Therap		Other
D7C8DAJ2	2019	Kundumadam, Sha	Variations in Screening Adenoma Dete	Cureus	Not on LMICs
R3HAPN8M	2022	Kunkel, Katherine J	2021 Update for the Diagnosis and Ma	J. Cardiothorac. Va	No specific focus on specialists or the professions
59YVLUGS	2018	Kurdin, Anton; Cai	TEAM: A Low-Cost Alternative to ATLS fo	J. Surg. Educ.	No specific focus on specialists or the professions
FQY65DQN	2011	Labov, Joanna; Ha	Pronunciation as life and death: Impro	Innov. Leadersh. En	Not on LMICs
7744JRUY	2008	Lachter, Jesse; Les	Overcoming barriers to colorectal can	Isr. Med. Assoc. J.	Not on LMICs
R9MYFKR9	2024	Ladadwa, Reem; H	Health information management syste	Global. Health	No specific focus on specialists or the professions
QFEUDRNN	2009	Lafata, Jennifer E;	Sustained hyperglycemia among patie	Diabetes Care	No specific focus on specialists or the professions
N9NB88WU	2024	Lagarde, Mylene; S	PHYSICIAN BEHAVIOUR AND INEQUALI	Contrib. to Econ. A	Other
FRFEQ9MR	2022	Lai, Ruby Y S	Premarital abortion in China: Intimacy, family and reprodu		Other
I56RX5LV	2021	Lalghrani, Ame	Regulating Assisted Reproductive Technologies: New Horiz		Other
E8W8ZCF8	2020	Lam, Stephen; Bry	Management of screen-detected lung n	Can. J. Respir. Crit.	No specific focus on specialists or the professions
2UZ4MAY4	2023	Lan, Shaowen; Fa	Physician scheduling problem in Mobil	Ann. Math. Artif. Int	No specific focus on specialists or the professions
QCECTG4M	2024	Lanerolle, Gayath	Quality Assurance Management: A Comprehensive Overvie		Other
PDMYCKX6	2011	Langan-Fox, Janice	Intern coping, stress and patient-adverse events: The huma		Other
446MXNVM	2015	Langer, Ana; Melei	Women and Health: The key for sustain	Lancet	Other
QRFGQFLT	2020	Lantz, Adam; Holm	Measuring the migration of surgical spe	Surg. (United States)	
CXHN23H3	2023	Lapidus, Aaron; Sh	Medical student intentions to practice	BMC Med. Educ.	Not on LMICs
J8HGDTEQ	2020	Lappeman, Maura;	'I Don't Want to See that the People Ar	Br. J. Psychother.	No specific focus on specialists or the professions
5UBQN32Y	2011	Lara Munoz, Maria	How many are we? Where are we? Whe	SALUD Ment.	
845MBQKX	2014	Lassi, Zohra S; Das	Evidence from community level inputs	Reprod. Health	No specific focus on specialists or the professions
Z8IMZNV2	2019	Lassi, Zohra S; Ked	Community-based maternal and newb	Cochrane Databas	No specific focus on specialists or the professions
UQDAPJHY	2015	Lathan, Christophe	Lung cancer care: The impact of faciliti	Transl. Lung Cance	Not on LMICs
HSYDYGNU	2018	Lau, Gary K K; Pend	Transient Ischemic Attack and Stroke Diagnosis, Investigati		Other
WF3S3SZB	2014	Lautier, Marc	International trade of health services:	Health Policy (New	No specific focus on specialists or the professions
MRMJ9HKX	2023	Lawler, Patrick R; D	Effect of Angiotensin-Converting Enzym	JAMA	No specific focus on specialists or the professions
UZJ54DEM	2013	Lawn, Joy E; David	Born Too Soon: Care for the preterm ba	Reprod. Health	No specific focus on specialists or the professions
YVN9G4VI	2013	Lawrence, Mark	Food Fortification: The evidence, ethics, and politics of add		Other
WH6EN7BC	2024	Lawrence, Monica	Disparities in Diagnosis, Access to Spe	J. Allergy Clin. Imm	No specific focus on specialists or the professions
KIWLTPES	2016	Lazenby, Mark; Se	Symptom burden and functional depe	Cancer Nurs.	No specific focus on specialists or the professions
X3P5HHRU	2011	Lazenby, Ramona E	Handbook of pathophysiology: Fourth edition		Other
RBLMVTBV	2020	Lederman, Regina	Psychosocial Adaptation to Pregnancy: Seven Dimensions o		Other
28X79K3V	2022	Ledesma, Jorge R;	Global, regional, and national sex differ	Lancet Infect. Dis.	No specific focus on specialists or the professions

Appendix 4

Table S4: List of papers excluded, with reason

4QZHXGHM	2022	Lee, Eric K P; Poon,	Global Burden, Regional Differences, T	J. Am. Heart Assoc.	No specific focus on specialists or the professions
CVFUNJDA	2024	Lee, Hwa-Young; C	Regional determinants of quality of car	J. Glob. Health	
BVWYYR4P	2020	Lee, Jessica S; Ros	Oral and Maxillofacial Surgery in Low-In	Oral Maxillofac. Su	No info on specialists' contribution to health systems/population health, or their governance
U3GGR78K	2023	Leke, Aminkeng Zav	The burden, prevention and care of infa	PLOS Glob. Public H	No specific focus on specialists or the professions
PK8IVFL7	2019	Leng, Anli; Jing, Jun	Geographical disparities in treatment a	BMC Cancer	No specific focus on specialists or the professions
WNX5KVZG	2003	Leon, Moises	Perceptions of health care quality in Ce	Int. J. Qual. Heal. ca	No specific focus on specialists or the professions
BQE4TDCD	2010	Leow, J J; Kingham,	Global Surgery: Thoughts on an Emergin	J. Surg. Educ.	No info on specialists' contribution to health systems/population health, or their governance
BGL8RXPM	2021	Leung, Chi Hung C	Mechanical Ventilation Discontinuatio	Ann. Am. Thorac. So	No info on specialists' contribution to health systems/population health, or their governance
I3H59B3E	2024	Léveillé, Nayla; Pro	Exploring Prognostic Implications of Ra	J. Surg. Res.	No specific focus on specialists or the professions
ANDDDGDF	2021	Levenets, Olena; S	Coping with cancer in post-communist	Health Policy Plan.	No specific focus on specialists or the professions
YMZKLCJY	2023	Leversedge, Chelse	Capacity Building During Short-Term Su	World J. Surg.	
8IIAIEZU	2016	Levesque, Janelle V	Cancer, chronic conditions and social disadvantage-the pe		Other
MXZRKBKG	2024	Levin, Adeera; Ahm	KDIGO 2024 Clinical Practice Guidelin	Kidney Int.	No specific focus on specialists or the professions
4XUZ4RD4	2023	Levitt, Peggy; Dobb	Transnational Social Protection: Social Welfare Across Nat		Other
4MMSNK4U	2019	Lewiecki, E Michae	Proceedings of the 2018 Santa Fe Bone Symposium: Advanc		Other
A9IQ4ZZ8	2010	Lewin, Simon; Mun	Lay health workers in primary and comm	Cochrane Databas	No specific focus on specialists or the professions
JEXIKZWB	2016	Lewis, Cara; Darne	Proceedings of the 3rd Biennial Confer	Implement. Sci.	Other
YI7CS8BG	2011	Lewis, Carol M; We	Quality of care in head and neck cancer	Curr. Oncol. Rep.	No info on specialists' contribution to health systems/population health, or their governance
PCBNPTD9	2023	Li, Bo; Zhang, Ruid	Current Anesthesia Practices of Pediat	J. Cardiothorac. Va	No info on specialists' contribution to health systems/population health, or their governance
TDELDIJF	2024	Li, Qian; Piaseu, N	Effects of a Comprehensive Dietary Inte	Nutrients	No specific focus on specialists or the professions
7DP5FMN4	2023	Li, Tingting; Zeng, Y	A Bibliometric Analysis of Research Art	J. Multidiscip. Heal	No specific focus on specialists or the professions
K86TUHTR	2023	Li, Wen; Gillies, Ro	Specialty preferences of studying-abro	BMC Med. Educ.	No info on specialists' contribution to health systems/population health, or their governance
7FQBBXM6	2021	Li, Wen; Gillies, Ro	Barriers and facilitators to online medi	Hum. Resour. Healt	No specific focus on specialists or the professions
F7KZMG8I	2018	Li, Zhang; Anindith	Burden of cancer pain in developing co	Clin. Outcomes Res	No specific focus on specialists or the professions
SDWT2LHQ	2019	Liang, Li-Lin	Impact of integrated healthcare: Taiwa	Health Policy Plan.	No specific focus on specialists or the professions
EX86Z2MP	2020	Liao, Peng-da; Che	Clinical Practice Guideline of Integrati	Chin. J. Integr. Med	No specific focus on specialists or the professions
GE5XQIFD	2022	Lidströmer, Niklas;	AIM in Primary Healthcare		Other
RF2QE33P	2021	Liese, Kylea L; Davi	Obstetric iatrogenesis in the United Sta	Anthropol. Med.	Not on LMICs
DAFMSQ93	2013	Light, Richard W	Pleural diseases: Sixth edition		Other
NR48UEKN	2018	Lin, Li-Rong; Xiao, Y	Development of tissue inflammation ac	BMC Infect. Dis.	No specific focus on specialists or the professions
S4969XDJ	2022	Lin, Ping-Lun; Huan	A survey of specialty choice among obs	J. Obstet. Gynaecol	Not on LMICs
7FK7YHCH	2012	Linden, Allison F; S	Challenges of surgery in developing cou	World J. Surg.	No info on specialists' contribution to health systems/population health, or their governance
TYMG6L9B	2012	Lindsay, K L; Gibne	Maternal nutrition among women from	J. Hum. Nutr. Diet.	No specific focus on specialists or the professions
WGE7L3IR	2020	Lindstrom, Nataliy	Person and Family Centeredness in Eth	JMIR Res. Protoc.	Other
DA5EIW2Z	2013	Lingard, Lorelei A;	Understanding palliative care on the he	J. Pain Symptom Ma	Other
HBU3ANJY	2015	Linhares, Jose Juve	Construction of a competence-based c	SAO PAULO Med. J.	Other
33P8I8BY	2024	Linzer, Mark; Mallio	Resident worklife and wellness through	BMC Med. Educ.	Not on LMICs
R5CV7NFE	2018	Lissauer, David; W	Prophylactic antibiotics to reduce pelv	Trials	Other
NV3V9XCD	2013	Little, James W; Fa	Little and Falace's Dental Management of the Medically Co		Other
TW8CNHDP	2015	Liu, Chuan; Wang,	Factors related to health-related quali	BMC Health Serv. R	No info on specialists' contribution to health systems/population health, or their governance
8HV3DI4D	2019	Liu, Jie; Zhang, Xue	Analysis of clinical and electrophysiolo	Neurol. Sci.	No specific focus on specialists or the professions
B9UMIDLX	2024	Liu, Paicheng; Che	Medical errors, affected sites, and adve	Front. public Heal.	No specific focus on specialists or the professions
SLMMAHWL	2022	Liu, Paicheng; Yang	Gender differences in medical errors a	Front. public Heal.	No specific focus on specialists or the professions
2KDREGQ6	2020	Liu, X; Wang, F; Din	Strengthening the maternal and child h	Public Health	No specific focus on specialists or the professions
9QV5H5PT	2024	Liu, Yuwen; Chen,	Application of AI-assisted MRI for the id	BMC Musculoskele	No specific focus on specialists or the professions
37LFF88R	2024	Liu, Zhe; Liu, Fang;	Uncovering the ceRNA Network Relate	Biochem. Genet.	No specific focus on specialists or the professions
AH4HI9YQ	2022	Lo, Jamie J; Yoon, S	Factors Influencing Potentially Futile T	Am. J. Hosp. Palliat	No specific focus on specialists or the professions
XLXNMVXP	2011	Loiselle, Carmen G	Canadian essentials of nursing research		Other
Q9QZTY38	2023	Lombe, Dorothy Ch	Delays in seeking, reaching and access	BMJ Open	No specific focus on specialists or the professions
K8XAX7PU	2018	Longman, Jo M; Ada	Improving implementation of the smoki	Midwifery	No specific focus on specialists or the professions
AT365IAX	2009	Loo, May	Integrative Medicine for Children		Other
5S2ZMMJK	2019	Lopes, Marcelo Ant	Guideline of the Brazilian society of ca	Arq. Bras. Cardiol.	No info on specialists' contribution to health systems/population health, or their governance
TPEQ9Z3S	2016	López-Medrano, F;	Clinical Presentation and Determinant	Am. J. Transplant.	No specific focus on specialists or the professions
SEWRLTVD	2022	Lopez, Keila N; Bak	Addressing Social Determinants of Hea	J. Am. Heart Assoc.	No specific focus on specialists or the professions
XH4LA4SL	2022	Loredo-Abdala, Art	Pediatric Abusive Head Trauma: Multic	J. Interpers. Violen	No specific focus on specialists or the professions
PDMTW9LU	2018	Lorentz, J; Liu, S K;	Male Oncology Research and Educatio	Curr. Oncol.	No specific focus on specialists or the professions
YRRPZ8QU	2021	Lorenz, Birgit; Tava	Current Management of Inherited Reti	Ophthalmic Res.	Not on LMICs
VMCU8E3E	2021	Losonczy I, Lia; Pap	White Paper on Early Critical Care Serv	Ann. Glob. Heal.	Other
LNT8STKF	2020	Loue, Sana	Case studies in society, religion, and bioethics		Other
BP4X5Y4X	2022	Loue, Sana; Johnso	Diversity, Cultural Humility, and the Helping Professions: B		Other
F2UREPRE	2023	Louw, Johnelize; M	Factors Contributing to Delays to Acce	World J. Surg.	No specific focus on specialists or the professions
3LD5QU3X	2014	Love, Allison; Yates	Advance Care Planning in palliative car	Palliat. Med.	No specific focus on specialists or the professions
X8K42HCY	2017	Loveday, Jonathan;	Survey of Emergency and Essential Surg	World J. Surg.	
QGCTDA7Y	2019	Lovero, Kathryn L;	Mixed-methods evaluation of mental h	BMC Health Serv. R	No specific focus on specialists or the professions
WFS4AMSK	2021	Løvseth, Lise Tevik	Quality of care		Other
9RMXH4LP	2021	Lowe, Dianne; Rya	Effects of consumers and health provid	Cochrane Databas	No specific focus on specialists or the professions
VS9WK95I	2021	Lozar, Taja; Nagvek	Cervical cancer screening postpandem	Int. J. Womens. Hea	No specific focus on specialists or the professions
KZTLCQS6	2010	Lu, Michael C; Kote	Innovative strategies to reduce disparit	Med. Care Res. Rev	No specific focus on specialists or the professions
JDU6LVZV	2009	Luboga, Sam; Macf	Increasing access to surgical services i	PLoS Med.	Other
JPX9BPFT	2009	Luboga, Samuel; G	Recasting the role of the surgeon in Ug	Trop. Med. Int. Heal.	
F8BS6Y4T	2022	Lucero-Prisno, Dor	Top ten public health challenges to tra	Public Heal. Challe	No specific focus on specialists or the professions
KJ935XDY	2021	Luckett, R; Nassali	Development and launch of the first ob	BMC Med. Educ.	
WJWBDBZL	2019	Luckett, Rebecca;	Promoting Health Equity Through Purpo	J. Surg. Educ.	Not on LMICs
5ZY4CVFU	2013	Luckett, Tim; David	Do community specialist palliative car	J. Pain Symptom Ma	Not on LMICs
AGN6YMJ7	2024	Ludeña-Poma, Jorg	Influence of resistance to use, perceive	Pharmacia	No specific focus on specialists or the professions
FQ6MNF3A	2012	Lueddeke, George	Transforming medical education for the 21st century: Mega		Other
CAWZYF29	2023	Lukama, Lufunda; A	Ear, Nose and Throat (ENT) disease dia	PLoS One	
S5824WUU	2020	Lukama, Lufunda; K	Availability of ENT Surgical Procedures	Biomed Res. Int.	No specific focus on specialists or the professions
63UQ82IM	2017	Lukas, Rimas V; Ma	Views on Careers in Clinical Neuroscie	World Neurosurg.	No info on specialists' contribution to health systems/population health, or their governance
YGIPTULR	2016	Lundblad, Roger L	Development and application of biomarkers		Other
G8KJ5SMA	2012	Lundblad, Roger L	Biotechnology of plasma proteins		Other
A3ADX27J	2017	Lundin, Johan; Du	Medical mobile technologies - what is r	Glob. Health Action	No specific focus on specialists or the professions
FVJKTMCV	2014	Lungren, Matthew	Educational strategies and volunteering in global health rad		Other
ZDB9UH3S	2021	Luntsi, G; Ugwu, A	Achieving universal access to obstetric	Radiography	No specific focus on specialists or the professions
WMT8NGNL	2016	Luppa, Peter B; Bie	Clinically relevant analytical technique	Biotechnol. Adv.	No specific focus on specialists or the professions
MQJPMI9Q	2020	Luyckx, Valerie A;	Preventing CKD in Developed Countrie	Kidney Int. Reports	Not on LMICs
57ZLCFSA	2016	Lyon, Camila B; Me	Anesthetic Care in Mozambique.	Anesth. Analg.	
7J8V24H6	2017	Lyon, G M; Mehta,	Clinical Management of Patients with E	MARBURG- AND EB	Other
752Y4VVI	2024	Lyren, Anne; Haine	Racial and ethnic disparities in commo	BMJ Qual. Saf.	No specific focus on specialists or the professions
VYTPXJRW	2021	M'Pele, P	Mercy Ships and Africa Mercy hospital	Bull. L Acad. Natl. M	Other
4BRERDZJ	2022	Ma, Huan; Zhang, X	A bispecific nanobody dimer broadly ne	Cell Discov.	No specific focus on specialists or the professions
P4NLXCR5	2024	Ma, Terence P	Considerations for implementation of a competency-based		Other
I63SUVFP	2020	Ma, Xiya; Vervoort,	Emergency and essential surgical healt	Int. J. Surg.	No specific focus on specialists or the professions
4D2YPSGY	2024	Ma, Yulong; Mi, Tia	Public Knowledge and Awareness of An	Anesth. Analg.	No info on specialists' contribution to health systems/population health, or their governance
8KYAW8QC	2022	Mabunda, Dirceu;	Cultural adaptation of psychological in	Int. J. Ment. Health	No specific focus on specialists or the professions
JN5XTGJX	2023	Mabunda, Sikhumb	Effectiveness of return-of-service sche	BMJ Glob. Heal.	No specific focus on specialists or the professions
SWZZ55T3	2019	Mabuza, Mbuso Pr	Evaluating international public health issues: Critical reflec		Other
5GTEDL3C	2021	Machado Fonseca,	Who is going to turn on the ventilators?	EINSTEIN-SAO PAU	Other
2FELSJMY	2024	MacKechnie, Made	Leadership development training for or	OTA Int.	No info on specialists' contribution to health systems/population health, or their governance

Appendix 4

Table S4: List of papers excluded, with reason

MP8MXKYP	2017	Madi, Haifa	Determinants of neonatal and perinatal mortality: A global p		Other
XEEQUYZF	2010	Madu, Chikezie O;	Novel diagnostic biomarkers for prostat	J. CANCER	No specific focus on specialists or the professions
TPEZPX7L	2020	Maghsoudi, Tahere	The role of collaborative healthcare in i	Sustain.	No specific focus on specialists or the professions
LIZZX5KB	2014	Magill, Shelley S; E	Multistate point-prevalence survey of h	N. Engl. J. Med.	Not on LMICs
GFRYE84X	2018	Mahapatro, Meera	Domestic violence and health care in India: Policy and prac		Other
VXPCX4QT	2021	Maïga, Mahamane	The contribution of family medicine to c	African J. Prim. Hea	No specific focus on specialists or the professions
IEMZAKLV	2023	Makkad, Benu; Hei	Practice Advisory for Preoperative and I	Anesth. Analg.	No specific focus on specialists or the professions
X5KEQRRE	2015	Malhi, Gin S; Basse	Royal Australian and New Zealand Coll	Aust. N. Z. J. Psychia	Not on LMICs
WPSHCQT9	2023	Malik, Amyn A; Ahm	Behavioral interventions for vaccinatio	Health Policy (New	No specific focus on specialists or the professions
R9ZNI729	2019	Malik, Ankita; Jee, E	Preeclampsia: Disease biology and burd	Pregnancy Hyperte	No specific focus on specialists or the professions
5HETIIJ5	2021	Malik, Khafsa; Ahm	Herbals of Asia: Prevalent Diseases and Their Treatments		Other
3WJHXZU5	2023	Malviya, Rishabha;	Remote Patient Monitoring: A Computational Perspective in		Other
6WZ3EKF3	2012	Manchikanti, Laxm	Assessment of infection control practic	Pain Physician	No specific focus on specialists or the professions
R6K9JFD3	2020	Mancini, Michael. A	Integrated behavioral health practice		Other
36PHKV4F	2023	Mandal, Saikat; Sm	Perioperative anaemia management	Ann. Blood	No specific focus on specialists or the professions
AKG4Z74E	2023	Mandato, Vincenzo	Primary Ovarian Leiomyosarcoma Is a V	Cancers (Basel).	No specific focus on specialists or the professions
DRG8K5FY	2013	Mander, Rosemary	The politics of maternity		Other
HCUZHBHZ	2010	Manderson, Lenore	Chronic conditions, fluid states: Chronicity and the anthrop		Other
7KVNCLGS	2017	Mandeville, Kate L;	Specialty training for the retention of M	Soc. Sci. & Med.	
3BXES4HE	2016	Mandeville, Kate L;	The use of specialty training to retain do	Soc. Sci. Med.	
KQAUW78E	2021	Manjooran, G; Scri	Career choice of anaesthetists in a dep	South. AFRICAN J. A	No info on specialists' contribution to health systems/population health, or their governance
LF2N2AVV	2015	Mannava, P; Durra	Attitudes and behaviours of maternal h	Global. Health	No specific focus on specialists or the professions
PLKNSGXX	2019	Mannix, Rebekah; Z	Practice Patterns in Pharmacological a	J. Neurotrauma	No specific focus on specialists or the professions
4B6F5WJS	2018	Mansouri, Alireza;	Exploratory Analysis into Reasonable Ti	World Neurosurg.	No info on specialists' contribution to health systems/population health, or their governance
UM8KD4HP	2020	Manuel, Leonardo;	Human toxoplasmosis in Mozambique:	Parasites and Vecto	No specific focus on specialists or the professions
N5WY65G7	2021	Mapanga, Witness;	Consensus study on the health system a	PLoS One	No specific focus on specialists or the professions
K8TRICI6	2023	Mapanga, Witness;	Drivers of disparities in stage at diagnos	PLoS One	No specific focus on specialists or the professions
DNN5XGIG	2020	Marathe, Shweta; I	The impacts of corporatisation of health	BMJ Glob. Heal.	
5W5N999M	2023	Marawar, Rohit	Seizure Safari: Bridging the Treatment G	EPILEPSY Curr.	
SDYI4IMD	2021	Marcolino, Milena	Development and Implementation of a	J. Med. Internet Res	No specific focus on specialists or the professions
PFS2TCHU	2020	Marcum, James A	From Systems Biology to Systems Medicine		Other
FFPF4F5G	2024	Marcum, James A	The Conceptual Foundations of Systems Medicine		Other
UN8PFKZF	2011	Marcus, Dawn A; D	Fibromyalgia: A practical clinical guide		Other
R52NARDV	2023	Marijon, Eloi; Nara	The Lancet Commission to reduce the g	Lancet (London, En	No specific focus on specialists or the professions
UT2AUZRH	2015	Marik, Paul Ellis	Evidence-based critical care, third edition		Other
RL9N48VE	2010	Marik, Paul Ellis	Handbook of evidence-based critical care		Other
CUS5NV67	2024	Marincowitz, Gert	Causes of stillbirths at Kgapane hospita	South African Fam.	No specific focus on specialists or the professions
FHEA5B8U	2011	Marino, Maria; Mas	Nutrition and human health from a sex-	Mol. Aspects Med.	No specific focus on specialists or the professions
42S9XKI5	2020	Marmor, Michael F	Anti-Infectives		Other
HRTHL824	2013	Marquis, Bessie L;	Leadership roles and management functions in nursing: The		Other
9KQBG8QI	2013	Mars, Maurice	Telemedicine and advances in urban ar	Prog. Cardiovasc. D	No specific focus on specialists or the professions
IFY4Q2Y9	2022	Martelletti, Paolo	Migraine in Medicine: A Machine-Generated Overview of Cu		Other
RNXV3P7S	2009	Martin, Graham P;	Reconfiguring or reproducing intra-prof	Soc. Sci. & Med.	Not on LMICs
2FQEV4JL	2023	Martin, M G; Chide	Impact of 10-Day Fulbright Specialist P	J. cancer Educ. Off.	No info on specialists' contribution to health systems/population health, or their governance
2E7T7IU3	2023	Marx, Nikolaus; Fe	2023 ESC Guidelines for the manageme	Eur. Heart J.	No specific focus on specialists or the professions
6SYQJ7Y5	2019	Massenga, Alicia; C	Indications for and complications of int	BMC Gastroentero	No specific focus on specialists or the professions
XKBGXN9B	2023	Mateso, G Q; Maka	Etiologies and factors associated with r	HELIYON	No specific focus on specialists or the professions
WXYE46VB	2024	Mateus, Julio; Stev	Fetal and Maternal Factors Predictive c	Am. J. Perinatol.	No specific focus on specialists or the professions
LK3PUXHI	2008	Mathur, Purva	Hospital acquired infections: Prevention & control		Other
4GZ35LGH	2017	Matlala, Moliehi; G	Structure and activities of pharmacy ar	Expert Rev. Clin. Ph	No specific focus on specialists or the professions
G3CT4MIA	2021	Matsushita, Tomon	Interventions to increase access to lon	Cochrane Databas	No specific focus on specialists or the professions
A5LDGVME	2017	Matsuzaka, Camila	Task shifting interpersonal counseling fo	BMC Psychiatry	No specific focus on specialists or the professions
YFYPKD5F	2019	Matthews-Trigg, Na	Understanding perceptions of global he	BMJ Open	Not on LMICs
NLNFLNS5	2021	Matthews, Elizabet	Shared Decision Making in Primary Car	Front. Psychiatry	No specific focus on specialists or the professions
UCGDTGCT	2020	Mattison, Cristina	A critical interpretive synthesis of the r	Heal. Res. Policy Sy	No specific focus on specialists or the professions
NK43ELR6	2024	Matuozzo, Daniela	Correction: Rare predicted loss-of-function variants of type		Other
IVIZLMWR	2004	Mayhew, Susannah	Sexual and reproductive health: challer	Health Policy Plan.	No specific focus on specialists or the professions
C24EDU3N	2004	Mayhew, Susannah	Improving health systems and enhancin	Health Policy Plan.	
L7RPD8B4	2022	Mayorga-Butron, Jo	Clinical practice guidelines for diagnos	Bol. Med. Hosp. Infa	No specific focus on specialists or the professions
GCLWCAFF	2022	Mayra, Kaveri; Mat	Why do some health care providers disr	Women and Birth	No specific focus on specialists or the professions
JEN2HXWU	2021	Mazzucchi, Eduard	The impact of COVID-19 in medical pra	Int. Braz J Urol	Other
M2MMXLLC	2013	Mbindyo, Patrick; B	The role of Clinical Officers in the Kenya	Hum. Resour. Healt	No specific focus on specialists or the professions
TDAXC243	2019	Mburu, Joy; Odame	Sickle cell disease: Reducing the global	Int. J. Lab. Hematol	No specific focus on specialists or the professions
TKZLF6QE	2008	McCaffery, Kirsten	Monitoring from the Patient's Perspective: The Social and Ps		Other
3AFKY4I6	2020	McCarthy, Matthew	Harnessing the potential of CRISPR-bas	Expert Rev. Anti. Inf	No specific focus on specialists or the professions
K7VXI8BJ	2021	McCauley, Linda; F	Implementing high-quality primary care: Rebuilding the four		Other
TUMBU5PE	2021	McCauley, Mary; R	Experiences and impact of internationa	BMJ Open	No info on specialists' contribution to health systems/population health, or their governance
2ZXFHN3F	2020	McClaren, Belinda	Preparing Medical Specialists for Geno	Front. Genet.	Not on LMICs
BWFSMKK5	2017	McCracken, Kevin;	Global health: An introduction to current and future trends:		Other
VNPDKHZQ	2011	McCullough, Jeffre	Transfusion Medicine: Third Edition		Other
QITRHZGD	2018	McKenna, M; Chen	Accuracy of trained rural ophthalmolog	Br. J. Ophthalmol.	
Y29PHWMI	2023	McNeil, Michael J;	Ideal vs Actual Timing of Palliative Care	JAMA Netw. open	No specific focus on specialists or the professions
QEC6G7CY	2023	McNeil, Michael J;	Physician Perceptions of and Barriers to	JCO Glob. Oncol.	No specific focus on specialists or the professions
UL5VA63F	2018	McVety, Amanda K	The Rinderpest Campaigns: A Virus, Its Vaccines, and Globa		Other
73KDMHNW	2015	Meara, John G; Lea	Global Surgery 2030: Evidence and solu	Lancet	
2LDD398F	2006	Mechanic, David	The truth about health care: Why reform is not working in An		Other
ZXL3WFTW	2020	Mechanick, Jeffrey	Clinical practice guidelines for the peri	Surg. Obes. Relat. D	No specific focus on specialists or the professions
QD467MYQ	2020	Mechanick, Jeffrey	Clinical Practice Guidelines for the Per	Obesity	No specific focus on specialists or the professions
VMDRVG46	2020	Medina-Lara, Anto	Cancer diagnostic tools to aid decision	Health Technol. Ass	No specific focus on specialists or the professions
2MAL7WWC	2022	Mehta, Aashna; Ch	Embracing robotic surgery in low- and n	Ann. Med. Surg.	No specific focus on specialists or the professions
Q7VIJRVF	2022	Mehta, Jodhbir S; C	Diagnosis, Management, and Treatmen	Front. Med.	No specific focus on specialists or the professions
TTCHE67N	2022	Mei, Fengyao; Li, Jia	Preference of Orthopedic Practitioners	Orthop. Surg.	No info on specialists' contribution to health systems/population health, or their governance
M7T2CX2B	2007	Meiser, Bettina; Ga	International perspectives on genetic c	Breast Dis.	No specific focus on specialists or the professions
I4WSLTEB	2019	Mekoguem, Carine	Madurella mycetomatis infection of the	J. Med. Case Rep.	No specific focus on specialists or the professions
2ZIIQ9YV	2020	Melchert, Timothy	Foundations of Health Service Psychology: An Evidence-Bas		Other
XKI9GENU	2012	Meleis, Afaf Ibrahim	Theoretical nursing: Development and progress: Fifth editio		Other
ISK4AY7L	2011	Meleis, Afaf; Birch,	Women's health and the world's cities		Other
P9EBH3XD	2013	Meliala, Andreasta	Addressing the unequal geographic dist	Soc. Sci. & Med.	
DZG8ILL2	2012	Melien, Øyvind; Ao	Report from subgroup education, traini	Japanese Pharmaco	No specific focus on specialists or the professions
F23VJYCG	2016	Meloni, Maurizio	Political biology: Science and social values in human hered		Other
YMSFFHTN	2024	Memba, Robert; Pu	The Use of a Theory of Change Model to	Ann. Glob. Heal.	Other
UPVZDSG7	2023	Mensah, George A;	Global Burden of Cardiovascular Disea	J. Am. Coll. Cardiol.	No specific focus on specialists or the professions
3Y3C5SWR	2021	Menz, Bradley D; C	Surgical antibiotic prophylaxis in an era	Infect. Drug Resist.	No specific focus on specialists or the professions
QKFQ348U	2019	Merriel, Abi; Ficqu	The effects of interactive training of hea	Cochrane Databas	No specific focus on specialists or the professions
7U9FQWMR	2020	Metogo, Junnette A	Anaesthesiologist-intensivist physician	Pan Afr. Med. J.	
63KCB3JZ	2013	Meurling, Lisbet; H	Systematic simulation-based team trai	BMJ Qual. Saf.	Not on LMICs
QLJMAIZW	2020	Meyer, Sarah R; Re	Strengthening health systems response	Pilot Feasibility Stu	No specific focus on specialists or the professions
GJEFY47N	2006	Mhando, S; Lyamuy	Challenges in developing paediatric su	Pediatr. Surg. Int.	Other
RN2MH9RR	2019	Mianda, Solange; V	Enablers and barriers to clinical leader	BMJ Lead.	No specific focus on specialists or the professions

Appendix 4

Table S4: List of papers excluded, with reason

B8SR9WTD	2020	Michel-Schuldt, Mi	The provision of midwife-led care in low	Midwifery	No specific focus on specialists or the professions
NZDS5JY4	2022	Mihretie, Getasew	Factors associated with discontinuatio	BMJ Open	No specific focus on specialists or the professions
PDBCMI3M	2023	Milia, Matías Feder	The transformation of medical research	Quant. Sci. Stud.	No specific focus on specialists or the professions
S9WG7M7F	2016	Miller, Suellen; Aba	Beyond too little, too late and too much	Lancet	No specific focus on specialists or the professions
TZ93Q8DQ	2024	Miller, Victoria; Jer	Associations of the glycaemic index and	lancet. Diabetes En	No specific focus on specialists or the professions
KXD75M54	2013	Milner, Kate M; Dul	Reducing newborn mortality in the Asia	J. Paediatr. Child H	No specific focus on specialists or the professions
U57WLGRN	2018	Mink, Richard; Schw	Creating the Subspecialty Pediatrics Investigator Network.		Other
VYUCKZT7	2018	Miotto, Bruno Alon	Physician's sociodemographic profile a	BMC Health Services Research	
55WZ55GK	2017	Miseda, Mumbo Ha	The implication of the shortage of healt	Hum. Resour. Health	
XDZYTJBF	2021	Mishra, Arima; Elia	A draconian law: Examining the navigat	J. Health Polit. Policy Law	
XJ3MUI4K	2023	Misra, Shivani; Agu	The case for precision medicine in the p	lancet. Diabetes En	No specific focus on specialists or the professions
L4GXDFVA	2021	Misran, Rohmah Na	The Role of Religiosity to Address the M	Pertanika J. Soc. Sc	No specific focus on specialists or the professions
ME3NVCPR	2020	Mitchell, Ian; Guicl	Ethics in Pediatrics: Achieving Excellence When Helping Ch		Other
3MVMDZ8E	2015	Mock, Charles N; D	Essential surgery: Key messages from D	Lancet	
8B7AU9IN	2017	Moghri, Javad; Rasl	Implications of dual practice among he	Iran. J. Public Health	
6FBU4UR9	2022	Mohamed, Mariam	Knowledge, Attitude, and Practice of Ce	Matern. Child Healt	Not on LMICs
GZABUTF5	2020	Mohammadibakhsl	Family physician model in the health sy	J. Educ. Health Pror	No specific focus on specialists or the professions
EWXJR3SD	2022	Mohammed, Elijah	Knowledge, causes, and experience of i	BMC Health Serv. R	No specific focus on specialists or the professions
CZ63QDX8	2020	Mohammed, Sawsa	Fruits	Adv. Neurobiol.	No specific focus on specialists or the professions
7KQGM4I2	2023	Mohebbi, Mohamm	Attitudes of medical students toward ps	Front. Psychiatry	Other
SGN2PRPI	2021	Mohsin, Shamaila;	Accuracy of community informant led d	Int. J. Environ. Res.	No specific focus on specialists or the professions
DXTZ84MN	2022	Moini, Jahangir; Av	Global epidemiology of cancer: Diagnosis and treatment		Other
NDWMSZ2Y	2024	Moja, Lorenzo; Zan	WHO's essential medicines and AWaRe	Clin. Microbiol. Infe	No specific focus on specialists or the professions
2XPWMTFC	2016	Mokdad, Ali H; Ford	Global burden of diseases, injuries, and	Lancet (London, Eng	No specific focus on specialists or the professions
AK89RF7P	2019	Mokitimi, Stella; Jo	Child and Adolescent Mental Health Se	Front. psychiatry	No specific focus on specialists or the professions
X3LIJPJU	2023	Moloi, Hlengiwe; D	Healthcare workers' perceptions and e	Cochrane database	No specific focus on specialists or the professions
H9S6JPBQ	2024	Moloney, Elizabeth	Core requirements of frailty screening i	Age Ageing	No specific focus on specialists or the professions
66JIR8NU	2016	Molyneux, Sassy; S	Public/community engagement in healt	Heal. Res. policy Sy	No specific focus on specialists or the professions
XDTZIQK2	2012	Monnais, Laurence	Global movements, local concerns: Medicine and health in		Other
5FHJ7LMB	2017	Monrouxe, Lynn V;	Healthcare professionalism: Improving practice through ref		Other
JQYJ4Y2Z	2007	Montbriand, Murie	Positive findings about herbs and natural products action or		Other
VVYE5K7Q	2023	Montesanti, Stepha	Exploring Factors Shaping Primary Heal	J. Fam. Violence	No specific focus on specialists or the professions
R7SHHCE7	2014	Montgomery, Ann L	The effect of health-facility admission a	PLoS One	No specific focus on specialists or the professions
JHMHI3R9	2020	Montgomery, Erwin	The Ethics of Everyday Medicine: Explorations of Justice		Other
Z6YHCT2I	2013	Montgomery, Mark	Cities transformed: Demographic change and its implicatio		Other
XXAD3YYQ	2023	Moodley, Saiendhr	Knowledge, confidence, and practices (	South African J. Psy	No specific focus on specialists or the professions
85CA3VK2	2022	Moodley, Saiendhr	Enabling mental health task-sharing: a (	BMC Med. Educ.	No specific focus on specialists or the professions
B7D3D7JE	2024	Moodley, Saiendhr	Developing mental health curricula and	BMC Med. Educ.	No specific focus on specialists or the professions
KCI8EJR9	2024	Moore, Kristine A; C	Lassa fever research priorities: towards	Lancet. Infect. Dis.	No specific focus on specialists or the professions
SCL4QGZD	2008	Moosa, M Y H; Jeen	Community psychiatry: An audit of the s	South African J. Psychiatry	
KGECA9ZP	2020	Morales-Morales, C	Recomendaciones para procedimiento	Cir. Cir.	No specific focus on specialists or the professions
5VU3ZAQV	2015	Moran, N F; Naidoo	Reducing maternal mortality on a coun	Best Pract. Res. Cli	No specific focus on specialists or the professions
4KPYFTYQ	2016	Morandi, Federica	Managerial solutions to hospitals' challenges		Other
ADU7YMZN	2023	Morawska, Lidia; B	Coronavirus Disease 2019 and Airborne	Clin. Infect. Dis. an	No specific focus on specialists or the professions
AS2GC59B	2017	Morency-Potvin, Ph	Antimicrobial stewardship: How the mi	Clin. Microbiol. Rev	No specific focus on specialists or the professions
KQV4WPLM	2018	Morgan, Melissa C;	Clinical cascades as a novel way to ass	PLoS One	No specific focus on specialists or the professions
BGWQ9LFX	2015	Morgano, Gian Pao	Guidelines 2.0: Systematic developmen	Recenti Prog. Med.	No specific focus on specialists or the professions
XHSXJJTR	2003	Moroz, A; Schoeb, V	Convalescence care for seniors in lowe	Top. Geriatr. Rehab	Not on LMICs
TSBNWMQ7	2009	Morrell, C J; Warne	Psychological interventions for postnat	Health Technol. Ass	No specific focus on specialists or the professions
CNVDCGWE	2021	Morris, Alan H; Stag	Enabling a learning healthcare system v	J. Am. Med. Informa	No specific focus on specialists or the professions
DXM7XVN4	2021	Morse, Michelle; Sp	Revitalizing Graduate Medical Educati	Acad. Med.	No specific focus on specialists or the professions
V58WR2IN	2021	Morse, R S; Lambde	A mobile app to improve symptom contr	JMIR Cancer	No info on specialists' contribution to health systems/population health, or their governance
NVH99HF2	2020	Mosca, Gloria; Cap	Myanmar health professionals' educati	Acta Biomed.	No specific focus on specialists or the professions
9P485RV6	2024	Moss, Esther; Taylo	British Gynaecological Cancer Society	Eur. J. Obstet. Gyne	No specific focus on specialists or the professions
FCVHN3QH	2024	Mostkowska, Agata	Repurposing of rituximab biosimilars to	FASEB J.	No specific focus on specialists or the professions
M3VNL8H8	2022	Moucheraud, Corri	Trust, Care Avoidance, and Care Experi	Heal. Syst. Reform	No specific focus on specialists or the professions
ARJHIJBX	2019	Moudi, Zahra; Taba	Empowering midwives to manage postp	East. Mediterr. Hea	No specific focus on specialists or the professions
A7MBCQFT	2020	Mousa, Ola; Shafi, S	Young educated adult awareness regar	Int. J. Adv. Appl. Sci.	No specific focus on specialists or the professions
PZCDUQ87	2021	Mouton, Johannes	Improving anticoagulation in sub-Sahar	Br. J. Clin. Pharmac	No specific focus on specialists or the professions
5VXVKU6F	2014	Moyad, Mark A	Complementary & alternative medicine for prostate and uro		Other
MKNJ5NW6	2016	Moyad, Mark A	Integrative medicine for breast cancer: An evidence-based		Other
5S5DUFZE	2008	Moyano, Jairo; Zam	Palliative sedation in Latin America: su	Support. CARE CAN	No specific focus on specialists or the professions
FQ8WV2QR	2014	Mpemba, Faraja; K	Towards 2015: post-partum haemorrha	J. Clin. Nurs.	No specific focus on specialists or the professions
3QQNCQWL	2016	Mpofu, Charles	International medical experiences outbound New Zealand:		Other
I9LL5H38	2018	Mrklas, Kelly J; Mac	Barriers, supports, and effective interve	Syst. Rev.	No specific focus on specialists or the professions
MYTIEWPX	2021	Mselle, Lilian; Sirili	Understanding barriers to implementin	PLoS One	No specific focus on specialists or the professions
ZBQQTY2R	2014	Mueller, Brigitta U	Quality and safety in pediatric hematol	Pediatr. Blood Canc	Not on LMICs
FHN2J2SZ	2024	Mukherjee, Ankita;	Determinants of stillbirths in sub-Sahar	BJOG An Int. J. Obst	No specific focus on specialists or the professions
Y8A39BNT	2010	Mulder, C J J; Puri, A	Gastroenterology training in private hos	World J. Gastroenterol.	
RQWS7AH5	2021	Muli, Elizabeth; Wa	Leveraging technology for health servic	J. Glob. Health	No specific focus on specialists or the professions
6BBVTSG7	2007	Mullan, Fitzhugh; F	Non-physician clinicians in 47 sub-Sah	Lancet (London, Eng	No specific focus on specialists or the professions
Y4BLYS5M	2016	Muller, Elmi	Transplantation in Africa - an overview.	Clin. Nephrol.	No specific focus on specialists or the professions
IJ2WR5VX	2017	Müller, Susan; Nels	Diagnostic Pathology: Head & Neck		Other
IPLS843F	2017	Mulwafu, Wakisa; K	Feasibility and acceptability of training	BMJ Open	No specific focus on specialists or the professions
57YBTVM6	2017	Munabi-Babigumir	Factors that influence the provision of i	Cochrane Databas	No specific focus on specialists or the professions
RV3TQIJI	2019	Munday, Daniel; Ka	Integrated management of non-commu	BMJ Support. Pallia	No specific focus on specialists or the professions
IZSQ4T7Z	2017	Munns, Christina; E	Privacy and Healthcare Data: 'Choice of Control' to 'Choice		Other
TDYIS7VD	2024	Muñoz-Villaverde,	Impact of telenurse-led intervention in	BMC Nurs.	No specific focus on specialists or the professions
EZ3Q7P8W	2020	Murphy, Adrianna;	The household economic burden of non	BMJ Glob. Heal.	No specific focus on specialists or the professions
RM6FRNXP	2012	Murthy, Lakshmi; S	Interventions to improve the use of syst	COCHRANE DATAB	No specific focus on specialists or the professions
VXF9ETXL	2021	Muruga, K; Vasiljev	PHYSICIANS' DUAL PRACTICE: A THEOR	Cent. Eur. Bus. Rev.	Not on LMICs
25EWDD2B	2021	Musarandega, Reu	Causes of maternal mortality in SubSah	J. Glob. Health	No specific focus on specialists or the professions
8ZW23JPG	2022	Musekiwa, Alfred; N	Mapping Evidence on the Burden of Bre	Front. Public Heal.	No specific focus on specialists or the professions
LQEWVVTT	2021	Mwapasa, Gerald;	Evaluation of a Managed Surgical Consu	World J. Surg.	
Y5ZSI3X5	2021	Mwita, Julius C; Og	Key issues surrounding appropriate ant	Int. J. Gen. Med.	No specific focus on specialists or the professions
NK3GGPA8	2024	Mwogosi, Augustino	Revolutionizing primary health care in T	J. Sci. Technol. Polic	No specific focus on specialists or the professions
I3MT2ASV	2023	Myerholtz, Linda; S	Integrated Behavioral Health Care		Other
NS2JCK2N	2024	Mysler, Eduardo; M	Opportunities and challenges of lupus c	Mod. Rheumatol.	No specific focus on specialists or the professions
4A8Z6QRH	2023	Naanyu, Violet; Nju	Community engagement to inform deve	BMC Health Serv. R	No specific focus on specialists or the professions
XTJMZW2V	2018	Nababan, Herfina Y	Trends and inequities in use of materna	Int. J. Womens. Hea	No specific focus on specialists or the professions
VYDEY2N5	2017	Naburi, Helga; Ekst	The potential of task-shifting in scaling u	Hum. Resour. Healt	No specific focus on specialists or the professions
99DZUC4F	2020	Nachega, Jean B; M	Responding to the Challenge of the Dua	Am. J. Trop. Med. Hy	No specific focus on specialists or the professions
MLJ59QL3	2022	Nadarajah, Anurad	House officers' specialist career choice	BMC Med. Educ.	Other
B3BDLTRQ	2021	Naehrig, Diana; Sc	Effect of interventions for the well-bein	BMJ Open	No specific focus on specialists or the professions
A9IHSV4C	2011	Nagamatsu, Soichi	The earthquake and tsunami–observati	Crit. Care	No specific focus on specialists or the professions
8G7Z3CSW	2024	Naghavi, Mohsen; C	Global burden of 288 causes of death ar	Lancet	No specific focus on specialists or the professions
P5TX34WH	2022	Naidoo, Kimesh Log	Insights of South African medical intern	Educ. Health (Abing	No specific focus on specialists or the professions
4XDL5BP3	2013	Naidoo, Mergan	Role of the family physician in reducing	Obstet. Gynaecol. F	No specific focus on specialists or the professions
RGU2QIV2	2020	Naidoo, T; Tomita, A	Burnout, anxiety and depression risk in	PLoS One	No specific focus on specialists or the professions

Appendix 4

Table S4: List of papers excluded, with reason

4DLRJPIW	2020	Naidu, Priyanka; Fa	The role of the University of Cape Town,	Am. J. Surg.	
RJG948SE	2014	Nair, Manisha; Yos	Facilitators and barriers to quality of ca	BMJ Open	No specific focus on specialists or the professions
MGF9BY8W	2023	Namagembe, Imelc	Barriers and facilitators to maternal de	Open Res. Africa	No specific focus on specialists or the professions
I7EWNVIY	2022	Namagembe, Imelc	Preventing death following unsafe abort	AJOG Glob. reports	No specific focus on specialists or the professions
Q3THHNXX	2021	Nancarrow, Susan;	The allied health professions: A sociological perspective		Other
MD7LXSKB	2020	Nanda, Sapna	Evaluation and management of high-risk pregnancies: Emer		Other
F5NMIW8X	2022	Nasa, Prashant; Az	Infection control in the intensive care u	Lancet. Infect. Dis.	Not on LMICs
UKH9UZQH	2014	Nash, Meredith	Reframing Reproduction: Conceiving G	Genders Sex. Soc. S	Other
XU4AHI35	2018	Natala, Nakita; Ow	Building Child and Adolescent Psychiat	Curr. Psychiatry Rep.	
WPEWLW4P	2023	Nath, Anita; Mathu	An assessment of childhood cancer car	Lancet Reg. Heal. S	No specific focus on specialists or the professions
TPRR2J73	2007	Nathanson, Consta	Disease prevention as social change: The state, society, an		Other
GT9ETREZ	2022	Nayyar, Abhishek S	Indian rural lands and shortages of heal	J. Datta Meghe Inst.	No specific focus on specialists or the professions
HX9X5EH7	2020	Ndetei, David; Nya	Psychiatric education in Africa-Kenyan	Int. Rev. Psychiatry	Other
97MUNDFI	2012	Negin, J; Martiniuk,	Frequency, cost and impact of inter-isl	Rural Remote Healt	No specific focus on specialists or the professions
ERC7YV47	2009	Nel Cordoba, R; Fe	The Latin American Psychiatrist: profile	ACTAS Esp. Psiquiat	Other
MTGMRWWK	2013	Nelson, Erin	Law, policy and reproductive autonomy		Other
HZRASLVI	2016	Nelson, Sarah	Tackling child sexual abuse: Radical approaches to prevent		Other
QHP9R69M	2022	Nepomuceno, Thya	The Core of Healthcare Efficiency: A Co	Healthc.	No specific focus on specialists or the professions
PRIR4R4Y	2018	Neville, Brad W; Da	Color Atlas of Oral and Maxillofacial Diseases		Other
H6DQDQWP	2010	Newman, D E; Shap	Obstacles faced by general practitione	Rural Remote Healt	No specific focus on specialists or the professions
E9ICGVTS	2013	Newman, M Sophia	Review of studies of mental health in Ba	Int. J. Ment. Health	No specific focus on specialists or the professions
DURCDLJI	2024	Ngarmbatedjimal,	Refugee women's and providers' percep	BMC Pregnancy Ch	No specific focus on specialists or the professions
NG3PV888	2022	Ngaruiya, Christine	When women win, we all win—Call for a	FASEB BioAdvances	No specific focus on specialists or the professions
3WQ38AQY	2023	Nguyen, Thanh N; C	Global Impact of the COVID-19 Pandem	Neurology	No specific focus on specialists or the professions
J8X9KBSA	2020	Nhamo, Godwell; D	Counting the cost of COVID-19 on the global tourism indust		Other
TJ3H2B7N	2014	Nhavoto, José Antó	Mobile technologies and geographic inf	JMIR mHealth uHea	No specific focus on specialists or the professions
BU7LDGU9	2020	Ni, Xiaojia; Lin, Hac	Evidence-based practice guideline on i	J. Evid. Based. Med.	No specific focus on specialists or the professions
E8242TXI	2017	Nicholls, David A	The end of physiotherapy		Other
TIZTPM27	2013	Nicolaides, Andrew	Prevention and treatment of venous thr	Clin. Appl. Thromb.	No specific focus on specialists or the professions
84BX6275	2018	Nieder, Rolf; Benbi	Soil components and human health		Other
AIUN5TSN	2023	Niederberger, Marl	Delphi Methods in Health Promotion. Results of a Systemat		Other
IY4E85VT	2021	Nigam, Shalu	Domestic violence law in India: Myth and misogyny		Other
TXSFBG9H	2023	Nigatu, Araya Mesf	Medical imaging consultation practices	BMC Health Serv. Res.	
WNJ2RDMK	2015	Nigenda, Gustavo;	Projections of specialist physicians in M	Human Resources for Health	
C9UTREJ5	2011	Nightingale, Kather	Laos builds specialty training system th	Lancet	
LSQJ2GQC	2021	Nikoloski, Zlatko; A	The impact of primary health care and s	Soc. Sci. & Med.	
YPAAAESK	2019	Nilan, Kapka; McKe	Prevalence of tobacco use in healthcar	PLoS One	No specific focus on specialists or the professions
GEJPYQGH	2012	Nissan, Ephraim	Computer Applications for Handling Leg	Law, Gov. Technol. S	Other
7XJKASHN	2021	Nkansah, Reginald	Racial disparities in vascular surgery: A	J. Vasc. Surg.	Not on LMICs
7NNVEU65	2019	Nkwanyana, Ntoml	Are there decision support tools that m	BMC Health Serv. R	No specific focus on specialists or the professions
FMAMWPLA	2021	Nolan, Samantha J	Social networking sites: Can midwives a	Int. J. Nurs. Pract.	No specific focus on specialists or the professions
TT7G79GP	2013	Noormahomed, Em	Strengthening research capacity throug	Hum. Resour. Health	
ZP3U9CDC	2024	Noreen, Humera; Y	Factors leading to lapses in professiona	BMC Med. Educ.	No specific focus on specialists or the professions
BY68NVWW	2011	Notrica, Michelle F	Rwandan surgical and anesthesia infras	World J. Surg.	Other
JRPRMHSW	2014	Noubiap, Jean Jacq	Local innovation for improving primary	Cardiovasc. Diagn.	No info on specialists' contribution to health systems/population health, or their governance
RCNJC94A	2018	Novoa, Monica; Ba	Interventions for infantile haemangiom	Cochrane database	No specific focus on specialists or the professions
PYXHCKJQ	2017	Ntuli, Samuel T; Ma	Geographical distribution and profile of	African J. Prim. Hea	No specific focus on specialists or the professions
7U4L3Y9L	2023	Nuampa, Sasitara;	"Because it eases my Childbirth Plan":	BMC Pregnancy Ch	No specific focus on specialists or the professions
ADY4V6SR	2016	Nwaopara, Uche Ac	Doctor to patient RAtio and infrastructu	African J. Psychiatry	Other
AYIFI7EX	2024	Nyadanu, Sylvester	Maternal exposure to ambient air temp	Sci. Total Environ.	No specific focus on specialists or the professions
JZ4QJJMS	2016	Nyamtema, A; Mwa	Increasing the availability and quality o	BJOG An Int. J. Obst	No specific focus on specialists or the professions
TL4WUKHD	2022	Nyawira, Lizah; Tso	Management of human resources for he	BMC Health Serv. Res.	
UYCN9RQ2	2020	Nydoo, Puvashnee;	The second victim phenomenon in heal	Scand. J. Public Hea	Not on LMICs
PMDA6BLN	2020	Nyondo-Mipando,	"It brought hope and peace in my heart:	BMC Pediatr.	No specific focus on specialists or the professions
DXEXKEUU	2004	O'Brien, Kylie	Complementary and alternative medic	Clin. Exp. Optom.	Not on LMICs
3VGKX4CD	2004	O'Brien, Sarah Har	Identification, evaluation, and manage	Pediatrics	Not on LMICs
E8ZLPA4Q	2016	O'Callaghan, Clare	"I might not have cancer if you didn't me	Support. Care Canc	Not on LMICs
ZKQ5L4HW	2017	O'Sullivan, Belinda	Reasons why specialist doctors underta	Hum. Resour. Healt	Not on LMICs
ZFHZ4RGY	2023	O'Sullivan, Declan	Can social prescribing put the 'social' ir	Musculoskeletal Ca	No specific focus on specialists or the professions
RE9BMIS2	2020	Oberai, Himani; Ga	Dealing with workplace adversity in em	Financ. India	No specific focus on specialists or the professions
YPVZVE2U	2015	Obermeyer, Ziad; A	Emergency care in 59 low- and middle-i	Bull. World Health	No specific focus on specialists or the professions
TDWCXZHA	2019	Oboirien, Kafayat;	Can institutional entrepreneurship stre	Health Policy Plan.	
P2KAP4CQ	2015	Obreli-Neto, Paulo	Economic evaluation of a pharmaceutic	J. Manag. Care Phar	No specific focus on specialists or the professions
D6Z7EAH4	2017	Obua, Thomas Ocw	Factors associated with occupancy of p	Hum. Resour. Healt	No specific focus on specialists or the professions
3R2C2P2Y	2017	Odonkor, Charles A	Connecting the Dots: A Comparative G	PAIN Med.	
AUL35C7I	2017	Ojelade, Olubunmi	The communication and emotional sup	Int. J. Gynecol. Obs	No specific focus on specialists or the professions
7D93DMJI	2024	Okai, Emmanuel; F	Neonatal transport practices and effec	Heal. Sci. reports	No specific focus on specialists or the professions
X8I3LD5E	2017	Okeahialam, Basil	Multidrug intolerance in the treatment	Ther. Adv. drug Saf.	No specific focus on specialists or the professions
3ISGFS6R	2021	Okedo-Alex, Ijeoma	Does disrespect and abuse during child	BMC Pregnancy Ch	No specific focus on specialists or the professions
B4DIFZY9	2021	Okedo-Alex, Ijeoma	Does it happen and why? Lived and shar	Women and Birth	No specific focus on specialists or the professions
8GZCRT53	2005	Okome-Nkoumou,	Delay between first HIV-related sympto	HIV Clin. Trials	No specific focus on specialists or the professions
WURG49RM	2022	Okonofua, Friday E	Assessment of Interventions in Primary	SAGE Open	No specific focus on specialists or the professions
LDZT4WZ8	2018	Okonofua, Friday; N	Association of the client-provider ratio	Reprod. Health	No specific focus on specialists or the professions
GYZ9M9IB	2021	Okoro, Roland Nna	Clinical pharmacist as a member of the	JACCP J. Am. Coll. C	No specific focus on specialists or the professions
AN62R6WY	2009	Olafsdottir, Sigrun;	Drawing the Line: The Cultural Cartogra	J. Health Soc. Beha	No specific focus on specialists or the professions
3U6HFSX7	2023	Olatunji, Gbolahan	Navigating unique challenges and adva	Ann. Med. Surg.	No specific focus on specialists or the professions
F8KEK5LR	2020	Oldstone, Michael	Viruses, plagues, and history: Past, present, and future		Other
8GBP5HTH	2017	Oleson, Christina V	Osteoporosis rehabilitation: A practical approach		Other
ZYB8PCRK	2024	Olivares-Tirado, Pe	Waste in health care spending: A scopir	Int. J. Healthc. Man	No specific focus on specialists or the professions
JRWEJMPQ	2024	Olive, Mattia Vince	Digitally-mediated coordination in hea	BMC Health Serv. R	Not on LMICs
6J849V6M	2023	Olmos, Brenda; Na	Theory Integration for Examining Health	West. J. Nurs. Res.	Not on LMICs
MV7GJIDJ	2012	Olson, Robert A; Bo	Oncologists' view of informed consent a	Radiother. Oncol. J	Not on LMICs
6NVNHK9M	2019	Oluwasanu, Mojiso	Causes and remedies for low research	BMC Res. Notes	No specific focus on specialists or the professions
64I8C4Y8	2009	Oman, Kimberly M;	Specialist training in Fiji: Why do gradua	Hum. Resour. Health	
W5MF958M	2012	Oman, Kimberly; Ro	Scaling up specialist training in develop	Hum. Resour. Health	
L9IV2529	2014	Omaswa, F G	The contribution of the medical educat	Acad. Med.	No specific focus on specialists or the professions
9K3K5UWW	2014	Omer, Khalid; Afi, N	Seeking evidence to support efforts to i	BMC Pregnancy Ch	No specific focus on specialists or the professions
DE5W7KSD	2014	Omonuwa, Toma; S	Prenatal maternal-fetal imaging for global health rad		No specific focus on specialists or the professions
W83UDRPV	2014	Onah, Michael N; C	Out-of-pocket payments, health care a	PLoS One	No specific focus on specialists or the professions
9H3UNL75	2022	Onambele, Luc; Or	Maternal Mortality in Africa: Regional T	Int. J. Environ. Res.	No specific focus on specialists or the professions
D8PRHN4Z	2023	Ong, Kanyin Liane;	Global, regional, and national burden o	Lancet	No specific focus on specialists or the professions
E9N9GBLA	2023	Oniani, David; Hils	Adopting and expanding ethical princip	npj Digit. Med.	No specific focus on specialists or the professions
3FW7RXDF	2022	Onyeodi, Ifeanyich	The path to becoming a clinical or radia	Ecancermedicalsci	No specific focus on specialists or the professions
MKIRBF6Z	2019	Oppy, Graham	A companion to atheism and philosophy		No specific focus on specialists or the professions
R5YXXT4R	2018	Oquendo, Maria A;	Building capacity for global mental hea	The Lancet Psychiat	No specific focus on specialists or the professions
CCAS3NSK	2023	Ortega-Villa, Ana M	Evaluating Demographic Representatic	Open forum Infect.	No specific focus on specialists or the professions
56ZFSUUN	2024	Oskvarek, Jonathar	Beyond the Four Walls: The American C	Ann. Emerg. Med.	Not on LMICs
PU8AWVUM	2019	Osman, Mohamed	Barriers and facilitators for implementa	BMJ Glob. Heal.	
IHFD7EF6	2013	Ost, David E; Yeung	Clinical and organizational factors in th	Chest	No specific focus on specialists or the professions

Appendix 4

Table S4: List of papers excluded, with reason

HZ53XSBR	2020	Ost, Suzanne; Bigg	Exploitation, ethics and law: Violating the ethos of the docto		No specific focus on specialists or the professions
4E37MIU5	2020	Othman, Mohd Yus	Pediatric Solid Tumor Care and Multidis	JCO Glob. Oncol.	
SSH7B4LR	2020	Ouchicha, Chaima	CVDNet: A novel deep learning archited	CHAOS SOLITONS &	No specific focus on specialists or the professions
TPEM4J3H	2022	Owen, Medge D; Is	Use of WhatsApp messaging technology	PLoS One	No specific focus on specialists or the professions
MS292PEE	2022	Owolabi, Eyitayo O	Telemedicine in Surgical Care in Low- a	World J. Surg.	No specific focus on specialists or the professions
39BYVQJB	2016	Owolabi, Mayowa;	Gaps in Hypertension Guidelines in Low	Hypertens. (Dallas,	No specific focus on specialists or the professions
6CS6CUV6	2022	Owusu-Agyemang,	Survey of Anesthesia, Sedation, and No	Cureus	No specific focus on specialists or the professions
N632F2FS	2023	Oxley, Samuel; We	Cost-Effectiveness of Genetic Testing Strategies for Breast		No specific focus on specialists or the professions
2A6MPN35	2013	Oyebode, Femi	Clinical errors and medical negligence	Med. Princ. Pract.	No specific focus on specialists or the professions
YJ544W4X	2008	Ozgediz, Doruk; Ga	The neglect of the global surgical workfo	World J. Surg.	
QWVXSSSN	2014	Özkaya, Esen; Yazg	Adverse cutaneous drug reactions to cardiovascular drugs		No specific focus on specialists or the professions
75YZKH7Q	2024	Özkaytan, Yasemin	We need a radical change to take place	Geriatr. Nurs. (Minn	Not on LMICs
97T5784P	2004	Ozuah, Philip O; Re	The role of telemedicine in the care of c	J. Telemed. Telecar	Not on LMICs
48SSVN33	2017	Paal, Piret; Bükki, J	"If I had stayed back home, I would not b	PLoS One	No specific focus on specialists or the professions
7DQ5K8I4	2012	Pacagnella, Rodolf	The role of delays in severe maternal m	Reprod. Health Mat	No specific focus on specialists or the professions
63B2HDDH	2017	Paez, Diana; Peix, A	Current status of nuclear cardiology pra	J. Nucl. Cardiol. Off	No specific focus on specialists or the professions
H498HUSV	2017	Pagalday-Olivares,	Exploring the feasibility of eHealth solu	BMC Med. Inform. D	No specific focus on specialists or the professions
BHATI2Z2	2011	Pagliaro, Louis A; Pa	Handbook of Child and Adolescent Drug and Substa		No specific focus on specialists or the professions
TVHCGMPJ	2022	Pai, Nagesh; Gupta	Indian psychiatrists in the Australian wo	Aust. N. Z. J. Psychia	No specific focus on specialists or the professions
5U9VG38V	2020	Palk, Andrea C; Ste	Ethical issues in global mental health		No specific focus on specialists or the professions
P74XH9VP	2021	Pallua, Johannes; S	Identification of Five Quality Needs for	Front. Med.	No specific focus on specialists or the professions
2NCDMTXX	2020	Pandian, Jeyaraj D;	Stroke systems of care in low-income a	Lancet	No info on specialists' contribution to health systems/population health, or their governance
W38P4VAD	2020	Pang, Jing; Sullivan	Familial Hypercholesterolaemia in 202	Hear. Lung Circ.	No specific focus on specialists or the professions
EVFG9YPS	2016	Pang, Wei Wei; Aris	Determinants of Breastfeeding Practice	Birth	No specific focus on specialists or the professions
MSIZ2I7C	2019	Pantoja, Tomas; Gr	Manually-generated reminders delivere	Cochrane Databas	No specific focus on specialists or the professions
BD8JAAWN	2018	Papadimos, Thoma	Ethics of outbreaks position statement.	Crit. Care Med.	No specific focus on specialists or the professions
85YSJIW6	2024	Papalois, Vassilios;	The role of health literacy in major healthcare crises		No specific focus on specialists or the professions
B5ER2XSR	2017	Papoutsi, Chrysant	Social and professional influences on a	J. Antimicrob. Chem	No specific focus on specialists or the professions
KVWX6TWY	2022	Parak, Yusuf; Davis	A 6-year audit of public-sector MR utilis	SA J. Radiol.	No specific focus on specialists or the professions
EWFCBY3M	2015	Park, Hee Sun; Lan	Diagnostic Ultrasound: Abdomen and Pelvis		No specific focus on specialists or the professions
7NMSZZD2	2018	Park, So Young; Phu	End-of-Life Care in ICUs in East Asia: A C	Crit. Care Med.	No specific focus on specialists or the professions
97J76U6X	2021	Parker, Anneka; Tek	Taking Paediatrics Abroad: Working wit	J. Paediatr. Child He	No info on specialists' contribution to health systems/population health, or their governance
YDWYDPUS	2013	Parker, Melissa J; M	Translating Resuscitation Guidelines in	PLoS One	No specific focus on specialists or the professions
9MD5L8YZ	2007	Parkhurst, Justin O	Life saving or money wasting?. Percepti	Health Policy (New	No info on specialists' contribution to health systems/population health, or their governance
S5WU3HXY	2019	Parks, Mitchell H; C	Altered emotional intelligence through	J. Health Care Poor	No specific focus on specialists or the professions
G6WVL9IF	2020	Parrington, John	Redesigning life: How genome editing will transform the wor		No specific focus on specialists or the professions
ILDRHUYD	2022	Parrish, Richard H;	Positive Patient Postoperative Outcom	J. Clin. Med.	No specific focus on specialists or the professions
VM3Q4UJI	2015	Parsa, Mojtaba; Ara	Informal Payments for Health Care in Ir	Iran. J. Public Healt	No specific focus on specialists or the professions
L488PBZF	2022	Pascucci, Tancredi	Systematic Review of Socio-Emotional	Front. Psychol.	No specific focus on specialists or the professions
HJ8XKLNY	2024	Pastor, Felipe Mart	Bone development in fetuses with intra	Bone	No specific focus on specialists or the professions
PGEX3YG2	2016	Patel, A A; Ciccone	A pediatric epilepsy diagnostic tool for	EPILEPSY & Behav.	No specific focus on specialists or the professions
79MPK2Y3	2020	Patel, Kepal N; Yip,	The American association of endocrine	Ann. Surg.	No specific focus on specialists or the professions
UL73F4X3	2024	Patel, Tushar N; Ch	Structure and Funding of Clinical Inform	Appl. Clin. Inform.	No specific focus on specialists or the professions
VQL3HQ2Z	2023	Patel, Vikram; Saxe	Transforming mental health systems glo	Lancet (London, Eng	No specific focus on specialists or the professions
9Z4P4LXM	2021	Patterson, Pandora	The Australian youth cancer service: de	Cancers (Basel).	Not on LMICs
YDSBYW2G	2020	Patterson, V	The development of a smartphone appli	Seizure	No specific focus on specialists or the professions
7SI5MEH5	2022	Patterson, Victor; L	Validation of an epilepsy management s	ACTA Epileptol.	No specific focus on specialists or the professions
4VBERBPJ	2016	Patton, George C; S	Our future: a Lancet commission on ado	Lancet	No specific focus on specialists or the professions
394LRT5X	2014	Paul, Mandira; Iyen	Simplified follow-up after medical abor	BMC Womens. Hea	No specific focus on specialists or the professions
QQ79I7J9	2013	Pearce, Elizabeth C	The usefulness of a yearly head and nec	Otolaryngol. neck S	No info on specialists' contribution to health systems/population health, or their governance
EW5G57AD	2008	Peate, Ian	Men's Health: The Practice Nurse's Handbook		No specific focus on specialists or the professions
2G3H4YP4	2021	Peckham, Allie; Lax	Putting the Patient First: A Scoping Revi	Healthc. Policy	No specific focus on specialists or the professions
K99EAWZM	2024	Peeler, Anna; Afola	Primary palliative care in low- and midd	Palliat. Med.	No specific focus on specialists or the professions
EB3TL5AH	2014	Peer, Nasheeta; Ke	Diabetes in the Africa Region: an updat	Diabetes Res. Clin.	No specific focus on specialists or the professions
T6J7QR9X	2016	Peersman, Wim; De	The contribution of primary care research to educati		No specific focus on specialists or the professions
268P924B	2011	Pei, Y Veronica; Xia	Emergency medicine in China: present	World J. Emerg. Med.	
DE8EGL2Y	2018	Peluso, Michael J; T	Building Health System Capacity throu	Ann. Glob. Heal.	
HLAECGUZ	2023	Penaloza, Maylin; S	Survivorship Care in Middle-Income Co	JCO Glob. Oncol.	No specific focus on specialists or the professions
FJLGXC3I	2022	Peng, Shuanglin; Ga	LncRNA-AK137033 inhibits the osteoge	Cell Prolif.	No specific focus on specialists or the professions
VW9NJ3P8	2023	Penman, Sarah V; B	Barriers to accessing and receiving ant	J. Adv. Nurs.	No specific focus on specialists or the professions
H6LPJCVP	2015	Pereira-Lima, K; Lo	Burnout, anxiety, depression, and socia	Psychol. Heal. & Me	No info on specialists' contribution to health systems/population health, or their governance
MZUAA228	2016	Perera, Antoinette	Primary care: Putting people first		No specific focus on specialists or the professions
62PJW3TC	2023	Periasamy, Mahali	Redefining venereology practice in Tam	Indian J. Sex. Transr	No info on specialists' contribution to health systems/population health, or their governance
2FHHFX2C	2017	Perkins, Barbara B	Cancer, radiation therapy, and the market		No specific focus on specialists or the professions
VQCUF36A	2024	Permashwar, B; Ma	An Internal Medicine Learning Collabor	CUREUS J. Med. Sci	No info on specialists' contribution to health systems/population health, or their governance
ZGA5EAQE	2019	Perreira, Tyrone A;	Physician engagement: A concept analy	J. Healthc. Leadersh.	
ZA2WNTEQ	2024	Perrone, Gennaro;	Goodbye Hartmann trial: a prospective	World J. Emerg. Sur	No specific focus on specialists or the professions
GY56WFEG	2020	Perrone, Gennaro;	Management of intra-abdominal-infect	Int. J. Infect. Dis.	No specific focus on specialists or the professions
7MKAUG6C	2012	Perrot, Serge; Choy	Survey of physician experiences and pe	BMC Health Serv. R	No info on specialists' contribution to health systems/population health, or their governance
R7RVXX6P	2023	Persaud, Nav; Sabi	Preventive care recommendations to p	C. Can. Med. Assoc	No specific focus on specialists or the professions
76CPR2BY	2024	Pestana, Joana; Fru	Heterogeneity in physician's job prefere	Soc. Sci. Med.	Not on LMICs
PST4IELE	2012	Peter, Greaves	Histopathology of Preclinical Toxicity Studies		No specific focus on specialists or the professions
Q792E79Z	2017	Péteri, Tamás; Varg	A survey on multimedia quality of exper	Lect. Notes Inst. Co	No specific focus on specialists or the professions
5KM4SY9A	2009	Peters, David H	Improving Health Service Delivery in Developing Countries:		No specific focus on specialists or the professions
QP7DY4M6	2007	Peters, George A; P	Medical error and patient safety: Human factors in medicin		No specific focus on specialists or the professions
TQKK7QY2	2022	Pfarrwaller, Eva; Vo	Students' intentions to practice primar	BMC Med. Educ.	No specific focus on specialists or the professions
NQQIZ7RR	2022	Pham, Sheila; Chur	A scoping review of gestational diabete	BMC Pregnancy Ch	No specific focus on specialists or the professions
28ZX66HS	2020	Philbin, Jesse; Soel	Health system capacity for post-aborti	Reprod. Health	No specific focus on specialists or the professions
77967GAK	2020	Phillips, Georgina;	Emergency care status, priorities and s	Lancet Reg. Heal. W	No specific focus on specialists or the professions
EYS3R4JX	2018	Phillips, Kari A; Sing	Humor During Clinical Practice: Analys	J. Am. Board Fam. M	No specific focus on specialists or the professions
VG63E2ZM	2021	Phua, Jason; Lim, C	The story of critical care in Asia: a narra	J. intensive care	No info on specialists' contribution to health systems/population health, or their governance
PKD9H4XV	2023	Phua, Teow J	Understanding human aging and the fun	Front. Aging	No specific focus on specialists or the professions
DI4I57UC	2016	Pietruck, C; Pollacl	The Malawian Bachelor of Science	Anasthesiol. & Intensivmed.	
U94SIGJA	2021	Pigoga, J L; Omer, Y	Derivation of a Contextually-Appropria	Ann. Glob. Heal.	No specific focus on specialists or the professions
CATFI5QP	2023	Piliuk, Konstantin; T	Artificial intelligence in emergency med	Int. J. Med. Inform.	No info on specialists' contribution to health systems/population health, or their governance
IVQ2YBTA	2013	Pirnazarova, G; Pai	Palliative Care in Kyrgyzstan Special Ch	J. Hosp. & Palliat. N	No info on specialists' contribution to health systems/population health, or their governance
Y7XSN7RE	2022	Piskorz, Daniel; Día	Blood pressure telemonitoring and tele	Blood Press.	No specific focus on specialists or the professions
V7UTEDP7	2019	Pittalis, Chiara; Bru	Evaluation of a surgical supervision mo	Pilot Feasibility Stud.	
C9GZDJ4V	2024	Pitzer, Stefan; Kuts	Barriers for Adult Patients to Access Pa	J. Pain Symptom Ma	No specific focus on specialists or the professions
7DR26P8M	2023	Pizzuti, Carol; Paln	Using eHealth Data to Inform CPD for M	J. Contin. Educ. Hea	No specific focus on specialists or the professions
TEYZWSAI	2018	Plant, Amelia; Bair	American College of Preventive Medici	Am. J. Prev. Med.	No specific focus on specialists or the professions
TYVNG93E	2020	Pokhrel, Nishan Ba	Depression, anxiety, and burnout amon	BMC Psychiatry	No specific focus on specialists or the professions
K2IN8EAY	2023	Poll-Hunter, Norma	Increasing the Representation of Black	Acad. Med.	Not on LMICs
CUCKUKMS	2020	Pollack, Todd M; Nh	Building HIV healthcare worker capacit	BMJ Glob. Heal.	No specific focus on specialists or the professions
BV6NPERA	2023	Pollard, Maria	Evidence-based Care for Breastfeeding Mothers: A Resourc		No specific focus on specialists or the professions
J57DJF9F	2018	Pols, Hans	Nurturing Indonesia: Medicine and Decolonisation in the D		No specific focus on specialists or the professions
P67KU9TW	2023	Pomerantz, Phyllis	Foreign Aid: Policy and Practice		No specific focus on specialists or the professions
AZK9HSGY	2006	Pond, Bob; McPake	The health migration crisis: the role of f	Lancet	No specific focus on specialists or the professions
JDPVHYEG	2007	Ponticelli, Claudio	Medical complications of kidney transplantation		No specific focus on specialists or the professions
XETGDZ5P	2015	Poore, Sophie; Fost	Planning and developing services for di	Int. J. Heal. policy M	No specific focus on specialists or the professions
YKUEG5DH	2018	Porter, D C; Bezuid	Surgical skills deficiencies and needs o	South African Med.	No info on specialists' contribution to health systems/population health, or their governance
BZHTB9A7	2024	Porterfield, Laura; Y	A community health worker led diabete	J. Diabetes Complic	No specific focus on specialists or the professions
VD5XX5E2	2014	Porth, Carol Mattso	Essentials of pathophysiology: Concepts of altered health s		No specific focus on specialists or the professions
9TW2UZ9G	2023	Portillo, Gilbert A C	Virtual avatars as a new tool for human	mHealth	No specific focus on specialists or the professions
6MJZIFB7	2023	Postmes, J J; Rolim	Respected physician in Syria, unemploy	Soc. Sci. & Med.	No specific focus on specialists or the professions
4Q54E2J3	2011	Pottie, Kevin; Gree	Evidence-based clinical guidelines for i	C. Can. Med. Assoc	No specific focus on specialists or the professions

Appendix 4

Table S4: List of papers excluded, with reason

Y2GK6H36	2013	Power, C; O'Conno	An evidence-based assessment of prim	Ir. J. Med. Sci.	No specific focus on specialists or the professions
QN23QI87	2017	Pozo-Martin, Franc	Health workforce metrics pre- and post	Hum. Resour. Healt	No specific focus on specialists or the professions
7958AJD7	2021	Pradhan, Nousheer	Quality of inpatient care of small and si	BMC Pediatr.	No specific focus on specialists or the professions
Z4T33UCW	2020	Prado-Galbarro, Fr	Satisfaction With Healthcare Services	Value Heal. Reg. iss	No specific focus on specialists or the professions
E8KMF6M7	2006	Prasetiyawan; Viora	Mental health model of care programm	Int. Rev. Psychiatry	No specific focus on specialists or the professions
UNM94I5Z	2010	Prata, Ndola; Passa	Maternal mortality in developing count	Women's Heal.	No specific focus on specialists or the professions
V5PCNEGE	2024	Prawiroharjo, Puko	Factors correlating to decisions for pre	HELIYON	No specific focus on specialists or the professions
J66KGF4T	2011	Pray, Walter Stever	Nonprescription product therapeutics: Second edition		No specific focus on specialists or the professions
65FQP5IA	2020	Preckel, Benedikt;	Ten years of the Helsinki Declaration or	Eur. J. Anaesthesiol	No specific focus on specialists or the professions
PKMR2U33	2012	Prescott, Julie; Bog	Gendered occupational differences in science, engineering		No specific focus on specialists or the professions
9F4KN76L	2012	Pretty, Jules	The pesticide detox: Towards a more sustainable agricultur		No specific focus on specialists or the professions
J6P6646I	2022	Price, Anna; de Bel	What is the volume, diversity and natur	Campbell Syst. Rev	No specific focus on specialists or the professions
SMPSPRX7	2015	Prinja, Shankar; Na	Costs, Effectiveness, and Cost-Effectiveness of Selected Su		No specific focus on specialists or the professions
K6D2KYNG	2021	Prystowsky, Michae	Prioritizing the Interview in Selecting Re	Acad. Pathol.	Not on LMICs
FGEWGMWL	2020	Pulok, Mohammad	Horizontal inequity in the utilisation of	Health Policy (New	Not on LMICs
8ZXQSX8X	2023	Purgato, Marianna;	Primary-level and community worker in	Cochrane Databas	No specific focus on specialists or the professions
KYC8WDMJ	2018	Putnam, Angelica R	Diagnostic Pathology: Pediatric Neoplasms		No specific focus on specialists or the professions
I77RAIPS	2017	Putranto, Rudi; Mu	Development and challenges of palliati	Biopsychosoc. Med	No specific focus on specialists or the professions
QAJ2ZVT5	2021	Qi, Miaojie; Cui, Jiy	Perceived factors influencing the public	J. Med. Internet Res	No specific focus on specialists or the professions
AI8UGP7A	2022	Qi, Xing; Chen, Dej	Long non-coding RNA PRNCR1 promote	Mol. Cell. Biochem	No specific focus on specialists or the professions
P8G3P3CA	2024	Qin, Rennie; Alayar	Colonisation and its aftermath: reimagi	BMJ Glob. Heal.	No specific focus on specialists or the professions
LHGCP45H	2023	Quarde, Akuffo	Endocrinology: Pathophysiology to therapy		No specific focus on specialists or the professions
SN6X42YV	2024	Quintao, V C; Conc	Pediatric anesthesiology in Brazil, Chile	Pediatr. Anesth.	
7WX72Y25	2024	Quyyumi, Arshed A;	Research Training in Medical Schools	Adv. Sci. Technol. In	No specific focus on specialists or the professions
G3W4RDEH	2008	Radecki, Linda; Ols	Reliability and validity of the children's	Pediatr. Asthma, All	No specific focus on specialists or the professions
8SFH72GW	2022	Radhakrishnan, N	Genesis, Pathophysiology and Management of Venous and L		No specific focus on specialists or the professions
QCPR7JX4	2019	Radovanovic, Deja	An international perspective on hospita	Eur. J. Intern. Med.	No specific focus on specialists or the professions
PTWTBMP5	2021	Radu, Razvan I; Ber	Antithrombotic and anticoagulation the	ESC Hear. Fail.	No specific focus on specialists or the professions
44EXVV92	2019	Rafiei, Sima; Abdol	Health manpower forecasting: A system	Bangladesh J. Med.	No specific focus on specialists or the professions
B8HBRJND	2017	Ragin, Deborah Fis	Health Psychology: An Interdisciplinary Approach, Third Edit		No specific focus on specialists or the professions
W7IRXVCT	2021	Raharjo, Sunu Budh	Trainees' perceptions and expectations	J. Educ. Eval. Health	No specific focus on specialists or the professions
Q7A87GDN	2021	Rahma, Azhar T; Elb	Stakeholders' Interest and Attitudes tov	Public Health Geno	No specific focus on specialists or the professions
UPC2B34L	2021	Rahma, Azhar T; Elb	Development of the pharmacogenomic	Hum. Genomics	No specific focus on specialists or the professions
D7VUPPGW	2019	Rahman, Atif; Khan	Effectiveness of a brief group psycholog	Lancet	No specific focus on specialists or the professions
P4YZB43P	2022	Rahman, Mohamm	Socio-economic factors affecting high i	Global. Health	No specific focus on specialists or the professions
X949UDI9	2019	Rai, Sulochana Dha	Caesarean Section rates in South Asian	J. Asian Midwives	No specific focus on specialists or the professions
RR9Q78MF	2022	Raiff, Elizabeth M;	Mental Health in Obstetric Patients and	Clin. Obstet. Gyneco	No specific focus on specialists or the professions
KCXV845Q	2011	Raj, Rishi; Prasad,	Isoniazid preventive therapy programm	Natl. Med. J. India	No specific focus on specialists or the professions
YI9VQ4FS	2011	Rajabi, Fatemeh; M	Trends in medical education, an examp	Arch. Iran. Med.	No info on specialists' contribution to health systems/population health, or their governance
ZZQHJT9N	2019	Rajaguru, Praveen	Understanding surgical care delivery in	Glob. Heal. Res. pol	No specific focus on specialists or the professions
NKWNJIAK	2016	Rajasulochana, S;	Expectant Mother's Preferences for Ser	J. Health Manag.	No specific focus on specialists or the professions
NHERNWPE	2019	Rajbhandari, H; Jos	Epilepsy field workers, a smartphone ap	Seizure	No specific focus on specialists or the professions
TJFPZ8U5	2023	Rajest, S Suman; Si	Recent developments in machine and human intelligence		No specific focus on specialists or the professions
YL558P9H	2023	Ramezani, Maryam	The application of artificial intelligence	Cost Eff. Resour. All	No specific focus on specialists or the professions
NAY2USYG	2021	Randall, T C; Soma	Reaching the women with the greatest	Int. J. Gynecol. Obst	No specific focus on specialists or the professions
AE4VID6K	2023	Rao, Krishna D; Me	Improving quality of non-communicable	Soc. Sci. Med.	No specific focus on specialists or the professions
WLZL3DQ2	2013	Rao, Krishna D; Rya	Rural clinician scarcity and job prefere	PLoS One	
76C8SNLV	2021	Rao, Pavithra K; Cu	Applying LEAN Healthcare in Lean Setti	J. Surg. Res.	No specific focus on specialists or the professions
VTPC3X6M	2021	Rasheed, Muneera	Development and feasibility testing of a	Pilot FEASIBILITY St	No specific focus on specialists or the professions
NWPAKUDY	2012	Raviele, Antonio; N	Venice chart international consensus d	J. Cardiovasc. Elect	No specific focus on specialists or the professions
X5CX6TH5	2022	Ray, Sunanda; Mad	Proposal to set up a College of Family M	AFRICAN J. Prim. He	No specific focus on specialists or the professions
TG42Q5N9	2016	Rayburn, William F	Racial and Ethnic Differences Between	Obstet. Gynecol.	Not on LMICs
FHT5EKUX	2021	Raykar, Nakul P; Ma	Assessing the global burden of hemorrh	SAGE Open Med.	No specific focus on specialists or the professions
3G27LLZ2	2014	Rech, Dino; Bertra	Surgical efficiencies and quality in the p	PLoS One	No specific focus on specialists or the professions
PQRJ7YB2	2022	Reddi, Alluru S	Absolute Nephrology Review: An Essential Q & A Study Guid		No specific focus on specialists or the professions
ZD9ATGWQ	2013	Reid, Michael J A; K	Assessing the impact of airborne outrea	J. Public Health Afri	No specific focus on specialists or the professions
TSGGRLAC	2004	Reimann, J O F; Tal	Cultural competence among physician	Soc. Sci. & Med.	No specific focus on specialists or the professions
EY63NM8Q	2019	Remme, Michelle;	Self care interventions for sexual and re	BMJ	No specific focus on specialists or the professions
LX6TW5TR	2009	Renfrew, M J; Craig	Breastfeeding promotion for infants in r	Health Technol. Ass	No specific focus on specialists or the professions
VMRDUP8C	2013	Rengerink, Katrien	Tools to assess Evidence-Based Practic	Evid. Based. Med.	No specific focus on specialists or the professions
5RSQIU89	2022	Restivo, Vincenzo;	Leadership Effectiveness in Healthcare	Int. J. Environ. Res.	No specific focus on specialists or the professions
YE733BRK	2021	Reyes, Ana M; Akan	Interventions Addressing Social Needs	Heal. Equity	No specific focus on specialists or the professions
74SWMAKC	2017	Rhoads, Christine J	Telehealth in Rural Hospitals: Lessons Learned from Pennsy		No info on specialists' contribution to health systems/population health, or their governance
DJSL9DK6	2017	Rhodes, Andrew; Ev	Surviving Sepsis Campaign: Internation	Crit. Care Med.	No specific focus on specialists or the professions
633XP3Y9	2020	Ribeiro, Reitan; Fo	Brazilian Society of Surgical Oncology g	J. Surg. Oncol.	No specific focus on specialists or the professions
2YCC37HW	2021	Riccomi, Giulia	Bioarchaeology and Dietary Reconstruction across Late An		No specific focus on specialists or the professions
YYVWELS5	2021	Rice, Thomas; Rose	Health Systems in Transition: USA, Second Edition		Not on LMICs
3Y5J9RA7	2024	Richmond, Jennifer	All of Us participant perspectives on the	Genet. Med.	No specific focus on specialists or the professions
VD84SZX7	2018	Riley, Sarah; Evans	POSTFEMINISM AND HEALTH: Critical Psychology and Medi		No specific focus on specialists or the professions
3KIUEVWD	2022	Rinaldi, Ikhwan; Fir	Identification and Validation of Ent	Acta Med. Indones.	
F59IER94	2019	Rispel, Laetitia Cha	Socio-economic characteristics and ca	PLoS One	
95ZT6LLS	2024	Riva, J; Calviño, J; E	Heterogeneity Among Countries in the S	J. Cardiothorac. Vasc. Anesth.	
ZZW7WCD2	2019	Rivas, Carol; Vigurs	A realist review of which advocacy inter	Cochrane Databas	No specific focus on specialists or the professions
NL8IMRBL	2005	Roberts, L W; Gepp	Bioethics principles, informed consent	Psychosomatics	No specific focus on specialists or the professions
X26L47N5	2005	Roberts, Laura W; V	Becoming a good doctor: perceived nee	Acad. psychiatry J. A	No specific focus on specialists or the professions
225HUQX6	2012	Roberts, Richard G	Depression and diabetes: The role and i	J. Affect. Disord.	No specific focus on specialists or the professions
LZ8RREBL	2020	Robertson, Faith C	The World Federation of Neurosurgical	World Neurosurg. X	
DH369GJK	2023	Robinson, David J;	Diabetes and Mental Health	Can. J. Diabetes	No specific focus on specialists or the professions
MPC8DBVB	2022	Robinson, Dudley;	International Urogynaecology Consulta	Int. Urogynecol. J.	No specific focus on specialists or the professions
HZIVFCAN	2017	Rocha, T A H; da Sil	Access to emergency care services: a tr	Public Health	No info on specialists' contribution to health systems/population health, or their governance
E9SZTZRN	2020	Roder-Dewan, San	Health system redesign for maternal an	BMJ Glob. Heal.	No specific focus on specialists or the professions
RMSIJNWE	2023	Rodler, Severin; Ra	The Impact of Telemedicine in Reducing	Eur. Urol. Focus	No specific focus on specialists or the professions
QGHGVT7I	2023	Rodriguez-Mena, R	The NED foundation experience: A mod	BRAIN AND SPINE	
GP8XKKPI	2023	Rodriguez-Saldana	Clinical Practice Guidelines, Evidence-Based Medicine and		No specific focus on specialists or the professions
NF2W727M	2023	Rodriguez-Saldana	Clinical Inertia and Diabetes Outcomes		No specific focus on specialists or the professions
ECXEH9Y3	2014	Rodriguez, Jose E; C	Where Are the Rest of Us? Improving Re	South. Med. J.	No specific focus on specialists or the professions
YXIIR6TX	2023	Rodríguez, Manuel	Bioethics of Displacement and Its Implications		No specific focus on specialists or the professions
PE62R5PB	2019	Rogerson, Jennifer	Privileges of birth: Constellations of care, myth, and race in		No specific focus on specialists or the professions
EG7MY9ZV	2023	Rokach, Ami; Clayt	Adverse Childhood Experiences and Their Life-Long Impact		No specific focus on specialists or the professions
UY3WZCFR	2021	Rokach, Ami; Patel	Human Sexuality: Function, Dysfunction, Paraphilias, and R		No specific focus on specialists or the professions
5CPN2K25	2022	Roman-Urrestaraz	Autism incidence and spatial analysis i	Lancet. Child Adole	No specific focus on specialists or the professions
IH64NHYC	2009	Romm, Aviva; Hard	Botanical Medicine for Women's Health		No specific focus on specialists or the professions
84QKMA74	2015	Roopnarinesingh, N	Barriers to optimal diabetes care in Trin	BMC Health Serv. R	No specific focus on specialists or the professions
X5M96XG9	2016	Rossaint, Rolf; Bou	The European guideline on managemen	Crit. Care	No specific focus on specialists or the professions
UBFIPPNL	2021	Rossaki, Foteini M;	Strategies for the prevention, diagnosis	Expert Rev. Respir.	No specific focus on specialists or the professions
QVAL2UVT	2014	Rossi, Paolo Giorgi	The possible effects on socio-economic	Front. Oncol.	No specific focus on specialists or the professions
YTPLVQC6	2019	Rouhi, Maryam; Sti	Mothers' views of health problems in th	J. Adv. Nurs.	No specific focus on specialists or the professions
334ZIPM9	2020	Rousseva, Christia	Health workers' views on audit in mater	Trop. Med. Int. Heal	No specific focus on specialists or the professions
2DQXTW3H	2023	Roy, Emily; Chino,	Increasing Diversity of Patients in Radia	Int. J. Radiat. Oncol	No specific focus on specialists or the professions
ARNU7I2T	2021	Royal, Abhishek; M	Harnessing the potential of the primary	J. Fam. Med. Prim. c	No specific focus on specialists or the professions
JE9NQAQU	2015	Rubin, Greg; Beren	The expanding role of primary care in ca	Lancet Oncol.	No specific focus on specialists or the professions
MPHCWFSZ	2023	Rudolfson, Niclas;	South Africa and the Surgical Diaspora-	World J. Surg.	No specific focus on specialists or the professions
EDHR4PUV	2013	Ruggunan, Shaun D	Sector switching among histopathologi	Hum. Resour. Health	
FGF4LEIL	2021	Rukavina, Tea Vuku	Dangers and benefits of social media or	J. Med. Internet Res	No specific focus on specialists or the professions
32J8PU54	2016	Rupp, Deborah E; S	Using assessment centres to facilitate collaborative, quasi-		No specific focus on specialists or the professions
F6VX74CV	2020	Russo, Giuliano; Ca	The role of private education in the sele	Hum. Resour. Healt	No specific focus on specialists or the professions

Appendix 4

Table S4: List of papers excluded, with reason

INU9CZG4	2015	Russo, Giuliano; G	Feminization of the medical workforce	Hum. Resour. Healt	No specific focus on specialists or the professions
59XNTSTH	2014	Russo, Giuliano; M	Negotiating markets for health: an expl	Health Policy Plan.	No specific focus on specialists or the professions
GJ4D9ZZZ	2020	Ryan, Nessa; Vieira	Implementation outcomes of policy and	Health Policy Plan.	No specific focus on specialists or the professions
JVIZU689	2022	Saad, Rima; Huijer	Pediatric palliative care through the ey	Ann. Palliat. Med.	No specific focus on specialists or the professions
6LLNQ2WG	2022	Sabatino, Marlena	Access to paediatric cardiac surgery in	Eur. J. cardio-thora	No specific focus on specialists or the professions
IIVBCLLC	2019	Sabitova, Alina; Sa	Job morale of physicians in low-income	BMJ Open	No specific focus on specialists or the professions
3MQQGXGZ	2011	Sachdeva, Ajit K; B	A New Paradigm for Surgical Procedura	Curr. Probl. Surg.	No specific focus on specialists or the professions
ZS23KSQJ	2010	Sacristan, Cristina	The Castaneda's contribution to the pr	SALUD Ment.	No specific focus on specialists or the professions
6WMCI8BD	2003	Sacristán, M C	Reforming psychiatric care in Mexico.:	SALUD Ment.	No specific focus on specialists or the professions
7RUQM6QQ	2013	Sadeghian, G.-H.; S	Prescribing quality in medical specialis	Iran. J. Pharm. Res.	No specific focus on specialists or the professions
49ZGSC9N	2020	Sadler, Diego; DeC	Perspectives on the COVID-19 pandem	Cardio-Oncology	No specific focus on specialists or the professions
9ZLSZKP6	2021	Safe, Izabella	From female warriors in the rainforest t	Lancet Respir. Med	No specific focus on specialists or the professions
CIGBVPJ7	2021	Sah, Pratha; Fitzpa	Asymptomatic SARS-CoV-2 infection: A	Proc. Natl. Acad. Sc	No specific focus on specialists or the professions
6ZVHGI98	2017	Saini, Vikas; Garcia	Drivers of poor medical care	Lancet	No specific focus on specialists or the professions
CNACP9UM	2018	Saint-Pierre, Cecil	Multidisciplinary collaboration in prima	Fam. Pract.	No specific focus on specialists or the professions
G3I5BG8Y	2020	Saint-Pierre, Cecil	Team Collaboration Networks and Mult	IEEE J. Biomed. Hea	No specific focus on specialists or the professions
RJ4MP4L9	2022	Sakr, Mahmoud F	Tongue Lesions: Diagnostic Challenges and Therapeutic Str		No specific focus on specialists or the professions
EWHH2AKW	2012	Sakran, Joseph V; G	Care of the injured worldwide: Trauma	Scand. J. Trauma. R	No specific focus on specialists or the professions
6HPSIC8W	2020	Salamanca-Buente	The ethical, social, and cultural dimens	PLoS One	No specific focus on specialists or the professions
WC6UBR5R	2018	Salampessy, Benja	The effect of cost-sharing design chara	BMC Health Serv. R	No specific focus on specialists or the professions
INJIF7R9	2022	Saleem, Zikria; Go	Ongoing Efforts to Improve Antimicrobi	Antibiotics	No specific focus on specialists or the professions
ESIPYARR	2019	Saleem, Zikria; Has	A multicenter point prevalence survey	Expert Rev. Anti. Inf	No specific focus on specialists or the professions
5AA8MRD6	2021	Samad, Nandeeta;	The implications of zinc therapy in com	J. Inflamm. Res.	No specific focus on specialists or the professions
YA65JCLM	2019	Samanta, Indranil;	Antimicrobial resistance in agriculture:	Perspective, policy	No specific focus on specialists or the professions
RWHNUJC8	2022	Sami, Ramin; Saleh	Barriers to rational antibiotic prescript	Antimicrob. Resist.	No specific focus on specialists or the professions
7PKU3WMP	2022	Sánchez-Viñas, Alb	Effectiveness and cost-effectiveness of	BMC Prim. Care	No specific focus on specialists or the professions
56IKN488	2024	Sandall, Jane; Fern	Midwife continuity of care models vers	Cochrane database	No specific focus on specialists or the professions
JG5SYD9F	1998	Sanders, D; Kravitz	Zimbabwe's Hospital Referral System:	Health Policy Plan.	
U4TPKBI3	2024	Sandhya, Shaifali	Displaced: Refugees, trauma, and integration within nation		No specific focus on specialists or the professions
HEVU4BBL	2021	Sanoe, I; Beyan-Da	The Role of Family Medicine Training in	Ann. Glob. Heal.	No info on specialists' contribution to health systems/population health, or their governance
SI76N9PC	2018	Santas, Gulcan; Sa	Trends of caesarean section rates in Tu	J. Obstet. Gynaecol	No specific focus on specialists or the professions
584URNUJ	2022	Santhanam, Indum	Triage and resuscitation tools for low a	Arch. Dis. Child. Ed	No specific focus on specialists or the professions
EM2A2UZW	2018	Saravanan, Sheela	A transnational feminist view of surrogacy biomarkets in Ind		No specific focus on specialists or the professions
VK56N3WX	2021	Sarikhani, Yaser; G	A thematic network for factors affecting	BMC Med. Educ.	
JL6K7NMV	2022	Sarikhani, Yaser; G	What do Iranian physicians value most	Cost Eff. Resour. Alloc.	
TVGLDP3P	2020	Sarin, Shiv K; Kuma	Liver diseases in the Asia-Pacific region	Lancet Gastroenter	No specific focus on specialists or the professions
C69LIZV2	2018	Sartelli, Massimo;	2018 WSES/SIS-E consensus conferenc	World J. Emerg. Sur	No specific focus on specialists or the professions
Y3IEH5A5	2017	Sartelli, Massimo;	The Global Alliance for Infections in Sur	World J. Emerg. Sur	No specific focus on specialists or the professions
YB9MVRVS	2020	Sawe, Hendry R; Si	Barriers and facilitators to implementi	African J. Emerg. Me	No specific focus on specialists or the professions
EII6D2K4	2020	Scazufca, Marcia;	A collaborative care psychosocial inter	Trials	No specific focus on specialists or the professions
YSVKGXRZ	2024	Scelo, Ghislaine; T	Analysis of comorbidities and multimor	Ann. allergy, asthm	No specific focus on specialists or the professions
62TPTLQM	2022	Scerpella, Tamara	Next Steps: Advocating for Women in O	J. Am. Acad. Orthop	Not on LMICs
HTV966BS	2021	Schaaf, Marta; Kho	Necessary but not sufficient: A scoping	BMJ Glob. Heal.	No specific focus on specialists or the professions
F7TH74RV	2023	Schaaf, Marta; Lav	A narrative review of challenges related	BMJ Glob. Heal.	No specific focus on specialists or the professions
U6UV5WXB	2024	Schantz, C; Coulib	Access to oncology care in Mali: a quali	BMC Cancer	No specific focus on specialists or the professions
YTZNW2WC	2017	Scheffer, Mário C;	The state of the surgical workforce in Br	Surg. (United States)	
2WUB4DND	2021	Schell, Carl Otto; K	Essential Emergency and Critical Care:	BMJ Glob. Heal.	No specific focus on specialists or the professions
YDUSSLKL	2018	Scheuner, Maren T	Genetic evaluation for common, chronic disorders of adulth		No specific focus on specialists or the professions
I6WLE3JJ	2018	Schleiss, Mark R; M	Viral Infections of the Fetus and Newborn		No specific focus on specialists or the professions
DUTA4MVH	2012	Schleiss, Mark R; P	Viral Infections of the Fetus and Newborn and Human Immu		No specific focus on specialists or the professions
NI98YB8V	2018	Schluger, Neil W; S	Creating a specialist physician workfor	BMJ Glob. Heal.	
SMG5SK8P	2022	Schneider, Patrick;	Anomaly Detection and Complex Event Processing Over IoT		No specific focus on specialists or the professions
IDS9P4PE	2017	Schoeman, Renata	The South African Society of Psychiatris	SOUTH AFRICAN J.	No specific focus on specialists or the professions
KVMCYZC8	2012	Schoenbaum, Step	Planning and managing the physician w	Isr. J. Health Policy	Not on LMICs
5FM45XPT	2022	Schror, Karsten	Acetylsalicylic acid		No specific focus on specialists or the professions
5XYD9SVS	2020	Schühle, Judith	Traversing Transnational Biomedical Landscapes: An Ethno		No info on specialists' contribution to health systems/population health, or their governance
5L3ESLLX	2008	Schulkin, Jay	Medical decisions, estrogen and aging		No specific focus on specialists or the professions
NBB8V2LY	2013	Schulman, Kathy L;	A checklist for ascertaining study cohor	Value Heal.	No specific focus on specialists or the professions
B8A7E7FA	2021	Schults, Jessica; K	International recommendations for a v	BMJ Qual. Saf.	No specific focus on specialists or the professions
RYNY3EXC	2008	Schutze, Gordon E	Education and preparation of physician	AIDS Patient Care STDS	
CFIQ782A	2015	Schwartz, David A	Pathology of maternal death - the importance of accurate a		No specific focus on specialists or the professions
4V8LWN7N	2009	Schwartz, G F; Plak	An assessment of readiness for behavio	Eye (Lond).	No specific focus on specialists or the professions
C2JNIQLI	2012	Scott, Anthony; Jan	Primary Care		No specific focus on specialists or the professions
ERR6UVMG	2023	Scott, Michael J; A	Consensus Guidelines for Perioperative	World J. Surg.	No specific focus on specialists or the professions
YQGGITTW	2022	Searle, Kendall; Bl	Adapting the depression component of	Int. J. Ment. Health	No specific focus on specialists or the professions
99B5WH5T	2019	Searle, Kendall; Bl	Current needs for the improved manag	Int. J. Ment. Health	No specific focus on specialists or the professions
BYXQQGI6	2012	Searles, Christoph	Beyond health care reform: Immigrants	Ethn. Racial Stud.	No specific focus on specialists or the professions
X4MRJJK4	2008	Seeber, Petra; Sha	Basics of Blood Management: First Edition		No specific focus on specialists or the professions
KUE4JWZS	2012	Seeber, Petra; Sha	Basics of Blood Management, Second Edition		No specific focus on specialists or the professions
V6DWQFSV	2014	Seeley, Janet	HIV and east africa: Thirty years in the shadow of an epidem		No specific focus on specialists or the professions
JTKGWKEW	2013	Segal, Leonie; Lea	Regional Primary Care Team to Deliver	Diabetes Care	No specific focus on specialists or the professions
IAP2MR44	2020	Semaan, Aline; Aud	Voices from the frontline: findings from	BMJ Glob. Heal.	No specific focus on specialists or the professions
2KGMY6UT	2022	Semaan, Aline; Ba	'We are not going to shut down, becaus	BMJ Glob. Heal.	No specific focus on specialists or the professions
4A5C35V6	2017	Sengoma, Jean Pau	Prevalence of pregnancy-related comp	BMJ Open	No specific focus on specialists or the professions
VLL3RXT8	2024	Serrano, Jose Anto	Vulnerable period in heart failure: a wi	DRUGS Context	No specific focus on specialists or the professions
9M96KSNF	2021	Sethi, Naqash J; Sa	Antibiotics for secondary prevention of	Cochrane Databas	No specific focus on specialists or the professions
IIQ3PVXQ	2022	Seward, Nadine; H	HeAlth System StrEngThening in four su	Glob. Health Action	No specific focus on specialists or the professions
CKTAZCQ8	2021	Shah, Nikhil; Math	The role of evaluation in iterative learn	Glob. Health Action	No specific focus on specialists or the professions
9AGBSURF	2023	Shah, Rupin; Agarw	Consensus and Diversity in the Manage	World J. Mens. Hea	No specific focus on specialists or the professions
ZXEGCSCT	2022	Shah, Sanket S; Bu	Impact of Technologic Innovation and C	Curr. Treat. Option	No specific focus on specialists or the professions
JRPANRXB	2021	Shahbaz, Sumbal; Z	Anesthesia Health System Capacities i	Inq. J. Heal. Care O	No info on specialists' contribution to health systems/population health, or their governance
HBJDA3V6	2019	Shahmalak, Ujala;	The experiences of lay health workers t	Int. J. Ment. Health	No specific focus on specialists or the professions
5DE8HFLL	2020	Shahrajabian, Moh	Traditional Herbs and Foods with Medicinal Value		No specific focus on specialists or the professions
HMESYB4F	2021	Shaikh, Irum; Küng,	Telehealth for Addressing Sexual and R	Front. Glob. Wome	No specific focus on specialists or the professions
N9IAUHAY	2011	Shale, Suzanne	Moral leadership in medicine: Building ethical healthcare o		No specific focus on specialists or the professions
Y3N9GRTW	2020	Shalowitz, David I;	Telemedicine and Gynecologic Cancer	Obstet. Gynecol. C	No specific focus on specialists or the professions
H8JRS6HB	2019	Shantha, Jessica G	Development of a screening eye clinic f	PLoS Negl. Trop. Dis	No specific focus on specialists or the professions
PWAFCAGI	2021	Sharma, Gaurav; M	Analysis of maternal and newborn train	PLoS One	No specific focus on specialists or the professions
T9WA4643	2022	Sharma, Vishnu; Ku	The role of innovative telehealth system in revolutionizing h		No specific focus on specialists or the professions
X5FLTBYX	2023	Sharmin, Sifat; Roo	The risk of secondary progressive multi	Brain	No specific focus on specialists or the professions
U8X3KV5Q	2006	Sharpe, Neil F; Car	Genetic Testing: Care, Consent, and Liability		No specific focus on specialists or the professions
VH2ZHCXF	2010	Shavers, Vickie L; B	Race, Ethnicity, and Pain among the US	J. Health Care Poor	No specific focus on specialists or the professions
I6KUYJBS	2022	Shayganmehr, Aref	Investigating the Role of Using Electron	J. Qual. Res. Heal. S	No specific focus on specialists or the professions
CS7FT25G	2021	Shead, D C; Chetty	Smartphone and app usage amongst So	South. AFRICAN J. A	No specific focus on specialists or the professions
I9QX2HFS	2020	Shen, Yang; Cui, Yi	Emergency Responses to Covid-19 Out	Cardiovasc. Interve	No specific focus on specialists or the professions
M7WE6ZVQ	2023	Sherertz, Robert J;	Covid chaos: What happened and Why		No specific focus on specialists or the professions
8EP4SMGK	2016	Sherman, Charles	The East African Training Initiative. A M	Ann. Am. Thorac. Soc.	
594QAF5T	2020	Sherman, Jodi D; T	The Green Print: Advancement of Envir	Resour. Conserv. R	No specific focus on specialists or the professions
5BRASJQ3	2011	Shi, Fu-Dong; Jia, J	Neurology and neurologic practice in C	Neurology	
ULDIHGKH	2020	Shikuku, Duncan N	The effect of the community midwifery	BMC Pregnancy Ch	No specific focus on specialists or the professions
SEBEXQQZ	2022	Shin, Gee Won; Le	Investigation of usability problems of el	Work	No specific focus on specialists or the professions
CSKC4AAN	2023	Shin, Ji-Hye; Yim, S	A Foundation for a "Cheerful Society": T	Uisahak.	No info on specialists' contribution to health systems/population health, or their governance
CX6LEUVR	2019	Shinn, Justin R; Zu	Community health workers obtain simi	Int. J. Pediatr. Otor	No specific focus on specialists or the professions
WMEGQDPJ	2021	Shirzad, Mahboube	Prevalence of and reasons for women's,	Reprod. Health	No specific focus on specialists or the professions
YVT3RREL	2019	Shmueli, Liora; Sh	Second opinion utilization by healthca	BMJ Open	No specific focus on specialists or the professions
SPP3SQKQ	2023	Shoham, Shmuel; B	Vaccines and therapeutics for immuno	EClinicalMedicine	No specific focus on specialists or the professions

Appendix 4

Table S4: List of papers excluded, with reason

T2GBWEW5	2024	Shore, Neal D; Broo	Expert Consensus Recommendations o	Eur. Urol. Oncol.	No specific focus on specialists or the professions
NC6WI99Q	2022	Shrestha, Sunil; KC	Pharmacist Involvement in Cancer Pair	J. Pain	No specific focus on specialists or the professions
7UV2MRPZ	2016	Shrime, Mark G; Ve	Task-sharing or public finance for the ex	Health Policy Plan.	No specific focus on specialists or the professions
DY2URCZC	2019	Shroff, Farah; Minh	Power of partnerships What makes a dif	Int. J. Heal. Gov.	No specific focus on specialists or the professions
B4LQVPTA	2024	Shumnalieva, Russ	Characteristics of emerging new autoir	Int. J. Rheum. Dis.	No specific focus on specialists or the professions
HSF9GF9C	2024	Siafis, Spyridon; Wu	Effectiveness of pharmacological treati	Syst. Rev.	No specific focus on specialists or the professions
SHEMMERK	2022	Sibbing-Plantholt, I	The Image of Mesopotamian Divine Hea	Cuneif. Monogr.	No specific focus on specialists or the professions
VEZK4X32	2021	Sidani, Souraya; Br	Nursing and Health Interventions: Design, Evaluation, and I		No specific focus on specialists or the professions
NN933TAH	2013	Sidani, Souraya; Jo	Design, Evaluation, and Translation of Nursing Interventions		No specific focus on specialists or the professions
YQMZHGSY	2023	Sideman, Alissa Be	Primary Care Pracitioner Perspectives (	JAMA Netw. Open	No specific focus on specialists or the professions
BWADTRSE	2013	Sifris, Ronli	Reproductive freedom, torture and international human rig		No specific focus on specialists or the professions
477PQQH8	2021	Silpa-Archa, Sukhu	Capacity building in screening and trea	Indian J. Ophthalmo	No specific focus on specialists or the professions
N3698KTH	2020	Sim, Ming Ann; Lee,	Quality improvement at an acute medic	Nurs. Outlook	No specific focus on specialists or the professions
GZFPAMFB	2020	Şimşek-Yavuz, Sera	Consensus report on diagnosis, treatme	Turkish J. Thorac. Ca	No specific focus on specialists or the professions
Y2FDL6ZU	2017	Singer, Merrill; Alle	Social justice and medical practice: Life history of a physici		No specific focus on specialists or the professions
4JWCVR6V	2022	Singh, Hardeep; Ta	Recommendations for the Design and D	JMIR Aging	No specific focus on specialists or the professions
FLUML3GB	2024	Singh, Khushbu; Wa	Use of mHealth in promoting maternal a	PLOS Digit. Heal.	No specific focus on specialists or the professions
TFYQLMY4	2024	Singh, Pratishtha; S	Examining representation of women in l	PLOS Glob. Public H	No specific focus on specialists or the professions
3C9HICQN	2021	Singh, Sanjeev; Me	Investigating infection management an	Clin. Microbiol. Infe	No specific focus on specialists or the professions
GV7QSIBK	2022	Singhal, Tanu; Rodr	Treatment of MRSA infections in India: (	Indian J. Med. Micro	No specific focus on specialists or the professions
VISC2C67	2021	Sipos, Adrienn; Ujla	The role of the microbiome in ovarian ca	Mol. Med.	No specific focus on specialists or the professions
LBLJ2IB5	2022	Skempes, Dimitrios	Supporting government policies to emb	Health Policy (New	No specific focus on specialists or the professions
RNEE46NU	2019	Skop, Ingrid	Abortion safety: At home and abroad	Issues Law Med.	No specific focus on specialists or the professions
WP7TXVKI	2014	Smart, Neil A; Dieb	Early referral to specialist nephrology s	Cochrane Databas	No specific focus on specialists or the professions
IJ4QBGD9	2018	Smelson, David A; (	An evidence-based co-occurring disord	BMC Health Serv. R	No specific focus on specialists or the professions
D8U3DQUA	2023	Smith, Emily R; Oak	Adverse maternal, fetal, and newborn o	BMJ Glob. Heal.	No specific focus on specialists or the professions
E77GE5HZ	2022	Smith, Shantelle; B	Development of an Australia and New Z	BMJ Open	No specific focus on specialists or the professions
EUDI7E3B	2020	Snodgrass, Alexand	Systemic Antibacterial Agents		No specific focus on specialists or the professions
LVLHXMLV	2019	Soekhai, Vikas; de E	Discrete Choice Experiments in Health	Pharmacoeconomi	No specific focus on specialists or the professions
W72KVPKP	2018	Solanke, Bola Lukm	Multilevel analysis of factors associate	BMC Pregnancy Ch	No specific focus on specialists or the professions
JYABX8LF	2024	Solomon, Scott D; I	Finerenone in Heart Failure with Mildly	N. Engl. J. Med.	No specific focus on specialists or the professions
DYLMTCTG	2024	Solomon, Scott D; (	Baseline characteristics of patients wit	Eur. J. Heart Fail.	No specific focus on specialists or the professions
VNY8S8WQ	2024	Somerville, Joel G;	Topical review: Task shifting and the rec	Optom. Vis. Sci.	No specific focus on specialists or the professions
L35VGN8D	2019	Song, Yujie; Li, Hon	Clinical Guidelines for the Surgical Mar	Orthop. J. Sport. Me	No specific focus on specialists or the professions
939KBM3Q	2022	Sorhaindo, Annik M	Why does abortion stigma matter? A sco	Soc. Sci. Med.	No specific focus on specialists or the professions
YIV3HT3K	2010	Sosenski, Susana; S	In defense of children and women: a life	SALUD Ment.	No specific focus on specialists or the professions
QZSFMUD3	2017	Sow, Doudou; Soro	Epidemiological profile of cutaneous la	Travel Med. Infect. I	No specific focus on specialists or the professions
SZ6IEHZQ	2018	Spanu, Fabio; Pique	Practical Challenges and Perspectives	World Neurosurg.	
PY6GQAVD	2019	Spedding, Maxine F	Emerging models of psychotherapy		No info on specialists' contribution to health systems/population health, or their governance
MW9F8X3B	2020	Speerin, Robyn; Ne	Implementing models of care for muscu	Best Pract. Res. Cli	No info on specialists' contribution to health systems/population health, or their governance
BJUSRF93	2019	Spence, Dingle; Arg	Advancing cancer care and prevention	Lancet. Oncol.	No info on specialists' contribution to health systems/population health, or their governance
UBSMANYT	2022	Spiess, Adrian Andr	Exploration of current challenges in reh	Health Policy (New	Not on LMICs
Z27Q2SIB	2024	Spijkerman, Sandra	Undergraduate Anesthesia Skills for a G	Anesth. Analg.	No info on specialists' contribution to health systems/population health, or their governance
JRA7JNLF	2018	Sriram, Veena; Bar	Regulating recognition and training for	Health Policy Plan.	
D8LTI9LP	2020	Sriram, Veena; Ben	Strengthening medical specialisation p	BMJ Glob. Heal.	
RPLDET9F	2018	Sriram, Veena; Geo	Socialization, legitimation and the tran	Int. J. Equity Health	No specific focus on specialists or the professions
L8M2KPKD	2022	Stabnick, Anna; Yel	“Once you get one maternal death, it's l	BMC Pregnancy Ch	No specific focus on specialists or the professions
AMZ2BGEY	2017	Stacey, Dawn; Léga	Decision aids for people facing health t	Cochrane Databas	No specific focus on specialists or the professions
2KQ4QCY5	2024	Stacey, Dawn; Lew	Decision aids for people facing health t	Cochrane Databas	No specific focus on specialists or the professions
M8R8TLW9	2008	Stalsberg, H; Awua	Re-establishing a Surgical Pathology Se	Cancer	No specific focus on specialists or the professions
NGIP7MY5	2009	Stange, Kurt C; Fer	The paradox of primary care	Ann. Fam. Med.	No specific focus on specialists or the professions
7MNCEF7Z	2008	Stanley, Ava; Cant	Holes in the safety net: A case study of a	J. Urban Heal.	No specific focus on specialists or the professions
28GRM6X3	2023	Stanley, Njaka; Bin	Career Intentions and the Determining	Creat. Nurs.	No specific focus on specialists or the professions
WAFLZFTP	2015	Starbuck, Gene H;	Families in context: Sociological perspectives, third edition		No specific focus on specialists or the professions
MU6RJZ3D	2018	Starrs, Ann M; Ezeh	Accelerate progress—sexual and repro	Lancet	No specific focus on specialists or the professions
3VNFRCDN	2006	Steel, Zachary; Mc	Pathways to the first contact with spec	Aust. N. Z. J. Psychia	No specific focus on specialists or the professions
R5DTTDAG	2015	Stein, Dan J; He, Ya	Global mental health and neuroscience	The lancet. Psychia	No specific focus on specialists or the professions
MWZIT2MW	2016	Stein, Paul D	Pulmonary Embolism: Third Edition		No specific focus on specialists or the professions
MTXTBZLB	2022	Stein, Richard A; D	Emergency Contraception: Access and	Am. J. Ther.	No specific focus on specialists or the professions
BK4T9QW5	2024	Steinmetz, Jaimie D	Global, regional, and national burden o	Lancet Neurol.	No specific focus on specialists or the professions
7G8RWDMB	2022	Stenkjaer, Rikke Lo	Development of a non-pharmacologic d	Nurs. Crit. Care	No specific focus on specialists or the professions
2FB42ZP6	2017	Stettner, Shannon;	Transcending borders: Abortion in the past and present		No specific focus on specialists or the professions
7S55BTKE	2020	Stevens, Craig W	The Drug Expert: A Practical Guide to the Impact of Drug Use		No specific focus on specialists or the professions
T9GTNSW6	2024	Steventon, Luke; Ki	The impact of inter-cycle treatment del	Eur. J. Cancer	No specific focus on specialists or the professions
5RFZM6ZR	2021	Stever, Gayle S; Gil	Understanding Media Psychology		No specific focus on specialists or the professions
4YGNZR5V	2016	Stewart, Barclay T;	Surgical Burn Care by Médecins Sans F	J. Burn care Res. Of	No specific focus on specialists or the professions
JHWXKDQJ	2013	Stewart, Moira; Bro	Patient-Centered Medicine: Transforming the Clinical Meth		No specific focus on specialists or the professions
XIDN8KP4	2013	Stiavetti, E; Ghinol	Analysis of patients' needs after liver tra	Transplant. Proc.	No specific focus on specialists or the professions
LTNK9UT2	2020	Stillhart, Cordula; V	Impact of gastrointestinal physiology or	Eur. J. Pharm. Sci.	No specific focus on specialists or the professions
S42L9CK3	2023	Stirling, Dale A	From Hippocrates to COVID-19: A bibliographic history of m		No specific focus on specialists or the professions
9G4N47JQ	2016	Stockmann, Reinha	The future of evaluation: Global trends, new challenges, sha		No specific focus on specialists or the professions
S5Q59SYV	2023	Stoehr, J R; Reddy,	The Personal Impact of Involvement in I	J. Pediatr. Surg.	No specific focus on specialists or the professions
9TF3J9IZ	2023	Stoumpos, Angelos	Digital Transformation in Healthcare: T	Int. J. Environ. Res. I	No specific focus on specialists or the professions
BGM9BNH9	2022	Straeuli, Christoph	Patients requiring palliative care attend	African J. Emerg. Me	No specific focus on specialists or the professions
UIVQKKW5	2014	Stratford, Elaine	Geographies, mobilities, and rhythms over the life-course: A		No specific focus on specialists or the professions
FIYIKQ3D	2024	Struckmeyer, Malte	Giraffes and Zebras on the Runway: the	Anasthesiol. Intensi	No specific focus on specialists or the professions
9CWKY73P	2023	Studart-Pereira, Lu	Brazilian Consensus on Sleep-Focused	SLEEP Sci.	No specific focus on specialists or the professions
LHQUB8TW	2019	Studdert, Ann L; Go	Application of pharmacoeconomics to f	Am. J. Heal. Pharm.	No specific focus on specialists or the professions
VSWETQIT	2016	Stulac, Sara; Mark	Initiating Childhood Cancer Treatment	Pediatr. Blood Canc	No specific focus on specialists or the professions
6KR6UH8H	2024	Su, Minzi	Adaptive leadership in times of crisis: E	J. Gen. Manag.	No specific focus on specialists or the professions
M44VH7A2	2015	Suleiman, Iman; Na	Does task shifting have a potential role in improving emerge		No specific focus on specialists or the professions
LHJ5IDU7	2019	Sullivan, S A; String	A Review of Gynecologic Oncology in th	Obstet. & Gynecol.	Not on LMICs
T95LPXYD	2018	Sultan, Sulaiman; J	Safety of dietary and herbal supplements: Side effects and d		No specific focus on specialists or the professions
WHND6YPV	2017	Summer, Anna; Gue	Professional midwifery in Guatemala: A	Soc. Sci. Med.	No specific focus on specialists or the professions
P48Z234Y	2020	Suryaningtyas, Wih	Neurosurgery at the epicenter of the CC	Neurosurg. Focus	No specific focus on specialists or the professions
4A9ET4FY	2023	Suttels, Veronique;	Barriers and facilitators to implementa	BMJ Open	No specific focus on specialists or the professions
IFAX7E25	2014	Sweetland, Annika	Closing the mental health gap in low-in	Ann. Glob. Heal.	No specific focus on specialists or the professions
X62ZACX6	2024	Swenson, Mary Sch	MEDICAL, DENTAL, AND WELLNESS TOURISM: A Post-Pande		No specific focus on specialists or the professions
9CGWSAHM	2015	Syah, Nur A; Robert	Perceptions of Indonesian general prac	Fam. Pract.	No specific focus on specialists or the professions
86TJXQ9Y	2024	Syairaji, M; Nurdiat	Trends and causes of maternal mortalit	BMC Pregnancy Ch	No specific focus on specialists or the professions
YTHVBBQP	2017	Synowiec-Pilat, Ma	Lay beliefs on Polish oncology in the eva	Ann. Agric. Environ.	No specific focus on specialists or the professions
VVYTRGGE	2011	Szablewski, Leszek	Glucose homeostasis and insulin resistance		No specific focus on specialists or the professions
2C4I47CK	2018	Taher, Ahmed; Crav	Position Statement on Resident Wellne	Can. J. Emerg. Med.	No specific focus on specialists or the professions
AUIA5GPY	2022	Tahir, Durr-E-Sami	Efficacy of linking Breast Awareness Cli	Pakistan J. Med. Sci	No specific focus on specialists or the professions
3222VB2F	2022	Tajvar, Maryam; Ha	A systematic review of individual and ec	BMC Public Health	No specific focus on specialists or the professions
Y4IFELC9	2013	Taket, Ann; Crisp, B	Practising social inclusion		No specific focus on specialists or the professions
MAN33FBF	2017	Talib, Zohray; van S	Medical Education in Decentralized Se	Acad. Med.	No specific focus on specialists or the professions
2RUPA79S	2014	Taljaard, Lian	The South African Depression and Anxie	World Hosp. Heal. S	No specific focus on specialists or the professions
BPN6SVGU	2015	Tam, Wilson W S; C	Parental Attitudes and Factors Associat	Medicine (Baltimor	No specific focus on specialists or the professions
PHFLS8B9	2021	Tan, Xiu Hui; Foo, M	Teaching and assessing communicatior	BMC Med. Educ.	No specific focus on specialists or the professions
5C6T8U5L	2022	Tang, Chia-Chun; H	Communication in Oncology Outpatien	J. Oncol.	No specific focus on specialists or the professions
FE9XGH73	2022	Tang, Julian W; Car	An exploration of the political, social, e	Interface Focus	No specific focus on specialists or the professions
Q3D3MMHF	2022	Tannor, A Y; Nelson	Building PRM in sub-Saharan Africa	Front. Rehabil. Sci.	No specific focus on specialists or the professions
EM5YY4PY	2019	Tarver, Will L; Hagg	The use of cancer-specific patient-cent	J. Med. Internet Res	No specific focus on specialists or the professions
XDLGXCG6	2018	Taylor, Bettina; var	Medicolegal storm threatening materna	South African Med.	No specific focus on specialists or the professions
R637HIA6	2015	Tedde, Miguel Lia; F	General thoracic surgery workforce: tra	Eur. J. cardio-thoracic Surg. Off. J. Eur. Assoc. Cardio-thoracic Surg.	

Appendix 4

Table S4: List of papers excluded, with reason

3WCDF2FB	2023	Teede, Helena J; Ta	Recommendations from the 2023 inter	Eur. J. Endocrinol.	No specific focus on specialists or the professions
6RXY5HVL	2022	Tegally, Houriiyah;	The evolving SARS-CoV-2 epidemic in A	Science	No specific focus on specialists or the professions
4IZLB6WP	2024	Tegegne, Yibeltal S	Incidence and development of validate	BMC Pediatr.	No specific focus on specialists or the professions
HBI96NST	2021	Teheux, Lara; Cooli	Intraprofessional workplace learning in	BMC Med. Educ.	No specific focus on specialists or the professions
QQYC6SFV	2019	Tekelab, Tesfalidet	Factors affecting utilization of antenata	PLoS One	No specific focus on specialists or the professions
R5AQIJLI	2014	Teklu, Sisay; Azazh,	Development and implementation of ar	Ethiop. Med. J.	No specific focus on specialists or the professions
YRNAHU4X	2024	Terefe, Felagot Tad	Advancing Antenatal Care in Ethiopia: T	Telemed. e-Health	No specific focus on specialists or the professions
S5JRX74E	2013	Terreri, M T; Campo	Profile of paediatric rheumatology spec	Rev. Bras. Reumatol.	
RFFKQPHD	2022	Tesfay, Neamin; Ta	Cause and risk factors of early neonata	PLoS One	No specific focus on specialists or the professions
NPG8MMK5	2022	Tesfay, Neamin; Ta	Target areas to reduce the burden of ma	PLoS One	No specific focus on specialists or the professions
DVFADKAI	2022	Testino, Gianni; Vig	Alcohol use disorder in the COVID-19 e	Addict. Biol.	No specific focus on specialists or the professions
JKRDTSMN	2017	Tevlin, Ruth; Dillon	Education in burns: Lessons from the pa	Burns	No specific focus on specialists or the professions
NYGG6JMD	2014	Thienemann, Fried	Rationale and design of the Pan African	BMJ Open	No specific focus on specialists or the professions
BIF7LMJY	2022	Thipanyane, Mvula	Perceptions of Pregnant Women on Trad	Int. J. Environ. Res.	No specific focus on specialists or the professions
I27WB6ME	2022	Thompson-Harvey,	Perceptions of Otolaryngology Residen	Laryngoscope	No specific focus on specialists or the professions
KT4G2YBR	2008	Thornton, Robert J	Unimagined community: Sex, networks, and AIDS in Uganda		No specific focus on specialists or the professions
ISFDSA9G	2021	Thoumi, Andrea; Bo	Policy considerations to promote equit	Ann. Glob. Heal.	No specific focus on specialists or the professions
ASH8UXCD	2010	Tiburcio Sainz, Mar	Mental health services utilization amon	SALUD Ment.	No specific focus on specialists or the professions
3YYP3BGS	2024	Tierney, Aaron A; M	Telemedicine Implementation for Safet	Telemed. e-Health	No specific focus on specialists or the professions
DLBCPI9H	2019	Tilly, Chris	Work under capitalism		No specific focus on specialists or the professions
HAPY355E	2008	Tiwari, Anjali; Rajar	Guideline-consistent antidepressant tr	Psychiatr. Serv.	No specific focus on specialists or the professions
IJ7LFFDH	2021	Tiwari, Ritika; Chik	Estimating the Specialist Surgical Work	Ann. Glob. Heal.	No specific focus on specialists or the professions
2ZKBW7GT	2009	Tobias, Jeffrey; Hoc	Cancer and its Management: Sixth Edition		No specific focus on specialists or the professions
UWHBIHLQ	2020	Tochie, Joel Noutal	Global epidemiology of acute generalis	BMJ Open	No specific focus on specialists or the professions
SSISCUBW	2017	Tochie, Joel Noutal	The neglected burden of snakebites in C	BMC Res. Notes	No specific focus on specialists or the professions
3YHYBBPV	2019	Tomines, Alan	Pediatric Telehealth: Approaches by Sp	Adv. Pediatr.	No specific focus on specialists or the professions
VEF8YPPL	2013	Tomlinson, Jared; H	The burden of surgical diseases on critic	Trop. Doct.	No specific focus on specialists or the professions
5AXYA4T9	2015	Tong, Steven Y C; D	Staphylococcus aureus infections: Epid	Clin. Microbiol. Rev	No specific focus on specialists or the professions
BQKCN52K	2024	Toniolo, Jean; Ngou	Role and knowledge of nurses in the ma	PLoS One	No specific focus on specialists or the professions
4HJEM7AN	2019	Torcetti, Shelley	Living in the Shadows of China's HIV/AIDS Epidemics: Sex, D		No specific focus on specialists or the professions
R3G65IIH	2019	Tosh, Jemma	The body and consent in psychology, psychiatry, and medici		No specific focus on specialists or the professions
U6N335CJ	2023	Toudou-Daouda, M	Epilepsy in Niger: An overview of the cur	Epilepsia open	No specific focus on specialists or the professions
DPDNTSS6	2024	Toumi, Dhekra; Dho	The SBAR tool for communication and p	BMC Med. Educ.	No specific focus on specialists or the professions
T9KTUHAU	2024	Towghi, Fouzieyha	Baloch Midwives: Contesting Global Perceptions of Midwife		No specific focus on specialists or the professions
6GYGJJY9	2023	Toyin-Thomas, Pati	Drivers of health workers' migration, int	BMJ Glob. Heal.	No specific focus on specialists or the professions
9QALLRR3	2024	Tran, Binh Thang; N	Beyond Medical Bills: High Prevalence	J. Prev. Med. & PUBL	No specific focus on specialists or the professions
5YS9XVU4	2015	Tran, Tu M; Saint-Fo	Estimation of Surgery Capacity in Haiti:	World J. Surg.	No info on specialists' contribution to health systems/population health, or their governance
HW9R3HUD	2011	Traore, Abdoulaye;	Perceptions of barriers to training gene	Glob. Health Promot.	
VUP7TZYZ	2019	Treanor, Charlene	Psychosocial interventions for informal	Cochrane Databas	No specific focus on specialists or the professions
QLF6P3Q6	2022	Trebilcock, Michae	Paradoxes of Professional Regulation: In Search of Regulato		No specific focus on specialists or the professions
GLAIJAYJ	2016	Trelles, Miguel; Ste	Averted health burden over 4 years at M	Surg. (United States	No specific focus on specialists or the professions
S7LV8C42	2015	Tricco, Andrea C; A	A scoping review of rapid review metho	BMC Med.	No specific focus on specialists or the professions
VP2SIAZU	2020	Tricco, Andrea C; L	Global evidence of gender inequity in ac	JBI Evid. Synth.	No specific focus on specialists or the professions
37GMYVGH	2023	Tricco, Andrea C; N	Global evidence of gender equity in aca	BMJ Open	No specific focus on specialists or the professions
IYLNXALM	2024	Tricco, Andrea C; P	Interventions on gender equity in the w	BMC Med.	No specific focus on specialists or the professions
KGRLWVR8	2021	Trivedi, Neha; Mos	Predictors of Patient-Centered Commu	J. Health Commun.	No specific focus on specialists or the professions
SKGTPE8M	2020	Truche, Paul; NeMo	Publicly funded interfacility ambulance	PLoS One	No specific focus on specialists or the professions
FRNTVWTG	2020	Truché, Paul; Shom	Globalization of national surgical, obst	Global. Health	
I78T4H5C	2022	Tsao, Connie W; Ad	Heart Disease and Stroke Statistics-20	Circulation	No specific focus on specialists or the professions
HMTM5VJA	2023	Tsao, Connie W; Ad	Heart Disease and Stroke Statistics - 20	Circulation	No specific focus on specialists or the professions
UDI4E58R	2023	Tsao, L; Kwete, X J;	Effect of Training on Physicians' Palliati	J. Pain Symptom Ma	No specific focus on specialists or the professions
IWFPJKET	2022	Tsele-Tebakang, Te	Herb-drug interactions: Perception and	Int. J. Africa Nurs. S	No specific focus on specialists or the professions
HVASNRMR	2024	Tsige, Abate Wonde	Cervical cancer: Challenges and preve	Heal. Sci. Reports	No specific focus on specialists or the professions
W38GYWWH	2024	Tu, Qiang; Lin, Shua	The effects of multidisciplinary collabo	Prim. Care Diabete	No specific focus on specialists or the professions
59WSG6ZK	2018	Tulchinsky, Theodo	Case Studies in Public Health		No specific focus on specialists or the professions
Y92FFZMN	2018	Tungland, Bryan	Human microbiota in health and disease: From pathogenes		No specific focus on specialists or the professions
QIH6WISR	2023	Turan, Janet M; Vin	Global health reciprocal innovation to a	BMJ Glob. Heal.	No specific focus on specialists or the professions
7GSW987Z	2016	Turner, J Rick; Karn	Cardiovascular safety in drug development and therapeutic		No specific focus on specialists or the professions
VUFGQVNN	2018	Turner, Sandra; Ch	Discipline-specific competency-based	Leadersh. Heal. Serv.	
ZXCL4Q7X	2014	Tutino, G E; Tam, W	Diabetes and pregnancy: Perspectives	Diabet. Med.	No specific focus on specialists or the professions
RGFZ6LFT	2014	Tyson, Anna F; Msis	Delivery of operative pediatric surgical	Int. J. Surg.	
IHMDXLIG	2021	Ubom, Akaninyene	Nigerian Surgical Trainees' Work Sched	World J. Surg.	No specific focus on specialists or the professions
YP4N98C2	2021	Uddin, Taslim; Islar	2017 Bangladesh landslides: physical re	Disabil. Rehabil.	No specific focus on specialists or the professions
YL3Y5Z3P	2024	Uhrig, Alexander; R	Building a High-Level Isolation Unit in R	Heal. Secur.	No specific focus on specialists or the professions
JIRH2L8M	2021	Ukachukwu, Faith U	Challenges in treating ophthalmia neon	Expert Rev. Ophtha	No specific focus on specialists or the professions
EYF4AC97	2022	Ukoha, Winifred Ch	Preconception care practices among p	Glob. Health Action	No specific focus on specialists or the professions
TEZ6N59M	2023	Ukoha, Winifred Ch	Integration of preconception care into t	Heliyon	No specific focus on specialists or the professions
MWQ86D54	2022	Ul Haq, Muhammad	Striking the Right Note: Assessing the R	Turkish J. Anaesthes	No specific focus on specialists or the professions
Q37FUWL5	2017	Ulijaszek, Stanley J	Models of Obesity: From Ecology to Complexity in Science a		No specific focus on specialists or the professions
F2GQLAS3	2016	Ulisubisya, Mpoki;	Establishing an Anaesthesia and Intens	Global. Health	No specific focus on specialists or the professions
IWVHVYHS	2019	Umar, Nasir; Wickr	Understanding mistreatment during ins	Reprod. Health	No specific focus on specialists or the professions
KQDFWVQR	2019	Umemura, Satoshi;	The Japanese Society of Hypertension G	Hypertens. Res.	No specific focus on specialists or the professions
HATJGMTT	2022	Umoren, Rachel	Simulation and Game-Based Learning for the Health Profes		No specific focus on specialists or the professions
CRHTCPW6	2010	Unger, Jean-Pierre;	International health and aid policies: The need for alternati		No specific focus on specialists or the professions
TT8MAGU5	2023	Unler, Mehmet; Ert	Completed physician and medical stud	DUSUNEN ADAM-JO	No specific focus on specialists or the professions
6S6ZI5ZU	2019	Unnithan, Maya	Fertility, health and reproductive politics: Re-imagining righ		No specific focus on specialists or the professions
VX4B2YZX	2016	Uribe-Leitz, Tarsici	Variability in mortality following caesar	Lancet Glob. Heal.	No specific focus on specialists or the professions
CD2WLPY5	2016	Utz, Bettina; De Br	"Why screen if we cannot follow-up and	BMC Pregnancy Ch	No specific focus on specialists or the professions
MZYZC83M	2024	Väänänen, Ari	The rise of mental vulnerability at work: A socio-historical a		No specific focus on specialists or the professions
HZHJZNNZ	2023	Vahos, Juanita; Roj	Barriers of Access to Opioid Medicines	J. Palliat. Med.	No specific focus on specialists or the professions
Q8F7VZWN	2024	Vaisman, Alex; Guil	Over 50% of self-reported burnout amo	J. ISAKOS Jt. Disord	No specific focus on specialists or the professions
6IZ3PW37	2023	Vajravelu, Mary Elle	Pediatric Obesity: Complications and C	Life	No specific focus on specialists or the professions
GVI8PSZ6	2022	Valk-Draad, Maria	Nursing Home-Sensitive Hospitalizatio	Int. J. Environ. Res.	No specific focus on specialists or the professions
RDTYIQU6	2023	Vallely, Lisa M; Cat	Improving maternal and newborn health	Lancet Reg. Heal. -	No specific focus on specialists or the professions
VRZX35GT	2020	van Heemskerken,	Barriers to surgery performed by non-ph	Hum. Resour. Health	
FIGF78PF	2018	van Rensburg, Andr	State and non-state mental health serv	Health Policy Plan.	No specific focus on specialists or the professions
SU78FF3Y	2022	van Rensburg, Bern	Profile of the current psychiatrist workf	Health Policy Plan.	
89H7BPUD	2021	Vanderslott, Samar	Attention and responsibility in global health: The currency o		No specific focus on specialists or the professions
5R9QPRPB	2016	Vardanyan, Ruben;	Synthesis of Best-Seller Drugs		No specific focus on specialists or the professions
ID67ZK8V	2010	Vargas-Lombardo,	Scope of information communications t	Diabetes Technol. T	No specific focus on specialists or the professions
5658DDJ7	2018	Vargas, I; Garcia-Su	Understanding communication breakdo	Health Policy Plan.	No specific focus on specialists or the professions
HQVZDB83	2020	Vargas, Ingrid; Egui	Can care coordination across levels be	BMC Health Serv. Res.	
TDEJTXBR	2001	Vassallo, D J; Hoqu	An evaluation of the first year's experien	J. Telemed. Telecar	No specific focus on specialists or the professions
2SVD6TA4	2021	Vaughan, Geraldine	Caring for Pregnant Women with Rheun	Glob. Heart	No specific focus on specialists or the professions
D364PTAN	2022	Vedam, Saraswath	Advancing quality and safety of perinata	Health Policy Plan.	No specific focus on specialists or the professions
U5JZJPXQ	2017	Velazquez, Maurici	Evaluation of the teleconsultation proc	Rev. Panam. SALUD	No specific focus on specialists or the professions
VENGN8SF	2023	Velleca, Angela; Sh	The International Society for Heart and	J. Hear. Lung Transp	No specific focus on specialists or the professions
XVA35J5Y	2011	Velu, Prasad Palani	Epidemiology and aetiology of materna	J. Glob. Health	No specific focus on specialists or the professions
VYH73IGL	2018	Venkatesh, Bala; M	Women in Intensive Care study: a prelir	Crit. Care	No specific focus on specialists or the professions
SBLQEI67	2022	Verduzco-Aguirre,	Barriers and Facilitators for the Implem	JCO Glob. Oncol.	No specific focus on specialists or the professions
EHXGP2N4	2015	Verguet, Stéphane;	Timing and cost of scaling up surgical se	Lancet. Glob. Heal.	No specific focus on specialists or the professions
7JWLCQ9N	2020	Vermund, Sten H; C	Sexually transmitted infections		No specific focus on specialists or the professions
AIXTHL95	2023	Versluis, Marco; Oz	Applicability of working abroad for phys	Global. Health	No specific focus on specialists or the professions
YZ7WLDEH	2024	Vervoort, Dominiqu	Barriers to Access to Cardiac Surgery: C	Can. J. Cardiol.	No specific focus on specialists or the professions
AB7MK2ZH	2023	Vervoort, Dominiqu	Tertiary prevention and treatment of rh	BMJ Glob. Heal.	No specific focus on specialists or the professions
NSCGIEAK	2015	Vezyridis, Paraskev	On the adoption of personal health reco	Ethics Inf. Technol.	No specific focus on specialists or the professions

Appendix 4

Table S4: List of papers excluded, with reason

RX4Q8VLP	2021	Vicencio, Daniel	Between exiled and native doctors: Spa	Hist. CIENCIAS SAU	No specific focus on specialists or the professions
L9A35DHU	2020	Vigo, Daniel; Haro,	Toward measuring effective treatment c	Psychol. Med.	No specific focus on specialists or the professions
AJS7AI38	2014	Vijayanarayana, K;	An appraisal of sensitivity and resistanc	Res. J. Pharm. Biol.	No specific focus on specialists or the professions
PDADE5DK	2021	Vilaly, Mohamed Ab	Access to paediatric surgery: the geogra	BMJ Glob. Heal.	
9YSLA4QD	2023	Vilendrer, Stacie; L	Physician Perceptions of Performance F	Jt. Comm. J. Qual. P	No specific focus on specialists or the professions
NXPSD64J	2021	Villarreal-Zegarra,	Development of the set of scales to ass	BMC Public Health	No specific focus on specialists or the professions
WSZD7ATA	2018	Vincent-Lambert, C	Challenges relating to the inter-facility	African J. Emerg. Me	No specific focus on specialists or the professions
DZ4VIH4F	2016	Vinekar, Anand; Jay	Telemedicine in Retinopathy of Premat	Adv. Ophthalmol. O	No specific focus on specialists or the professions
PWFRQZ32	2006	Vio, F	Management of expatriate medical ass	Hum. Resour. Health	
BGB2LR5A	2020	Virani, Salim S; Alon	Heart disease and stroke statistics—20	Circulation	No specific focus on specialists or the professions
9GJQR6PT	2023	Virani, Salim S; Nev	2023 AHA/ACC/ACCP/ASPC/NLA/PCNA	Circulation	No specific focus on specialists or the professions
PY9QQN5W	2021	Virk, Amrit; Bella Ja	What factors shape surgical access in V	BMJ Open	No specific focus on specialists or the professions
YTTQW4VV	2019	Vishnevetsky, Anas	Palliative Care: Perceptions, Experienc	J. Palliat. Med.	No specific focus on specialists or the professions
6X9M8MTD	2022	Visseren, Frank L J;	2021 ESC Guidelines on cardiovascular	Eur. J. Prev. Cardiol	No specific focus on specialists or the professions
5QP6VSD3	2021	Visseren, Frank; Ma	2021 ESC Guidelines on cardiovascular	Eur. Heart J.	No specific focus on specialists or the professions
V6YMMYNC	2024	Viswanathan, Shan	Multi-actor system dynamics in access	Mult. Scler. Relat. D	No specific focus on specialists or the professions
YM5EIPGT	2021	Vitalis, Debbie	Adherence to Antiretroviral Therapy among Perinatal Wome		No specific focus on specialists or the professions
5AHRXQBL	2021	Vogel, Birgit; Aceve	The Lancet women and cardiovascular c	Lancet	No specific focus on specialists or the professions
3NUU5JV5	2020	Voss, Miranda; Swa	Capacity-building partnerships for surg	Health Policy Plan.	No specific focus on specialists or the professions
YWMKILHM	2023	Votruba, Nicole; Pr	SMARThealth PRegnancy And Mental H	Front. Glob. Womer	No specific focus on specialists or the professions
D6GNCGYU	2008	Vukasinovic, Zoran	ORTHOPEDIC AND TRAUMA SERVICES	Srp. Arh. Celok. Lek	No specific focus on specialists or the professions
NB8QGCYS	2016	Vyas, Dinesh; Hollis	Prehospital care training in a rapidly de	J. Surg. Res.	No specific focus on specialists or the professions
9SYHI375	2018	Wade, A J; Doyle, J S	Community-based provision of direct-a	Trials	No specific focus on specialists or the professions
FXUCHWHX	2021	Wakefield, Mary K;	The future of nursing 2020-2030		No specific focus on specialists or the professions
F6YTJ59Q	2009	Walker, Isabeau A	Con: Pediatric anesthesia training in de	Paediatr. Anaesth.	No specific focus on specialists or the professions
7D7RKWBK	2010	Walker, Isabeau A;	Paediatric surgery and anaesthesia in s	Bull. World Health (	No specific focus on specialists or the professions
SA6VBJZJ	2022	Walkowiak, Darius;	The Awareness of Rare Diseases Among	Front. public Heal.	No specific focus on specialists or the professions
PJ2ZKWGR	2018	Wall, L Lewis	Tears for my sisters: The tragedy of obstetric fistula		No specific focus on specialists or the professions
Q2FVU539	2012	Wall, L Lewis	Preventing obstetric fistulas in low-reso	Obstet. Gynecol. Su	No specific focus on specialists or the professions
NYM7TUAS	2008	Wallace, Dana V; D	The diagnosis and management of rhinit	J. Allergy Clin. Immu	No specific focus on specialists or the professions
BWVRAFSW	2018	Wallis, Christophe	Personal prostate-specific antigen scre	Can. Urol. Assoc. J.	No specific focus on specialists or the professions
5T4NVYX5	2016	Walsh-Childers, Ki	Mass media and health: Examining media impact on individu		No specific focus on specialists or the professions
369N4L3T	2017	Walton-Roberts, M	Causes, consequences, and policy resp	Hum. Resour. Healt	No specific focus on specialists or the professions
CS8I4YBC	2022	Wang, Bo; Feldman	Mental health services in Scandinavia a	Int. Rev. Psychiatry	No specific focus on specialists or the professions
MNC2HJ2L	2021	Wang, Kailu; Guo, X	Patient-Identified Problems and Influen	Front. Endocrinol. (	No specific focus on specialists or the professions
Y8G8PEJ8	2023	Wang, Shuangwen;	The role of different educational progra	BMC Med. Educ.	No specific focus on specialists or the professions
UCI5WEYN	2022	Wang, Xia; Carcel,	Blood Pressure and Stroke: A Review of	Stroke	No specific focus on specialists or the professions
433YAGSY	2023	Wang, Xin; Jia, Yum	Nuclear receptor coactivator 6 (NCoA6	Cancer Med.	No specific focus on specialists or the professions
D4T43564	2015	Wang, Yi-Xiang J; Li	AME survey-003 A1-part1: in current Ch	Quant. Imaging Med	No specific focus on specialists or the professions
ZQU7BVLE	2024	Wang, Youping; Wa	Patterns and characteristics of visits to	Eur. Arch. Psychiatr	No specific focus on specialists or the professions
FP3GZ3SG	2022	Wang, Zhiguo; Yang	Polypharmacology: Principles and methodologies		No specific focus on specialists or the professions
ZHH7XFCT	2022	Wanitphakdeedech	A Cross-Sectional Study on Factors Affe	Dermatol. Ther. (He	No specific focus on specialists or the professions
9ZUIPXLK	2019	Ward, Martha C	Poor women, powerful men: America's great experiment in f		No specific focus on specialists or the professions
TTG48JND	2023	Warren, Annabelle	Syndrome of Inappropriate Antidiuresis	Endocr. Rev.	No specific focus on specialists or the professions
KEWZ9938	2011	Warriner, I K; Wang	Can midlevel health-care providers adr	Lancet	No specific focus on specialists or the professions
C6MK46J8	2020	Wasserman, Isaac;	Breaking specialty silos: Improving glob	Glob. Heal. Sci. Pra	No specific focus on specialists or the professions
UX6P8QN6	2020	Watts, Gerald F; Gi	Familial hypercholesterolaemia: evolvi	Nat. Rev. Cardiol.	No specific focus on specialists or the professions
CJGXPCEU	2011	Watts, Gerald F; Su	Familial hypercholesterolaemia: A mod	Atheroscler. Suppl.	No specific focus on specialists or the professions
HIT2KQP8	2016	Watts, Jacqueline I	Gender, health and healthcare: Women's and men's experie		No specific focus on specialists or the professions
6MTMVVBE	2021	Webair, Hana Hasa	Patient-centred infertility care among A	BMJ Open	No specific focus on specialists or the professions
3GJWENPJ	2024	Webb, Rebecca; Fo	Conceptual framework on barriers and	Heal. Soc. Care Del	No specific focus on specialists or the professions
PHK2LLBR	2012	Weber, David J; Ru	Vaccines for health care personnel		No specific focus on specialists or the professions
N5E9A8EL	2019	Webster, Jessica; S	Canadian maternity and pediatric nursing		No specific focus on specialists or the professions
AHLR4VX7	2006	Weeks, William B; V	Association of race and gender with ger	J. Am. Coll. Surg.	No specific focus on specialists or the professions
EGU2BNP9	2022	Wei, Yue; Yan, Vinc	Association of Long-Acting Injectable Ar	JAMA Netw. open	No specific focus on specialists or the professions
S8RPT5G6	2014	Weinhold, Ines; Gu	Understanding shortages of sufficient h	Health Policy (New	No specific focus on specialists or the professions
6W2TYD8C	2013	Weiser, Thomas G;	Safety in the operating theatre - A trans	Nat. Rev. Urol.	No specific focus on specialists or the professions
FCTMHSEU	2020	Weiss, Gregory L; C	The sociology of health, healing, and illness		No specific focus on specialists or the professions
LX3X8F8S	2015	Weiss, Gregory L; L	Sociology of health, healing, and illness		No specific focus on specialists or the professions
EYX85VPL	2020	Weiss, Scott L; Pete	Surviving sepsis campaign internationa	Intensive Care Med	No specific focus on specialists or the professions
AWF2PHMH	2014	Weisz, George	Chronic disease in the twentieth century: A history		No specific focus on specialists or the professions
UNU5V6QW	2016	Wekesah, Frederic	Effective non-drug interventions for imp	Syst. Rev.	No specific focus on specialists or the professions
EV74Q4ZL	2018	Welcome, Menizib	Gastrointestinal physiology: Development, principles and m		No specific focus on specialists or the professions
KP3RN9VL	2022	Weld, Ethel D; Bail	Ethical issues in therapeutic use and re	Br. J. Clin. Pharmac	No specific focus on specialists or the professions
2AZHFQB6	2003	Westhoff, Carolyn	Depot-medroxyprogesterone acetate in	Contraception	No specific focus on specialists or the professions
PLJ5BVJA	2018	Whetten, Justin; va	Cost-effectiveness of Access to Critica	J. Med. Econ.	No specific focus on specialists or the professions
RSJPU2Z7	2017	White, Russell E; Pa	Delivery of subspecialty surgical care in low-resource settin		No specific focus on specialists or the professions
7H7IB32B	2022	Whiting, Danielle; S	Female urinary incontinence in sub-Sal	BJU Int.	No specific focus on specialists or the professions
G44XT58W	2024	Whittaker, Andrea;	Access to assisted reproductive techno	Sex. Reprod. Heal. r	No specific focus on specialists or the professions
ILWT5A4C	2021	Whop, Lisa J; Smith	Achieving cervical cancer elimination a	Prev. Med. (Baltim)	No specific focus on specialists or the professions
BKNZ5UBD	2009	Widge, Anjali; Clela	The public sector's role in infertility mar	Health Policy Plan.	No specific focus on specialists or the professions
3D2QD239	2010	Wildschut, A	Exploring internal segregation in the So	J. Work. Learn.	No specific focus on specialists or the professions
ZUHL9XQH	2012	Wilhelm, T J; Mothe	Gastrointestinal endoscopy in a low bu	Endoscopy	No specific focus on specialists or the professions
B7Z3PQ32	2021	Willemsen, Antonia	Status of palliative care education in M	Palliat. Support. Ca	No specific focus on specialists or the professions
433U5C3R	2018	Williams, Bryan; M	2018 ESC/ESHGuidelines for themanag	J. Hypertens.	No specific focus on specialists or the professions
LPD9GH8M	2018	Williams, Christop	Cancer and AIDS: Part III: Presentation and management		No specific focus on specialists or the professions
JHGX3RHL	2016	Williams, K M; Ahn,	The incidence, mortality and timing of P	Bone Marrow Trans	No specific focus on specialists or the professions
95Z8RCSK	2024	Williams, Lee Ann F	Fundamentals of case and caseload management: Skills for		No specific focus on specialists or the professions
IT7JCQGR	2020	Willis, Evan	Medical dominance: The division of labour in Australian hea		No specific focus on specialists or the professions
V8F54FPN	2016	Wilmshurst, J M; M	The African pediatric fellowship progran	Pediatrics	No specific focus on specialists or the professions
5XMS8WAM	2018	Wilson, Michael L;	Access to pathology and laboratory med	Lancet	No specific focus on specialists or the professions
JVRCJ77Q	2016	Wilunda, Calistus;	Barriers to Institutional Childbirth in Ru	PLoS One	No specific focus on specialists or the professions
QCJGKBA2	2018	Winston, Karin; Gre	Video-based patient decision aids: A sc	Patient Educ. Coun	No specific focus on specialists or the professions
XRHNU3VS	2024	Winter, Sebastian I	National plans and awareness campaig	Lancet. Glob. Heal.	No specific focus on specialists or the professions
3TUTAHDS	2015	Wissow, Lawrence	Collaboratively reframing mental healt	Health Policy Plan.	No specific focus on specialists or the professions
PRN4L5X5	2010	Wiwanitkit, Viroj; S	Emerging infectious diseases in india		No specific focus on specialists or the professions
QBFPAV8H	2019	Woldemichael, Abr	Inequalities in healthcare resources an	BMJ Open	No specific focus on specialists or the professions
3CW57XGC	2018	Woldie, Mirkuzie; F	Community health volunteers could he	Health Policy Plan.	No specific focus on specialists or the professions
A7KZ9S37	2018	Wolf, Jacqueline H	Cesarean Section: An American History of Risk, Technology,		No specific focus on specialists or the professions
ZE48Q3X4	2021	Wolfenden, Luke; F	Designing and undertaking randomised	BMJ	No specific focus on specialists or the professions
82FNDUUU	2018	Wong, D J N; Harris	Cancelled operations: a 7-day cohort st	Br. J. Anaesth.	No specific focus on specialists or the professions
KL2Y8IKC	2024	Wong, Jie Jun; Wan	Current frailty knowledge, awareness, a	Eur. Hear. J. open	No specific focus on specialists or the professions
3DRU7VLT	2024	Wong, Judith Ju Mir	Lung-Protective Ventilation for Pediatri	Crit. Care Med.	No specific focus on specialists or the professions
V3MJF55P	2021	Wong, Sarah Hui M	‘Decolonising the Medical Curriculum‘	London Rev. Educ.	No specific focus on specialists or the professions
NAWAHF48	2018	Wong, Xin Yi; Groot	Women's preferences, willingness-to-p	Patient Prefer. Adhe	No specific focus on specialists or the professions
P859QVAP	2015	Wong, Zhi Y; Hassa	Medical specialists' knowledge, percep	J. Generic Med.	No specific focus on specialists or the professions
B7QZ7ULF	2018	Woodrow, Philip	Intensive care nursing: A framework for practice, fourth Edit		No specific focus on specialists or the professions
7D5ZZMM5	2021	Woodruff, Teresa K	A View from the past into our collective	J. Assist. Reprod. Ge	No specific focus on specialists or the professions
ENLXFC7E	2024	Woods, Charles R;	Clinical Practice Guideline by the Pedia	J. Pediatric Infect. D	No specific focus on specialists or the professions
4RRAQTQQ	2018	Woodward, Aniek; I	Specialist training aspirations of junior	BMC Med. Educ.	No specific focus on specialists or the professions
P6I36YFQ	2005	Woodward, K N	The potential impact of the use of home	Hum. Exp. Toxicol.	No specific focus on specialists or the professions
4BHS3WCN	2013	Woodward, Kevin	Toxicological effects of veterinary medi	Issues Toxicol.	No specific focus on specialists or the professions
H76Q7BZQ	2019	Woolley, Torres; La	Career choices of the first seven cohort	Rural Remote Healt	No specific focus on specialists or the professions
2F63DWSV	2024	Woss, Joana Lucilia	The perception of clinical professionals	Observatorio	No specific focus on specialists or the professions
HXQRILLK	2017	Wu, Dan; Lam, Tai P	Challenges to healthcare reform in Chi	Health Policy Plan.	
FHY8KFZD	2018	Wu, Jingxian	Measuring inequalities in the demograp	Int. J. Health Plann.	No specific focus on specialists or the professions

Appendix 4

Table S4: List of papers excluded, with reason

DVI3J7YQ	2019	Wu, Jingxian; Yang,	Inequality trends in the demographic ar	Int. J. Health Plann.	No specific focus on specialists or the professions
R3IR5CAV	2024	Wu, Yuhan; Stratin	Prevalence, risk factors, consequences	Aggress. Violent Bel	No specific focus on specialists or the professions
UHUD4XBF	2017	Wynn, L M; Foster,	Abortion pills, test tube babies, and sex toys: Emerging sexu		No specific focus on specialists or the professions
MHEN3EIT	2023	Xiao, Leon Y; Hend	What are the odds? Poor compliance w	PLoS One	No specific focus on specialists or the professions
B5YM76P4	2024	Xiao, Yu; Liu, Liang;	Sexual Harassment Among Chinese Psy	Risk Manag. Health	No specific focus on specialists or the professions
ZGDC8GD8	2022	Xie, Jiaying; Tang, S	Efficacy of psychosocial interventions f	Ann. Palliat. Med.	No specific focus on specialists or the professions
8FI6G9LE	2022	Xie, Jinzhao; Fan, X	Association between childhood friends	BMC Geriatr.	No specific focus on specialists or the professions
FKLRNZ7J	2023	Xie, La; Feng, Mei; (	Developing a core competency training	BMJ Open	No specific focus on specialists or the professions
3EN9VG7R	2014	Xing, Weijia; Liao, (	Hand, foot, and mouth disease in China	Lancet. Infect. Dis.	No specific focus on specialists or the professions
XA93K98U	2011	Xirasagar, Sudha; H	Colonoscopy screening rates among pa	Cancer	No specific focus on specialists or the professions
XY3WIZ3U	2022	Xu, Li; You, Dingyur	Two-stage mental health survey of first-	Eur. Arch. Psychiatr	No specific focus on specialists or the professions
QTII55TR	2023	Xu, Nini; Deng, Sich	Impact of migration on oral health outc	BMC Oral Health	No specific focus on specialists or the professions
68GWP4V4	2008	y Huesca, Andres; F	Surgical exercise in the mexica empire	Rev. Investig. Clin. T	No specific focus on specialists or the professions
54J8L7KB	2024	Yakubu, Aliu; Moral	Evaluating the knowledge of stroke man	J. stroke Cerebrova	No specific focus on specialists or the professions
4L64UCTV	2010	Yam, Carrie H K; W	Avoidable readmission in Hong Kong–sy	BMC Health Serv. R	No specific focus on specialists or the professions
N67EYVY2	2017	Yamaguchi, Takash	Palliative care development in the Asia-	BMJ Support. Pallia	No specific focus on specialists or the professions
DKUUK3WC	2016	Yamin, Alicia Ely; F	Power, suffering, and the struggle for dignity: Human rights f		No specific focus on specialists or the professions
TFNS73K8	2023	Yan, Vincent Ka Ch	Effectiveness of BNT162b2 and Corona	Emerg. Microbes In	No specific focus on specialists or the professions
D4NQAU75	2012	Yan, Xixi; Liu, Tiany	Attitudes of physicians, patients, and vi	Arch. Ophthalmol. (	No specific focus on specialists or the professions
YVFUTYZQ	2023	Yan, Yulin; Jiang, W	Evaluation of a computer-aided diagnos	Front. Med.	No specific focus on specialists or the professions
ULG9WFUF	2023	Yánez, Diego Busta	A Theoretical Journey on Health and Ed	Lect. Notes Networ	No specific focus on specialists or the professions
YT4YMX7M	2016	Yang, L	Acute Kidney Injury in Asia	KIDNEY Dis.	No specific focus on specialists or the professions
SKPPR72A	2023	Yang, Lin; Zheng, R	The burden of appendicitis and surgical	J. Infect. Dev. Ctrie	No specific focus on specialists or the professions
NJCXFVLH	2023	Yang, Xiangyun; Lu	Characteristics and economic burden	Ann. Gen. Psychiatr	No specific focus on specialists or the professions
3HKMVEXT	2021	Yao, Hao; Wang, Pe	Burnout and job satisfaction of psychia	BMC Psychiatry	No specific focus on specialists or the professions
WNE6HYI2	2013	Yao, Nengliang; Ma	Radiation therapy resources and guidel	Health Serv. Res.	No specific focus on specialists or the professions
TCR8BICN	2021	Yap, Adrian Ujin; C	Comparison of emotional disturbance,	Clin. Oral Investig.	No specific focus on specialists or the professions
NBV9LQQ2	2022	Yap, Adrian Ujin; Zh	Comparison of psychological states and	J. Oral Rehabil.	No specific focus on specialists or the professions
NGGKW4J2	2008	Yates, M; Low, W.-Y	Physician attitudes to the concept of 'm	J. Mens. health	No specific focus on specialists or the professions
KLIFTZJD	2020	Yavuz, Serap Şimşe	Diagnosis, treatment and prevention of	Turk Kardiyol. Dern.	No specific focus on specialists or the professions
J2XB33LL	2022	Yeh, Ping Teresa; R	Self-monitoring of blood pressure amon	BMC Pregnancy Ch	No specific focus on specialists or the professions
9ZNPBM8D	2019	Yellamaty, Victoria	General practitioners with special inte	Aust. J. Gen. Pract.	No specific focus on specialists or the professions
JM4Y5THF	2021	Yeung, Emily; Bello	Current status of health systems financ	BMJ Open	No specific focus on specialists or the professions
GAYC4CEG	2020	Yeung, Suet Ying; N	Fertility preservation in Hong Kong Chir	BMC Womens. Hea	No specific focus on specialists or the professions
T9BTXA35	2022	Yilmaz, Mehmet; S	Quality and utility of YouTube videos ab	Urolithiasis	No specific focus on specialists or the professions
4CU4R72A	2013	Ying-ying, Su; Miao	An overview of neurocritical care in Chi	Chin. Med. J. (Engl)	No specific focus on specialists or the professions
HAFPR286	2023	Yoon, Sungwon; Go	Socioeconomic and behavioral determ	Sci. Rep.	No specific focus on specialists or the professions
UC5XCM53	2020	Yoong, Sze Lin; Hal	Nudge strategies to improve healthcare	Implement. Sci.	No specific focus on specialists or the professions
8TMLFTQD	2018	Yore, Mackensie A;	Characteristics and outcomes of pedia	Int. J. Emerg. Med.	No specific focus on specialists or the professions
5QSS69LK	2017	Yoshiura, Vinicius T	A web-based information system for a r	Int. J. Ment. Health	No specific focus on specialists or the professions
CB47RPPR	2016	Young, Gerald	Unifying causality and psychology: Being, brain, and behavio		No specific focus on specialists or the professions
A6Q9Z4CD	2022	Young, Michael J	Neuroethics in the Era of Teleneurology	Semin. Neurol.	No specific focus on specialists or the professions
Q4A3S62X	2016	Young, S; Banza, L;	The impact of long term institutional co	Springerplus	No specific focus on specialists or the professions
BNBPXMBS	2015	Yu, Foo Qing; Muru	Meta-synthesis exploring barriers to he	Asian Pacific J. Can	No specific focus on specialists or the professions
WKP9FJ65	2022	Yuan, Xiaoxia; Xiao	Identification and validation of PGLS as	J. Clin. Lab. Anal.	No specific focus on specialists or the professions
EGSV7W4N	2024	Yuliyanti, Suryani;	A mixed-method analysis of provider ad	PLoS One	No specific focus on specialists or the professions
XTV8PWMA	2018	Yusvirazi, Liga; Ran	State of emergency medicine in Indone	Emerg. Med. Austra	No specific focus on specialists or the professions
4MLLL9AR	2009	Zachariah, R; Ford,	Task shifting in HIV/AIDS: opportunities	Trans. R. Soc. Trop.	No specific focus on specialists or the professions
3AHCEB2R	2023	Zadey, Siddhesh; Iy	Evaluating the status of the Lancet Com	Lancet Reg. Heal. S	No specific focus on specialists or the professions
65VNM42L	2024	Zadey, Siddhesh; R	Achieving Surgical, Obstetric, Trauma, a	Front. Public Heal.	No specific focus on specialists or the professions
ZKEPFQFX	2023	Zahroh, Rana Islam	Educational interventions targeting pre	BMC Public Health	No specific focus on specialists or the professions
4QDDBELR	2020	Zaidi, Mussaret Ba	The Growing Threat of Vaccine Resistar	Curr. Treat. OPTION	No specific focus on specialists or the professions
Q6QDNLRG	2018	Zaka, Nabila; Alexa	Quality improvement initiatives for hos	Implement. Sci.	No specific focus on specialists or the professions
JE8QGS3N	2020	Zalan, Abdelnaser	Genetic counseling (GC) in the Arab society of Israel		No specific focus on specialists or the professions
U62NWZTC	2021	Zaman, Sumbul; An	Work-life enablers for job satisfaction i	Ind. Commer. Train.	No specific focus on specialists or the professions
QPSC6ASX	2020	Zanetto, Alberto; S	Perioperative management of antithror	Best Pract. Res. Cli	No specific focus on specialists or the professions
MVHR7CD9	2024	Zarei, Fatemeh; De	ChecKAP: A Checklist for Reporting a Kr	Asian Pac. J. Cance	No specific focus on specialists or the professions
AP27EL9U	2024	Zavaleta-Monestel	Effects of clinical interventions through	Explor. Res. Clin. So	No specific focus on specialists or the professions
Y5E2AJ7Z	2023	Zhang, Cevin	A Literature Study of Medical Simulatio	Int. J. Environ. Res.	No specific focus on specialists or the professions
RXVBZP4U	2022	Zhang, Huanyu; Xia	Physicians' knowledge on specific rare	Orphanet J. Rare Di	No specific focus on specialists or the professions
MXQ8XYBX	2020	Zhang, Jiaxing; Li, X	The Effectiveness of Clinical Pharmacis	Front. Pharmacol.	No specific focus on specialists or the professions
YHPKI3PX	2022	Zhang, Junyue; Lu,	The influence of telemedicine on capac	Clin. eHealth	No specific focus on specialists or the professions
RS9H6Z9G	2024	Zhang, Kun; Huang,	Telemedicine in Improving Glycemic Co	J. Med. Internet Res	No specific focus on specialists or the professions
9PKDDAPN	2024	Zhang, Mengjia; Su	Resilience mediates the prediction of a	Front. psychiatry	No specific focus on specialists or the professions
KMV9AKTX	2023	Zhang, Mengyue; Si	Exploring the sources of cervical cance	Heal. Expect. an Int	No specific focus on specialists or the professions
LI4G5XZR	2017	Zhang, Ping; Wang,	Impact of organizational and individual	PLoS One	No specific focus on specialists or the professions
ZTXRPT8F	2020	Zhang, Shuang; Wa	Exploring Types of Information Sources	J. Med. Internet Res	No specific focus on specialists or the professions
6Z72ZL2U	2019	Zhang, Tao; Liu, Ch	General practice for the poor and speci	International Journal for Equity in Health	
6K96TFFN	2020	Zhang, Xiaoning; M	Competency framework for specialist c	Nurs. Crit. Care	No specific focus on specialists or the professions
JT3SJXQ8	2019	Zhang, Yongjun; Hu	Characteristics and Workload of Pediat	Pediatrics	
IJNRJHXV	2023	Zhang, Yue; Lin, Xin	The impacts of altruism levels on the jol	BMC Med. Educ.	No specific focus on specialists or the professions
CPF5NYDN	2020	Zhang, Yuhui; Coat	Management of heart failure patients w	Eur. J. Heart Fail.	No specific focus on specialists or the professions
XMF7XZNR	2023	Zhang, Zhiying; Zha	Barriers and facilitators of family docto	BMJ Open	No specific focus on specialists or the professions
MTZQBJ2I	2015	Zhanzeng, Feng; Yu	Basic investigation into the present bur	Burns	No specific focus on specialists or the professions
RZ9YN28D	2022	Zhao, Y; Osano, B;	Characterising Kenyan hospitals' suitab	BMJ Open	No specific focus on specialists or the professions
8HA9G2JY	2023	Zheng, Junyao; Lu,	Prevalence and determinants of defens	Int. J. Qual. Heal. C	No specific focus on specialists or the professions
6NHUEHKI	2021	Zhong, Adrina; Dar	Ethical, social, and cultural issues relat	Genet. Med.	No specific focus on specialists or the professions
A8TJBEYY	2021	Zhu, Xiangjia; Du, Y	Ophthalmic services in Shanghai 2017:	BMC Health Serv. R	No specific focus on specialists or the professions
Z65GKBQQ	2005	Ziemer, David C; M	Clinical inertia contributes to poor diab	Diabetes Educ.	No specific focus on specialists or the professions
VV4VR6R7	2021	Zivarpour, Parinaz;	Chitosan as possible inhibitory agents a	Cancer Cell Int.	No specific focus on specialists or the professions
XYNNT75R	2010	Zolfo, Maria; Iglesia	Mobile learning for HIV/AIDS healthcare	AIDS Res. Ther.	No specific focus on specialists or the professions
2PPB9VXC	2019	Żuk, Piotr; Żuk, Paw	Labour migration of doctors and nurses	The Economic and L	No specific focus on specialists or the professions
SDTZ2KGL	2019	Zuniga-Villanueva,	Factors Associated With Knowledge and	J. Palliat. Care	No specific focus on specialists or the professions
NYK8MEVC	2019	Zweigenthal, V E M	Career paths of public health medicine	Front. Public Heal.	No specific focus on specialists or the professions
5P6IP7SB	2019	Zweigenthal, Virgin	Career Paths of Public Health Medicine	Front. public Heal.	No specific focus on specialists or the professions
K5I2WYCW	2019	Zwitter, Matjaž	Medical ethics in clinical practice		No specific focus on specialists or the professions
