# Supplementary material for: Medical specialists in LMICs: a systematic review and best-fit framework synthesis of the evidence on their roles and contribution to health systems
Source: BMJ Glob Health. 2026 Jan 9;11(1):e018905. doi: 10.1136/bmjgh-2025-018905 (PMC12815179; doi:10.1136/bmjgh-2025-018905)
Supplement: online supplemental file 5 [file bmjgh-11-1-s005.docx]

Appendix 5

Table S5: Full evidence table

| **Author** | **Publication Year** | **Specialty** | **Type of evidence** | **Contribution to systems and population health** | **Governance and policies** | **Scarcity of specialists** | **Markets and private sector** | **Other themes** |
| --- | --- | --- | --- | --- | --- | --- | --- | --- |
| Abukmail, Eman; Albarqouni, Loai | 2021 | Medical specialty students | Quantitative |  | A large proportion of participants intend to postgraduate abroad, mainly in surgical specialities. There is a general impression among doctors and the population that training abroad increases the quality of practice. |  | A minority intends never to return to Palestine, while the majority intends to return after a few years of practice abroad. UK is the first preferred destination. |  |
| Akl, E A; Maroun, N; Major, S; Afif, C; Abdo, A; Choucair, J; Sakr, M; Li, C K; Grant, B J B; Schünemann, H J | 2008 | Medical specialty students | Quantitative |  |  |  |  | Intention to train abroad: prevalence of males, singles and those with additional citizenship. More females intend to return than males. There is a prevalence of surgical specialties for training |
| Ameh, Charles A; Meka, Ramya Jyothi; West, Florence; Dickinson, Fiona; Allott, Helen; Godia, Pamela | 2022 | Obstetrician/Gynaecologists | Qualitative | The most frequent highest cadre of skilled healthcare professional involved in the care of women who died were medical officers (n = 277, 38.2%) followed by obstetricians . There was a reduction in deaths from hemorrhage after training offered by specialists in the context of The FIGO IAP project (description below). | Associations of experts creating guidelines, protocols and recommendations (of practices and medications) for the reduction of postpartum hemorrhage mortality in LMICs in Africa and South Asia. |  |  |  |
| Asamani, James Avoka; Christmals, Christmal Dela; Nyoni, Champion N; Nabyonga-Orem, Juliet; Nyoni, Jennifer; Okoroafor, Sunny C; Ahmat, Adam | 2022 | All specialties | Qualitative |  | Authors describe pathways to become a medical specialist in the East and Southern Africa (ESA) countries and present a general description of the training programmes available. Programmes are not evenly distributed across ESA countries (18), with only Kenya, South Africa and Botswana having some prominence. Sspecialisation in general surgery (11 countries) is the most widely available. |  |  |  |
| Ashengo, Tigistu; Skeels, Alena; Hurwitz, Elizabeth J H; Thuo, Eric; Sanghvi, Harshad | 2017 | Surgery & Aenesthesia | Systematic review |  | “We found reports of performance of surgical and anesthesia tasks by non-specialists across low-resource settings in Africa (29 countries), Asia (10 countries), and Central America (1 country). The practice of task sharing is not only widespread but has become the leading mode of surgical provision for emergency and essential surgeries in some regions”. Page 7, lines 1-4 / Table 1 |  |  |  |
| Ashmore, J; Gilson, L. | 2015 | All specialties | Qualitative |  | RWOPS was viewed by policy-makers interviewed at provincial level as a ‘privilege’ and not a ‘right’, meaning it could be withdrawn at any point. Around the time of fieldwork, in fact, RWOPS was entirely banned in another of South Africa’s nine provinces.” Page 4, Results, 2nd paragraph. Doctors did not always ask permission for the dual practice, but they practiced it anyway, even though penalties exist to prevent fraud. For example, in the study, there was no penalties in the public hospital. Page 4, Results, 3rdd paragraph |  | In practice, the private work undertaken by specialists in South Africa is often undertaken in private wards established in the public sector. Page 4, Results, lines 3-5 In terms of dual practice, surgery and anaesthesiology were both the most profitable specialties in private sector. Page 4, Results, lines 9-12 |  |
| Ashmore, John | 2013 | All specialties | Qualitative |  |  |  | Specialists described several advantages/disadvantages of migrating to the private work or staying in the public sector. Financial rewards in the private sector are much higher than in the public sector, but is not the only factor defining where specialists work. On the other hand, public sector offers some advantages as income stability, pension and paid holidays. There is a significant cost of moving to private sector until patients are not frequent. In this case, establishing a GP network seems to have an important role on the continuous supply of patients. |  |
| Atiyeh, Bishara S; Gunn, S William A; Hayek, Shady N | 2010 | Surgery & Aenesthesia | Systematic review | The authors argue that for years, surgeries were not planned in the functioning of health systems considering similarities and disparities between developed or urban areas (with greater assistance) and rural and remote areas (with more precarious access to the health system). In LMICs surgery have been thought to lie outside the scope of public health. | Some Africa countries (Mozambique, Zâmbia, Tanzania and Malawi) have developed surgical training programs for medical officers or medical assistants, where they learn how to conduct procedures such as caesarean section and strangulated hernia. | “Most surgical cases (i.e., abscesses, tropical pyomyositis, incomplete abortion and postpartum hemorrhage, strangulated hernias, obstructed labor) can be managed by unqualified personnel if properly selected and trained on the job.” Page 582, Health care infrastructure and surgical workforce in remote and rural areas |  |  |
| Baatiema, Leonard; de-Graft Aikins, Ama; Sav, Adem; Mnatzaganian, George; Chan, Carina K Y; Somerset, Shawn | 2017 | Emergency Medicine | Qualitative |  | Lack of collaboration/cooperation and communication were identified as a barrier to promote adequate acute stroke care. Page 7, Team collaboration | Lack of specialists was worst in the regional hospitals, where other health professionals consequently experience higher workload and frustration to not being able to give optimal care. Page 6, Limited staff |  |  |
| Badejo, Okikiolu; Sagay, Helen; Abimbola, Seye; Belle, Sara Van | 2020 | All specialties | Qualitative | There are no specialized professionals working in the management of the health system (Page 2, Introduction, 2nd paragraph), only specialist doctors, both on a macro scale (health system) and on a micro scale (e.g. hospitals), thus controlling the activities of all lower hierarchical levels (Page 11, Specialization, 1st paragraph). This dominance of power has led all other professions to join together in a single association against medical centralization (‘Joint Health Sector Union - JOHESU’) (Page 11, Specialization, 2nd paragraph) | Historically, the governance of the health workforce in Nigeria has been through medical dominance, in which specialist doctors occupy the highest hierarchical level among the health professions, have complete work autonomy, are the main responsible for the health system management direction, and exercise power over the other health professions lower in the hierarchy. |  |  | The development of technologies has been responsible for the evolution of other health professions in Nigeria, threatening medical power and dominance. |
| Bandyopadhyay, Soham; Philipo, Godfrey Sama; Bokhary, Zaitun Mohamed; Lakhoo, Kokila | 2024 | Surgery & Aenesthesia | Qualitative |  | In the past two decades, the creation of some national paediatric surgeons’ associations, together with some collaborative efforts established among them, international associations and NGOs improved surgical care for children in Africa, specially by means of increasing training education and infrastructure. |  |  |  |
| Bayat, Mahboubeh; Salehi Zalani, Gholamhossein; Harirchi, Iraj; Shokri, Azad; Mirbahaeddin, Elmira; Khalilnezhad, Roghayeh; Khodadost, Mahmoud; Yaseri, Mehdi; Jaafaripooyan, Ebrahim; Akbari-Sari, Ali | 2018 | All specialties | Quantitative |  | The current strategies used to control DP in Iran include complete ban for full-time geographic (FTG) specialists and partial restrictions using different incentives for non-full-time specialists (e.g. overpayment). Page 2, 3^rd^ paragraph; |  | 48% of public sector specialists were engaged in dual practice. 62% of non FTG specialists engage in DP, despite implementing incentive mechanisms, and despite complete ban, 24% of FTG specialists are engaged DP. DP. Page 4, Results, Status of the study specialists |  |
| Bedoya-Vaca, Rita; Derose, Kathryn P; Romero-Sandoval, Natalia | 2016 | All specialties | Qualitative |  |  |  | The authors claim that public and private sector to be separated in Equador – strong doubts…. | Drivers and feminisation - Authors have not found strong evidence of gender having influenced decisions to pursue medicine / As in other countries, the number of women in medicine schools is increasing in Ecuador. |
| Belrhiti, Zakaria; Belle, Sara Van; Criel, Bart; Van Belle, Sara; Criel, Bart | 2021 | All specialties | Qualitative |  | The study confirmed the existence of professional hierarchies with stratified order in care professions. The hierarchy is defined on the level of expertise, clinical seniority and shared norms and values. In this hierarchy specialists are in the tip, above generalists and the hospital administration, as they define their own rules of work. Also, specialists considered themselves above nurses, who nevertheless try to maintain a cooperative relationship with them. |  |  |  |
| Binyaruka, Peter; Balabanova, Dina; McKee, Martin; Hutchinson, Eleanor; Andreoni, Antonio; Ramesh, Mary; Angell, Blake; Kapologwe, Ntuli A; Mamdani, Masuma | 2021 | All specialties | Quantitative | Moreover, the probability of engaging in informal payment was significantly higher among specialists [AOR ¼ 2.60 (CI: 0.89–7.55)] compared to low-level cadres/seniority (e.g. paramedic, pharmacist, laboratory technicians, etc.). In other words, being a specialist increased the chances of engaging in informal payment by almost three times (and by two times for medical doctors), compared to other providers (e.g. paramedics) (Table 3). | Informal payment is widespread in Africa, including Tanzania, where public health workers receive low salaries. Almost 30% of professionals have received informal payments, and a significant proportion of facilities do not have supervision or electronic control of payments made. Being young (<35 years old), a specialist and being in charge of the department increase the chance of receiving informal payment. On the other hand, the existence of supervision throughout the period and the provision of rights and benefits decreases the chance of receiving informal payment |  |  |  |
| Botezat, Alina; Moraru, Andreea | 2020 | All specialties | Mixed-methods |  |  | Emigration. Romania has a low density of doctors, but it is one of the countries that exports the most doctors, specialists or not, to more developed European countries. | Evidence of drivers of migration among specialists; Most doctors who want to leave the country live in underserved or rural areas. Leaving the country is done through direct contact with employers abroad or through recruitment agencies. The most requested specialties in host countries are cardiology, surgery, psychiatry, radiology, or anaesthetics. |  |
| Brandao, Gabriela R; Motter, Sarah Bueno; Iaroseski, Julia; Trindade, Bruna Oliveira; Brasil, Candida Mozzaquatro de Assis; Rodrigues, Giovanna Severino; de Andrade, Rafaela; da Silveira, Izadora Bouzeid Estacia; de Moraes, Aline Deborah; de Paiva, Marilia Paz | 2024 | Surgery & Aenesthesia | Quantitative | In general, gender inequality within surgical residencies in Brazil is decreasing as the proportion of females entering in training programs are increasing. But disparities remain in some subspecialties such as urology, orthopedic and neurological surgeries | There is an underrepresentation of women in Brazilian surgical societies. |  |  |  |
| Bulamba, F; Bisegerwa, R; Kimbugwe, J; Ochieng, J P; Musana, F; Nabukenya, M T | 2022 | Aenesthesiologists | Mixed-methods |  | Over the past decade, there has been a separation of the previously united bodies representing anaesthetists and non-physician anaesthetist providers (NPAPs) in Uganda. This separation has been seen as positive for the development of both groups and the services they provide, bringing an increased sense of belonging, greater opportunities for development and networking | Despite recent efforts to increase anesthesia services, Uganda still faces a shortage and poor distribution of professionals. Specialist doctors are concentrated in urban centers. |  |  |
| Çalış, Fatih; Şimşek, Abdullah Talha; İnan, Neslihan Gökmen; Topyalın, Nur; Adam, Baha E; Elias, Çimen; Aksu, Muhammed Emin; Aladdam, Mohammed; Gültekin, Güliz; Sorkun, Muhammet Hüseyin; Tez, Müjgan; Balak, Naci | 2024 | Surgery & Aenesthesia | Quantitative |  |  | In 2023, 41% of the 246 spots opened for neurosurgery residency were left vacant. Page e927, 1st paragraph | Financial issues: 55% of the respondents thought that the income of a neurosurgeon was not enough to compensate for the difficulties of the profession. Page e930, Financial issues |  |
| Dalglish, Sarah L; Sriram, Veena; Scott, Kerry; Rodriguez, Daniela C | 2019 | Paediatrics | Qualitative | Specialists usually do not advocate for broader health systems and infrastructural changes. Page 548, Broader health systems and infrastructural changes require the intervention of non-medical actors | Medical relationship networks or specialty associations overlap with other actors in discussions and decisions. Page 547, 1st and 2nd paragraphs in Niger and 3rd paragraph in India. In India, the influence of medical associations outweighs the influence of Ministry of Health. Page 547, 4th paragraph |  |  |  |
| Daniels, Kimberly M; Riesel, Johanna N; Verguet, Stéphane; Meara, John G; Shrime, Mark G | 2020 | Surgery & Aenesthesia | Quantitative | Estimates of the need for surgical services worldwide: 90% of maternal mortality could be averted with timely surgical intervention (Guterres 2016); 32% of the global burden of disease requires surgical decision making (Shrime et al. 2015); 77.2 million disability-adjusted life years (DALYs) could be averted with timely surgical intervention (Bickler et al. 2015); people worldwide without access to safe, timely, affordable surgical care —five billion. |  | Without significant scale-up efforts on global health workforces, and based on the minimum need for surgeons (including surgeons, anesthesiologists, and obstetricians) estimated by The Lancet Commission on Global Surgery (at least 20–40 surgeons/100,000 population), most LMICs will not achieve the proposed 2030 global health development targets |  |  |
| Davies, Justine I.; Vreede, Eric; Onajin-Obembe, Bisola; Morriss, Wayne W. | 2018 | Aenesthesiologists | Quantitative | There was an inverse non-linear relationship between physician anaesthesia providers density and maternal deaths per 100 000 live births, i.e., the greater the number of anesthesia specialists, the lower the maternal mortality rate. Maternal mortality has been used as an indicator of the functioning of a country's health system. 168 countries (98% of all countries) were analyzed |  |  |  |  |
| De Silva, A Pubudu; Liyanage, Isurujith Kongala; De Silva, S Terrance G R; Jayawardana, Mahesha B; Liyanage, Chiranthi K; Karunathilake, Indika M | 2013 | All specialties | Mixed-methods |  | In Sri Lanka, the entire training of a specialist is funded by the government, including two mandatory years of overseas training. The specialist undertakes to return to the country after training and practice for at least four years for each year of overseas training. If he fails to do so, the entire cost must be reimbursed to the government. |  |  |  |
| DeVries, Catherine R | 2022 | Paediatrics | Qualitative | LMICs have the highest birth rates in the world, but in many countries, especially in sub-Saharan Africa, urological pediatricians are absent. Also, the burden of urological diseases is the third highest in the world. There is a large economic impact from not resolving treatable and/or preventable urological surgical problems (e.g., with folic acid supplementation in pregnancy). The search for treatment in these countries is also late, leading to complications. | Pediatric urology is a recent specialty derived from surgery and pediatrics. It has developed well in HICs and MICs, reaching an excess of professionals (including Latin America). However, it is absent in Africa and urological care on the continent is provided by general surgeons or pediatric surgeons. | Considering the population growth rates in this region, the pediatric surgeon deficit in all of Africa, including North and South Africa was >2500 in 2016. Other regions with large total need were in Asia (>5500) where, although the growth rate is leveling off, the baseline population and backlog of surgical cases is great; | There is a current saturation of pediatric surgeons and urological subspecialists in HICs and some MICs. This includes Canada, the UK, the US, and many countries in Latin America and Europe |  |
| Emejulu, J K C | 2008 | Surgery & Aenesthesia | Mixed-methods |  |  | Nigeria has a population of 150 million, and there is only one public and one private training center for neurosurgery in the country. There are few neurosurgeons in practice (1 for every 10 million people), a rate much lower than the average on the African continent |  |  |
| English, M; Strachan, B; Esamai, F; Ngwiri, T; Warfa, O; Mburugu, P; Nalwa, G; Gitaka, J; Ngugi, J; Zhao, Y; Ouma, P; Were, F | 2020 | Paediatrics | Mixed-methods |  | In 2014/2015, based on criteria established by the WHO, the government of Kenya set a target (HR Strategy 2014–2018) to increase the number of doctors (specialists and non-specialists) in the country's public sector. The strategy has not worked, and the gap in doctors in the public sector has increased. | The strategy recommended that by 2030, Kenya should employ 1416 pediatricians in public hospitals. However, in 2009 there were only 305 practicing pediatricians, 1.33 per 100 000 individuals of the population aged <19 years which in total numbers approximately 25 million (50% of Kenya's population is pediatric). Page 928, Medical workforce aspirations | The number of medical schools has increased considerably in the country, producing a number of doctors who would be able to meet the targets, but there has been no concern with the retention of professionals in the public sector. It is estimated that 60% of pediatricians and 40% of non-specialists are employed in the private sector |  |
| English, Mike; Rispel, Laetitia; Ssengooba, Freddie; Edwards, Nigel | 2024 | All specialties | Qualitative | In many LMICs there is an overproduction of specialists, which fragments care and undermines the functions of First Referral Hospitals (FRH), reduce the system’s resilience, and its ability to deal with multi-morbidity | In some LMICs specialists can be found in large First Referral Hospitals, while in other these only represent the door to more sophisticated levels of care |  |  |  |
| Erem, Anna Sarah; Appiah-Kubi, Adu; Konney, Thomas Okpoti; Amo-Antwi, Kwabena; Bell, Sarah G; Johnson, Timothy R B; Johnston, Carolyn; Tawiah Odoi, Alexander; Lawrence, Emma R | 2020 | Obstetrician/Gynaecologists | Case-study | Impact of the lack of specialists. Cervical cancer is the most prevalent cancer in SSA women, diagnostic is at a late-stage and mortality is high (3 times higher than in Europe, even with SSA women having lower chances of developing cancer). It emphasizes the lack of treatment and screening actions. The treatment of gynecological cancer requires specialists to lead diagnosis, imaging, and surgical and oncologic care. | Establishment of an internationally partnered gynecologic oncology training program, using existing infrastructure and partnerships to raise funds and develop treatment and research capacity. Partnership between the Komfo Anokye Teaching Hospital in Ghana and the University of Michigan, US. | The number of oncologists per country in Africa ranges from zero in Togo, Chad, and Burundi, to 1,500 in Egypt. Ghana has only four clinical oncologists. The workload is daunting with 25 countries in Africa reporting an incidence of cancer >1,000 per oncologist. |  |  |
| Falk, Ryan; Taylor, Robert; Kornelsen, Jude; Virk, Roohina | 2020 | Surgery & Aenesthesia | Systematic review | One third of the global burden of disease is surgical in nature, and could/should be tackled through specialists | Task-shifting of basic surgery is already happening in many LMICs, particularly in Sub-saharan African countries |  | Trying to fill the surgery needs gap by training specialists does not work, as these are then attracted by private sector and higher levels of care |  |
| Fitts, Jessica J; Gegbe, Fatmata; Aber, Mark S; Kaitibi, Daniel; Yokie, Musa Aziz | 2020 | Psychiatry | Qualitative |  | General perception that the government is not interested in prioritizing and expanding the provision of mental health services. Lack of leadership | it is suggested a 98% treatment gap for severe mental disorders using global estimates of prevalence |  |  |
| Gajewski, Jakub; Bijlmakers, Leon; Brugha, Ruairí | 2018 | Surgery & Aenesthesia | Qualitative |  | District level hospitals in many African countries deliver only emergency obstetrical interventions, as well as limited general surgery, executed by non-physician clinicians and medical officers without supervision and regulation |  |  |  |
| Gajewski, Jakub; Monzer, Nasser; Pittalis, Chiara; Bijlmakers, Leon; Cheelo, Mweene; Kachimba, John; Brugha, Ruairi | 2020 | Surgery & Aenesthesia | Case-study |  | A task-shifting program was implemented in Zambia in 2011–2016 where surgeons supervise the practice and education of non-physician clinicians in district hospitals. The Clinical Officer Surgical Training in Africa (COST-Africa) project involved intensive training in general surgery (3 months) and quarterly face-to-face supervision and assessment by a surgery specialist | Zambia has two-thirds of the population living in rural areas and there is a shortage of surgeons in district hospitals, especially in rural areas. Surgical services are heavily dependent on the work of non-physician clinicians (known locally as medical licentiates - MLs). The retention of MLs in district hospitals and rural areas is higher. In Zambia, district hospitals are also often affected by shortages of surgical and anaesthetic supplies, and communication channels with higher-level hospitals are limited |  |  |
| Gautam, Bishnu; Sapkota, Vishnu Prasad; Wagle, Rajendra Raj | 2019 | Obstetrician/Gynaecologists | Quantitative |  | Main motivations in order of importance: 1) presence of a complete work team (Gyn-Obs + pediatrician + anesthesiologist); 2) the provision of primary and secondary education for children; 3) the private practice opportunity together with public employment (to increase income |  |  |  |
| Giavina-Bianchi, Mara; Santos, Andre P; Cordioli, Eduardo | 2020 | Dermatology | Quantitative |  | Through teletriage, a large proportion of patients with less complex skin lesions were able to continue treatment in primary care without the need for an in-person consultation with a specialist. In this way, patients with more serious issues were able to be directly and more quickly referred for an in-person consultation with a dermatologist. Another benefit was the option to refer the patients to the biopsy unit before the dermatologist’s visit, optimizing the time available for more severe diagnostics such as skin cancer | The city of São Paulo has nearly 12 million inhabitants, and 58% of them depend exclusively on the public health care system. The demand for public dermatologist consultations in São Paulo is incredibly high, and in July 2017, there were 57,832 individuals waiting for appointments which could take up to one year to obtain |  |  |
| Gong, Yanjun; Huo, Yong; CCCP | 2016 | Cardiology | Quantitative | Unclear what the contribution of cardiologists is in the health system as the training of the majority of these specialists is presented as below the expected standard. | “China only has national medical license certification and interventional cardiology license certification,of which the latter was initiated in 2010, which is a sub-sub-speciality license. But CVD license certification did not exist” Introduction page A2 | Overall, there are 25240 cardiologists in mainland China and the ratio to population is 19 per million.When compared with 25901 actively practicing cardiologists and 55.7 general cardiologists per million population in the USA according to the ACC 2009 cardiologist workforce survey,a scarcity of cardiologists was suggested in China. This survey showed that there are 21.3 cardiologists per 100000 population of 65 years or older, which is close to the ratio in Canada reported in 2005. Discussion A3 |  |  |
| Haastrup, Oluwatosin O O; Buchan, John C; Cassels-Brown, Andy; Cook, Colin | 2015 | Surgery & Aenesthesia | Qualitative |  | There is an absence of official governance systems for the work of ophthalmologists in South Africa. It is expected that specialists will self govern to maintain the perceived quality of services |  |  |  |
| Hagander, Lars E; Hughes, Christopher D; Nash, Katherine; Ganjawalla, Karan; Linden, Allison; Martins, Yolanda; Casey, Kathleen M; Meara, John G | 2013 | Surgery & Aenesthesia | Quantitative | Surgeons go where others are and likely where they will have a lower burden of work in their speciality |  | 56% of respondents came from critical shortage of surgeons countries and the main reason for their departure was the lack of training and career opportunities |  |  |
| He, Fan; Chen, Sijian; Ke, Xiaoyan; Zheng, Yi | 2020 | Psychiatry | Quantitative | Child psychiatrists are not the only medical professionals providing their service to the population as this role is also fulfilled by adult psychiatrists and other physicians | The Chinese authorities have increased the number of training hospitals for CAPs to expand their capacity to train CAPs. | Small number of child and adolescent psychiatrists available but unclear how this compares to set targets. Is the number in comparison with similar sized nations or WHO targets? |  |  |
| Henry, Jaymie Ang; Bem, Chris; Grimes, Caris; Borgstein, Eric; Mkandawire, Nyengo; Thomas, William E G; Gunn, S William A; Lane, Robert H S; Cotton, Michael H | 2015 | Surgery & Aenesthesia | Qualitative | “clinical results of surgery done by trained clinical officers have been shown in several studies, including a meta-analysis, to be as good as their medically qualified counterparts” Pg 827. “Watters and Bayley found that 86.4 % of 21,245 surgical procedures done in eight hospitals in Zambia in a year were not complicated and could be taught to non-surgeons” pg 828 |  | Evidence in the literature of a notable proportion of preventable morbidity |  |  |
| Henry, Jaymie Ang; Frenkel, Erica; Borgstein, Eric; Mkandawire, Nyengo; Goddia, Cyril | 2015 | Surgery & Aenesthesia | Quantitative | Non physician specialists have improved the delivery of service care in the absence of trained specialists | Rather than increase the training specialists exclusively, Malawi is also increasing the training of non-physician specialists | Surgical and anaesthetist specialists are in the minority among those working in hospitals in Malawi. |  |  |
| Hoogland, Romy; Hoogland, Lisa; Handayani, Krisna; Sitaresmi, Mei; Kaspers, Gertjan; Mostert, Saskia | 2022 | All specialties | Systematic review |  |  |  | “PDP-reports were found in 157 countries (81%). No significant difference in prevalence of PDP was found between HIC (77%) and LMIC (82%). Most common reason for working in private sector was low government salaries in public hospitals (55%). This was more reported in LMIC (65%) than HIC (30%; P<0.001).” Abstract. Our study shows that low government salaries, poor working conditions, inadequate facilities, and drugs or equipment shortages in public hospitals are important reasons for working in private sector.” Discussion, Page 1451 |  |
| Hoyler, Marguerite; Finlayson, Samuel R G; McClain, Craig D; Meara, John G; Hagander, Lars | 2014 | Surgery & Aenesthesia | Systematic review |  |  | “the number of surgeons, obstetricians, and anesthesiologists practicing in LMICs represents a small minority of LMICs, and indicates consistently low levels of surgical physicians. Across LMICs, general surgeon density ranged from 0.13 to 1.57 per 100,000 population, obstetrician density ranged from 0.042 to 12.5 per 100,000, |  |  |
| Kakuma, Ritsuko; Minas, Harry; Van Ginneken, Nadja; Dal Poz, Mario R; Desiraju, Keshav; Morris, Jodi E; Saxena, Shekhar; Scheffler, Richard M | 2011 | Psychiatry | Systematic review |  | Task shifting is proposed as a strategy for mitigating specialist shortages | The proportion of psychiatrists has decreased in low income countries between 2005 and 2011. A variety of negative misconceptions about psychiatry have resulted in difficulties recruiting to this speciality alongside brain drain to HIC and an absence of leadership |  |  |
| Karekezi, Claire; El Khamlichi, Abdeslam; El Ouahabi, Abdessamad; El Abbadi, Najia; Ahokpossi, Semevo Alidegnon; Ahanogbe, Kodjo Mensah Hobli; Berete, Ibrahima; Bouya, Soueilem Mohamed; Coulibaly, Oumar; Dao, Ibrahim; Djoubairou, Ben Ousmanou; Doleagbenou, Agbeko Achille Komlan; Egu, Komi Prosper; Ekouele Mbaki, Hugues Brieux; Kinata-Bambino, Sinclair Brice; Habibou, Laminou Mahamane; Mousse, Adio Nabil; Ngamasata, Trésor; Ntalaja, Jeff; Onen, Justin; Quenum, Kisito; Seylan, Diawara; Sogoba, Youssouf; Servadei, Franco; Germano, Isabelle M | 2020 | Surgery & Aenesthesia | Quantitative |  | Training neurosurgeons locally in Sub-saharan countries, as these are more likely to stay in the region |  | Over half of neurosurgeons trained within SSA return to SSA countries for professional practice and are involved in dual practice. Less than 10% work exclusively in private practice. |  |
| Kempthorne, Peter; Morriss, Wayne W; Mellin-Olsen, Jannicke; Gore-Booth, Julian | 2017 | Aenesthesiologists | Quantitative |  | Training availability and duration is inconsistent across regions | Most countries did not meet the minimum ratio of anaesthetists required and supplemented this with non-physician anaesthetists |  |  |
| Klein, A; Berger, T C; Hapfelmeier, A; Schaffert, M; Matuja, W; Schmutzhard, E; Winkler, A S | 2023 | Neurology | Quantitative | The presence of a neurologist improves the occurrence of seizures |  |  |  |  |
| Kruk, Margaret E; Wladis, Andreas; Mbembati, Naboth; Ndao-Brumblay, S Khady; Hsia, Renee Y; Galukande, Moses; Luboga, Sam; Matovu, Alphonsus; de Miranda, Helder; Ozgediz, Doruk; Quiñones, Ana Romàn; Rockers, Peter C; von Schreeb, Johan; Vaz, Fernando; Debas, Haile T; Macfarlane, Sarah B | 2010 | Surgery & Aenesthesia | Quantitative |  |  | In the countries surveyed, surgery is largely provided by non-specialist doctors or non-phyisicians |  |  |
| Lantz, Adam; Holmer, Hampus; Finlayson, Samuel R G; Ricketts, Thomas C; Watters, David A; Gruen, Russell L; Johnson, Walter D; Hagander, Lars | 2020 | Surgery & Aenesthesia | Quantitative |  |  | In low-income countries and lower-middle income countries, the proportion of surgical specialists abroad was 6.0% and 11.0%, compared with 1.2% and 3.0% in upper-middle income countries and HICs. Results Pg 551 “Overall, anesthesiologists and obstetricians had a similar international proportion of migrants to that of the surgeons (3.3% and 3.2% vs 2.8%, P ¼ 519)... The proportion of specialists abroad was not greater for SAOs than for physicians and other medical specialists.” Results Pg 551 |  |  |
| Lara Munoz, Maria del Carmen; Fouilloux, Claudia; Arevalo Ramirez, Minou del Carmen; Santiago Ventura, Yuridia | 2011 | Psychiatry | Mixed-methods | The community should be at the forefront in terms of the practice of psychiatry. From said position, the psychiatrist can: 1. support selfcare and informal care programs, 2. supervise and offer support to primary care providers, 3. provide direct attention to mental health issues requiring specialized attention and 4. act as a link between the different care levels. A psychiatrist's activities within a general hospital allow: 1. integration with medicine, 2. the appropriate management of non-psychiatric co-morbidities due to more available resources and 3. blunting of stigmatization. Abstract |  |  |  |  |
| Luboga, Samuel; Galukande, Moses; Ozgediz, Doruk | 2009 | Surgery & Aenesthesia | Qualitative | In Uganda specialist surgeons are deployed at the National Referral and Teaching Hospital and the Regional Referral Hospitals, but they do not exist at lower level units such as district hospitals. Specialist surgeons are expected to perform difficult operations that Medical Officers (general physicians) cannot handle. Clinical role, Pg 606 |  |  |  |  |
| Luckett, R; Nassali, M; Melese, T; Moreri-Ntshabele, B; Moloi, T; Hofmeyr, G J; Chobanga, K; Masunge, J; Makhema, J; Pollard, M; Ricciotti, H A; Ramogola-Masire, D; Bazzett-Matabele, L | 2021 | Obstetrician/Gynaecologists | Case-study |  |  | “In the fifteen years preceding the establishment of Botswana’s first medical school, approximately 1000 Ba tswana were sponsored for undergraduate medical education abroad, but only 10% returned.” Introduction, Pg 2 |  |  |
| Lyon, Camila B; Merchant, Amina I; Schwalbach, Teresa; Pinto, Emilia F V; Jeque, Emilia C; McQueen, K A Kelly | 2016 | Aenesthesiologists | Qualitative | Physician anesthesiologists are found only in the central (level 4) and provincial (level 3) hospitals. The first referral hospitals that should have capacity to perform emergency surgery according to the WHO are staffed by technicians only. Results, Pg 1635 |  | “lack of teachers, lack of medical student interest in and exposure to anesthesia, need for more schools, low allocation to anesthesia from the list of available specialist prospects by MOH, and low public payments to anesthesiologists.” Abstract | for the population of 2.3 million people, only 40 OBGYNs are practicing clinically in-country. Of these 40 OBGYNs, only 12 practice in the public sector where a majority of the population seeks care.” Introduction, Pg 2 |  |
| Mandeville, Kate L; Hanson, Kara; Muula, Adamson S; Dzowela, Titha; Ulaya, Godwin; Lagarde, Mylène | 2017 | Medical specialty students | Quantitative |  | Expanding specialty training in Malawi is more cost-effective than training outside Malawi. At least two years of mandatory service would be more cost-effective, with five years adding the most value in terms of doctor-years. After 40 years of expanded specialty training in Malawi, the medical workforce would be over fifty percent larger with over six times the number of specialists compared to current trends | With regard to specialty training, our reliance on tuition fees may have underestimated the true cost of providing training. Larger numbers of registrars are likely to be easily absorbed by the extensive medical educational system in South Africa, whereas more teaching staff would be required in Malawi (this would be the main associated cost as most specialties are taught via apprenticeship-style training in central hospitals). These posts are likely to be filled by expatriate doctors until sufficient Malawian specialists were trained, with expenses covered by the Malawian government and development partners.” Discussion, Pg 94 |  |  |
| Mandeville, Kate L; Ulaya, Godwin; Lagarde, Mylène; Muula, Adamson S; Dzowela, Titha; Hanson, Kara | 2016 | Medical specialty students | Quantitative |  | Despite evidence that specialty training is highly sought after, Malawian junior doctors would not accept all types of training. Doctors preferred timely training outside of Malawi in core specialties (internal medicine, general surgery, paediatrics, obstetrics & gynaecology). Specialty preferences are particularly strong, with most junior doctors requiring nearly double their monthly salary to accept training all in Malawi and over six-fold to accept training in ophthalmology (representing a bundle of unpopular but priority specialties). Abstract | A lack of speciality training opportunities contributes to doctors migrating outside of their countries of origin |  |  |
| Marathe, Shweta; Hunter, Benjamin M; Chakravarthi, Indira; Shukla, Abhay; Murray, Susan F | 2020 | All specialties | Mixed-methods |  | “...a restratification taking place within India’s medical profession is one which appears to favour senior hospital specialists and ‘star doctors’ who contribute to the corporates’ brands and patient recruitment while the status of senior general practitioners has diminished.” Conclusion, Pg 8 |  | “Participation in networks of cash- for- referrals, referred to as ‘cut practices’, has been long standing within Maha rashtra’s healthcare system, encouraged by the financial strains on smaller private providers. Now the incentives are for practitioners to by- pass smaller hospitals and refer their patients to specialists in the larger, corporate hospi tals in search of more substantial commissions.” Results, Pg 6 |  |
| McKenna, M; Chen, T; McAneney, H; Membrillo, M A V; Jin, L; Xiao, W; Peto, T; He, M; Hogg, R; Congdon, N | 2018 | Ophthalmology | Quantitative |  | Non-medical graders can achieve high levels of accuracy, whereas accuracy of trained rural ophthalmologists is not optimal. Abstract - Screening by centrally-located, non-medical graders offers many advantages in low resource settings over reliance on rural doctors. Abstract |  |  |  |
| Meara, John G; Leather, Andrew J M; Hagander, Lars; Alkire, Blake C; Alonso, Nivaldo; Ameh, Emmanuel A; Bickler, Stephen W; Conteh, Lesong; Dare, Anna J; Davies, Justine; Mérisier, Eunice Dérivois; El-Halabi, Shenaaz; Farmer, Paul E; Gawande, Atul; Gillies, Rowan; Greenberg, Sarah L M; Grimes, Caris E; Gruen, Russell L; Ismail, Edna Adan; Kamara, Thaim Buya; Lavy, Chris; Lundeg, Ganbold; Mkandawire, Nyengo C; Raykar, Nakul P; Riesel, Johanna N; Rodas, Edgar; Rose, John; Roy, Nobhojit; Shrime, Mark G; Sullivan, Richard; Verguet, Stéphane; Watters, David; Weiser, Thomas G; Wilson, Iain H; Yamey, Gavin; Yip, Winnie | 2015 | Surgery & Aenesthesia | Mixed-methods | Beyond a specific threshold of specialists, the gains in health benefits noted by a population are minimal. There is a recognised need for surgical procedures in a population of 100,000 that require a minimum number of specialists | Placing surgical specialist trainees in facilities they are less likely to work in when fully qualified could help improve the number of available surgeons in these areas. | “A fifth of the world’s specialist surgeons, a sixth of the world’s specialist anaesthesiologists, and a third of the world’s specialist obstetricians attend to the poorest half of the world’s population. Only 12% of the specialist surgical workforce practise in Africa and southeast Asia, where a third of the world’s population lives.” The surgical workforce, Pg 588 | “Specialist providers are often concentrated in urban areas, which have more surgical infrastructure and better-equipped tertiary care centres than do rural areas.” Surgical workforce, Pg 588-589 |  |
| Meliala, Andreasta; Hort, Krishna; Trisnantoro, Laksono | 2013 | All specialties | Mixed-methods |  | regulatory policies and financial incentives have not been effective in addressing the maldistribution of specialist doctors in a context of a growing private sector and predominance of doctors' income from private sources. Abstract |  | 65% and 80% of specialist doctors' income derives from private practice in non-state hospitals or private clinics. Despite regulations limiting practice locations to three, most specialists studied in a provincial capital city were working in more than three locations, with some working in up to 7 locations, and spending only a few hours per week in their government hospital practice. Abstract |  |
| Miotto, Bruno Alonso; Guilloux, Aline Gil Alves; Cassenote, Alex Jones Flores; Mainardi, Giulia Marcelino; Russo, Giuliano; Scheffer, Mário César | 2018 | All specialties | Quantitative | Public sector physicians were found to be younger (PR 0.84 [0.68-0.89]; PR 0.47 [0.38-0.56]), less experienced (PR 0.78 [0.73-0.94]; PR 0.44 [0.36-0.53]) and predominantly female (PR 0.79 [0.71-0.88]; PR 0.68 [0.6-0.78]) when compared to dual and private practitioners; their income was substantially lower than those working exclusively for the private (PR 0.58 [0.48-0.69]) and mixed sectors (PR 0.31 [0.25-0.37]). Conversely, physicians from the private sector were found to be typically senior (PR 1.96 [1.58-2.43]), specialized (PR 1.29 [1.17-1.42]) and male (PR 1.35 [1.21-1.51]), often working less than 20 h per week (PR 2.04 [1.4-2.96]). Dual practitioners were mostly middle-aged (PR 1.3 [1.16-1.45]), male specialists with 10 to 30 years of medical practice (PR 1.23 [1.11-1.37]). | Simultaneous engagement with public and private sectors is not really regulated in Brazil |  | Specialists in Brazil are more likely to engage in dual practice than non-specialists |  |
| Miseda, Mumbo Hazel; Were, Samuel Odhiambo; Murianki, Cirindi Anne; Mutuku, Milo Peter; Mutwiwa, Stephen N | 2017 | All specialties | Mixed-methods |  | Norms and Standards Guidelines and CDH were used to assess needs. Interestingly, the two sources were often discordant. | In general, the findings reveal that on average, special ists’ skill gaps range between 85 and 62% when compared to the Norms and Standards and as perceived by the CDH respectively. However, on average, gynecologists, plastic surgeons, and Bachelor of Science in Nursing (BSN) have surpassed the required level by more than 100% when compared with the national guideline. Results, Pg 4 |  |  |
| Mock, Charles N; Donkor, Peter; Gawande, Atul; Jamison, Dean T; Kruk, Margaret E; Debas, Haile T; Adanu, Richard M K; Adhikari, Sweta; Ahimbisibwe, Asa; Alkire, Blake C; Babigumira, Joseph B; Barendregt, Jan J; Beard, Jessica H; Bergström, Staffan; Bickler, Stephen W; Chang, David; Charles, Anthony; Cherian, Meena; Coonan, Thomas; Desalegn, Dawit; De Vries, Catherine R; Dovlo, Delanyo; Dutton, Richard P; English, Mike; Farmer, Diana; Feres, Magda; Gathuya, Zipporah; Gosselin, Richard A; Higashi, Hideki; Horton, Sue; Hsia, Renee; Johansson, Kjell Arne; Johnson, Clark T; Johnson, Timothy R B; Joshipura, Manjul; Kassebaum, Nicholas J; Laxminarayan, Ramanan; Levin, Carol; Lofberg, Katrine; Lozo, Svjetlana; Mabweijano, Jackie; McCord, Colin; McPake, Barbara; McQueen, Kelly; Meara, John G; Mkandawire, Nyengo; Morgan, Mark A; Bedane, Mulu Muleta; Nandi, Arindam; Niederman, Richard; Noormahomed, Emilia; Nuevo, Florian R; Ogunbodede, Eyitope; Ohene-Yeboah, Michael; Olson, Zachary; Ottaway, Andrew; Ozgediz, Doruk; Pereira, Caetano; Polan, Mary Lake; Prajna, N Venkatesh; Price, Raymond R; Prinja, Shankar; Ravilla, Thulasiraj D; Hicks, Eduardo Romero; Russell, Sarah; Schecter, William P; Sitkin, Nicole; Sleemi, Ambereen; Spiegel, David; Shrime, Mark G; Srinivasan, Sathish; Stergachis, Andy; Thind, Amardeep; Verguet, Stéphane; Vincent, Jeffrey R; Vlassoff, Micahel; Von Schreeb, Johan; Vos, Theo; Weiser, Thomas G; Wilson, Iain H; Zakariah, Ahmed | 2015 | Surgery & Aenesthesia | Qualitative | First, provision of essential surgical procedures would avert about 1·5 million deaths a year, or 6-7% of all avertable deaths in low-income and middle-income countries. Second, essential surgical procedures rank among the most cost effective of all health interventions. The surgical platform of the first-level hospital delivers 28 of the 44 essential procedures, making investment in this platform also highly cost effective. | Task shifting to non-specialists proposed as a more attainable means of improving access to surgery |  |  |  |
| Naidu, Priyanka; Fagan, Johannes J; Lategan, Carina; Devenish, Liam P; Chu, Kathryn M | 2020 | Surgery & Aenesthesia | Quantitative |  | Doctors from SSA countries that do not have their own training programs have four options for surgical training: 1) programs located in high-income countries (HICs), 2) programs located in SSA that are funded and supported through HIC partners, 3) programs located in SSA run by regional accreditation bodies, or 4) university-based surgical programs in another SSA country (pag.1208). The four available training modalities have different retention rates, from very low for those programmes in HICs, to higher if organised in the receiving country. |  |  |  |
| Nigenda, Gustavo; Muños, José Alberto | 2015 | All specialties | Quantitative |  | Perceived weak regulation of medical training in Mexico. Specialist physicians in Mexico are predominantly trained at public sector health institutions. |  | Feeble connection between training of specialists and labour market demand.  Specialist doctors in Mexico currently represent more than 50% of doctors hired in public institutions. This abundant presence is a reflection of a health care model dominated by hospital. Interesting idea of specialists as an elite controlling the governance of their profession by participating simultaneously in public and private institutions. care services and the lack of human resources for health planning. |  |
| Nikoloski, Zlatko; Albala, Sarah; Montero, Andres Madriz; Mossialos, Elias | 2021 | All specialties | Quantitative | “The density of GPs and specialist physicians was negatively associated with amenable mortality rate. More specifically, a unit increase in the density of GPs and specialist physicians was associated with a 0.6% and 0.5% reduction in the overall amenable mortality, respectively (pag.5 and Tab.3). |  |  | Many specialists don’t do clinical activity in the public, or do office work. Moving them to clinical employment might have additional impact on reduction of amenable mortality. |  |
| Noormahomed, Emilia Virginia; Mocumbi, Ana Olga; Preziosi, Michael; Damasceno, Albertino; Bickler, Stephen; Smith, David M; Funzamo, Carlos; Aronoff-Spencer, Eliah; Badaró, Roberto; Mabila, Francisco; Bila, David; Nguenha, Alcido; Do Rosário, Virgilio; Benson, Constance A; Schooley, Robert T; Patel, Sam; Ferrão, Luis Jorge; Carrilho, Carla | 2013 | All specialties | Case-study | Developing research, providing mentorship particularly in bio-clinical science.  Creation of regional networks for mutual support.  Development of projects and attracting research funds focussing on local health priorities.   Development of research ethics institutional capacity |  |  |  |  |
| Oman, Kimberly M; Moulds, Robert; Usher, Kim | 2009 | All specialties | Qualitative |  | “Although it was believed that offering training in the Pacific and awarding a local specialist qualification not recognized elsewhere would improve retention in the public sectors [12], within a few years, many doctors who had started training were leaving the public system to enter local private practice or to migrate overseas.” Pag.2 Policies to provide localised diplomas (not valid internationally) do not really stop migration |  | While private practice was much more lucrative than public-sector work, this was not mentioned as the main motivation for any of the doctors. Doctors appreciated being able to control their hours, spending more time with their patients, having clinical autonomy and logistical support, with the main trade-offs being the loss of opportunities for further specialist training, and missing the "rich" and varied work in the public hospital. |  |
| Oman, Kimberly; Rodgers, Elizabeth; Usher, Kim; Moulds, Robert | 2012 | All specialties | Mixed-methods |  | With support from the Australian government, regional postgraduate specialist training was established in the late 1990s in Fiji, a small developing Pacific Island nation. This was done in order to address the failure of most overseastrained Pacific Island specialists to return to or remain in the Pacific, along with an ongoing dependence on expatriates for specialist services in the region |  | Career pathways and advancement are areas that require particular attention. The early years of new courses are likely to be full of uncertainties, and the quality of the graduates will initially be unclear. There is a very real risk that until they have “proven themselves”, graduates may be undervalued compared to overseas-trained specialists or expatriate recruits. | The policy of regional markets to allow or ban recruitment from lower-income countries can have an effect on migraton |
| Pei, Y Veronica; Xiao, Feng | 2011 | Emergency Medicine | Qualitative |  | Still underdeveloped in China, EM specialists are recruited from non-specialists doctors to deliver emergency care in their own departments. With a view to attracting customers under the new health insurance system, hospitals have built new EM departments and actively advertise their services |  |  |  |
| Quintao, V C; Concha, M; Argüello, L A S; Cavallieri, S; Cortinez, L I; de Sousa, G S; Clemente, M M M; Carlos, R V; Rodríguez, J M; Gutiérrez, K; Jablonka, D H; García-Marcinkiewicz, A G | 2024 | Aenesthesiologists | Qualitative |  | While Anesthesiology is a specialty recognized by the Brazilian Medical Association (AMB), Pediatric Anesthesiology is not. Out of the 46 specialties recognized by the AMB, 17 (37%) are pediatric-related, while none of them are in surgical areas | There is a critical shortage of anaesthesiologists in Brazil. The proportion of anesthesia providers to surgeons and/or other medical services is currently 1 to 5. (….) Brazil has approximately 14 anesthesiologists for every 100 000 inhabitants, with significant variability across different regions in the country.9 According to the Lancet commission, they recommend 20 anesthesiologists for every 100 000 inhabitants. In Chile, There are 10 857 general anesthesiologists certified by the National Council for Certification of Anesthesiology (CNCA), however only 272 are certified pediatric anesthesiologists who have a university degree in the subspecialty |  |  |
| Rao, Krishna D; Ryan, Mandy; Shroff, Zubin; Vujicic, Marko; Ramani, Sudha; Berman, Peter | 2013 | Medical specialty students | Quantitative |  | Incentivizing doctors to serve in rural areas is challenging and expensive. The supply of both student and in-service doctors for rural posts was not responsive to increases in salary, particularly at lower salary levels. “Strategies that offer ‘packages of incentives’ that address both the professional and personal needs of doctors can improve their rural recruitment to a certain extent. Elements of this package should include, substantial salary increases, improvements to the living environment, provision of good children’s education, and where possible, reservation of seats for specialist training.” (Rao et al., 2013, p. 9) |  |  |  |
| Rispel, Laetitia Charmaine; Ditlopo, Prudence; White, Janine Anthea; Blaauw, Duane | 2019 | Medical specialty students | Mixed-methods |  |  |  |  | Declared reasons for choosing the medical profession did not vary substantially from those for other professions |
| Riva, J; Calviño, J; Bouchacourt, J P; Turconi, L; Cavalleri, F; Caetano, N N; Enriquez, L; Tonelotto, B; Lema, G; Motta, P | 2024 | Aenesthesiologists | Mixed-methods | Most anaesthesiologists worked on general anaesthesia, rather than focussing in CVA | Most countries in LAC do not have formal training in this subspecialty; Only in Mexico there is a council for this subspecialty, in other they are part of the main anestesionlogy council |  |  |  |
| Robertson, Faith C; Gnanakumar, Sujit; Karekezi, Claire; Vaughan, Kerry; Garcia, Roxanna M; Abou El Ela Bourquin, Bilal; Derkaoui Hassani, Fahd; Alamri, Alexander; Mentri, Nesrine; Höhne, Julius; Laeke, Tsegazeab; Al-Jehani, Hosam; Moscote-Salazar, Luis Rafael; Al-Ahmari, Ahmed Nasser; Samprón, Nicolás; Stienen, Martin N; Nicolosi, Federico; Fontoura Solla, Davi J; Adelson, P David; Servadei, Franco; Al-Habib, Amro; Esene, Ignatius; Kolias, Angelos G | 2020 | Surgery & Aenesthesia | Mixed-methods | An excess of 23,000 more neurosurgeons are needed in LMICs to address the 5 million essential neurosurgical cases that go untreated each year.6 These untreated cases predominantly include traumatic brain injury but also incorporate stroke, hydrocephalus, tumors, epilepsy, and infection.4-9 To address these issues, a systems-level approach is required. | A list of suggested policies and interventions are provided, from opportunity to do research, further education, and creation of subspecialties |  |  |  |
| Ruggunan, Shaun D; Singh, Suveera | 2013 | Histopathology | Qualitative | Histopathologists and medical laboratory personnel are key for the functioning of the healthcare – exams serving provision of differentiated care – but they are supposedly ‘invisible’ to the population, and do not receive recognitions |  |  | Many pathologist ‘switch sector’ and move permanently to the private sector because of better pay |  |
| Russo, Giuliano; Cassenote, Alex J Flores; Guilloux, Aline G Alves; Scheffer, Mário César | 2020 | All specialties | Mixed-methods | Specialists provide clinical services simultaneously in public and private sector |  |  | Local market conditions are one of the determinants of dual practice’s forms and participation in the countries – specialists have greater access to dual practice | Specialists tend to concentrate in urban areas (deployment capture), but in more diversified markets (like Maputo) there is also a strong presence of generalist and junior doctors catering for hospital needs (night shifts) |
| Russo, Giuliano; Gonçalves, Luzia; Craveiro, Isabel; Dussault, Gilles | 2015 | All specialties | Quantitative |  |  |  | Female doctors engage as much as their male peers in private practice, although overall they dedicate fewer hours to the profession, particularly in the public sector. | Overall, 52.3% of female physicians declared holding a specialty, as opposed to 76.3% of males, with the highest proportion recorded for Bissau (63%). Paediatrics, general practice and gynaecology were the most frequent specialties (8.5%, 6.5% and 5.2%, respectively); in total, female doctors represented 76.5% of paediatricians. By contrast, they were practically absent from surgery, orthopaedics, stomatology and otorhinolaryngology. |
| Russo, Giuliano; McPake, Barbara; Fronteira, Inês; Ferrinho, Paulo | 2014 | All specialties | Quantitative | A strong, stable PHC medical workforce is at the core of Brazil’s family health programme and progress towards UHC |  |  | Private medical schools have doubled the supply of medical doctors and students, but there are doubt they truly benefit all the specialties. However, the evidence is that also public medical schools are unable to train PHC-minded doctors. | Most of the medical students are today enrolled in private medical colleges. Students who declared opting for PHC specialty were mostly from state secondary schools, and less-well off family background. Not being from an affluent family, not having attended private secondary school, having trained in a medical school outside the South East, not coming from an affluent family, not having attended a private secondary school, and not having a high valuation for career development plans were significant predictors of graduates’ willingness to practice in PHC. |
| Scheffer, Mário C; Guilloux, Aline G A; Matijasevich, Alicia; Massenburg, Benjamin B; Saluja, Saurabh; Alonso, Nivaldo | 2017 | Surgery & Aenesthesia | Quantitative | Operation is key to save lives, and should be seen as keyway to reduce burden of disease for many conditions Brazil’s recent decrease in maternal mortality has been associated with the increase in surgical workforce. Aenestesiologists are more scarce than surgeons, and this might be a stumbling block for efficient surgery teams in the future |  | As a nation, Brazil meets the minimum requirement of 20–40 SAO per 100,000 population as recently recommended by The Lancet Commission on Global Surgery. But inequal distribution North-South and urban-rural is the key issue |  | Very male dominated, and located predominantly in urban areas. Most of the surgical workforce graduated from public medical schools – due to the only recent introduction of private ones. |
| Schluger, Neil W; Sherman, Charles B; Binegdie, Amsalu; Gebremariam, Tewedros; Kebede, Dawit; Worku, Aschalew; Carter, E Jane; Brändli, Otto | 2018 | Pneumologists | Case-study |  | Clinical training gas the easy part through USA volunteers travelling to the field, while creating a path to progression for specialists was the stumbling block. However, Specialist training from the outside is limited in scope, and not really sustainable | In Ethiopia (like in the rest of LMICSs) the vast majority of doctors are generalists, with very few specialists spread across a handful of specialties |  |  |
| Sriram, Veena; Baru, Rama; Bennett, Sara | 2018 | All specialties | Qualitative | Reflections on multiple contributions of specialists to systems. However, in some instances, patients try to bypass the PHC filter and access specialists directly, undermining the functions of the system. The authors believe that specialists contribution to systems are shaped by market forces and the strength of PHC. Specialists also perform functions that are considered fundamental for UHC | The number of specialties in LMICs is expanding because of specialists’ influence on policy and policy-makers. Three policies questions need to be responded: (1) how many specialists to train (2) how to link specialists to systems’ development, and (3) how to develop governance and institutions for specialties |  | Market forces can make it difficult to deploy and retain specialists where they are needed the most. But the demand and supply of specialists is likely to contibue to grow in LMICs |  |
| Sriram, Veena; Bennett, Sara | 2020 | Emergency Medicine | Qualitative | Medical specialisation is actually shaping the organisation of health systems in LMICs. Poor links between the tertiary care and the rest of the system | Regulation of specialties and sub-specialties in LMICs is underdeveloped. In India, there are two paths to recognise specialties, but they are not transparent. There are government, medical and board actors influencing regulation of specialties in India. The multiplicity of actors and influences has created confusion, and a vacuum of responsibilities and accountability |  |  |  |
| Tyson, Anna F; Msiska, Nelson; Kiser, Michelle; Samuel, Jonathan C; Mclean, Sean; Varela, Carlos; Charles, Anthony G | 2014 | Surgery & Aenesthesia | Quantitative |  | In Malawi, clinical officers are given a 3-year long training + interneship, and then they are licensed to work as surgeons and anaesthesiologists. Surgical task-shifting for non-physicians |  |  |  |
| van Heemskerken, P; Broekhuizen, H; Gajewski, J; Brugha, R; Bijlmakers, L | 2020 | Surgery & Aenesthesia | Systematic review |  | Lack of regulation is hampering the operationalisation of surgical task-shifting. As many as 14 types of barriers were identified to operationalise surgical task-shiting | 5 billion people lack access to safe surgery (quoting from The Lance Commission on Global Surgery 2030) |  |  |
| van Rensburg, Bernard Janse; Kotze, Carla; Moxley, Karis; Subramaney, Ugasvaree; Zingela, Zukiswa; Seedat, Soraya | 2022 | Psychiatry | Quantitative |  | Psychiatrists are medical specialists who require at least 13 years of rigorous training to qualify as independent practitioners (de Kock and Pillay, “2017; Wishnia et al., 2019). In South Africa, this includes a 6-year medicine undergraduate programme, 2 years of internship in a public hospital, 1 year of community service in a public hospital and then at least 4 years of registrar (or resident) training in a particular field of specialization also within the public sector.” (Janse van Rensburg et al., 2022, p. 492) | Psychiatrist in ZA are few (1.5 per 100,000), working in the private sector, and concentrated in Gauteng and Cape Town, now female, but still white | A substantial proportion of psychiatrists (80%) work full-time in the private sector. Those in public, are joint-appointment academics, or on RWOPS contracts, reducing dramatically the availability of psychiatric services in public. |  |
| Vilaly, Mohamed Abd salam El; Jones, Maureen A; Stankey, Makela Cordero; Seyi-Olajide, Justina; Onajin-Obembe, Bisola; Dasogot, Andat; Klug, Stefanie J; Meara, John G; Ameh, Emmanuel A; Osagie, Olabisi O; Juran, Sabrina | 2021 | Surgery & Aenesthesia | Quantitative | Referral district hospitals. “In sub-Saharan Africa, the burden of surgical disease is estimated between 257.8 and 294.7 million people. The burden of surgical disease is estimated between 115.3 million and 131.8 million in children under 15 years of age.4 6 Almost 85% of children in LMICs will have a surgically treatable condition by the age of 15 years.1 6 7” (Vilaly et al., 2021, p. 2) |  | “Findings suggest that from less than 2% to 22.7%30.5% of Nigeria’s youth population resides within 2 hours of a health facility with a paediatric surgical and anaesthesia workforce.” (Vilaly et al., 2021, p. 1)  “Globally, approximately 5 billion people lack timely access to safe and affordable surgical care when needed.2 3 Of those, approximately 1.7 billion are children and adolescents and 453 million are children under 5 years of age.” (Vilaly et al., 2021, p. 1) |  |  |
| Vio, F | 2006 | All specialties | Case-study |  | Contracting of foreign specialists to fill vacancies outside Maputo |  | There is an oversupply of international specialists from the former soviet bloc that could be used to fill vacancies in LMIC, if salaries were internationally competitive. As the country develops economically, the attraction of more lucrative employment in urban areas’ private sector grows, making it harder to keep specialists in rural areas |  |
| Wu, Dan; Lam, Tai Pong; Lam, Kwok Fai; Zhou, Xu Dong; Sun, Kai Sing | 2017 | All specialties | Mixed-methods | After the 2009 healthcare reforms, China’s healthcare system is now strongly focussed on hospitals and specialists, and patients seek care there directly through personal connections - Guanxi. However, The practice of doctors generating revenues for the hospitals is seen as actually harming patients. | According to the reform creating hospital competitions, Specialists have to generate revenues for their hospitals by selling drugs and medical appointments |  |  |  |
| Zhang, Tao; Liu, Chaojie; Liu, Lingrui; Gan, Yong; Lu, Wei; Tao, Hongbing | 2019 | All specialties | Quantitative | According to the 2009 reforms, PHC should be the first point of contact with the health system, but patients (with insurance) end up selecting the hospital they want, and hospitals accept them as a way to generate their own revenues. A stark divide rich/poor has been created in accessing specialist services. |  |  |  | The poor in China predominatly access GPs, and the rich spcialists |
| Zhang, Yongjun; Huang, Lisu; Zhou, Xin; Zhang, Xi; Ke, Zheng; Wang, Zhaoxi; et al | 2019 | Paediatrics | Quantitative | Importance of paediatrics for China’s recent progress in infant health | Many paediatricians had only three years of training after secondary school. To mitigate scarcity of paediatricians, The Govt has decreased the entry requirements for paediatrists to increase numbers | There are in general few paediatricians in China, and their distribution is highly skewed in favour of the East Coast provinces | Unmet demand for paediatric services, as number of paediatric institutions has increased, but not specialists doctors |  |
