# Supplementary material for: Medical specialists in LMICs: a systematic review and best-fit framework synthesis of the evidence on their roles and contribution to health systems
Source: BMJ Glob Health. 2026 Jan 9;11(1):e018905. doi: 10.1136/bmjgh-2025-018905 (PMC12815179; doi:10.1136/bmjgh-2025-018905)
Supplement: online supplemental file 6 [file bmjgh-11-1-s006.docx]

**Appendix 6**

**Table S6: Tensions found in the literature on the role of specialists in LMICs**

| Domain | Tensions | Key references from this review |
| --- | --- | --- |
| Cost-effectiveness and equity of specialty services in LMICs | Since most of the burden of deaths and disability in LMICs are imputable to basic, preventable diseases, it would not be cost-effective to invest in specialty services.  Patients in LICs can develop complex disease too and have a right to find care for that disease.  A comprehensive health system must establish mechanisms to effectively address complex cases; failure to do so may erode patients’ trust in the system. | (Henry *et al.*, 2015; John G Meara *et al.*, 2015; Mock *et al.*, 2015; B A Miotto *et al.*, 2018) |
| Geographical location of specialists and specialised services | Most specialty healthcare services can only be provided in large specialised hospitals, often located in capital cities or urban areas.  Certain specialties (such as surgery and psychiatry) are needed to be located in rural areas, closer to communities in order to be effective. First-referral (district) hospitals are needed to guarantee access for all.  Most specialists are reluctant to be based in rural areas that typically offer lower quality of life. | (English *et al.*, 2024).  (Davies and Lund, 2017; Jacob, 2017).  (Gajewski *et al.*, 2020)  (Bolton *et al.*, 2023).  (Jeffries Mazhar *et al.*, 2024). |
| Specialists’ engagement with the private sector | Specialists contribute to the development of the private sector, therefore expanding supply and types of available services.  However, specialists’ simultaneous engagement in public and private services (dual practice) can impact negatively on availability and quality of public services. | (Russo *et al.*, 2014)  (Jan *et al.*, 2005; McPake *et al.*, 2013; Coveney, Musoke and Russo, 2023)  (Bruno Alonso Miotto *et al.*, 2018)  (Karekezi *et al.*, 2020) (Janse van Rensburg *et al.*, 2018).  (John G. Meara *et al.*, 2015). (English *et al.*, 2020). |
|  |  |  |
