# Supplementary material for: Medical specialists in LMICs: a systematic review and best-fit framework synthesis of the evidence on their roles and contribution to health systems
Source: BMJ Glob Health. 2026 Jan 9;11(1):e018905. doi: 10.1136/bmjgh-2025-018905 (PMC12815179; doi:10.1136/bmjgh-2025-018905)
Supplement: online supplemental file 7 [file bmjgh-11-1-s007.docx]

### BMJ Global Health Author Reflexivity Statement

Adapted from Morton, B., Vercueil, A., Masekela, R., Heinz, E., Reimer, L., Saleh, S., Kalinga, C., Seekles, M., Biccard, B., Chakaya, J., Abimbola, S., Obasi, A. and Oriyo, N. (2022), Consensus statement on measures to promote equitable authorship in the publication of research from international partnerships. Anaesthesia, 77: 264-276. <https://doi.org/10.1111/anae.15597>

| **Study conceptualisation** | |
| --- | --- |
| 1. How does this study address local research and policy priorities? | This study directly addresses the issue of specialist doctors in LMICs, providing evidence-based policy recommendations. |
| 1. How were local researchers involved in study design? | All the co-authors contributed to the study design. |
| **Research management** | |
| 1. How has funding been used to support the local research team(s)? | Funding from the UK MRC has been used to support the researchers in Brazil and in Mozambique. |
| **Data acquisition and analysis** | |
| 1. How are research staff who conducted data collection acknowledged? | All staff who conducted data collection are named as co-authors. |
| 1. How have members of the research partnership been provided with access to study data? | Yes |
| 1. How were data used to develop analytical skills within the partnership? | Junior members of the research team were provided training in systematic review methodology and referencing software. |
| **Data interpretation** | |
| 1. How have research partners collaborated in interpreting study data? | All contributed to interpretation and discussion of the findings. |
| **Drafting and revising for intellectual content** | |
| 1. How were research partners supported to develop writing skills? | All research partners contributed to drafting the manuscript, and given feedback on their contributions on writing skills in the English language. |
| 1. How will research products be shared to address local needs? | Once published, we will disseminate the paper and give presentations in Brazil and in Mozambique. |
| **Authorship** | |
| 1. How is the leadership, contribution and ownership of this work by LMIC researchers recognised within the authorship? | The senior researcher by Brazil (MS) is last author of the paper. All the other members have been given equal authorship positions. |
| 1. How have early career researchers across the partnership been included within the authorship team? | Junior researches (TW, RM) have been central to this study, conducting data collection, analysis, and writing up. |
| 1. How has gender balance been addressed within the authorship? | The study team includes 4 female researchers (VS, TW, RM and AM), and two male one (GR and MS). |
| **Training** | |
| 1. How has the project contributed to training of LMIC researchers? | LMIC researchers received direct training in systematic review methodology, access to databases, and use of referencing software for organising and sharing bibliographic references. |
| **Infrastructure** | |
| 1. How has the project contributed to improvements in local infrastructure? | The project did not include an infrastructure component. |
| **Governance** | |
| 1. What safeguarding procedures were used to protect local study participants and researchers? | Safeguarding procedures were incorporated throughout the systematic review to protect both the individuals represented in the included studies and the researchers conducting the analysis. Only studies that demonstrated appropriate ethical approval and participant protections—such as informed consent, confidentiality measures, and safe interview practices. All extracted data were anonymised and contextualised to avoid inadvertently identifying individuals or facilities. For researchers, especially those based in LMIC settings where discussions about specialist doctors can be professionally sensitive, the project provided secure data-sharing platforms, clear authorship options that protected anonymity, and access to supervisory support for handling potentially distressing accounts of working conditions. |
